# Highly connected 3D chromatin networks established by an oncogenic fusion protein shape tumor cell identity

**DOI:** 10.1126/sciadv.abo3789

**Published:** 2023-03-31

**Authors:** Rajendran Sanalkumar, Rui Dong, Lukuo Lee, Yu-Hang Xing, Sowmya Iyer, Igor Letovanec, Stefano La Rosa, Giovanna Finzi, Elettra Musolino, Roberto Papait, Ivan Chebib, G. Petur Nielsen, Raffaele Renella, Gregory M. Cote, Edwin Choy, Martin Aryee, Kimberly Stegmaier, Ivan Stamenkovic, Miguel N. Rivera, Nicolò Riggi

**Affiliations:** ^1^ Experimental Pathology Service, Lausanne University Hospital and University of Lausanne, Lausanne, Switzerland.; ^2^ Department of Pathology and Cancer Center, Massachusetts General Hospital, Charlestown, MA, USA.; ^3^ Department of Histopathology, Central Institute, Valais Hospital, Sion, Switzerland.; ^4^ Institute of Pathology, Lausanne University Hospital and University of Lausanne, Lausanne, Switzerland.; ^5^ Pathology Unit, Department of Medicine and Surgery, University of Insubria, Varese, Italy.; ^6^ Department of Pathology, ASST Sette Laghi, Varese, Italy.; ^7^ Department of Biotechnology and Life Sciences, University of Insubria, Varese, Italy.; ^8^ IRCSS Humanitas Research Hospital, via Manzoni 56, 20089 Rozzano, Milan, Italy.; ^9^ Department of Pathology and Cancer Center, Massachusetts General Hospital and Harvard Medical School, Boston, MA, USA.; ^10^ Department Woman-Mother-Child, Division of Pediatrics, Lausanne University Hospital and University of Lausanne, Lausanne, Switzerland.; ^11^ Department of Medicine, Division of Hematology and Oncology, Massachusetts General Hospital, Boston, MA, USA.; ^12^ Dana-Farber/Boston Children’s Cancer and Blood Disorders Center, Boston, MA, USA.; ^13^ Broad Institute, Cambridge, MA, USA.

## Abstract

Cell fate transitions observed in embryonic development involve changes in three-dimensional genomic organization that provide proper lineage specification. Whether similar events occur within tumor cells and contribute to cancer evolution remains largely unexplored. We modeled this process in the pediatric cancer Ewing sarcoma and investigated high-resolution looping and large-scale nuclear conformation changes associated with the oncogenic fusion protein EWS-FLI1. We show that chromatin interactions in tumor cells are dominated by highly connected looping hubs centered on EWS-FLI1 binding sites, which directly control the activity of linked enhancers and promoters to establish oncogenic expression programs. Conversely, EWS-FLI1 depletion led to the disassembly of these looping networks and a widespread nuclear reorganization through the establishment of new looping patterns and large-scale compartment configuration matching those observed in mesenchymal stem cells, a candidate Ewing sarcoma progenitor. Our data demonstrate that major architectural features of nuclear organization in cancer cells can depend on single oncogenes and are readily reversed to reestablish latent differentiation programs.

## Teaser

Remodeling of nuclear organization by an oncogenic transcription factor determines tumor cell identity.

## INTRODUCTION

Perturbations in chromatin regulation are widely recognized to play a critical role in cancer development (*1*–*3*). Genomic technologies for the analysis of chromatin states have led to major advances in our understanding of the cancer epigenome, including the identification of key regulatory elements driving oncogenic and cell identity programs (*4*–*6*). More recently, genome-wide approaches to profile three-dimensional (3D) genome organization have shown the importance of looping networks and large-scale compartmentalization in gene regulation (*7*–*11*). However, the relationship between these 3D features, oncogenic pathways, and tumor cell identity remains incompletely understood.

The human genome is functionally partitioned into multiple layers of nuclear organization. Most of the cis-regulatory transcriptional control occurs inside insulated chromatin neighborhoods, commonly referred to as topologically associating domains (TADs) (*12*–*14*). The stability of these self-interacting domains ensures precise spatiotemporal gene expression patterns by serving as a barrier to ectopic enhancer-promoter interactions. Multiple TADs that share similar epigenetic and transcriptional profiles further organize themselves into compartments, higher-order chromatin structures that segregate the genome into megabase-sized active (A) or repressed (B) regions (*15*, *16*). The compartmental organization of each cell is influenced by its transcriptional output, with compartments sharing similar activity interacting preferentially with each other in distinct subnuclear localizations (*8*, *17*).

Our current insight into the topological alterations in cancer, and their contribution to the malignant phenotype, stems primarily from comparisons between cancer cells and their normal counterparts (*18*–*21*). As a consequence, many of the better-characterized differences in genome folding arise from structural DNA abnormalities in cancer cells and their effects on 3D nuclear architecture (*22*, *23*). However, it is becoming increasingly recognized that alterations in nuclear organization within tumor cells may also be orchestrated by more dynamic epigenetic events. A/B compartmental changes can be observed in different cancer types, where they may play causative roles in tumor development in association with transcriptional alterations favoring oncogenic programs (*21*, *24*–*26*). For example, recent studies have shown that A/B compartmentalization may be altered in cancer cells, giving rise to new intermediate compartments at their interface that are involved in controlling tumor evolution (*24*). Specific epigenetic regulators may also play major roles in the topological changes observed during normal development and cancer.

Transcription factors (TFs) with the ability to modify chromatin activation states can drive conformational changes, either at the level of compartmentalization or by reshaping TADs themselves along with intra-TAD interactions (*27*–*30*). As many TFs tend to act through distal regulatory elements in the genome, the possibility to integrate 3D looping data with chromatin occupancy profiles also provides a powerful means to define direct transcriptional targets and their relationship to topological changes (*31*). For example, during physiological B cell differentiation, the lineage-specifying TF Paired box 5 (PAX5) has been shown to organize the genomic architecture in a way that helps induce expression programs that maintain lineage fidelity (*32*). Similar regulatory events driven by oncogenic TFs may also be involved in cancer development, enabling tumor cells to establish oncogenic expression programs and elude differentiation barriers.

In this study, we used the Ewing’s sarcoma EWS-FLI1 oncogenic fusion protein as a paradigm to investigate the impact of a single TF on 3D chromatin conformation in tumor cells and its direct relationship to gene regulation and cell fate. Ewing’s sarcoma (EwS), the second most common pediatric bone malignancy, features a low mutational burden and the presence of a fusion between *EWSR1* and a gene encoding one of the ETS family of TFs, most frequently *FLI1* (*33*, *34*). EWS-FLI1 is recognized as the main oncogenic driver of this malignancy and has been shown to behave as a powerful aberrant TF that remodels the epigenetic landscape of permissive cells, resulting in their transformation (*35*–*38*). The phase transition properties of the prion-like domain of EWSR1 enable EWS-FLI1 to operate as a pioneer factor at GGAA repeats, where it induces de novo formation of active enhancers (*39*). This notion is also supported by EWS-FLI1 depletion studies, which show loss in chromatin activity at GGAA repeats following the knockdown of the fusion, alongside a marked reduction of the corresponding target gene expression (*36*). Site-specific epigenetic silencing of GGAA repeats using CRISPR-KRAB or ZNF-KRAB technologies also leads to enhancer decommissioning and target gene repression (*40*, *41*). Given that EWS-FLI1 remains, to date, the only TF able to bind and activate these repetitive regions, these data confirm the direct role of these regulatory elements in maintaining the transcriptional program induced by EWS-FLI1 in EwS. In contrast, EWS-FLI1 can also act as a repressor at canonical nonrepetitive GGAA ETS binding sites by displacing wild-type ETS factors from enhancers that are involved in mesenchymal differentiation programs (*36*).

We now show that, in keeping with its powerful role in regulating chromatin states, EWS-FLI1 is also a major determinant of 3D chromatin conformation in EwS. EWS-FLI1 converts repetitive GGAA genomic regions into highly connected chromatin interaction hubs (hereafter EWS-FLI1 enhancer hubs) covering/bridging megabase-sized genomic regions and enabling oncogenic programs. EWS-FLI1 depletion leads to the loss of EWS-FLI1–bound enhancer hubs and the establishment of a different gene-regulatory architecture that mirrors the topological and transcriptional states of primary mesenchymal stem cells (MSCs). In aggregate, our observations support the notion that tumor cells may display a dynamic and reversible nuclear organization and emphasize the critical role of TFs in modeling chromatin topology and activity.

## RESULTS

### The active enhancer network of EwS tumor cells is dominated by EWS-FLI1–centered chromatin loops forming highly connected interaction hubs

The ability of EWS-FLI1 to bind GGAA microsatellite repeats and convert them into de novo active enhancers has been demonstrated in EwS cell lines and primary tumor models (*36*). Although these distal regulatory elements are required for maintaining oncogenic properties of EwS cells and restricting their differentiation, only a fraction of EWS-FLI1 target genes have been identified so far, limiting our understanding of the direct mechanisms of action of the fusion protein and their impact on the tumor biology. To obtain a more comprehensive picture of the EWS-FLI1 target gene repertoire, we performed Histone H3 lysine 27 acetylation (H3K27ac) antibody-mediated HiChIP in two well-established EwS cell lines, A673 and SKNMC (table S1). This technology couples HiC with chromatin immunoprecipitation to enrich interactions between H3K27ac marked regions and therefore provides a detailed map of all active chromatin loops in a given cell type (*42*).

**Fig. 1.**
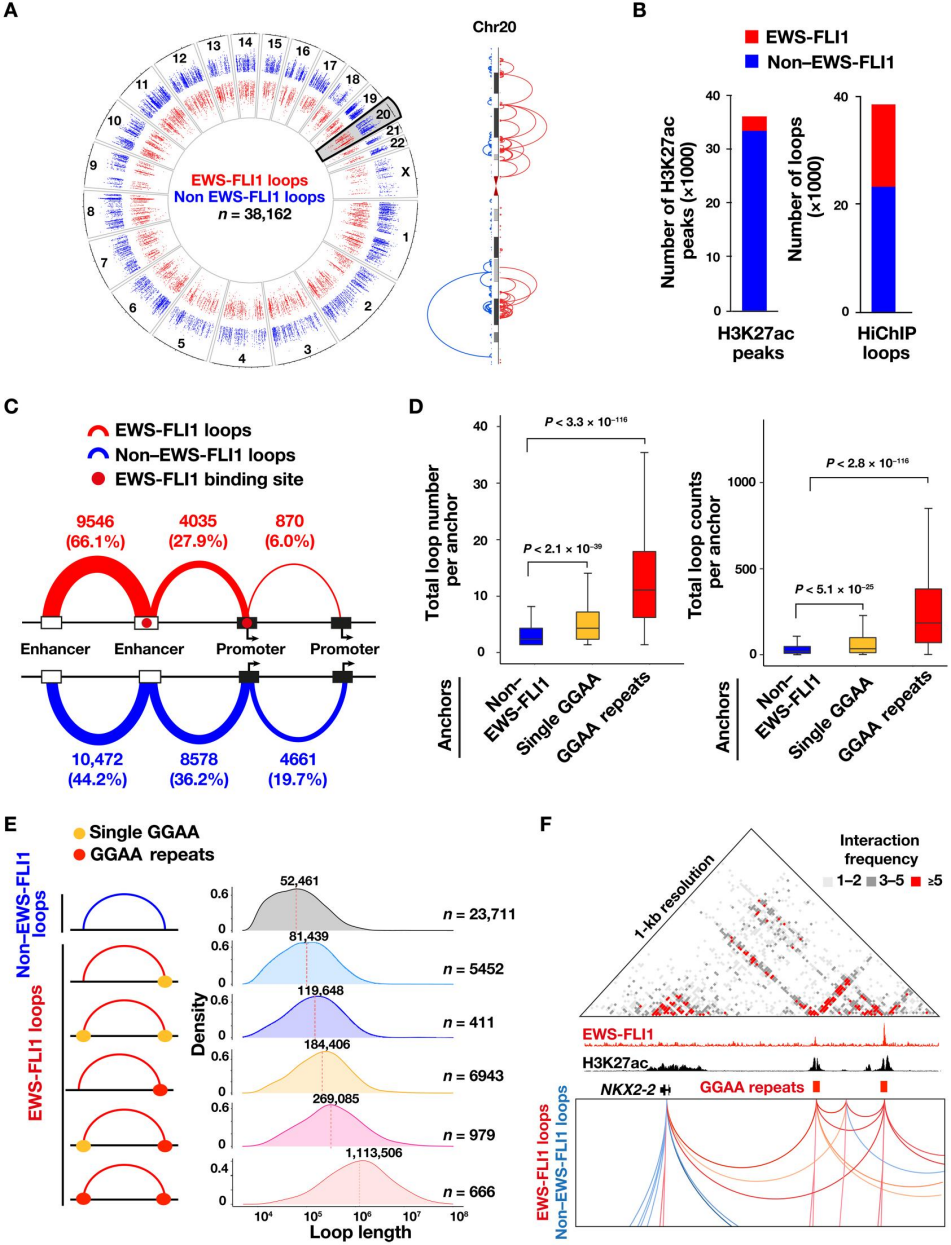
EWS-FLI1–associated enhancer hubs dominate the active chromatin landscape of EwS tumor cells. (**A**) Left: Circos plot depicts the genome-wide pattern of H3K27ac-mediated HiChIP anchor loops that are categorized as EWS-FLI1–associated (red) or EWS-FLI1–independent (non–EWS-FLI1, blue) in A673 cells. Loops are organized according to their length. Right: A representative chromosome (chr20) illustrates loops associated or not with EWS-FLI1. (**B**) Bar plots showing the number of H3K27ac ChIP-seq peaks (left) and chromatin loops (right) associated with EWS-FLI1 binding or independent of the fusion protein in A673 cells. (**C**) Distribution of EWS-FLI1–associated or EWS-FLI1–independent loops in A673 cells. The total number and percentage of each category (enhancer-enhancer, enhancer-promoter, or promoter-promoter) is shown for both EWS-FLI1 (red) and non EWS-FLI1 (blue) loops. (**D**) Box plots depicting total loop numbers (left) and loop counts (right) per HiChIP anchor in A673 cells based on the underlying DNA motif, defined as GGAA repeats, single GGAA, or unrelated genomic sequences. The significance in comparison among anchors was calculated by unpaired two-sided *t* test. (**E**) Loop length distribution for both EWS-FLI1–associated and EWS-FLI1–independent loops. EWS-FLI1–associated loops are further subdivided on the basis of the presence and distribution of GGAA repeat or single GGAA motifs on either side of the loop anchors. The total number of loops for each category is shown on the right. (**F**) Image of the *NKX2.*2 genomic locus, showing raw HiChIP interaction frequency (top), H3K27ac signal and EWS-FLI1 occupancy (middle), and the associated chromatin interactions (bottom) in A673 cells. Loops are color-coded according to their association (red) or not (blue) with EWS-FLI1.

To annotate EWS-FLI1 loops, we integrated HiChIP data with existing chromatin states and EWS-FLI1 occupancy profiles for A673 and SKNMC cells and identified long-range chromatin contacts that are anchored by at least one EWS-FLI1 binding site. We generated HiChIP maps from two independent replicates for A673 cells and found high reproducibility (at least 95% shared loops; fig. S1A). One HiChIP map was generated for SKNMC cells. For A673 cells, HiChIP interactions were called from combined loops from both replicates, and loops identified in both replicates and showing a signal greater than five counts in one of the two replicates were selected for further analysis. Our analysis yielded a total of 38,162 and 31,537 high-confidence, H3K27ac-anchored chromatin interactions in A673 and SKNMC cells, respectively, of which 14,451 and 14,619 were associated with at least one EWS-FLI1 binding site. Both EWS-FLI1 and non–EWS-FLI1 loops were widely distributed across the genome (Fig. 1A and fig. S1B). However, despite the fact that EWS-FLI1 peaks are a minority of all H3K27ac ChIP-seq peaks (7.3% in A673 and 8.4% in SKNMC), EWS-FLI1–anchored loops accounted for almost 40% of all chromatin interactions in both cell lines. The fusion protein thus has a dominant role in the long-range regulatory landscape of EwS cells (Fig. 1B and fig. S1C). This includes connections involving all categories of regulatory elements, enhancer-enhancer (e-e), enhancer-promoter (e-p), and promoter-promoter (p-p) in a proportion similar to non–EWS-FLI1 loops except for a small relative increase in e-e connections and a decrease in p-p connections (Fig. 1C and fig. S1D).

Next, we sought to compare the looping patterns associated with the fusion protein binding sites. To this end, all H3K27ac anchors were grouped on the basis of the presence or absence of EWS-FLI1 occupancy (*36*) and its underlying binding motif (single or repeat GGAA). Notably, EWS-FLI1–bound anchors exhibited a higher number of connected loops and were associated with increased contact intensity compared to anchors lacking EWS-FLI1 occupancy (Fig. 1D and fig. S1E). This trend was even more evident for loops associated with GGAA repeat sites, where loop number (median value = 11) and intensity (median value = 184) were significantly higher compared to both single GGAA (4 and 38, respectively) and non–EWS-FLI1 anchors (2 and 21, respectively) (Fig. 1D) and was not observed for loops associated with the related ETS family factors E74 Like ETS Transcription Factor 1 (ELF1) and GABPA (fig. S1E). These differences held true when loop intensity was assessed after segregating these genomic regions based on their corresponding H3K27ac signal. For comparable levels of H3K27ac signals, loops associated with EWS-FLI1 repeats still displayed significantly higher counts as compared to non–EWS-FLI1 anchors (fig. S1F). A similar pattern was also observed when categorizing loop length according to EWS-FLI1 occupancy and underlying DNA binding motifs. This analysis revealed a progressive increase in loop length associated with the presence of the fusion protein and the underlying binding sequence, with GGAA repeat-to-repeat loops showing the longest interaction distance that averaged 1.1 Mb, as compared to 52 to 79 kb for non–EWS-FLI1–associated loops, including chromatin loops associated with other ETS TFs such as ELF1 and GABPA (Fig. 1E and fig. S1G). Given this notable pattern, we sought to determine whether the observed differences in loop length may be linked to the distribution of GGAA repeats across the human genome. To this end, we computed the expected loop length by shuffling one of the anchors and found the values to be similar for loops associated or not with GGAA repeat anchors (fig. S1H). When we surveyed DNA sequences underlying enhancers connected to EWS-FLI1 binding sites, GGAA repeats scored as the top motif (fig. S1I), further pointing to the marked interconnectivity between regulatory elements bound by the fusion protein.

In aggregate, these observations identify a distinctive set of “enhancer hubs” associated with EWS-FLI1 binding sites that display high connectivity, intensity, and looping length compared to all other chromatin connections. These EWS-FLI1 enhancer hubs predominantly engage e-e and e-p connections and cooperate with both EWS-FLI1 and non–EWS-FLI1–centered loops in a broad collaborative network (Fig. 1F).

### EWS-FLI1 enhancer hubs depend on EWS-FLI1 expression and regulate EwS-specific transcriptional programs through complex looping networks

**Fig. 2.**
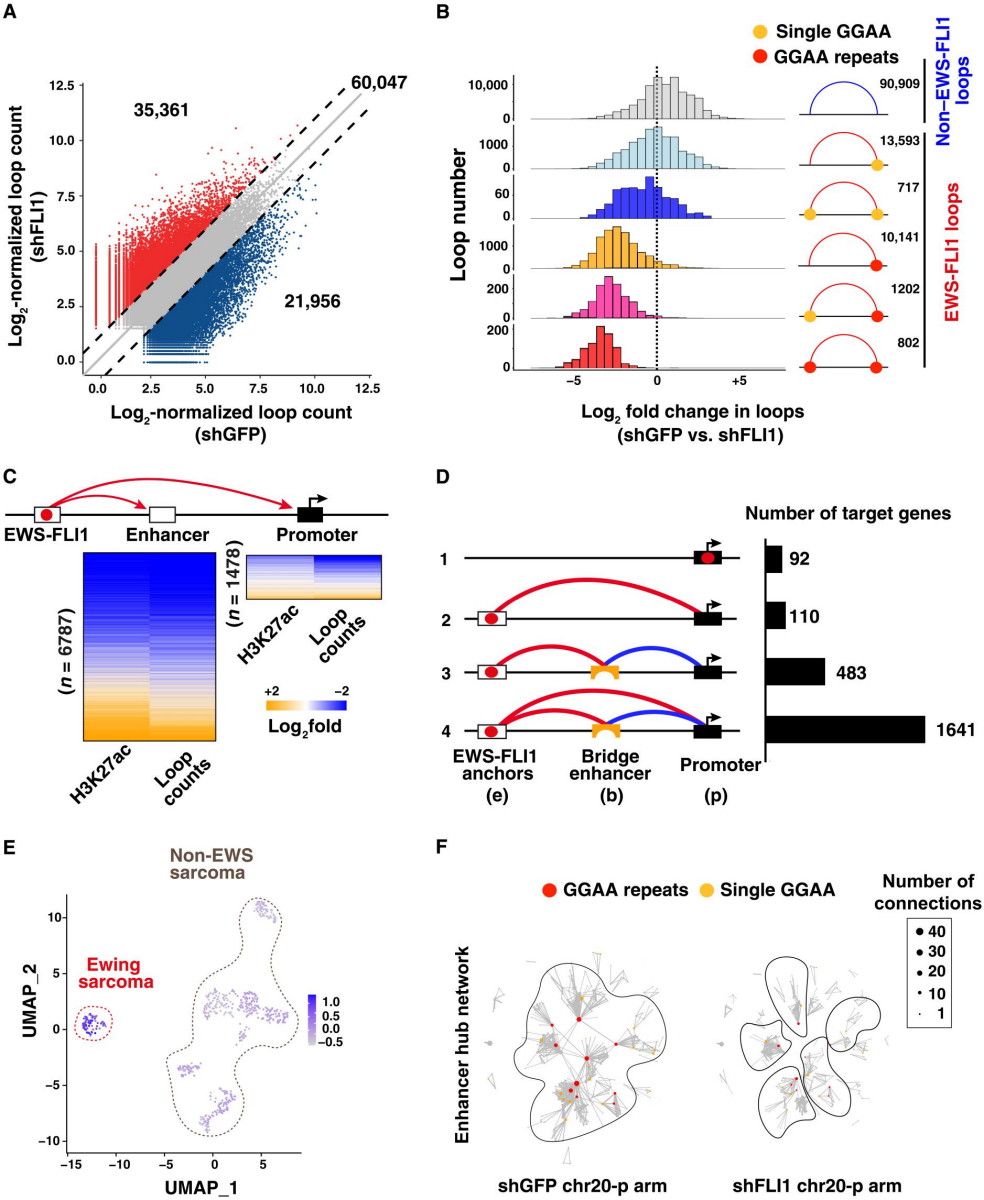
EWS-FLI1 depletion leads to disruption of 3D connectivity and decreased target expression. (**A**) Scatterplot showing changes in loop counts between shGFP- and shFLI1-infected A673 cells. Log-transformed normalized loop counts are represented. (**B**) Distribution of interaction changes following EWS-FLI1 depletion in A673 cells, across the different categories represented in Fig. 1E. Loop numbers are plotted against log_2_ fold changes in normalized loop counts between shGFP and shFLI1 A673 cells. (**C**) Heatmaps showing changes in loop counts for EWS-FLI1–associated connections between shGFP- and shFLI1-infected A673 cells and corresponding changes in H3K27ac signal at target enhancers and promoters. (**D**) Different modalities for EWS-FLI1 target gene regulation in A673 cells, including direct and indirect connections via bridge elements. The number of target genes regulated through the different modes is shown in the right part of the figure. (**E**) UMAP depicts the expression score for EWS-FLI1 direct target genes across 11 different sarcoma subtypes included in the Treehouse gene expression dataset. (**F**) Organization of EWS-FLI1–connected enhancer hubs into larger network regulatory modules in A673 shGFP cells (left), which undergo fractionation following EWS-FLI1 depletion (right). The image shows chr20 p-arm. The network vertices indicate the anchors, and the edges are HiChIP loop connections between anchors.

To evaluate how EWS-FLI1 regulates cis-chromatin interactions across the genome, we depleted the fusion protein in A673 and SKNMC cells using short hairpin RNA (shRNA) and profiled differential looping patterns by H3K27ac HiChIP at 96 hours after infection (fig. S2A). Two HiChIP replicates were performed for shGFP and shFLI1 A673 cells, which showed high correlation scores (fig. S2B), whereas one replicate was generated for SKNMC cells. In A673 tumor cells, EWS-FLI1 knockdown resulted in a global reorganization of the genomic interactions, including increases (35,361) and decreases (21,956) in H3K27ac-mediated contact frequency (Fig. 2A). Similar changes (30,610 increased and 26,877 decreased loops) were also observed in SKNMC cells (fig. S2C). Decreases in looping upon EWS-FLI1 depletion were mostly observed at EWS-FLI1 enhancer hubs with GGAA repeats at anchor sites (either repeat-repeat or repeat-other), whereas single GGAA sites showed a mixed pattern, displaying both loop gains and losses (Fig. 2B and fig. S2D). In contrast, the vast majority of increases in chromatin interactions were associated with regions lacking EWS-FLI1 binding, reflecting the changes in lineage specification that follow the loss of the fusion protein (Fig. 2B and fig. S2D).

Loss of EWS-FLI1–mediated loops had strong effects on the chromatin states of target promoters and enhancers (Fig. 2C and fig. S2, E and F), indicating the importance of the identified interactions in determining the activity of the wide network of regulatory sites. The observed effect led us to investigate all the looping patterns that link EWS-FLI1 to its target gene promoters. We identified four main regulatory mechanisms (Fig. 2D and fig. S2G), including (i) direct binding of the fusion protein to gene transcription start site (TSS), (ii) direct promoter regulation through distal sites (e-p), (iii) presence of intermediate enhancer elements bridging EWS-FLI1 to its target TSS (e-b-p), or (iv) a mix of the two patterns. As expected, EWS-FLI1 depletion resulted in robust contact decreases for EWS-FLI1–associated e-b. Notably, this also reduced EWS-FLI1–independent b-p chromatin loops, confirming the central role of EWS-FLI1 enhancer hubs in maintaining the activation state of nodes in this regulatory network (fig. S2, H and I). All three connection mechanisms were associated with transcriptional regulation. Transcriptional and looping changes were highly concordant (fig. S3A), and the functional annotation of this target gene set revealed their enrichment for terms related to cell cycle and chromosome conformation regulation (table S2 and fig. S3B). This target gene repertoire was found to be highly expressed in primary EwS tumors compared to 11 other sarcoma types, confirming the tumor-specific nature of these regulatory processes and the biological relevance of our findings (Fig. 2E and fig. S3, C and D).

Given that EWS-FLI1 enhancer hubs are characterized by high connectivity and increased interaction length, we generated the global interaction map of A673 and SKNMC cells genome wide. We observed that EWS-FLI1 enhancer hubs are organized in large regulatory modules bridging together a high number of genomic sites into single interconnected 3D structures that cover large genomic regions. Following EWS-FLI1 loss, these hubs become partitioned into multiple smaller and narrower regulatory networks (Fig. 2F and fig. S3, E and F). EWS-FLI1 thus controls an oncogenic gene expression program that is highly specific for Ewing sarcoma through a complex network of promoters and enhancers centered on EWS-FLI1 binding sites. On a larger genomic scale, these enhancer hubs are organized into 3D interaction structures bridging multiple regions across the genome and converting them into broad interconnected modules.

### EWS-FLI1 depletion results in reorganization of A/B compartment strength and connectivity

Our results point to the pivotal role of EWS-FLI1 enhancer hubs in regulating a large set of e-p connections and corresponding target genes in EwS tumors. Given the substantial number of altered loops following EWS-FLI1 depletion, we next considered whether these changes are paired with alterations in high-order nuclear architecture. For this purpose, we complemented our high-resolution looping interaction maps in A673 and SKNMC cells with large-scale genome partitioning profiles for TADs and compartments generated by HiC (*7*). To this end, two HiC replicate maps for A673 and one map for SKNMC cells were generated. In addition, as an orthogonal approach to HiC, shGFP- and shFLI1-infected A673 cells were also profiled by MicroC to further substantiate our findings.

We first determined TAD boundaries and compared the results obtained for control cells and cells depleted of EWS-FLI1. We found similar insulation scores (correlation = 0.91) at most sites (fig. S4A), indicating that there are no widespread changes in TAD configuration. This is consistent with previous studies showing that TADs tend to remain stable despite major variations in transcriptional programs (*43*–*45*). We next calculated A/B compartments and compared compartment assignment scores across the genome. This analysis showed that compartment scores for EWS-FLI1–expressing and EWS-FLI1–depleted cells do not undergo strong changes. A/B assignment switches were observed in a subset of sites. These changes mostly affected low scoring sites and occurred at a similar frequency in loci with (9.7% of A-B switch) or without EWS-FLI1 binding (8.6% of A-B switch; fig. S4B and C). These observations suggest that compartment changes are the result of both direct and indirect EWS-FLI1 effects.

**Fig. 3.**
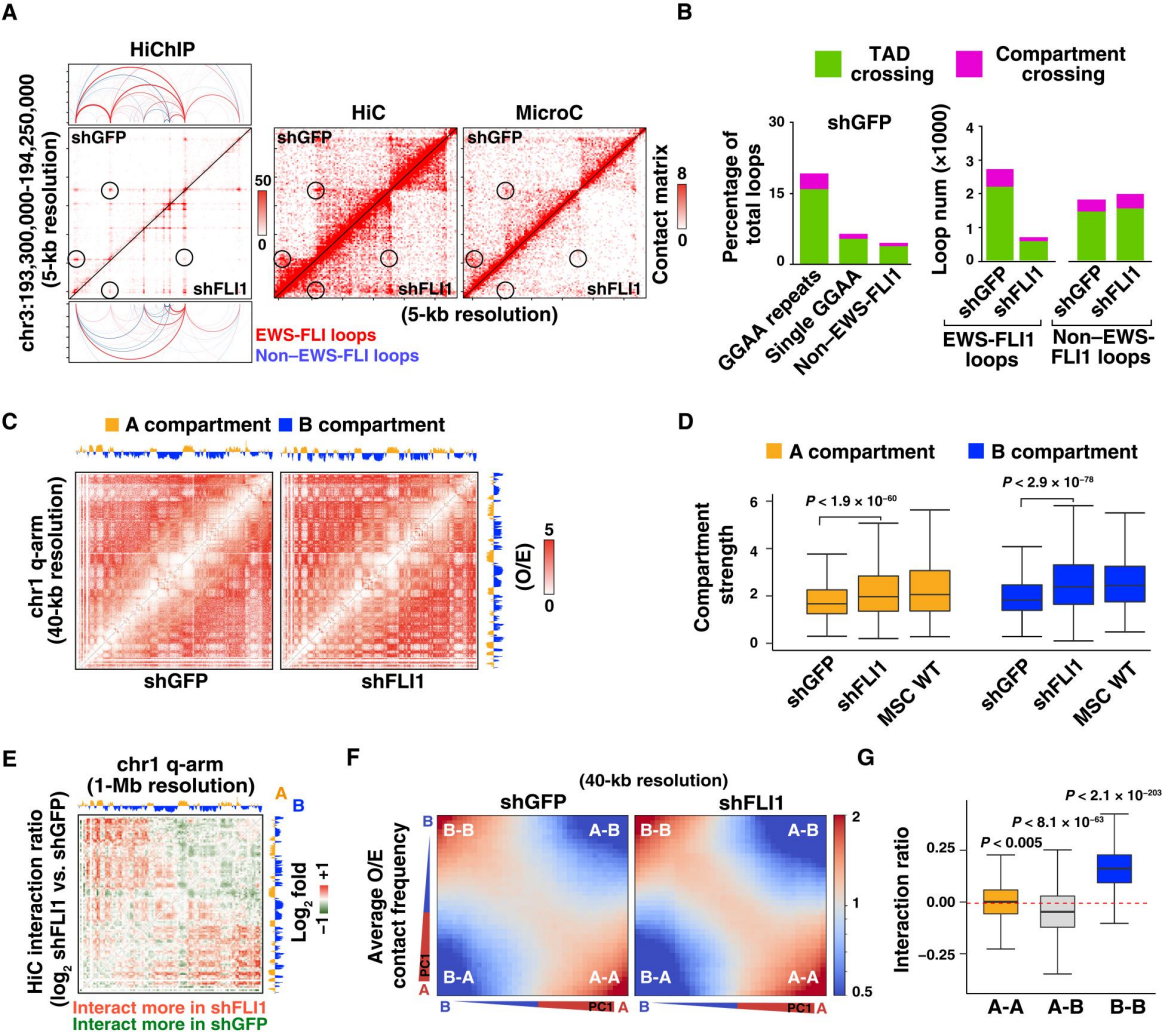
EWS-FLI1 induces changes in long-range connectivity and compartmental segregation. (**A**) Interaction matrix comparison between HiChIP (left), HiC (middle), and MicroC (right), showing similar changes in chromatin connectivity upon EWS-FLI1 depletion in A673 cells (circled). (**B**) Left: Bar plots depict the percentage of loops that cross TAD or compartments (as depicted in fig. S5A), classified according to the underlying DNA motif in shGFP-infected A673 cells. Right: Bar plots depicting the changes in loop number between shGFP and shFLI1 A673 cells for the same categories as in fig. S5A and indicating that decreases in TAD crossing and compartment bridging in A673 shFLI1 cells are mostly limited to fusion protein–associated loops. (**C**) HiC contact maps (O/E) across human chr.1 q-arm for shGFP- and shFLI1-infected A673 cells, showing the increase in compartmental segregation following the loss of EWS-FLI1 expression. Eigenvector-based A/B compartmental organization is represented on top of the interaction matrix. (**D**) Box plot showing strength for A and B compartments in A673 (shGFP and shFLI1) and MSC cells. The significance in comparison among samples was calculated by unpaired two-sided *t* test. (**E**) Heatmap showing changes in HiC contact frequencies between shGFP and shFLI1 A673 cells across chr.1 q-arm. (**F**) Saddle plots illustrating the changes in A-B compartmentalization strength between shGFP- and shFLI1-infected A673 cells. B-B interactions are shown in the top left corner, whereas A-A interactions are in the bottom right corner of the plots. (**G**) Box plots showing changes in compartment interactions between A673 shGFP and shFLI1 cells for A-A, A-B, and B-B HiC contacts. The significance in each category was calculated by Wilcoxon signed-rank test.

We next analyzed our HiChIP looping data in the context of large-scale HiC partitioning. As expected, changes in 3D interaction frequencies measured by HiC/MicroC matched changes in EWS-FLI1–mediated looping alterations observed by HiChIP in EWS-FLI1 depletion experiments, providing orthogonal confirmation for our HiChIP profiles (Fig. 3A and fig. S4, D and E). As most interactions are restricted within individual TADs, enhancers and other regulatory elements preferably regulate their target gene repertoire within the same TAD, with TAD boundaries functioning as a physical barrier for gene transcription (*12*, *14*). However, because EWS-FLI1–associated chromatin loops in EwS can span megabase-sized genomic regions, these connections may potentially link regulatory elements originating from different TADs or compartments. To this end, we computed the distribution of EWS-FLI1–anchored loops within all chromatin domains across the genome and determined whether EWS-FLI1 enhancer hub–associated loops may extend beyond single insulated neighborhoods and compartments (Fig. 3B and fig. S5A and B). This analysis revealed that inter-TAD and intercompartment chromatin contacts occur at higher frequency in association with EWS-FLI1 enhancer hubs, with loops originating from GGAA repeats showing the highest incidence of inter-TADs and intercompartment connections (Fig. 3B and fig. S5B). EWS-FLI1–centered inter-TAD and intercompartment cis-connectivity was strongly reduced in the absence of the fusion protein, whereas most other connections remained unaltered (Fig. 3B and fig. S5C).

We next considered whether knockdown of EWS-FLI1 results in global changes in A/B compartment interactions. Notably, EWS-FLI1 knockdown led to increased genome-wide compartment segregation, which is readily visible as a more distinct checkerboard pattern in HiC matrices for O/E (observed-over-expected) signals (Fig. 3C and fig. S5D). In keeping with this observation, calculation of compartment strength, a genome-wide measurement of relative intracompartment and intercompartment signals, showed increases in both EWS-FLI1–depleted A673 and SKNMC cells (Fig. 3D and fig. S5, E and F). In addition, we also generated HiC profiles for primary MSCs, a model for the cell of origin of EwS (*46*, *47*). A/B compartment strength observed after EWS-FLI1 depletion in EwS cells resembled the values calculated for MSCs, suggesting the reestablishment of the compartmental segregation observed in these primary cells.

To further define the pattern of changes in A/B compartment connectivity, we calculated HiC matrices depicting genome-wide interaction ratios after EWS-FLI1 depletion. The most prominent changes observed in these matrices were increases in B-B connections (Fig. 3, E and F, and fig. S5G). This matched genome-wide quantitation, which showed strong increases in B-B connections as well as decreases in A-B interactions (Fig. 3G and fig. S5, H and I). Comparable changes in B-B connections were also observed in MicroC-based analyses, further validating our observations (fig. S5, J and K).

Given these large-scale changes in B-B compartments, we surveyed for transcriptional changes associated to the high-order chromatin alterations induced by EWS-FLI1, independently to its direct binding sites. To this end, we set out to calculate the average expression of genes located within B compartments, showing increased interactions in the knockdown condition and that are not direct EWS-FLI1 targets (*n* = 3351 in A673 and *n* = 3537 in SKNMC). Consistent with the observed increases in B-B connections following EWS-FLI1 removal, we identified a moderate decrease in expression for genes located within B compartments (fig. S5L). Together, these observations show that EWS-FLI1 alters compartmental segregation, strength, and connectivity beyond its direct binding sites, which shape gene expression programs in EwS cells.

### EWS-FLI1 depletion induces a de novo enhancer connectivity landscape driving MSC programs

**Fig. 4.**
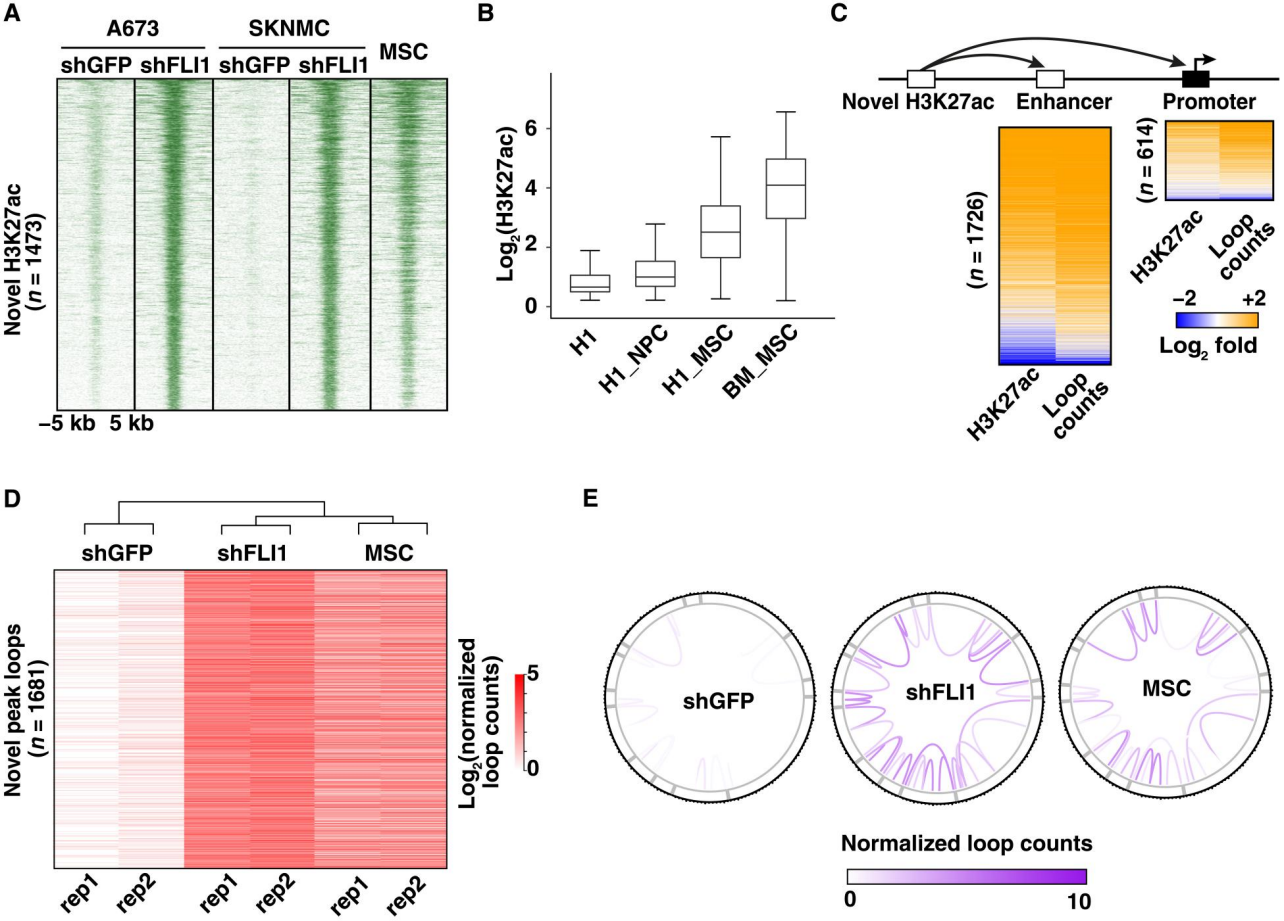
Chromatin loops induced de novo in EWS-FLI1–depleted cells recapitulate the chromatin connectivity of MSC precursors. (**A**) Heatmap showing ChIP-seq signals for de novo H3K27ac peaks emerging in A673 and SKNMC shFLI1 cells, as compared to their shGFP counterparts and illustrating their similarity to wild-type MSCs. (**B**) Relative H3K27ac peak signals for the 1473 de novo regulatory sites from (A), surveyed in H1 embryonic stem cells (H1) and their in vitro differentiated progeny (H1-NPC and H1-MSC), as well as bone marrow–derived MSCs (BM-MSC). H1-NPC, H1-derived NPCs. H1-MSC, H1-derived MSCs. (**C**) Heatmaps depicting coordinated changes in H3K27ac signal and normalized loop counts for enhancers and promoters targeted by the 1473 de novo peaks shown in (A) in shFLI1 A673 cells. (**D**) Heatmap showing normalized counts for chromatin loops associated with the 1473 de novo H3K27ac peaks in shGFP and shFLI1 A673 cells, as well as normal MSCs. (**E**) Circos plots for human chr20 illustrating that the 2444 de novo loops identified in shFLI1 A673 cells (D) display high similarity with the corresponding looping pattern of wild-type MSCs.

Given that EWS-FLI1 depletion is associated with both decreases and increases in chromatin looping and HiC contacts (Fig. 2A and fig. S2C), we next examined whether the new active connections identified in EWS-FLI1–depleted cells may be driven by regulatory elements that control transcriptional programs associated with candidate EwS cells of origin. To evaluate this hypothesis, H3K27ac ChIP-seq profiles for shGFP- and shFLI1-infected A673 and SKNMC cells were surveyed for “de novo” H3K27ac regions, which are absent in shGFP cells. This analysis yielded 1473 de novo distal sites shared between the two cellular models. These regions displayed a similar active chromatin state in primary MSCs (Fig. 4A). Moreover, H3K27ac annotation across a panel of H1 embryonic stem cell–derived cell types profiled by the Roadmap Epigenomics Mapping Consortium (*48*) revealed that these distal sites were selectively activated in H1- and bone marrow–derived MSCs but not in H1-derived neural progenitor cells (NPCs) and parental H1 embryonic stem cells (Fig. 4B). This de novo enhancer landscape was associated with the induction of corresponding chromatin loops and transcriptional programs. Accordingly, we observed increases in both H3K27ac signal at target enhancers and promoters of de novo distal regulatory sites and counts of connecting chromatin loops (Fig. 4C). Given the substantial number of newly generated enhancers, we next determined which sequence-specific TFs may preferentially engage these new regulatory elements and drive their long-range chromatin contacts. Motif enrichment analysis for the 1473 de novo distal elements identified consensus binding sequences for Basic Leucine Zipper (bZIP) TFs of the AP1 (activating protein 1)/ATF (activating transcription factor) family (fig. S6A), which are known to regulate cellular differentiation in different cancer types (*49*).

**Fig. 5.**
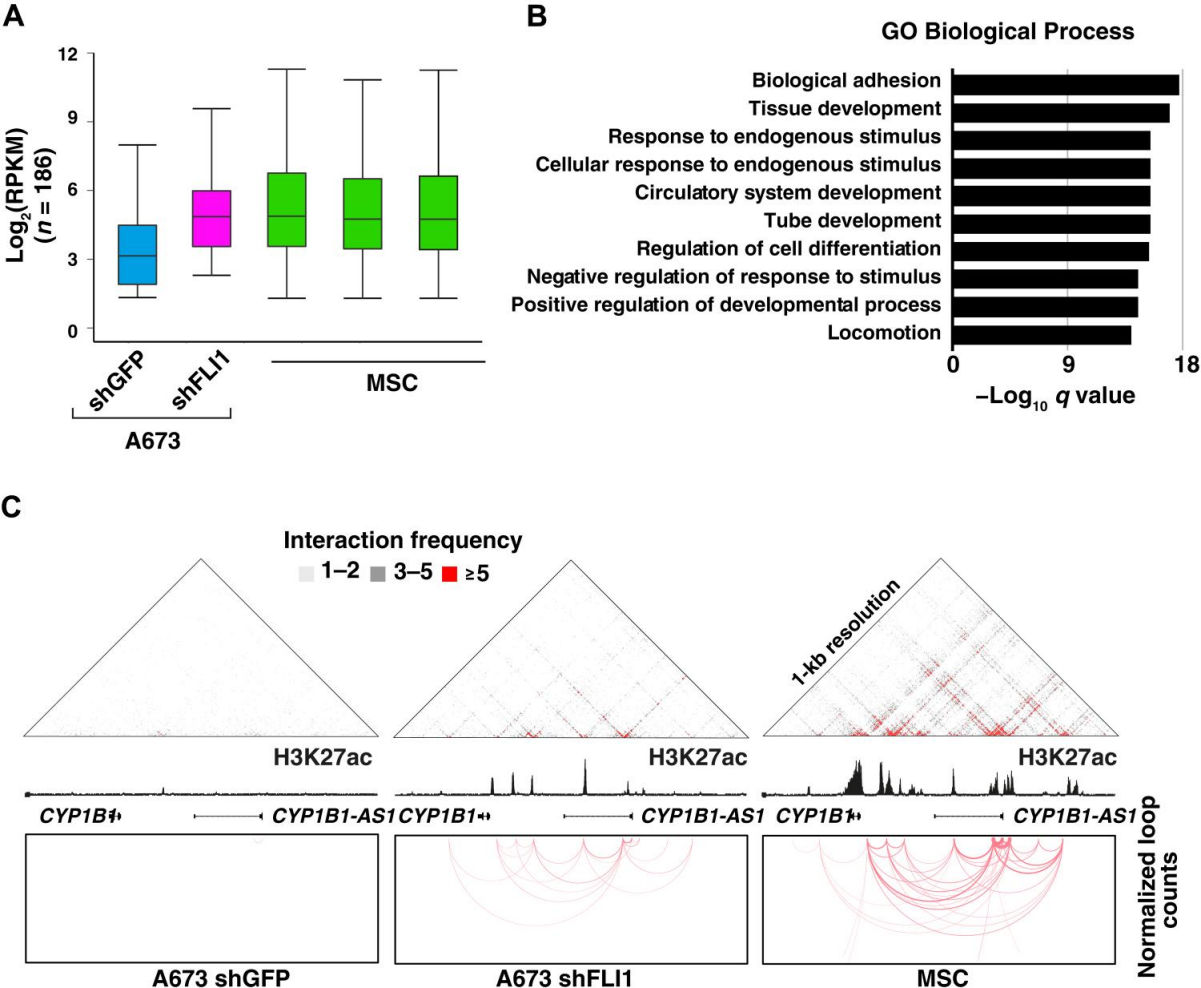
Genes targeted by de novo H3K27ac loops in EWS-FLI1–depleted cells are involved in mesenchymal differentiation pathways. (**A**) Box plots depicting the transcriptional levels of the 241 genes targeted by de novo loops described in Fig. 4D and that show increases in shFLI1 A673 cells matching the expression levels observed in MSCs. (**B**) Functional annotation of the 241 genes as in (A) for Biological Process (GO: BP) showing their involvement in mesenchymal differentiation pathways. (**C**) Schematic representation of the *CYP1B1* genomic locus, showing signals for raw HiChIP interaction frequencies (top), H3K27ac signal (middle), and chromatin looping (bottom) in shGFP and shFLI1 A673 cells, as well as wild-type MSCs, which illustrate that the emergence of loops associated with de novo H3K27ac sites in A673 shFLI1 cells recapitulates the 3D interaction pattern observed in MSCs.

In view of the marked similarity between the enhancer landscapes of EWS-FLI1–depleted EwS cells and primary MSCs, we determined whether the corresponding genome-wide 3D active contacts might also be comparable. We therefore identified all newly generated active chromatin interactions in EWS-FLI1–depleted tumor cells and compared them to corresponding HiChIP profiles in MSCs. Differential loops were selected on the basis of fourfold loop count increase (1681 new loops identified) and used to generate a heatmap comparing their relative strength between shGFP and shFLI1 A673 cells, as well as wild-type MSCs. A robust subset of the interactions enriched in shFLI1 A673 cells were also present in wild-type MSCs (45.6%; 768 loops with normalized counts ≥5; Fig. 4, D and E, and fig. S6, B and C), suggesting that the differentiation program activated upon EWS-FLI1 removal relies, at least in part, on a chromatin architecture shared with primary MSCs. Last, H3K27ac HiChIP profiles of EWS-FLI1–depleted EwS cells were used to identify direct target promoters of these novel chromatin loops and survey the expression level of their corresponding transcripts. The average expression level of this gene set in shFLI1-infected cells was increased to a level similar to the one observed in multiple wild-type MSC cultures (Fig. 5A). In addition, functional gene set enrichment analysis [Gene Ontology (GO)] for these genes revealed their significant enrichment for terms related to cellular migration, locomotion, and adhesion, consistent with the acquisition of a mesenchymal phenotype by shFLI1 tumor cells (Fig. 5B). As an example, *CYP1B1*, a representative mesenchymal target locus, showed acquisition of de novo H3K27ac peaks and chromatin loops in EWS-FLI1–depleted cells, which mirrored both the enhancer and 3D landscape of primary MSCs (Fig. 5C). Together, these observations suggest that the loss of the fusion protein initiates structural remodeling of the tumor cell cis-connectivity, which is driven by a group of de novo regulatory elements and chromatin connections, and restores latent differentiation programs inherent to the tumor precursor cells.

## DISCUSSION

Most of our current knowledge about variations in genome topology is derived from studies focusing on 3D conformational changes during normal development and from comparisons between static states in physiological and pathological conditions (*22*, *50*, *51*). As a consequence, the role of TFs in changing 3D topology and the degree to which major features of nuclear organization are plastic and reversible remain largely unexplored. The present study uses EWS-FLI1 as a model to tackle these questions, and we find that a single TF can reconfigure both looping patterns and high-order 3D interactions that shape the nuclear architecture of tumor cells. In particular, we identified a set of EWS-FLI1–dependent enhancer hubs that show high connectivity, form a looping network that covers large genomic regions, and coordinate the oncogenic and differentiation programs that define EwS identity. Conversely, EWS-FLI1 loss causes a partial disassembly of these hubs into smaller nodes, leading to the emergence of a different interaction landscape that includes de novo regulatory elements that control transcriptional programs related to mesenchymal differentiation. Although single-cell looping technologies would be required to formally prove the presence of these enhancer hubs in individual tumor cells, our previous study targeting selected single distal GGAA repeats by CRISPRi (*40*) shows an almost complete loss of target gene expression, suggesting that most tumor cells share the same enhancer landscape and 3D organization. From a more general point of view, these findings argue that nuclear organization in cancer cells is supported by selected TFs endowed with topological remodeling properties but remains highly plastic.

Regulatory elements similar to the EWS-FLI1 enhancer hubs that we have identified in EwS may be relevant to other biological systems. For example, similar hubs of multiconnected enhancers that function as co-regulatory networks for transcriptional programs implicated in cell identity have been described during induced pluripotent stem cells and B cell reprogramming, as well as in tumor cells (*32*, *52*, *53*). However, the driving forces underlying the assembly and coordination of these chromatin structures have until now remained elusive. Here, we identify EWS-FLI1 as one such force, which, by relocating multiple genomic loci in different chromatin contexts and modulating their transcriptional activity, becomes a potent oncogenic driver. Our results thus suggest that analysis of similar enhancer hubs in other cellular models may uncover related TFs that determine lineage identity. By analogy to super-enhancers, gaining deeper insight into the organizational principles of 3D enhancer hubs may allow modulation of their assembly and the consequent control of cell fate changes during normal development and cancer evolution. Given the increasingly recognized role of differentiation therapies in enhancing tumor cell sensitivity toward standard treatments and novel immunotherapies, the possibility to regulate malignant cell states through disruption of selected enhancer hubs may offer additional options for clinical management (*54*–*57*).

A major finding of our study is the extent of local and global topological changes induced by EWS-FLI1 depletion despite the limited number of its binding sites. Additional experiments will be required to define further details of EWS-FLI1–dependent and EWS-FLI1–independent mechanisms of nuclear reorganization. These include whether the fusion protein has a structural role independent of enhancer activation and whether the nuclear structure reorganization that follows EWS-FLI1 depletion also reflects interactions or competition with other TFs. Three major mechanisms are likely to be implicated. First, recent studies show that active loop extrusion can weaken compartmentalization (*58*–*61*), and thus, the establishment of a large network of loops by EWS-FLI1 may, by itself, antagonize compartment formation. This may be particularly relevant for EWS-FLI1–centered loops, which display a high tendency to cross TADs and bridge genomic regions of different compartmental identity, preventing normal interactions. Given the pattern of changes observed after EWS-FLI1 depletion, B-B interactions, which normally ensure proper genomic segregation (*62*), may be most affected by this mechanism. Second, TFs that contain intrinsically disordered regions and generate biomolecular condensates have been identified as drivers of 3D chromatin reorganization (*63*). Thus, in addition to enabling pioneer activity at GGAA repeats, the prion-like domains of EWS-FLI1 may play an important role in modeling the nuclear architecture of EwS through condensate formation (*39*). This process may also entail the subsequent recruitment of additional TFs and chromatin regulators but remains dependent on the presence of EWS-FLI1.

Third, alterations in compartment strength that follows EWS-FLI1 depletion may be connected to reactivated differentiation programs (*64*). In particular, the changes observed upon EWS-FLI1 depletion are reminiscent of embryonic stem cell differentiation, which has been linked to loss of a permissive chromatin configuration through stronger B-to-B interactions and increased compartmental segregation (*65*). The compartment mixing provided by the enhancer hubs regulated by EWS-FLI1 in EwS may thus generate a plastic epigenetic environment that resembles cellular reprogramming and promotes tumor development (*27*, *63*).

In summary, our findings provide a proof of concept for the instructive role of an epigenetic regulator in shaping all layers of the 3D genome within tumor cells. They also suggest that changes in chromatin looping and segregation induced by EWS-FLI1 are highly dynamic and may override the original nuclear architecture of EwS precursor cells to favor cellular transformation. Considering that similar mechanisms are likely to be involved in driving additional diseases either through the action of fusion proteins or other gene regulation abnormalities, our findings provide a roadmap for investigating 3D regulatory hubs in diverse physiological and pathological contexts.

## MATERIALS AND METHODS

### Cell lines and MSC culture conditions

The EwS cell lines A673 and SKNMC (American Type Culture Collection) were grown in RPMI containing 10% fetal bovine serum (FBS) at 37°C with 5% CO_2_. MSCs were collected as anonymized discarded materials with approval from the Institutional Review Boards of Centre Hospitalier Universitaire Vaudois (University of Lausanne, Switzerland). Primary pediatric MSCs were cultured in Iscove’s modified Dulbecco’s medium containing 10% FBS and platelet-derived growth factor BB (10 ng/ml; PeproTech), as described previously (*66*).

Lentivirus-mediated knockdown experiments, RNA isolation, and reverse transcription polymerase chain reaction (PCR) analysis were carried out as previously described (*36*, *66*). The lentiviral shRNAs were obtained from the RNAi Consortium for pLKO.1 shFLI1 (TRCN0000005322, target sequence CGTCATGTTCTGGTTTGAGAT) and pLKO.1 shGFP (target sequence GCAAGCTGACCCTGAAGTTCAT).

### Western blotting

The antibodies used for Western blot analysis were anti-FLI1 (ab15289, Abcam), anti–glyceraldehyde-3-phosphate dehydrogenase (MAB374 Millipore), and horseradish peroxidase–conjugated sheep anti-mouse (Cytiva) or goat anti-rabbit secondary antibodies (Dako). Whole-cell protein lysates were prepared by standard methods and quantified using the Bradford reagent (Bio-Rad). Protein electrophoresis was performed in precast Bolt 4 to 12% bis-tris gels in MES running buffer and Mini Gel Tank according to the manufacturer’s protocol (Invitrogen). Blotting was performed by the standard procedure, membranes were developed using the enhanced chemiluminescence substrates SuperSignal West Pico PLUS (Thermo Fisher Scientific) or WesternBright Sirius (Advansta), and proteins were visualized with a FUSION FX camera (Vilbert-Lourmat).

### HiC analysis

HiC was performed according to Rao *et al.* (*7*) with few modifications. Briefly, 1 to 3 M formaldehyde cross-linked cells were lysed in HiC lysis buffer [10 mM tris-HCl (pH 8.0), 10 mM NaCl, and 0.2% IGEPAL] for 30 min at 4°C. Nuclei were washed using HiC lysis buffer, resuspended in 0.5% SDS (100 μl), and incubated for 10 min at 62°C. The SDS was quenched using 50 μl of 10% Triton X-100 and 265 μl of water for 15 min at 37°C. Furthermore, the chromatin was digested using 200 U each of Mbo I and Hinf I enzymes (1× NEB Buffer2.1) for 2 hours at 37°C. Restriction enzymes were heat-inactivated at 65°C for 20 min and washed off. The digested chromatin was end filled using 37.5 μl of 0.4 mM biotin-14-dATP (2′-deoxyadenosine 5′-triphosphate). The reaction was carried out in 1× NEB buffer with 50 U of Klenow polymerase I and a combination of 2′-deoxycytidine 5′-triphosphate (dCTP), 2′-deoxyguanosine 5′-triphosphate (dGTP), and 3′-deoxythymidine 5′-triphosphate (dTTP; 1.5 μl of 10 mM each) for 1.5 hours at 37°C. End-filling proceeded with proximity ligation [1× ligation buffer, 4000 U of T4 DNA Ligase, 125 μl of 10% Triton X-100, and 7.5 μl of bovine serum albumin (BSA) in 1.5-ml reaction] for 4 hours at 25°C.

### MicroC analysis

The MicroC library was prepared using the Dovetail MicroC Kit according to the manufacturer’s protocol. Briefly, chromatin was fixed with disuccinimidyl glutarate and formaldehyde in the nucleus. The cross-linked chromatin was then digested in situ with micrococcal nuclease. Following digestion, the cells were lysed with SDS to extract the chromatin fragments that were subsequently bound to Chromatin Capture Beads. Next, chromatin fragments were end-repaired and ligated to a biotinylated bridge adapter followed by proximity ligation of adapter-containing ends. After proximity ligation, the cross-links were reversed, the associated proteins were degraded, and the DNA was purified and converted into a sequencing library using Illumina-compatible adaptors. Biotin-containing fragments were isolated using streptavidin beads before PCR amplification. The library was sequenced on an Illumina NextSeq 500 platform to generate more than 500 million 2 × 42–base pair (bp) read pairs.

### HiChIP analysis

HiChIP was performed according to Mumbach *et al.* (*42*) with few modifications. Briefly, 5 M formaldehyde cross-linked cells were lysed in HiC lysis buffer [10 mM tris-HCl (pH 8.0), 10 mM NaCl, and 0.2% IGEPAL] for 30 min at 4°C. Nuclei were washed using HiC lysis buffer, resuspended in 0.5% SDS (100 μl), and incubated for 10 min at 62°C. The SDS was quenched using 50 μl of 10% Triton X-100 and 265 μl of water for 15 min at 37°C. Furthermore, the chromatin was digested using 200 U of Mbo I enzyme (1× NEB Buffer2.1) for 2 hours at 37°C. The restriction enzyme was heat-inactivated at 65°C for 20 min and continued to end fill the digested chromatin using 37.5 μl of 0.4 mM biotin-14-dATP. The reaction was carried out in 1× NEB buffer with 50 U of Klenow polymerase I and a combination of dCTP, dGTP, and dTTP (1.5 μl of 10 mM each) for 1.5 hours at 37°C. End-filling proceeded with proximity ligation (1× ligation buffer, 4000 U of T4 DNA Ligase, 125 μl of 10% Triton X-100, and 7.5 μl of BSA in 1.5-ml reaction) for 4 hours at 25°C. After ligation, chromatin was collected by spinning, followed by ChIP. Chromatin was lysed in nuclei lysis buffer (10 mM tris, 1% SDS, 10 mm EDTA, and 1× protease inhibitor cocktail), diluted in ChIP dilution buffer (20 mM tris 7.4, 0.1% SDS, 0.12% DOC, 160 mM NaCl, 1.2% Triton X-100, and 1× PIC), and sonicated a total volume of 800 μl. H3K27ac antibody (5 μg; Active Motif, #39133) was used for immunoprecipitation overnight, followed by ChIP washes and reverse cross-linking as previously described (*67*). ChIP DNA was purified using a Zymo ChIP DNA concentrator followed by biotin pull-down. Two microliters of Dynabeads MyOne Streptavidin T1 beads (10 mg/ml) was washed in 1× binding buffer (5 mM tris-HCl, 0.5 mM EDTA, and 1 M NaCl) for 15 min (50-μl reaction), followed by washes using 1× Tween Washing Buffer (5 mM tris-HCl, 0.5 mM EDTA, 1 M NaCl, and 0.05% Tween 20) and magnetic separation. Bead-bound DNA was used for library preparation using the Active Motif Next Gen DNA Library Kit (#53216) according to the instructions. Instead of free DNA, every reaction was carried out on the beads and followed by magnetic separation. Last, the HiChIP DNA library was PCR-amplified for 8 cycles and purified using SPRI beads. All HiChIP DNA libraries were sequenced at 100-bp paired-end to 300 million read-pairs/sample.

### ChIP-seq processing

The H1 NPC, H1 MSC, and bone marrow MSC ChIP-seq datasets were obtained from the Roadmap Epigenomics Mapping Consortium (https://egg2.wustl.edu/roadmap/web_portal/). EWS-FLI1 and H3K27ac ChIP-seq results for A673 and SKNMC cells were obtained from the Gene Expression Omnibus (GEO) (GSE61953) and were aligned against hg19 genome using bwa v.0.7.12 with default settings (*68*). The duplicate reads were removed by using picard-tools v.1.95; we then extended aligned reads to 200 bp to approximate fragment sizes. The density maps were normalized to 10 million reads by BEDTools v.2.17.0 (*69*). ChIP-seq peaks were identified with MACS2 v.2.2.7.1 (*70*). The narrow peak setting was used for TFs, while broad peaks were called for H3K27ac histone markers. We identified 3621 and 3093 EWS-FLI1 ChIP-seq peaks in A673 and SKNMC cells, respectively, and the total number of H3K27ac ChIP-seq peaks are 36,047 and 25,187 in A673 and SKNMC cells, respectively. Peaks within ±1 kb of TSS were defined as promoter sites, and the remaining sites were considered distal sites. Chromatin and TF peak signals were quantified using Python (pyBigWig) as the average coverage within 2-kb windows. To identify novel H3K27ac peaks after EWS-FLI1 knockdown in A673 and SKNMC cells, we calculated the union of H3K27ac peaks from A673 shGFP, A673 shFLI1, SKNMC shGFP, and SKNMC shFLI1. H3K27ac signals in the union peak set were calculated using pyBigWig v.0.3.17. The novel H3K27ac peaks were defined as peaks with fourfold signal increases in shFLI1 and an average signal of less than 5 relative units in shGFP.

### HiChIP data processing

For the analysis of H3K27ac HiChIP data, paired-end reads were aligned against the hg19 genome using the HiC-Pro pipeline (v 2.7.6) (*71*). Default settings were used to align paired reads, identify valid interactions, and generate interaction matrices. Then, the hichipper tool (v 0.7.7) (*72*) was used for loop calling within H3K27ac ChIP-seq peak (*q* < 0.00001) regions by applying the following parameters: --max-distance 100000000 --read-length 100. Loops with counts greater than 5 and identified in both replicates were kept for further analysis. To eliminate the H3K27ac difference–induced loop bias when comparing shGFP and shFLI1, we performed the hichipper analysis using the union H3K27ac peaks from shGFP and shFLI1. Loops were normalized by using the method described in diffloop (*73*). The EWS-FLI1 binding anchors with GGAA repeats motif were defined as EWS-FLI1 GGAA repeat anchors, and other anchors were defined as EWS-FLI1 GGAA nonrepeat anchors.

To identify EWS-FLI1–connected genes, we considered three possible connectivity patterns: (i) promoters (±1 kb of TSS) bound by EWS-FLI1, (ii) promoters connected to EWS-FLI1 binding sites by loops (with or without bridge anchors), and (iii) promoters connected to bridge anchors that have decreased looping upon EWS-FLI1 loss (twofold decrease in shFLI1). Bridge anchors are defined as EWS-FLI1–connected enhancers.

When comparing the loops between shGFP- and shFLI1-infected samples, we annotated the union set of loops into six categories based on the overlapping between loop anchors and the binding of EWS-FLI1 in shGFP sample. EWS-FLI1 connected genes that were down-regulated after EWS-FLI1 depletion with at least 1.5-fold decreases in A673 or SKNMC cells were selected as EWS-FLI1 target genes.

Novel H3K27ac loops were defined as H3K27ac loops with more than fourfold increase in shFLI1 and less than two normalized loop counts in shGFP. Genes targeted by novel HiChIP loops that increased more than 1.5-fold expression in shFLI1 were defined as novel loop-targeted genes. The HiChIP networks for A673 and SKNMC were constructed using the R package “Intergraph” v.2.0-2. The vertexes were loop anchors, and the edges were interactions between anchors. A673 and SKNMC HiChIP networks were visualized through R package ggnet v.1.0.

### HiC and MicroC data processing

Paired-end HiC and MicroC reads were aligned against the hg19 genome using HiC-Pro (*71*). Default settings were used to control quality, align paired reads, identify valid interactions, and generate interaction matrices. The iterative correction and eigenvector decomposition method was performed for each sample (*16*). For each chromosome in each sample, compartments were called as previously described (*24*) using the standard principal components analysis method (*15*). Briefly, for each sample, an O/E matrix was generated by dividing each interaction of the matrix by the expected interaction frequency (means) for a given distance from the diagonal. The correlation matrix was generated by performing the pairwise correlation coefficients of the O/E matrix. An eigendecomposition was performed on the correlation matrix, and the first eigenvector was used to assign compartment labels. H3K27ac signals for each 40-kb bin were used for selecting the sign of the eigenvector.

We calculated the compartment strength for each 40-kb bin based on the compartment A/B definition. The mean values of same compartment and different compartment interactions were calculated for each 40-kb-resolution bin, and the compartment strength was defined as the ratio of the mean value of same compartment interactions versus the value of different compartment interactions. We further calculated the compartment score changes, for a given 40-kb bin *b* in B compartment

$$\text{Repress ratio}=\frac{\sum_{1}^{m}[\log_{2}(I_{bm}\mathrm{shEF}+1)-\log_{2}(I_{bm}\mathrm{shGFP}+1)]}{m}-\frac{\sum_{1}^{n}[\log_{2}(I_{bn}\mathrm{shEF}+1)-\log_{2}(I_{bn}\mathrm{shGFP}+1)]}{n}$$

where *I* indicates the O/E interactions, *m* is the bin in B compartment, and *n* is the bin in A compartment.

TADs were defined for each chromosome on the basis of 40-kb bin matrix using the analysis method described by ENCODE (https://github.com/dekkerlab/cworld-dekker; version v0.0.1; access date, 16 September 2020). Briefly, we calculated the boundary strength for each chromosome from the O/E matrix by using matrix2insulation.pl with default settings. We next used insulation2tads.pl to define TAD boundaries from the information of boundary strength. To compare the HiC and MicroC interactions corresponding to HiChIP loops (EWS-FLI1 loops or loops associated with novel H3K27ac peaks), we used BEDTools pairtopair (v2.17.0) with the default settings. To evaluate the changes in compartment strength between shGFP and shFLI1 samples, we generated saddle plots using the compute-saddle utility of cooltools package (https://github.com/open2c/cooltools; version v0.5.2; access date, 28 April 2022).

### RNA sequencing processing and analysis

A673 and SKNMC RNA sequencing (RNA-seq) samples for EWS-FLI1 knockdown experiments were downloaded from the GEO series: GSE94278. The MSC RNA-seq datasets were obtained from GSE94278. Reads were aligned against hg19 using STAR v.2.4.0h (*74*). Aligned fragments were quantified using featureCounts (*75*), and FPKM expression values were calculated for hg19 RefSeq genes. Function enrichment (GO) analysis was performed using gene set enrichment analysis (www.gsea-msigdb.org/gsea/index.jsp).

### Treehouse sample processing

Treehouse public data were downloaded from https://treehousegenomics.soe.ucsc.edu/public-data/. We selected sarcoma samples (alveolar rhabdomyosarcoma, alveolar soft part sarcoma, dedifferentiated liposarcoma, embryonal rhabdomyosarcoma, endometrial stromal sarcoma, epithelioid sarcoma, EwS, infantile fibrosarcoma, leiomyosarcoma, myxofibrosarcoma, osteosarcoma, sclerosing epithelioid fibrosarcoma, and synovial sarcoma) for further analysis. Seurat (v.3.2.2) (*76*) was used for expression normalization. The scaled expression (*z* scores for each gene) was used for dimensional reduction. The top 25 principal components were used for visualization with Uniform Manifold Approximation and Projection (UMAP).

### Motif analysis

HOMER v.4.7 (*77*) was used for motif analysis. We used findMotifsGenome.pl (parameters: -size given -len 4,5,6,7,8,9,10,12,16) to identify motifs and used annotatePeaks.pl to annotate peaks with selected motifs.

## Notes

View/request a protocol for this paper from *Bio-protocol*.
