# Supplementary material for: Highly connected 3D chromatin networks established by an oncogenic fusion protein shape tumor cell identity
Source: Sci Adv. 2023 Mar 31;9(13):eabo3789. doi: 10.1126/sciadv.abo3789 (PMC10718307; doi:10.1126/sciadv.abo3789)
Supplement: Supplementary file 1 — Figs. S1 to S6 Tables S1 and S2 [file sciadv.abo3789_sm.pdf]

Science Advances
AAAS

Supplementary Materials for

Highly connected 3D chromatin networks established by an oncogenic fusion protein shape tumor cell identity

Rajendran Sanalkumar *et al.*

Corresponding author: Nicolò Riggi, nicolo.riggi@chuv.ch; Miguel N. Rivera, mnrivera@mgh.harvard.edu

Sci. Adv. **9**, eabo3789 (2023)
DOI: 10.1126/sciadv.abo3789

This PDF file includes:

Figs. S1 to S6
Tables S1 and S2

Fig. S1. The active enhancer network of EwS tumor cells is dominated by EWS-FLI1-associated loops

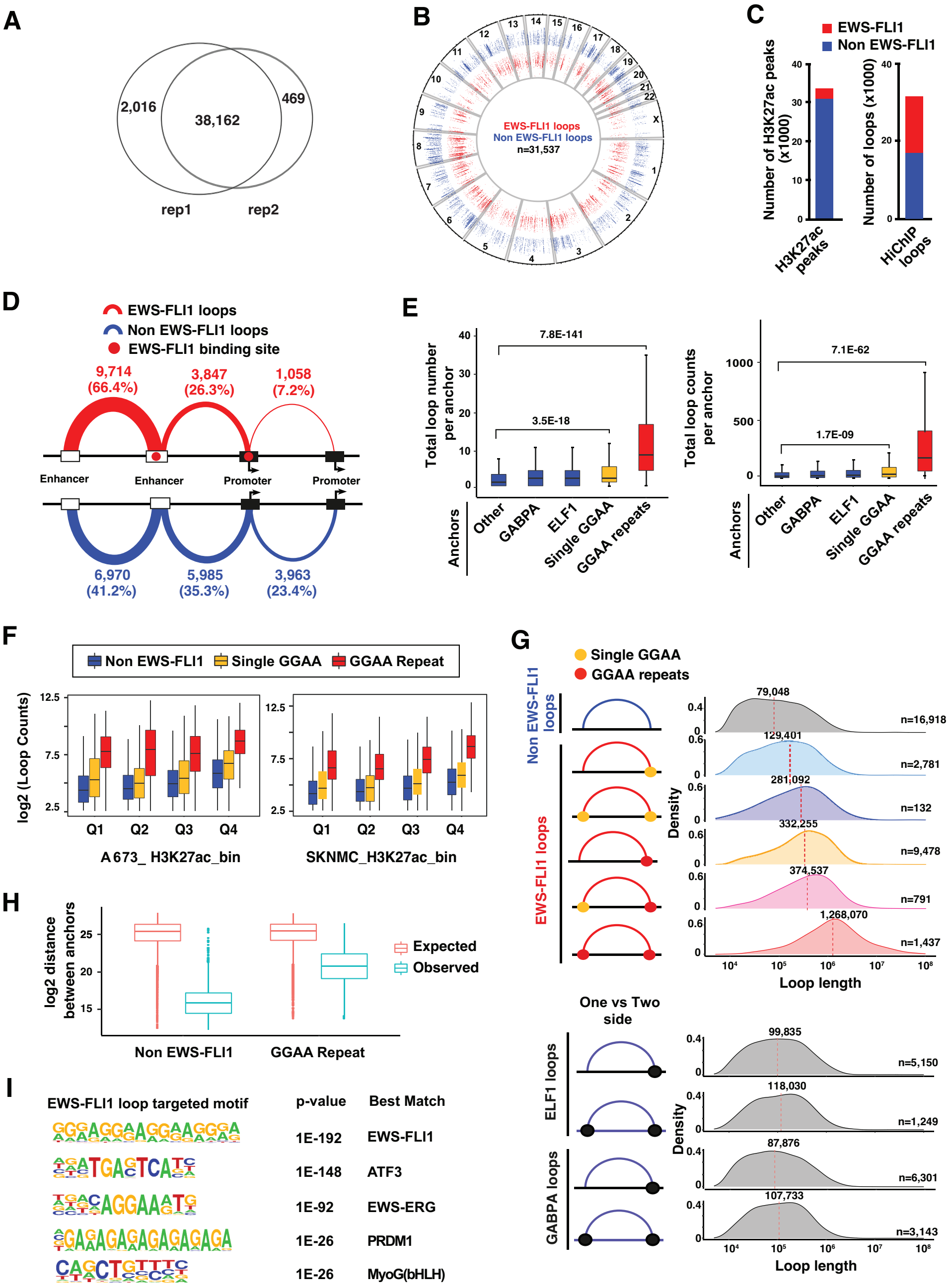

A, Venn diagram shows the overlap between two independent replicates of HiChIP loops from A673 cells. B, Circos plot depicts the genome-wide H3K27ac looping pattern in SKNMC cells.

EWS-FLI1-bound (red) or -independent loops (blue) are shown separately, and arranged according to their length. C, Number of H3K27ac ChIP-seq peaks (*left*) and loops (*right*) associated or not with EWS-FLI1 binding in SKNMC cells. D, Looping distribution for EWS-FLI1-associated or -independent loops in SKNMC cells. Total number and percentage of each category (enhancer-enhancer, enhancer-promoter or promoter-promoter) is shown for both EWS-FLI1 (red) and non EWS-FLI1 (blue) loops. E, Chromatin loops in SKNMC cells are categorized based on their association with EWS-FLI1, GABPa and ELF1 binding sites. EWS-FLI1-associated loops are further subdivided based on the underlying DNA motif, i.e. GGAA repeats or single GGAA. The total number of loops (*left*) and loop counts (*right*) per anchor are shown. The significance in comparison among anchors was calculated by unpaired two-sided t test. F, Box plots represent the loop counts in four quantiles based on H3K27ac signal intensity. H3K27ac ChIP-seq signals for HiChIP anchors were segregated into four quantiles based on their signal intensity, and the corresponding loops from A673 (*left*) and SKNMC (*right*) are categorized. G, *Top*: loop length distribution for both EWS-FLI1-associated and independent loops in SKNMC cells. EWS-FLI1 loops are further subdivided based on the presence of GGAA repeat or single GGAA motifs on either side of the loop anchors. *Bottom*: loop length distribution for chromatin interactions associated with the ETS transcription factors ELF1 and GABPA. Presence of the ETS factor in either side of the loop anchor is represented. Total number of loops in each category is shown in the right side of each plot. H, Observed loop lengths were plotted for GGAA repeat and non-EWS-FLI1 anchors and compared to their expected loop length after the anchors were randomly shuffled. I, Homer Motif analysis for HiChIP anchors targeted by loops associated with GGAA repeats in A673 cells. p-value was calculated and reported by HOMER. The presence of GGAA repeat motifs in this analysis suggest the high degree of inter-connectivity displayed by EWS-FLI1-associated networks.

Fig. S2. EWS-FLI1 depletion leads to disruption of 3D tumor connectivity

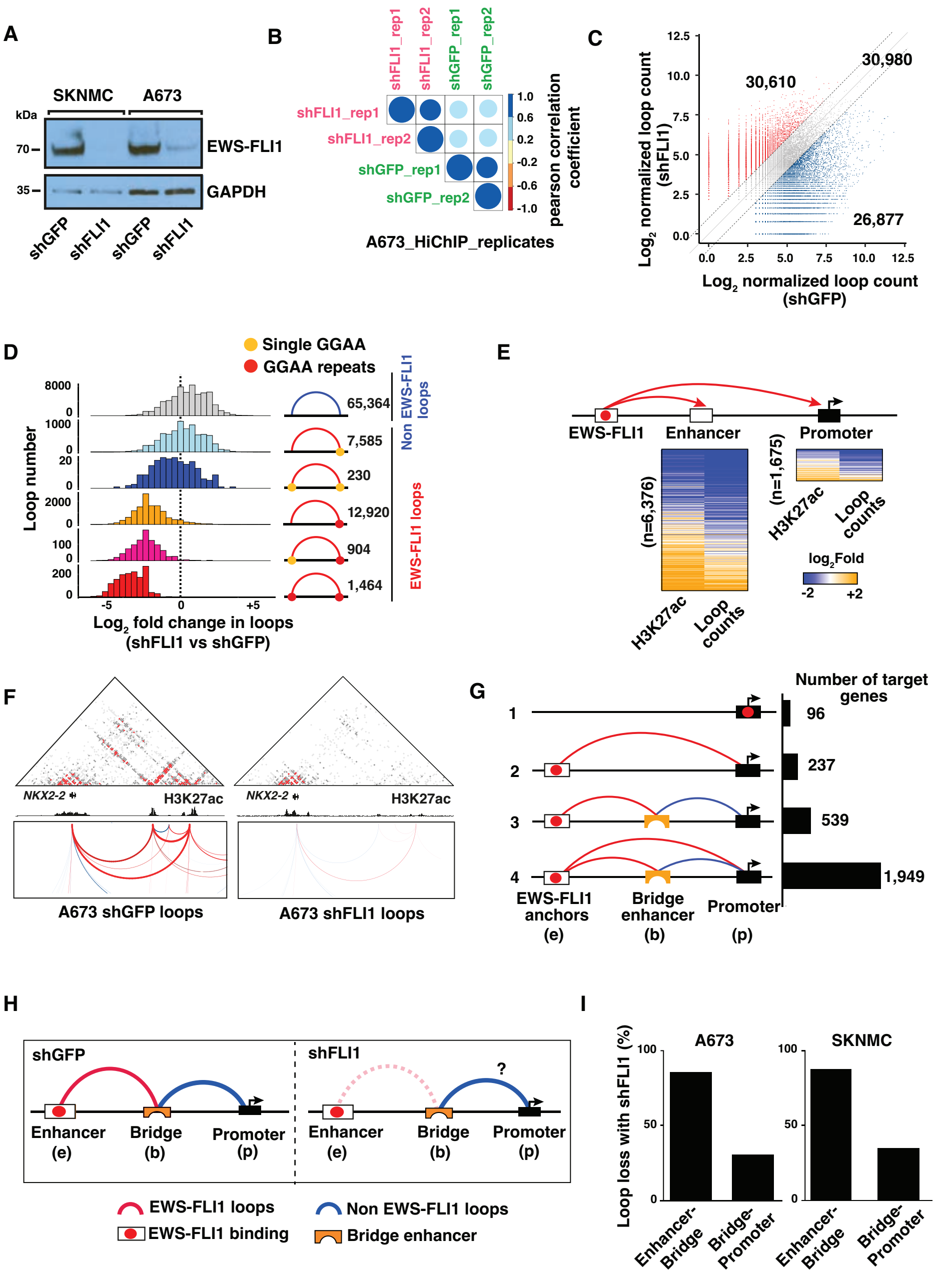

A, Western blot analysis confirming EWS-FLI1 protein knock-down in shFLI1-infected A673 and SKNMC cells after 96 hours. B, Pearson correlation for replicates of shGFP and shFLI1 HiChIP samples in A673 cells C, Scatter plot for differential chromatin interactions between shGFP- and shFLI1-infected SKNMC cells. Log2 transformed normalized loop counts are represented. D,

Magnitude and distribution of loop changes following EWS-FLI1 depletion in SKNMC cells, categorized by their association with the fusion protein binding sites. Loop numbers are plotted against Log2 fold change in normalized loop counts between shGFP and shFLI1 SKNMC cells. E, Changes in H3K27ac signal and loop counts at enhancer and promoters targeted by GGAA repeat-associated loops, following EWS-FLI1 knockdown in SKNMC cells. F, Image of the *NKX2-2* genomic locus in A673 cells, illustrating the major looping re-organization that follows EWS-FLI1 depletion. G, Different modalities for EWS-FLI1 target gene regulation in SKNMC cells, including direct and indirect connections via bridge elements. The number of target genes regulated through the different modes are shown in the right part of the figure. H, Image depicting the regulatory model for EWS-FLI1 targeted genes that includes a bridge element (left), and the possible changes in interactions that could follow EWS-FLI1 depletion (right). I, Bar plot depicting the percentage of e-b and b-p loops lost upon EWS-FLI1 depletion in A673 and SKNMC cells.

Fig. S3. EWS-FLI1-associated loops are organized in multi-connected 3D hubs regulating their corresponding target genes

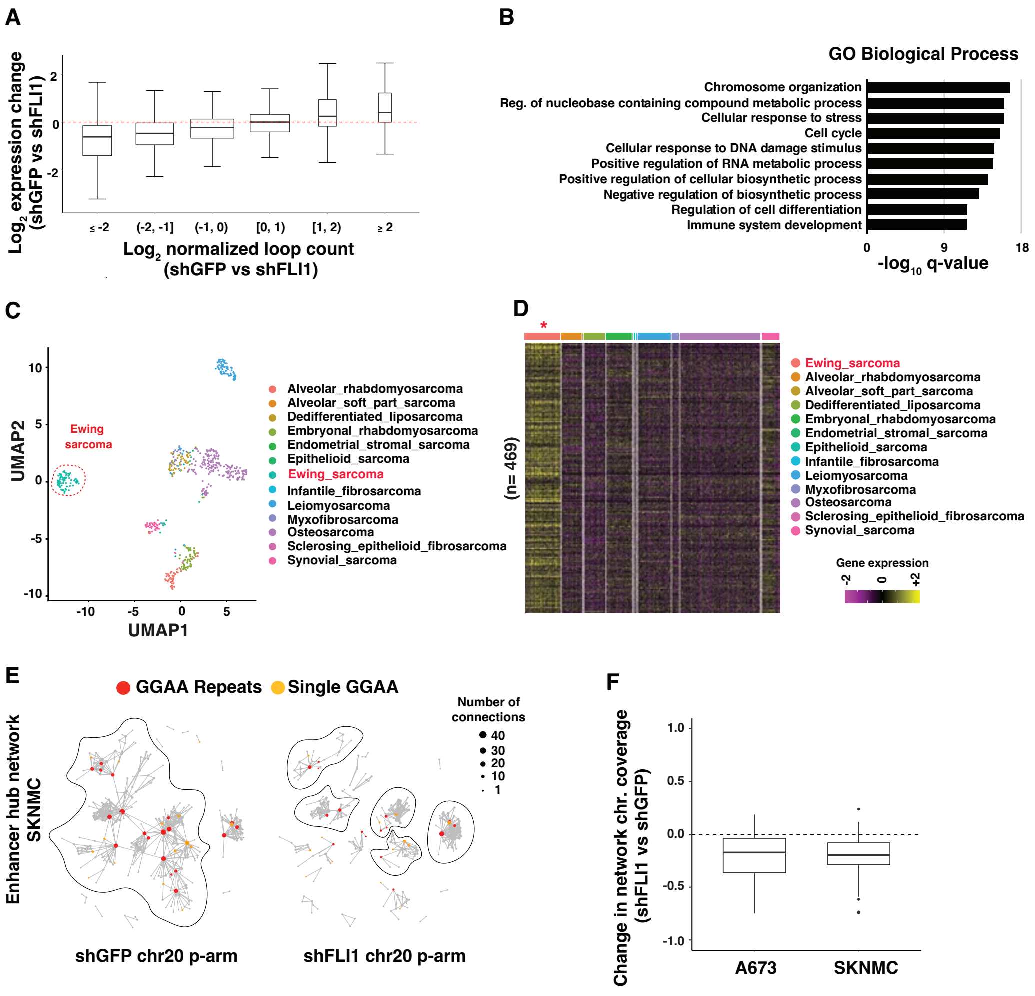

A, Boxplots showing the correlation between changes in loop counts and target gene expression between shGFP- and shFLI1-infected A673 cells. The x axis indicates the total number of loop count changes in the promoter region for EWSR1-FLI1 connected genes and y axis indicates the gene expression changes between shGFP and shFLI1 samples. B, Functional annotation (GO biological process) for 475 EWS-FLI1 direct target genes. C, UMAP gene expression clustering for 11 different sarcoma sub-types included in the Treehouse dataset. D, Heatmap showing the

expression of 469 expressed EWS-FLI1 direct target genes across the 11 sarcoma sub-types as in (C). E, Organization of EWS-FLI1-connected enhancer hubs into larger regulatory modules in shGFP-infected SKNMC cells (left), and their partial disassembly following EWS-FLI1 depletion (right). F, Boxplots depicting changes in genomic coverage for the enhancer hub networks between shGFP- and shFLI1-infected A673 and SKNMC cells genome wide. The network vertices indicate the anchors, and the edges are HiChIP loop connections between anchors.

Fig. S4. EWS-FLI1 depletion leads to modest changes in TADs insulation and A/B compartmental organization

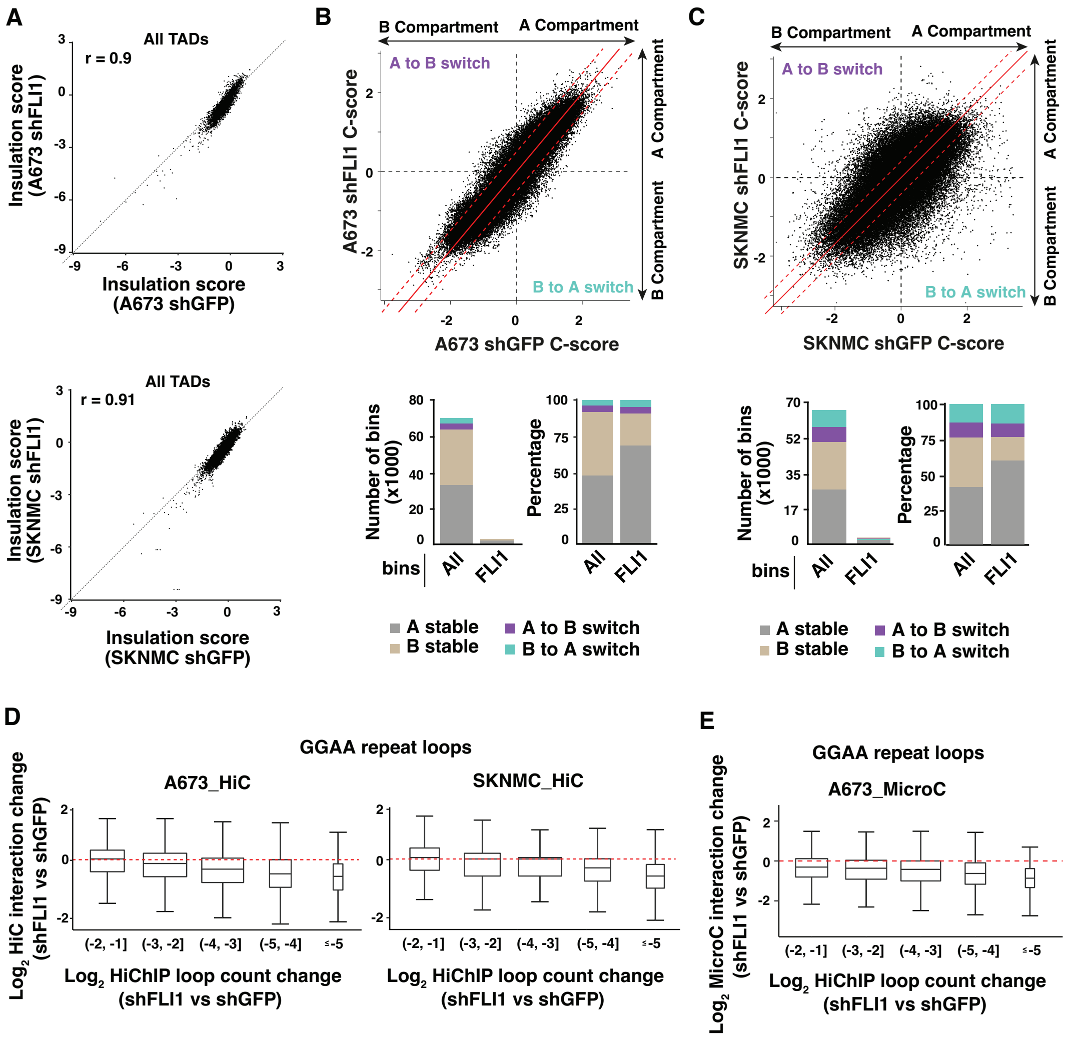

A, Analyses of TAD boundary insulation scores between shGFP- and shFLI1- infected A673 (*top*) and SKNMC (*bottom*) cells, showing no major changes in TAD organization upon EWS-FLI1 depletion. B, Scatter plots for A673 (*top*) and quantification of A/B compartmental switching between shGFP- and shFLI1-infected A673 (*bottom*) cells are shown. C, Scatter plots comparing compartment scores for SKNMC (*top*) and quantification of A/B compartmental switching between shGFP- and shFLI1-infected SKNMC (*bottom*) cells are shown. The total number and percentage of stable or switching compartments, and their further subdivision into EWS-FLI1-associated or all genomic bins are shown. 40kb bin H3K27ac segments were used for the eigenvector calculation and A/B compartmental assignment D, Boxplots depicts the positive correlation between changes in HiC interactions vs HiChIP loop counts that follow EWS-FLI1

depletion in A673 (*left*) and SKNMC (*right*) cells. E, Boxplots depicts the positive correlation between changes in MicroC interactions vs HiChIP loop counts that follow EWS-FLI1 depletion in A673 cells.

Fig. S5. EWS-FLI1 induces changes in inter-compartmental interactions and inter-TAD long range connectivity

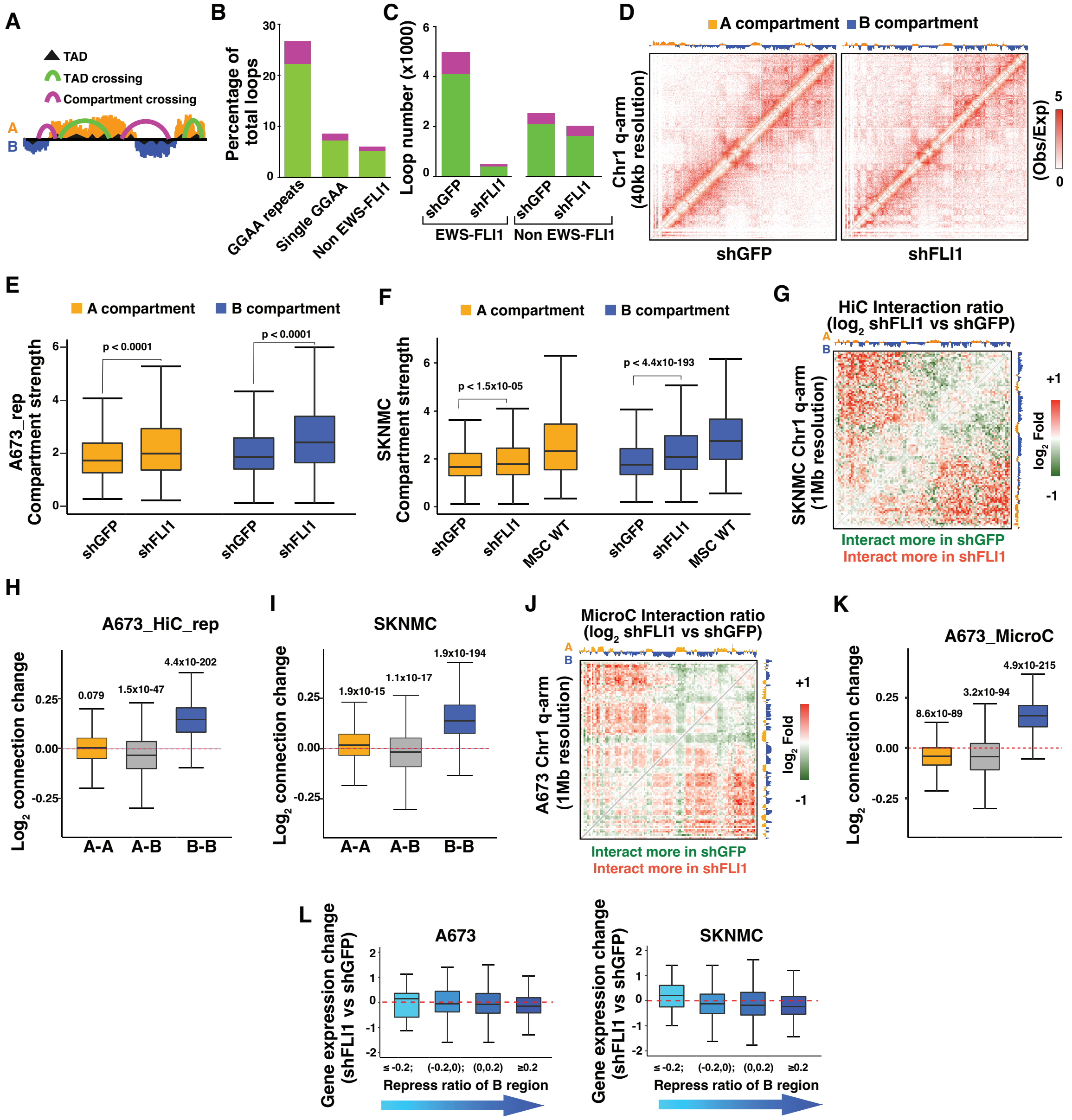

A, Graphic representation of A/B compartments, TAD boundaries and chromatin loops, illustrating that EWS-FLI1-associated loops tend to cross TAD boundaries (green) and bridge different compartments (purple) more frequently than all other loops. B, Bar plots illustrate the percentage of EWS-FLI1-associated or -independent loops that cross TADS (green) or connect compartments with opposite activity (purple) in SKNMC cells. EWS-FLI1-associated loops are further subdivided based on the underlying DNA motif (GGAA repeat or single GGAA). C,

Changes in loop number as in (A) following EWS-FLI1-depletion, and categorized based on their association with EWS-FLI1. D, HiC contact maps (Obs/Exp) for shGFP- and shFLI1-infected SKNMC cells (chr1 q-arm, 40kb resolution). E, Boxplot showing compartment strength for A/B compartment in A673 replicate sample (shGFP and shFLI1) F, Boxplot showing compartment strength for A/B compartment in SKNMC cells (shGFP, shFLI1) and MSCs. The significance in comparison among samples was calculated by unpaired two-sided t test G, Heatmap showing fold change (log2) in HiC contact frequencies between shGFP- and shFLI1- infected SKNMC cells (chr1 q-arm ,1Mb resolution). Interactions that increase (red) or decrease (green) in the shFLI1 condition are depicted. H, Boxplots showing fold change (log2) of HiC contact frequencies between shGFP and shFLI1 in A-to-A, A-to-B and B-to-B regions in A673 replicate sample. I, Boxplots showing fold change (log2) of HiC contact frequencies between shGFP and shFLI1 in A-to-A, A-to-B and B-to-B regions in SKNMC sample. The significance in each category was calculated by Wilcoxon signed rank test. J, Heatmap showing fold change (log2) in MicroC contact frequencies between shGFP- and shFLI1- infected A673 cells (chr1 q-arm ,1Mb resolution). K, Boxplots showing fold change (log2) of MicroC contact frequencies between shGFP and shFLI1 in A-to-A, A-to-B and B-to-B regions in A673 cells. L, Expression changes for non EWS-FLI1 direct target genes located in genomic segments that undergo progressive increases in B to B interactions in A673 and SKNMC cells.

Fig. S6. *de novo* H3K27ac loops emerging in EWS-FLI1-depleted cells exhibit similarity to MSC 3D looping organization

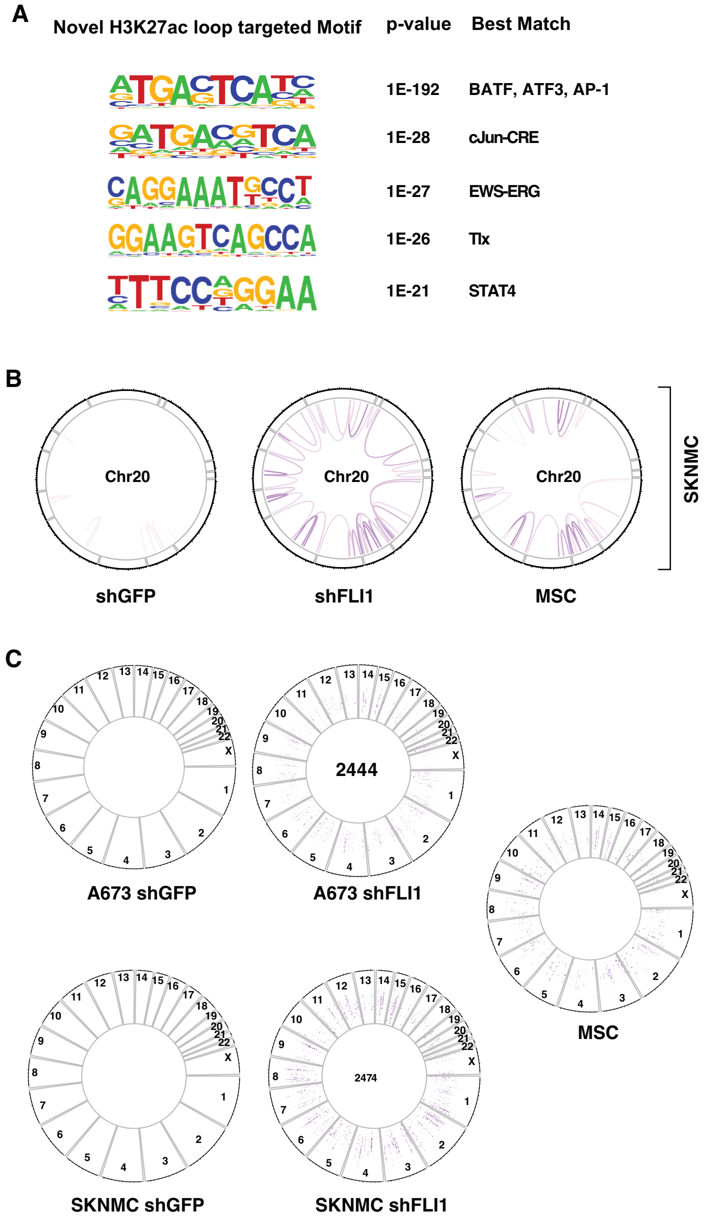

A, Homer motif enrichment analysis for the 1,473 novel H3K27ac peaks in A673 and SKNMC cells. p-value was calculated and reported by HOMER. B, Circos plots depicting the distribution of *de novo* H3K27ac loops across chromosome 20 in shGFP- and shFLI1-infected SKNMC cells, as well as wt MSCs. C, Circos plots showing the genome-wide distribution of *de novo* H3K27ac loops in shGFP- and shFLI1-infected A673 and SKNMC cells, and their comparison to wt MSCs.

Table S1. Sequencing read depth and QC metrics for HiChIP, HiC and MicroC

WT, wild type
rep, replicate

**HiChIP**	A673 shGFP	A673 shFLI1	SKNMC shGFP	SKNMC shFLI1	MSC WT	A673 shGFP rep	A673 shFLI1 rep	MSC WT rep
Total_pairs_processed	85'445'015	86'968'805	102'481'963	98'464'696	228'018'678	182'903'461	124'890'259	225'537'326
Unique_paired_alignments	58'900'200	60'642'084	71'470'561	69'459'245	143'638'701	109'102'009	77'182'362	137'283'818
valid_interaction	47'651'586	48'295'004	56'878'688	54'755'091	130'320'295	87'356'966	62'098'960	119'705'443
valid_interaction_rmdup	32'577'525	32'928'045	41'832'535	40'362'278	58'270'920	35'138'028	29'288'101	77'535'327
trans_interaction	4'387'818	4'355'132	4'080'122	5'181'127	12'873'822	5'286'964	4'268'678	14'566'400
cis_interaction	28'189'707	28'572'913	37'752'413	35'181'151	45'397'098	29'851'064	25'019'423	62'968'927
cis_shortRange	9'279'627	9'737'437	11'627'155	10'983'034	8'153'015	8'353'747	7'944'507	12'818'589
cis_longRange	18'910'080	18'835'476	26'125'258	24'198'117	37'244'083	21'497'317	17'074'916	50'150'338
loop number (≥5)	48'726	80'128	31'537	53'947	58'341	27'181	61'266	57'246
**HiC**	A673 shGFP	A673 shFLI1	SKNMC shGFP	SKNMC shFLI1	MSC WT	A673 shGFP rep	A673 shFLI1 rep	
Total_pairs_processed	873'723'575	840'655'465	105'265'357	93'647'311	79'854'869	382'159'538	393'741'134	
Unique_paired_alignments	468'598'844	435'731'565	62'785'919	55'844'533	44'817'240	222'403'504	232'346'413	
valid_interaction	320'390'112	293'326'076	47'390'988	42'340'888	36'336'301	166'695'795	176'658'196	
valid_interaction_rmdup	283'235'143	257'789'266	46'458'871	41'611'159	35'480'629	129'725'081	135'884'487	
trans_interaction	52'678'114	43'373'004	6'274'623	7'029'701	10'730'065	21'132'632	21'933'112	
cis_interaction	230'557'029	214'416'262	40'184'248	34'581'458	24'750'564	108'592'449	113'951'375	
cis_shortRange	57'511'884	60'978'073	9'109'353	8'252'853	3'236'974	22'820'704	26'851'562	
cis_longRange	173'045'145	153'438'189	31'074'895	26'328'605	21'513'590	85'771'745	87'099'813	
**MicroC**	A673 shGFP	A673 shFLI1						
Total_pairs_processed	554'251'739	562'648'119						
Unique_paired_alignments	326'867'670	342'674'821						
valid_interaction	258'906'611	265'565'629						
valid_interaction_rmdup	207'732'605	164'060'037						
trans_interaction	32'123'621	37'566'543						
cis_interaction	175'608'984	126'493'494						
cis_shortRange	56'899'184	42'934'539						
cis_longRange	118'709'800	83'558'955						

Table S2. EWS-FLI1 target genes in A673 and SKNMC cells

A673_EWS-FLI1_target genes				
Target Name	shGFP_RPKM	shFLI1_RPKM	Loop_Target_Category	Log2 Fold Change
UTS2	64.27070934	5.484270415	4_EWS-FLI1_loop_and_bridge_target	-3.331419556
PARK7	160.571094	178.7723286	3_EWS-FLI1_bridge_target	0.154001857
ERRFI1	58.45744355	261.596282	4_EWS-FLI1_loop_and_bridge_target	2.142917151
RERE	9.191024218	5.140554181	4_EWS-FLI1_loop_and_bridge_target	-0.730858284
LOC102724552	0.459394638	0.120012128	4_EWS-FLI1_loop_and_bridge_target	-0.381855703
ENO1	441.4395919	428.1831516	4_EWS-FLI1_loop_and_bridge_target	-0.04388705
ENO1-AS1	1.601247822	0.506880181	4_EWS-FLI1_loop_and_bridge_target	-0.787639146
PGD	155.0930305	132.2563026	3_EWS-FLI1_bridge_target	-0.228202354
TARDBP	49.70475246	27.63959937	4_EWS-FLI1_loop_and_bridge_target	-0.824109663
MAD2L2	52.06751153	28.16001451	3_EWS-FLI1_bridge_target	-0.863837461
MFN2	60.84094894	63.27721671	4_EWS-FLI1_loop_and_bridge_target	0.055745002
PDPN	17.18631722	20.02355761	4_EWS-FLI1_loop_and_bridge_target	0.2091534
PRDM2	6.36641678	3.719862518	4_EWS-FLI1_loop_and_bridge_target	-0.642218188
PAX7	19.86349354	7.194041502	4_EWS-FLI1_loop_and_bridge_target	-1.348333649
ALDH4A1	18.57898138	8.469389043	3_EWS-FLI1_bridge_target	-1.047962456
UBR4	31.74949756	31.08313477	4_EWS-FLI1_loop_and_bridge_target	-0.029657662
LOC101927895	0.977850107	0.709592478	4_EWS-FLI1_loop_and_bridge_target	-0.210280629
RAP1GAP	14.99410954	3.983761885	1_EWS-FLI1_in_promoter	-1.682233628
SNHG3	55.16426479	30.22260854	3_EWS-FLI1_bridge_target	-0.847061415
RCC1	48.49179788	25.33754007	3_EWS-FLI1_bridge_target	-0.910068848
SNORA73B	498.811861	578.9523965	3_EWS-FLI1_bridge_target	0.214549348
TAF12	19.10280763	16.66588917	3_EWS-FLI1_bridge_target	-0.186430642
RNU11	61.21341673	45.03744035	3_EWS-FLI1_bridge_target	-0.434418118
YTHDF2	61.34081977	41.8598005	3_EWS-FLI1_bridge_target	-0.540551992
EPB41	102.200722	31.44022392	4_EWS-FLI1_loop_and_bridge_target	-1.669597381
SRSF4	52.55361354	37.37397236	4_EWS-FLI1_loop_and_bridge_target	-0.480855808
MATN1-AS1	0.174184519	0.818373994	4_EWS-FLI1_loop_and_bridge_target	0.630989815
YRDC	35.73699948	43.16066431	4_EWS-FLI1_loop_and_bridge_target	0.265528077
C1orf122	14.8814863	20.21274288	4_EWS-FLI1_loop_and_bridge_target	0.417585242
INPP5B	11.19390329	8.682435656	3_EWS-FLI1_bridge_target	-0.332718095
SF3A3	95.00988617	55.36874369	4_EWS-FLI1_loop_and_bridge_target	-0.768287554
FHL3	30.23882447	21.2762807	4_EWS-FLI1_loop_and_bridge_target	-0.487831788
UTP11	59.8634867	40.2164071	4_EWS-FLI1_loop_and_bridge_target	-0.562358236
POU3F1	18.91577505	1.121565335	4_EWS-FLI1_loop_and_bridge_target	-3.230710612
RRAGC	14.12466554	20.80142475	4_EWS-FLI1_loop_and_bridge_target	0.52751918
PABPC4	41.71326254	21.47569627	3_EWS-FLI1_bridge_target	-0.926318289
ELOVL1	49.84729169	79.30453663	4_EWS-FLI1_loop_and_bridge_target	0.659310559
PTPRF	29.630088	24.02748741	2_EWS-FLI1_loop_target	-0.29143605
KDM4A-AS1	2.321662707	0.686442998	4_EWS-FLI1_loop_and_bridge_target	-0.977922029
ST3GAL3	9.239677322	6.849308336	4_EWS-FLI1_loop_and_bridge_target	-0.383532815
ARTN	9.590836683	1.181265763	4_EWS-FLI1_loop_and_bridge_target	-2.279579106
IPO13	20.36267018	18.24491736	4_EWS-FLI1_loop_and_bridge_target	-0.150614509
DPH2	23.2595474	24.19374327	4_EWS-FLI1_loop_and_bridge_target	0.054512858
ATP6V0B	55.80272888	65.52720241	4_EWS-FLI1_loop_and_bridge_target	0.227984127
CCDC24	2.784491207	1.767242252	4_EWS-FLI1_loop_and_bridge_target	-0.451650408
SLC6A9	3.345556239	5.224675765	4_EWS-FLI1_loop_and_bridge_target	0.518457835
RNF11	26.5883748	67.84069552	4_EWS-FLI1_loop_and_bridge_target	1.319201202
EPS15	36.28603997	20.90836348	4_EWS-FLI1_loop_and_bridge_target	-0.76715386

OSBPL9	45.72796533	45.13435257	4_EWS-FLI1_loop_and_bridge_target	-0.018444809
NRDC	148.5660021	91.89300192	4_EWS-FLI1_loop_and_bridge_target	-0.687140452
TXNDC12	26.47890708	30.72234813	4_EWS-FLI1_loop_and_bridge_target	0.20717494
BTF3L4	19.50960671	9.21013418	4_EWS-FLI1_loop_and_bridge_target	-1.006298001
ZFYVE9	19.28261904	21.00475933	4_EWS-FLI1_loop_and_bridge_target	0.117571637
CC2D1B	8.264280409	6.652046248	4_EWS-FLI1_loop_and_bridge_target	-0.275833328
ORC1	35.55410071	3.07126742	4_EWS-FLI1_loop_and_bridge_target	-3.166483367
PRPF38A	35.72806896	12.24173189	4_EWS-FLI1_loop_and_bridge_target	-1.471791221
OMA1	9.363479377	6.029151185	4_EWS-FLI1_loop_and_bridge_target	-0.560086057
MYSM1	8.110042231	8.229893059	4_EWS-FLI1_loop_and_bridge_target	0.018856191
JUN	17.46169664	27.08010397	4_EWS-FLI1_loop_and_bridge_target	0.605013134
LINC01135	0.056780032	0.084761142	4_EWS-FLI1_loop_and_bridge_target	0.037702293
FGGY	1.37498611	1.601700328	4_EWS-FLI1_loop_and_bridge_target	0.131535722
LOC101926944	0.86950876	0.09013898	4_EWS-FLI1_loop_and_bridge_target	-0.778147159
HOOK1	142.4254459	47.58322382	4_EWS-FLI1_loop_and_bridge_target	-1.561770872
NFIA	6.790548511	3.731068469	4_EWS-FLI1_loop_and_bridge_target	-0.719558868
NFIA-AS1	0.422924329	0.18211753	3_EWS-FLI1_bridge_target	-0.267485461
ATG4C	10.62264765	4.625678293	2_EWS-FLI1_loop_target	-1.046839795
FOXD3-AS1	3.046214226	1.088674509	4_EWS-FLI1_loop_and_bridge_target	-0.953985019
FOXD3	0.518005219	0.379734424	4_EWS-FLI1_loop_and_bridge_target	-0.137786152
ALG6	19.79690805	5.174421301	4_EWS-FLI1_loop_and_bridge_target	-1.751993222
ITGB3BP	42.09723706	7.001407267	4_EWS-FLI1_loop_and_bridge_target	-2.429269717
EFCAB7	10.98481814	5.191284709	4_EWS-FLI1_loop_and_bridge_target	-0.952897308
RAVER2	27.48240514	12.9842296	3_EWS-FLI1_bridge_target	-1.026270201
JAK1	228.6995165	159.9064204	4_EWS-FLI1_loop_and_bridge_target	-0.513525927
LINC01359	0.73278341	0.463142264	4_EWS-FLI1_loop_and_bridge_target	-0.244021284
PDE4B	10.45873401	13.86543511	4_EWS-FLI1_loop_and_bridge_target	0.375514031
SGIP1	0.21591548	0.195019709	4_EWS-FLI1_loop_and_bridge_target	-0.025008537
GADD45A	28.61985984	69.39839224	2_EWS-FLI1_loop_target	1.248977667
ANKRD13C	16.22960272	16.73368837	4_EWS-FLI1_loop_and_bridge_target	0.041603193
HHLA3	1.91761273	1.626482533	4_EWS-FLI1_loop_and_bridge_target	-0.15165641
CTH	11.81719289	15.62352817	4_EWS-FLI1_loop_and_bridge_target	0.375146282
PTGER3	15.59116045	10.53721163	4_EWS-FLI1_loop_and_bridge_target	-0.524120209
ZRANB2-AS1	1.925093779	1.221071236	4_EWS-FLI1_loop_and_bridge_target	-0.397227214
ZRANB2	49.9974449	24.42849462	4_EWS-FLI1_loop_and_bridge_target	-1.00397891
ZRANB2-AS2	0.211732231	0.227937649	4_EWS-FLI1_loop_and_bridge_target	0.01916638
NEGR1	5.430454665	4.884009438	4_EWS-FLI1_loop_and_bridge_target	-0.128121186
ZZZ3	32.47910872	28.0799502	4_EWS-FLI1_loop_and_bridge_target	-0.203236323
USP33	72.42208965	39.58225746	4_EWS-FLI1_loop_and_bridge_target	-0.855365056
NEXN-AS1	0.139101169	0.114207503	3_EWS-FLI1_bridge_target	-0.031877949
NEXN	2.536259644	1.516696715	3_EWS-FLI1_bridge_target	-0.490692837
FUBP1	81.63083597	32.45081701	4_EWS-FLI1_loop_and_bridge_target	-1.304638815
DNAJB4	15.29446006	17.98794906	4_EWS-FLI1_loop_and_bridge_target	0.220702537
KYAT3	30.31751858	19.61678862	4_EWS-FLI1_loop_and_bridge_target	-0.603150278
RBMXL1	16.41793895	6.754948146	4_EWS-FLI1_loop_and_bridge_target	-1.167384882
GBP1	26.38365161	23.68355879	4_EWS-FLI1_loop_and_bridge_target	-0.149764431
FLJ27354	0.95363107	0.346846568	3_EWS-FLI1_bridge_target	-0.536572541
LRRC8C	7.872510841	5.182309683	3_EWS-FLI1_bridge_target	-0.521196509
GEMIN8P4	4.533161614	0.810933052	4_EWS-FLI1_loop_and_bridge_target	-1.61137085
ZNF326	4.950403209	5.48621885	4_EWS-FLI1_loop_and_bridge_target	0.124390271
BARHL2	18.53247103	4.525507001	1_EWS-FLI1_in_promoter	-1.821695722
ZNF644	46.45281607	29.53668553	4_EWS-FLI1_loop_and_bridge_target	-0.635950226
CDC7	20.93474952	4.146129108	2_EWS-FLI1_loop_target	-2.091658675
OLFM3	55.59935291	35.09483047	4_EWS-FLI1_loop_and_bridge_target	-0.648993331
PRMT6	31.96861064	14.43965225	4_EWS-FLI1_loop_and_bridge_target	-1.094452831
NTNG1	25.40143862	20.30744505	4_EWS-FLI1_loop_and_bridge_target	-0.309258933

SARS	58.72970281	79.9191682	3_EWS-FLI1_bridge_target	0.438032946
CYB561D1	3.600556475	2.503336174	4_EWS-FLI1_loop_and_bridge_target	-0.393078944
GNAI3	68.24578014	56.13494479	4_EWS-FLI1_loop_and_bridge_target	-0.277352761
AMPD2	29.31646329	35.44569923	4_EWS-FLI1_loop_and_bridge_target	0.265647121
GSTM4	59.79910048	9.719775031	1_EWS-FLI1_in_promoter	-2.50377535
GSTM1	45.57644512	9.920308146	4_EWS-FLI1_loop_and_bridge_target	-2.092586966
GSTM5	0.024368398	0.068207075	4_EWS-FLI1_loop_and_bridge_target	0.060456693
GSTM3	39.32137589	7.743870964	4_EWS-FLI1_loop_and_bridge_target	-2.205200853
EPS8L3	0.005494526	0	4_EWS-FLI1_loop_and_bridge_target	-0.007905228
AHCYL1	40.44289203	41.49811372	4_EWS-FLI1_loop_and_bridge_target	0.036274126
STRIP1	24.08574469	26.43235644	4_EWS-FLI1_loop_and_bridge_target	0.129010789
LOC440600	0.229736057	0.123133508	4_EWS-FLI1_loop_and_bridge_target	-0.130819264
RBM15	29.28942751	16.1465158	4_EWS-FLI1_loop_and_bridge_target	-0.820898863
KCNA2	14.86017194	2.307941446	1_EWS-FLI1_in_promoter	-2.261402809
LRIF1	19.72381331	29.60850603	4_EWS-FLI1_loop_and_bridge_target	0.562643138
CTTNBP2NL	12.64039763	10.41206047	4_EWS-FLI1_loop_and_bridge_target	-0.257326405
PHTF1	18.6847454	13.59890673	4_EWS-FLI1_loop_and_bridge_target	-0.431217718
RSBN1	7.908284751	6.756932401	4_EWS-FLI1_loop_and_bridge_target	-0.199661445
AP4B1-AS1	0.979614153	0.504073288	4_EWS-FLI1_loop_and_bridge_target	-0.396344396
AP4B1	15.55701714	8.827142631	4_EWS-FLI1_loop_and_bridge_target	-0.752598884
DCLRE1B	21.14175349	4.145091412	4_EWS-FLI1_loop_and_bridge_target	-2.105500864
HIPK1-AS1	0.150953735	0.109541978	4_EWS-FLI1_loop_and_bridge_target	-0.052865592
HIPK1	24.55010565	16.08837656	4_EWS-FLI1_loop_and_bridge_target	-0.580313913
OLFML3	311.5532601	68.12971119	4_EWS-FLI1_loop_and_bridge_target	-2.176724247
SYT6	13.48858673	2.896222522	4_EWS-FLI1_loop_and_bridge_target	-1.894768896
BCAS2	60.46059992	49.46821654	3_EWS-FLI1_bridge_target	-0.284286744
NRAS	76.2419015	57.68587076	4_EWS-FLI1_loop_and_bridge_target	-0.396370479
CSDE1	202.8945167	220.1318022	4_EWS-FLI1_loop_and_bridge_target	0.117083545
SIKE1	24.0013438	25.95223451	3_EWS-FLI1_bridge_target	0.108399246
MAB21L3	6.571895674	0.346374871	4_EWS-FLI1_loop_and_bridge_target	-2.491574379
ATP1A1	403.8810224	303.9529153	4_EWS-FLI1_loop_and_bridge_target	-0.408911515
ATP1A1-AS1	1.510008382	0.903100212	4_EWS-FLI1_loop_and_bridge_target	-0.39934065
LOC101929099	0.091392689	0.03279588	4_EWS-FLI1_loop_and_bridge_target	-0.079615133
TTF2	20.82115813	7.218917779	4_EWS-FLI1_loop_and_bridge_target	-1.408707327
ETV3	12.53517066	10.53795428	3_EWS-FLI1_bridge_target	-0.230325626
KIRREL	56.44171869	91.46358451	4_EWS-FLI1_loop_and_bridge_target	0.686786376
POP3	2.125465859	7.340791982	4_EWS-FLI1_loop_and_bridge_target	1.416113135
IFI16	41.25102966	84.5184321	4_EWS-FLI1_loop_and_bridge_target	1.017248902
ADAMTS4	7.753136441	11.80532535	3_EWS-FLI1_bridge_target	0.548871945
NDUFS2	61.07888174	29.84261902	3_EWS-FLI1_bridge_target	-1.009177292
DUSP12	37.93806407	39.74831836	4_EWS-FLI1_loop_and_bridge_target	0.065559364
ATF6	24.07753413	30.78022774	4_EWS-FLI1_loop_and_bridge_target	0.341733968
NOS1AP	2.606126765	0.276504756	4_EWS-FLI1_loop_and_bridge_target	-1.498251199
UHMK1	70.73544485	44.31439622	4_EWS-FLI1_loop_and_bridge_target	-0.662716676
UAP1	72.82051644	165.3499707	4_EWS-FLI1_loop_and_bridge_target	1.172127876
DDR2	75.62832094	53.28213999	4_EWS-FLI1_loop_and_bridge_target	-0.497400096
HSD17B7	19.0890896	33.57932098	4_EWS-FLI1_loop_and_bridge_target	0.783497354
RGS4	16.2695916	26.35402725	4_EWS-FLI1_loop_and_bridge_target	0.663519287
LOC101928404	0.019373787	0.013556793	4_EWS-FLI1_loop_and_bridge_target	-0.00825623
PBX1	5.244847955	1.313420124	4_EWS-FLI1_loop_and_bridge_target	-1.432639159
LOC100505795	0.012993169	0.012122614	4_EWS-FLI1_loop_and_bridge_target	-0.001240369
LOC101928650	0.382181558	0.412876335	2_EWS-FLI1_loop_target	0.031688061
GORAB	8.04125334	9.587021824	2_EWS-FLI1_loop_target	0.227702125
VAMP4	5.67258182	4.833197876	3_EWS-FLI1_bridge_target	-0.193958076
IER5	21.50877064	21.47493833	4_EWS-FLI1_loop_and_bridge_target	-0.002170106
LOC284648	2.379380572	3.545547189	4_EWS-FLI1_loop_and_bridge_target	0.427695145

LAMC1	52.12173584	108.7185096	4_EWS-FLI1_loop_and_bridge_target	1.046432735
SMG7-AS1	0.389755353	0.635160242	4_EWS-FLI1_loop_and_bridge_target	0.234601084
SMG7	10.67490391	6.035335781	4_EWS-FLI1_loop_and_bridge_target	-0.730719489
ARPC5	121.4388892	84.70868936	4_EWS-FLI1_loop_and_bridge_target	-0.514548481
RGL1	69.1709573	42.05434744	4_EWS-FLI1_loop_and_bridge_target	-0.704715122
APOBEC4	0.201988116	0.089944295	4_EWS-FLI1_loop_and_bridge_target	-0.141168229
COLGALT2	13.73143214	7.353344145	4_EWS-FLI1_loop_and_bridge_target	-0.81847191
TSEN15	40.08436707	33.76352156	4_EWS-FLI1_loop_and_bridge_target	-0.241015306
C1orf21	5.215927858	4.056149144	4_EWS-FLI1_loop_and_bridge_target	-0.29793074
EDEM3	19.49374867	55.01230764	4_EWS-FLI1_loop_and_bridge_target	1.450559964
FAM129A	58.51573117	70.06348055	4_EWS-FLI1_loop_and_bridge_target	0.255837299
RNF2	23.68837984	18.89461254	4_EWS-FLI1_loop_and_bridge_target	-0.311454362
TRMT1L	9.159107688	4.271403322	4_EWS-FLI1_loop_and_bridge_target	-0.946514707
SWT1	4.457821169	3.941986642	4_EWS-FLI1_loop_and_bridge_target	-0.143234013
IVNS1ABP	85.20702445	40.91137026	4_EWS-FLI1_loop_and_bridge_target	-1.04046374
HMCN1	54.11885164	28.01259342	4_EWS-FLI1_loop_and_bridge_target	-0.925866568
TPR	61.2754332	48.35342385	4_EWS-FLI1_loop_and_bridge_target	-0.335512978
C1orf27	20.75602517	23.52488548	4_EWS-FLI1_loop_and_bridge_target	0.1728314
RGS21	0.362739851	0.072995983	4_EWS-FLI1_loop_and_bridge_target	-0.3448655
RGS2	17.14542459	16.14649525	4_EWS-FLI1_loop_and_bridge_target	-0.081692096
UCHL5	21.52580189	10.96915499	4_EWS-FLI1_loop_and_bridge_target	-0.912257162
SCARNA18B	1.983768135	0.705087129	4_EWS-FLI1_loop_and_bridge_target	-0.807289968
TROVE2	21.84969766	33.97837511	4_EWS-FLI1_loop_and_bridge_target	0.614288196
CDC73	13.7019003	13.46702763	4_EWS-FLI1_loop_and_bridge_target	-0.023234104
TMEM9	27.13130073	27.15288107	4_EWS-FLI1_loop_and_bridge_target	0.001106309
IGFN1	0.032394907	0.138024835	4_EWS-FLI1_loop_and_bridge_target	0.140537112
PHLDA3	8.83302667	9.332448595	4_EWS-FLI1_loop_and_bridge_target	0.071474725
CSRP1	263.3693646	203.6695672	4_EWS-FLI1_loop_and_bridge_target	-0.369258405
RPS10P7	2.498733448	1.46786553	1_EWS-FLI1_in_promoter	-0.50356897
NAV1	3.608084448	4.626586132	4_EWS-FLI1_loop_and_bridge_target	0.288092693
IPO9-AS1	0.348354262	0.084786309	4_EWS-FLI1_loop_and_bridge_target	-0.31378872
IPO9	26.28644207	13.90572205	4_EWS-FLI1_loop_and_bridge_target	-0.872318028
TIMM17A	77.78513209	99.9131093	4_EWS-FLI1_loop_and_bridge_target	0.3571183
LAMB3	15.44520952	12.19216428	4_EWS-FLI1_loop_and_bridge_target	-0.317986119
TRAF3IP3	0.459448316	0.054065834	4_EWS-FLI1_loop_and_bridge_target	-0.469458145
C1orf74	5.182546825	2.28899166	4_EWS-FLI1_loop_and_bridge_target	-0.910555911
IRF6	0.956813487	0.652362106	3_EWS-FLI1_bridge_target	-0.243976373
DIEXF	6.697997642	6.17070677	4_EWS-FLI1_loop_and_bridge_target	-0.102367906
CENPF	70.62219514	6.131761863	3_EWS-FLI1_bridge_target	-3.3280763
SLC35F3	13.62913731	1.991039955	4_EWS-FLI1_loop_and_bridge_target	-2.290125608
LOC101927765	2.108860657	0.898572952	4_EWS-FLI1_loop_and_bridge_target	-0.711470519
COA6	25.60553384	13.36232665	4_EWS-FLI1_loop_and_bridge_target	-0.889436871
IRF2BP2	13.93426792	20.81636306	4_EWS-FLI1_loop_and_bridge_target	0.546784096
B3GALNT2	8.304885668	7.653025874	3_EWS-FLI1_bridge_target	-0.104783707
HEATR1	23.10955595	16.10007119	4_EWS-FLI1_loop_and_bridge_target	-0.495602748
KMO	10.28748078	0.25080067	4_EWS-FLI1_loop_and_bridge_target	-3.173799729
EXO1	21.72616574	1.00355772	3_EWS-FLI1_bridge_target	-3.503718322
CEP170	8.849527658	8.812428462	2_EWS-FLI1_loop_target	-0.00544431
SDCCAG8	4.010527478	5.469510658	2_EWS-FLI1_loop_target	0.368694104
TMEM18	17.08778484	7.05834043	3_EWS-FLI1_bridge_target	-1.166461076
LINC01250	0.369961634	0.192891263	2_EWS-FLI1_loop_target	-0.199672949
LINC01304	0.401164086	0.019699247	4_EWS-FLI1_loop_and_bridge_target	-0.458482212
LINC01249	0.615224598	0.097892145	4_EWS-FLI1_loop_and_bridge_target	-0.556998452
LINC01248	0.108998249	0.186441308	4_EWS-FLI1_loop_and_bridge_target	0.097383646
SOX11	9.192719065	9.721735508	4_EWS-FLI1_loop_and_bridge_target	0.072999487
ID2-AS1	1.345813093	0.46141072	4_EWS-FLI1_loop_and_bridge_target	-0.682726373

ID2	117.0283995	39.84724437	4_EWS-FLI1_loop_and_bridge_target	-1.530823377
ADAM17	46.67971309	29.21490536	3_EWS-FLI1_bridge_target	-0.658115132
FAM49A	71.40371152	15.19007734	4_EWS-FLI1_loop_and_bridge_target	-2.160953776
SMC6	30.71680095	13.46850153	4_EWS-FLI1_loop_and_bridge_target	-1.132331751
GEN1	8.618867117	3.142574942	4_EWS-FLI1_loop_and_bridge_target	-1.215339191
DNMT3A	12.46138655	8.191726139	4_EWS-FLI1_loop_and_bridge_target	-0.550419298
AGBL5-AS1	0.125334596	0.526216745	3_EWS-FLI1_bridge_target	0.439605839
AGBL5	27.2756708	10.75903083	3_EWS-FLI1_bridge_target	-1.265792092
SLC5A6	138.7768793	78.20091705	2_EWS-FLI1_loop_target	-0.819536702
ATRAID	89.05063395	50.21476198	2_EWS-FLI1_loop_target	-0.814176725
CAD	83.2947184	23.56253705	2_EWS-FLI1_loop_target	-1.778982657
GTF3C2	21.48162171	15.94716353	3_EWS-FLI1_bridge_target	-0.407702281
ZNF513	6.186785073	5.696446538	3_EWS-FLI1_bridge_target	-0.101950808
PPM1G	94.97563861	63.27004552	3_EWS-FLI1_bridge_target	-0.57852176
CLIP4	8.659152849	13.28825274	4_EWS-FLI1_loop_and_bridge_target	0.564860936
YPEL5	169.9528023	79.34200374	4_EWS-FLI1_loop_and_bridge_target	-1.089371724
LBH	180.2232998	16.8188659	4_EWS-FLI1_loop_and_bridge_target	-3.34629103
LCLAT1	14.76272128	10.31175456	4_EWS-FLI1_loop_and_bridge_target	-0.478693901
SLC1A4	53.9471527	40.232245	4_EWS-FLI1_loop_and_bridge_target	-0.414271709
ACTR2	121.7265149	72.41157057	4_EWS-FLI1_loop_and_bridge_target	-0.741367602
SPRED2	11.12921349	7.901524791	4_EWS-FLI1_loop_and_bridge_target	-0.446361613
MEIS1-AS3	0.544459417	0.159650888	4_EWS-FLI1_loop_and_bridge_target	-0.413411414
MEIS1	23.03601413	7.716531389	4_EWS-FLI1_loop_and_bridge_target	-1.463371619
MEIS1-AS2	2.947150948	0.746829091	4_EWS-FLI1_loop_and_bridge_target	-1.176073229
LOC100507073	0.283395448	0.046660178	4_EWS-FLI1_loop_and_bridge_target	-0.294172657
ETAA1	19.51181543	10.66610728	4_EWS-FLI1_loop_and_bridge_target	-0.814131939
LOC101927701	4.503055943	1.066748199	4_EWS-FLI1_loop_and_bridge_target	-1.412870365
FBXO48	1.379177558	0.44179148	4_EWS-FLI1_loop_and_bridge_target	-0.722600415
APLF	6.613296732	4.63383206	4_EWS-FLI1_loop_and_bridge_target	-0.43440475
PROKR1	12.11623431	1.288484093	4_EWS-FLI1_loop_and_bridge_target	-2.51888941
GKN1	0.870922596	0.217652658	4_EWS-FLI1_loop_and_bridge_target	-0.619647217
ANTXR1	34.1405378	20.94863799	4_EWS-FLI1_loop_and_bridge_target	-0.679004854
GFPT1	31.46070457	67.18197034	3_EWS-FLI1_bridge_target	1.070695977
NFU1	38.90991016	23.15892334	3_EWS-FLI1_bridge_target	-0.72419087
EXOC6B	47.86141958	24.86060827	2_EWS-FLI1_loop_target	-0.917939569
SMYD5	35.03913681	29.34492562	3_EWS-FLI1_bridge_target	-0.248109173
TMEM37	15.73121915	2.440324524	3_EWS-FLI1_bridge_target	-2.281926009
SCTR	0.860446409	0.221910993	3_EWS-FLI1_bridge_target	-0.606509634
RNU4ATAC	19.0310943	23.79669059	4_EWS-FLI1_loop_and_bridge_target	0.307906351
NIFK	45.40523383	43.08871421	3_EWS-FLI1_bridge_target	-0.073878126
TSN	35.56633026	38.42674703	4_EWS-FLI1_loop_and_bridge_target	0.108658836
GPR39	1.075918201	2.454745467	4_EWS-FLI1_loop_and_bridge_target	0.734829825
NCKAP5	10.29090449	8.959012169	4_EWS-FLI1_loop_and_bridge_target	-0.181086508
LOC101928161	5.139544562	3.943707669	4_EWS-FLI1_loop_and_bridge_target	-0.312538202
LRP1B	3.411339916	3.579364295	4_EWS-FLI1_loop_and_bridge_target	0.053930406
KYNU	3.343334578	7.39658804	4_EWS-FLI1_loop_and_bridge_target	0.951000115
GTDC1	9.752466128	5.507383048	4_EWS-FLI1_loop_and_bridge_target	-0.724518203
ZEB2	20.70250428	15.34351649	4_EWS-FLI1_loop_and_bridge_target	-0.409143098
ZEB2-AS1	0.713443085	0.44376113	4_EWS-FLI1_loop_and_bridge_target	-0.247066203
LINC01412	0.060296904	0	4_EWS-FLI1_loop_and_bridge_target	-0.084468304
LOC105373656	2.043474594	0.776746577	4_EWS-FLI1_loop_and_bridge_target	-0.776481403
ACVR2A	8.106682202	8.907910543	2_EWS-FLI1_loop_target	0.121655304
RBM43	2.857486292	1.963804564	4_EWS-FLI1_loop_and_bridge_target	-0.380210712
NMI	75.87007744	39.4278501	4_EWS-FLI1_loop_and_bridge_target	-0.927072637
TNFAIP6	121.1605096	65.91716717	4_EWS-FLI1_loop_and_bridge_target	-0.868329707
RIF1	46.15136212	27.83527818	4_EWS-FLI1_loop_and_bridge_target	-0.709464508

PSMD14	172.9388948	122.5037555	4_EWS-FLI1_loop_and_bridge_target	-0.49402566
SCN2A	0.599413285	0.078968454	2_EWS-FLI1_loop_target	-0.567890091
CSRNP3	16.01090129	3.125786227	4_EWS-FLI1_loop_and_bridge_target	-2.043718605
TTC21B	32.11366637	7.857643772	4_EWS-FLI1_loop_and_bridge_target	-1.902431873
DYNC1I2	51.21325916	37.76641806	4_EWS-FLI1_loop_and_bridge_target	-0.429608776
HAT1	105.55038	60.58893917	4_EWS-FLI1_loop_and_bridge_target	-0.790792554
DLX1	10.14548565	8.536638167	4_EWS-FLI1_loop_and_bridge_target	-0.224906796
DLX2	3.503697626	2.24643238	4_EWS-FLI1_loop_and_bridge_target	-0.47225481
DLX2-AS1	0.469839821	0.112721166	4_EWS-FLI1_loop_and_bridge_target	-0.401566827
ATF2	25.95766795	31.71747693	4_EWS-FLI1_loop_and_bridge_target	0.279365799
KIAA1715	14.16737015	11.6375557	3_EWS-FLI1_bridge_target	-0.26325351
EVX2	0.220827622	0.095657693	4_EWS-FLI1_loop_and_bridge_target	-0.15606237
HOXD13	23.26960387	7.132871375	4_EWS-FLI1_loop_and_bridge_target	-1.577313859
HOXD12	0.219605329	0.043905333	4_EWS-FLI1_loop_and_bridge_target	-0.224423472
HOXD11	28.2252568	12.82548909	4_EWS-FLI1_loop_and_bridge_target	-1.079885184
HOXD10	24.66435825	20.01085345	4_EWS-FLI1_loop_and_bridge_target	-0.288631421
HOXD9	17.16384154	6.539355824	4_EWS-FLI1_loop_and_bridge_target	-1.268556188
HOXD8	26.63583042	9.24169688	4_EWS-FLI1_loop_and_bridge_target	-1.432085198
HOXD-AS2	6.08153526	2.389985544	4_EWS-FLI1_loop_and_bridge_target	-1.062783046
LINC01116	32.97887291	15.68827192	4_EWS-FLI1_loop_and_bridge_target	-1.025803428
LINC01117	3.168758551	1.21522734	4_EWS-FLI1_loop_and_bridge_target	-0.912163051
HNRNPA3	20.33245232	16.23321077	4_EWS-FLI1_loop_and_bridge_target	-0.307858305
NFE2L2	33.01707204	54.4023952	4_EWS-FLI1_loop_and_bridge_target	0.70368938
LOC100130691	0.181272924	0.514238896	4_EWS-FLI1_loop_and_bridge_target	0.358250505
AGPS	17.46459109	14.64901299	4_EWS-FLI1_loop_and_bridge_target	-0.238689647
TTC30B	5.701136108	1.768551324	4_EWS-FLI1_loop_and_bridge_target	-1.275274443
PPP1R1C	1.111185798	1.087969263	4_EWS-FLI1_loop_and_bridge_target	-0.015953077
PDE1A	1.822409654	0.157682047	2_EWS-FLI1_loop_target	-1.285688324
DNAJC10	30.41237296	116.2463646	4_EWS-FLI1_loop_and_bridge_target	1.900138354
NCKAP1	124.3213165	108.9167174	4_EWS-FLI1_loop_and_bridge_target	-0.189221006
DUSP19	4.502105129	0.898197548	4_EWS-FLI1_loop_and_bridge_target	-1.535353562
NUP35	49.26927339	20.28531451	3_EWS-FLI1_bridge_target	-1.239818428
TFPI	52.58846783	114.7739713	4_EWS-FLI1_loop_and_bridge_target	1.111316465
SLC39A10	11.89414541	23.68255262	2_EWS-FLI1_loop_target	0.936775444
PGAP1	7.493605295	5.919421473	4_EWS-FLI1_loop_and_bridge_target	-0.295725647
SF3B1	132.9546729	134.5555544	4_EWS-FLI1_loop_and_bridge_target	0.01713932
HSPD1	278.4883788	198.706274	4_EWS-FLI1_loop_and_bridge_target	-0.48490864
HSPE1	37.05402914	38.3418871	4_EWS-FLI1_loop_and_bridge_target	0.048016947
SATB2	3.479961356	3.979801846	4_EWS-FLI1_loop_and_bridge_target	0.152602049
ERBB4	5.726617902	2.928930927	4_EWS-FLI1_loop_and_bridge_target	-0.775744505
IKZF2	16.54020496	6.20606454	4_EWS-FLI1_loop_and_bridge_target	-1.283382128
ATIC	50.29521274	15.04421298	3_EWS-FLI1_bridge_target	-1.676771166
FN1	392.3289016	1356.62967	4_EWS-FLI1_loop_and_bridge_target	1.787281896
PECR	18.918234	4.831718661	3_EWS-FLI1_bridge_target	-1.772096714
TMEM169	2.052385698	0.622248049	3_EWS-FLI1_bridge_target	-0.91194284
XRCC5	216.7380293	146.5577815	3_EWS-FLI1_bridge_target	-0.561313402
ARPC2	165.5184552	180.9214974	3_EWS-FLI1_bridge_target	0.127633955
EPHA4	11.11128938	25.23171181	4_EWS-FLI1_loop_and_bridge_target	1.114959494
PAX3	12.59512423	6.980362574	4_EWS-FLI1_loop_and_bridge_target	-0.768563135
CCDC140	0.427845394	0.350157307	4_EWS-FLI1_loop_and_bridge_target	-0.080712268
FARSB	61.50030696	33.52523301	3_EWS-FLI1_bridge_target	-0.856212124
ACSL3	58.33650779	134.346806	3_EWS-FLI1_bridge_target	1.189668916
KCNE4	40.93980414	22.50086829	4_EWS-FLI1_loop_and_bridge_target	-0.835606063
SCG2	26.18487994	31.98489867	4_EWS-FLI1_loop_and_bridge_target	0.279001217
WDFY1	73.04967308	47.55001243	4_EWS-FLI1_loop_and_bridge_target	-0.609021701
MRPL44	38.68882877	13.21887103	4_EWS-FLI1_loop_and_bridge_target	-1.480926068

SERPINE2	177.8155396	606.741465	4_EWS-FLI1_loop_and_bridge_target	1.764985611
DOCK10	2.670585338	5.71587176	2_EWS-FLI1_loop_target	0.871564538
IRS1	10.28281697	8.121546819	4_EWS-FLI1_loop_and_bridge_target	-0.306776909
RHBDD1	5.78908153	8.647469105	4_EWS-FLI1_loop_and_bridge_target	0.506934108
TM4SF20	0.439176468	0.165975174	4_EWS-FLI1_loop_and_bridge_target	-0.303706431
SPHKAP	8.201783197	0.693625511	2_EWS-FLI1_loop_target	-2.44179856
ATG16L1	34.91756511	31.15234899	4_EWS-FLI1_loop_and_bridge_target	-0.159765409
DGKD	35.36971886	63.61160062	4_EWS-FLI1_loop_and_bridge_target	0.829055441
USP40	10.55051434	12.04450621	4_EWS-FLI1_loop_and_bridge_target	0.175485236
HDLBP	162.6835688	215.7677412	3_EWS-FLI1_bridge_target	0.405240569
2-Sep	94.33790961	169.8831435	3_EWS-FLI1_bridge_target	0.841888194
FARP2	10.26182089	8.547351229	3_EWS-FLI1_bridge_target	-0.238267672
NEU4	2.181948934	2.620494687	2_EWS-FLI1_loop_target	0.186276151
EGOT	0.819947157	1.912524723	4_EWS-FLI1_loop_and_bridge_target	0.678373734
BHLHE40-AS1	0.023459888	0.153216367	4_EWS-FLI1_loop_and_bridge_target	0.172208657
BHLHE40	2.328625087	23.57315543	4_EWS-FLI1_loop_and_bridge_target	2.884084836
EDEM1	16.51458992	38.89345343	4_EWS-FLI1_loop_and_bridge_target	1.187594808
LMCD1-AS1	1.455256084	0.969823208	4_EWS-FLI1_loop_and_bridge_target	-0.317807353
LMCD1	3.677681666	2.907731108	4_EWS-FLI1_loop_and_bridge_target	-0.259462486
THUMPD3	23.51897397	14.98952345	2_EWS-FLI1_loop_target	-0.616771668
THUMPD3-AS1	9.596348989	4.713693415	3_EWS-FLI1_bridge_target	-0.891071733
SETD5	17.35348707	20.65894131	3_EWS-FLI1_bridge_target	0.238908531
MTMR14	21.9095546	17.16660274	3_EWS-FLI1_bridge_target	-0.334660758
VGLL4	7.402513239	5.278318411	3_EWS-FLI1_bridge_target	-0.420442712
RBMS3-AS3	0.101442689	0.229854398	4_EWS-FLI1_loop_and_bridge_target	0.159093096
RBMS3	3.311305567	6.289836569	4_EWS-FLI1_loop_and_bridge_target	0.757761653
TGFBR2	33.12255853	94.37464908	4_EWS-FLI1_loop_and_bridge_target	1.482880019
GADL1	24.3785991	2.633587235	1_EWS-FLI1_in_promoter	-2.804145987
CLASP2	26.56084505	11.96346525	4_EWS-FLI1_loop_and_bridge_target	-1.088168709
PDCD6IP	36.38557052	28.20262315	4_EWS-FLI1_loop_and_bridge_target	-0.356383583
TCAIM	7.969622852	5.813247483	3_EWS-FLI1_bridge_target	-0.396704713
ZNF197-AS1	0.040939952	0.057295403	4_EWS-FLI1_loop_and_bridge_target	0.022491667
ZNF197	4.332241034	5.57299986	4_EWS-FLI1_loop_and_bridge_target	0.301809958
ZNF35	8.285224785	7.177635253	4_EWS-FLI1_loop_and_bridge_target	-0.183253121
KIAA1143	6.118690976	5.731631693	2_EWS-FLI1_loop_target	-0.08065573
KIF15	14.76445169	1.340623979	2_EWS-FLI1_loop_target	-2.751709903
ZDHHC3	10.74930148	4.557311982	4_EWS-FLI1_loop_and_bridge_target	-1.080115849
EXOSC7	33.15726739	17.51874788	4_EWS-FLI1_loop_and_bridge_target	-0.883206007
LARS2-AS1	0.123813545	0.121935577	4_EWS-FLI1_loop_and_bridge_target	-0.002412857
ELP6	28.70087923	8.423217019	4_EWS-FLI1_loop_and_bridge_target	-1.656214065
CSPG5	26.2884839	5.632189704	4_EWS-FLI1_loop_and_bridge_target	-2.040735065
SMARCC1	76.97309813	20.38240079	4_EWS-FLI1_loop_and_bridge_target	-1.866552613
DUSP7	4.323248836	4.556186662	2_EWS-FLI1_loop_target	0.061788061
WNT5A	12.24431701	4.888895762	2_EWS-FLI1_loop_target	-1.169304406
ARF4	63.51478321	114.5386688	4_EWS-FLI1_loop_and_bridge_target	0.840674089
ARF4-AS1	0.862620513	1.065848116	4_EWS-FLI1_loop_and_bridge_target	0.149400417
ABHD6	77.6486455	32.73717415	1_EWS-FLI1_in_promoter	-1.221082782
RPP14	11.57959471	7.63570853	4_EWS-FLI1_loop_and_bridge_target	-0.542698986
PXK	6.747991731	9.984135375	4_EWS-FLI1_loop_and_bridge_target	0.503526992
MAGI1	7.048256221	3.001840387	4_EWS-FLI1_loop_and_bridge_target	-1.008012608
SLC25A26	4.10604862	3.071654043	2_EWS-FLI1_loop_target	-0.32659229
LRIG1	19.81744732	28.75891524	2_EWS-FLI1_loop_target	0.515528766
FOXP1	10.58142079	5.674406173	4_EWS-FLI1_loop_and_bridge_target	-0.795100862
GXYLT2	6.417695487	15.85161676	4_EWS-FLI1_loop_and_bridge_target	1.183844063
ROBO2	7.21866995	13.33685313	4_EWS-FLI1_loop_and_bridge_target	0.802751552
ROBO1	30.12022659	34.12736377	3_EWS-FLI1_bridge_target	0.174742744

VGLL3	7.078032617	5.12703789	4_EWS-FLI1_loop_and_bridge_target	-0.398814198
CHMP2B	28.36866075	31.47085166	4_EWS-FLI1_loop_and_bridge_target	0.144867746
POU1F1	29.48370103	1.385180497	4_EWS-FLI1_loop_and_bridge_target	-3.675867721
HTR1F	7.35296297	2.692418358	4_EWS-FLI1_loop_and_bridge_target	-1.17772202
CGGBP1	51.93498841	23.88090037	4_EWS-FLI1_loop_and_bridge_target	-1.089182922
ZNF654	14.24752462	7.486572318	4_EWS-FLI1_loop_and_bridge_target	-0.845321166
C3orf38	23.63873626	20.01455498	4_EWS-FLI1_loop_and_bridge_target	-0.229539356
EPHA3	22.36993899	11.37063924	4_EWS-FLI1_loop_and_bridge_target	-0.917733916
CMSS1	60.17080946	45.38591297	4_EWS-FLI1_loop_and_bridge_target	-0.39915663
TBC1D23	19.28015222	28.04041334	4_EWS-FLI1_loop_and_bridge_target	0.517993507
TOMM70	36.92226745	49.96707527	4_EWS-FLI1_loop_and_bridge_target	0.426520339
LNP1	1.921260072	2.023309056	4_EWS-FLI1_loop_and_bridge_target	0.049537662
TMEM45A	2.437200851	18.31967451	4_EWS-FLI1_loop_and_bridge_target	2.490764726
ADGRG7	2.02505525	0.145057547	1_EWS-FLI1_in_promoter	-1.401541386
TFG	94.81347275	134.6133985	4_EWS-FLI1_loop_and_bridge_target	0.501199284
CEP97	11.72003922	4.486819148	3_EWS-FLI1_bridge_target	-1.21306119
NFKBIZ	12.92599251	35.33499542	4_EWS-FLI1_loop_and_bridge_target	1.383579572
LOC152225	3.040525026	8.864296253	4_EWS-FLI1_loop_and_bridge_target	1.287673359
ALCAM	20.74505372	32.4912762	4_EWS-FLI1_loop_and_bridge_target	0.623098079
CBLB	3.014384215	5.101421138	4_EWS-FLI1_loop_and_bridge_target	0.60396661
ZBTB20	1.097266494	1.094772164	4_EWS-FLI1_loop_and_bridge_target	-0.001716854
KALRN	4.753074688	1.121534833	4_EWS-FLI1_loop_and_bridge_target	-1.439224834
UMPS	22.72967468	11.44754491	4_EWS-FLI1_loop_and_bridge_target	-0.930831101
ITGB5	52.58344302	79.83844025	4_EWS-FLI1_loop_and_bridge_target	0.593254201
HEG1	8.61746872	22.7063988	4_EWS-FLI1_loop_and_bridge_target	1.301547384
OSBPL11	8.523860494	10.46428427	4_EWS-FLI1_loop_and_bridge_target	0.267527896
FAM86JP	1.555828512	1.218399886	4_EWS-FLI1_loop_and_bridge_target	-0.204271592
SLC41A3	15.88025583	16.58432334	2_EWS-FLI1_loop_target	0.058953051
KLF15	10.85881567	4.321102035	4_EWS-FLI1_loop_and_bridge_target	-1.156162963
ZXDC	18.07752621	12.17412152	4_EWS-FLI1_loop_and_bridge_target	-0.534167346
ASTE1	7.406723297	3.728398421	2_EWS-FLI1_loop_target	-0.830191984
NEK11	3.374027189	4.362407019	2_EWS-FLI1_loop_target	0.293918539
MRPL3	86.22595679	99.6587595	2_EWS-FLI1_loop_target	0.206643301
TSC22D2	8.047682254	9.848630351	3_EWS-FLI1_bridge_target	0.261892743
LOC101928105	0.979588817	0.7277793	4_EWS-FLI1_loop_and_bridge_target	-0.196281852
IGSF10	9.870463164	0.660793502	4_EWS-FLI1_loop_and_bridge_target	-2.710468802
AADACP1	2.651473313	0.29538173	4_EWS-FLI1_loop_and_bridge_target	-1.495101385
MBNL1-AS1	0.404272803	0.584549115	4_EWS-FLI1_loop_and_bridge_target	0.174249149
MBNL1	44.88382135	34.76941936	4_EWS-FLI1_loop_and_bridge_target	-0.359258849
P2RY1	22.47191071	3.243079443	4_EWS-FLI1_loop_and_bridge_target	-2.467751692
PLCH1	1.155087933	0.240297059	3_EWS-FLI1_bridge_target	-0.797061039
SSR3	163.4159805	147.3713529	4_EWS-FLI1_loop_and_bridge_target	-0.148137962
TIPARP-AS1	0.521545692	0.243300808	4_EWS-FLI1_loop_and_bridge_target	-0.29136227
TIPARP	83.05926449	48.6991656	4_EWS-FLI1_loop_and_bridge_target	-0.758185203
LINC00886	2.86622606	1.176641878	4_EWS-FLI1_loop_and_bridge_target	-0.828821934
LEKR1	0.332381678	0.497349078	4_EWS-FLI1_loop_and_bridge_target	0.168403176
LOC105374177	0.247048136	0.127170023	4_EWS-FLI1_loop_and_bridge_target	-0.145812005
LINC00881	0.048367112	0.015042156	4_EWS-FLI1_loop_and_bridge_target	-0.046604355
CCNL1	29.82088767	34.15976553	4_EWS-FLI1_loop_and_bridge_target	0.190017036
TERC	26.66493532	11.87196316	4_EWS-FLI1_loop_and_bridge_target	-1.103826449
ACTRT3	2.444869365	1.657333169	4_EWS-FLI1_loop_and_bridge_target	-0.374470154
MYNN	10.23882883	11.65448132	4_EWS-FLI1_loop_and_bridge_target	0.171156672
LOC100128164	0.029099361	0.015271692	4_EWS-FLI1_loop_and_bridge_target	-0.019516431
SEC62	32.00579196	40.64100759	4_EWS-FLI1_loop_and_bridge_target	0.335285763
PHC3	6.424143912	8.145119327	4_EWS-FLI1_loop_and_bridge_target	0.300777318
PRKCI	40.17757146	22.74912948	4_EWS-FLI1_loop_and_bridge_target	-0.793984113

TBL1XR1	38.1829069	39.11406759	4_EWS-FLI1_loop_and_bridge_target	0.033883832
LINC00578	0.135668034	0.058420681	4_EWS-FLI1_loop_and_bridge_target	-0.101628027
SOX2	31.48092465	7.398572991	4_EWS-FLI1_loop_and_bridge_target	-1.951376579
LOC344887	1.371838075	0.856944537	4_EWS-FLI1_loop_and_bridge_target	-0.353074795
ETV5	24.3957054	9.777991711	4_EWS-FLI1_loop_and_bridge_target	-1.236496165
TBCCD1	14.05963298	10.92443252	4_EWS-FLI1_loop_and_bridge_target	-0.336766
DNAJB11	110.0239672	267.8077589	4_EWS-FLI1_loop_and_bridge_target	1.275703631
AHSG	2.059256178	0.098333432	4_EWS-FLI1_loop_and_bridge_target	-1.477864827
BCL6	9.406590034	16.96898604	4_EWS-FLI1_loop_and_bridge_target	0.788011589
LPP-AS2	1.278375927	0.660331338	4_EWS-FLI1_loop_and_bridge_target	-0.456534632
LPP	11.51573814	18.16422352	4_EWS-FLI1_loop_and_bridge_target	0.614672166
TPRG1-AS1	0.8050545	0.426769917	4_EWS-FLI1_loop_and_bridge_target	-0.339289694
TP63	1.05752026	1.074206833	4_EWS-FLI1_loop_and_bridge_target	0.011653126
CLDN1	56.91470513	86.86274619	4_EWS-FLI1_loop_and_bridge_target	0.601321883
CLDN16	0.276641752	0.178129826	4_EWS-FLI1_loop_and_bridge_target	-0.115855209
IL1RAP	78.30393672	65.23394935	1_EWS-FLI1_in_promoter	-0.2598216
GMNC	1.784124487	0.059097742	2_EWS-FLI1_loop_target	-1.394387982
UTS2B	6.249293305	0.321176264	4_EWS-FLI1_loop_and_bridge_target	-2.456017405
CCDC50	19.69280101	10.02896621	4_EWS-FLI1_loop_and_bridge_target	-0.907831377
LOC647323	0.276376366	0.007367396	4_EWS-FLI1_loop_and_bridge_target	-0.341463856
LOC285389	0.033533513	0.00938602	4_EWS-FLI1_loop_and_bridge_target	-0.034107159
HES1	73.96795764	41.07820238	4_EWS-FLI1_loop_and_bridge_target	-0.833201026
LOC100505920	1.091218554	0.110397018	3_EWS-FLI1_bridge_target	-0.913268248
LINC00887	0.402096097	0.019745014	4_EWS-FLI1_loop_and_bridge_target	-0.459376779
TMEM44	4.954359704	8.546033725	3_EWS-FLI1_bridge_target	0.680955056
LSG1	46.1592633	43.45960557	4_EWS-FLI1_loop_and_bridge_target	-0.085046032
FAM43A	10.36848878	16.12046083	3_EWS-FLI1_bridge_target	0.590681047
XXYLT1	29.45341211	25.82458249	3_EWS-FLI1_bridge_target	-0.183048165
XXYLT1-AS2	0.57012496	0.699902665	4_EWS-FLI1_loop_and_bridge_target	0.114572759
ACAP2	26.32376126	38.39664065	3_EWS-FLI1_bridge_target	0.527916525
PPP1R2	29.13615388	24.7339566	3_EWS-FLI1_bridge_target	-0.227822016
FAM86EP	1.614168015	1.526259332	4_EWS-FLI1_loop_and_bridge_target	-0.049349122
STK32B	11.60081614	11.3100017	4_EWS-FLI1_loop_and_bridge_target	-0.033686217
AFAP1	19.82345686	30.30020057	4_EWS-FLI1_loop_and_bridge_target	0.587962314
LOC389199	0.098401197	0.146893152	4_EWS-FLI1_loop_and_bridge_target	0.062325889
ABLIM2	1.80999317	0.948588552	1_EWS-FLI1_in_promoter	-0.528137129
BOD1L1	19.07838478	18.8091372	4_EWS-FLI1_loop_and_bridge_target	-0.019477171
LINC01182	0.131649142	0.037382593	4_EWS-FLI1_loop_and_bridge_target	-0.125478666
LINC01085	0.623498553	2.027036648	4_EWS-FLI1_loop_and_bridge_target	0.898800043
CPEB2-AS1	0.289480626	0.386878712	4_EWS-FLI1_loop_and_bridge_target	0.105051526
CPEB2	14.68228732	23.29912657	4_EWS-FLI1_loop_and_bridge_target	0.63176846
FAM200B	10.05100571	14.54392949	3_EWS-FLI1_bridge_target	0.492173591
TAPT1	12.12738562	9.898863121	4_EWS-FLI1_loop_and_bridge_target	-0.268401973
TAPT1-AS1	0.921285555	0.761456727	4_EWS-FLI1_loop_and_bridge_target	-0.125302925
LDB2	24.10266784	8.736897373	4_EWS-FLI1_loop_and_bridge_target	-1.366306657
SMIM20	30.83812003	23.33906193	3_EWS-FLI1_bridge_target	-0.387481585
RBPJ	17.16691173	16.57201836	4_EWS-FLI1_loop_and_bridge_target	-0.048033278
CCKAR	0.735446569	0.099034979	3_EWS-FLI1_bridge_target	-0.659069645
LOC105374546	1.025516601	0.771617724	3_EWS-FLI1_bridge_target	-0.193222574
STIM2	15.07678908	10.69626314	3_EWS-FLI1_bridge_target	-0.458931621
PCDH7	44.0675929	31.8865597	4_EWS-FLI1_loop_and_bridge_target	-0.454592303
KLF3-AS1	0.619329156	0.9647307	4_EWS-FLI1_loop_and_bridge_target	0.278935312
KLF3	14.51978974	14.69970104	4_EWS-FLI1_loop_and_bridge_target	0.016628074
RFC1	46.91643744	28.79015823	3_EWS-FLI1_bridge_target	-0.685684862
RPL9	150.7557548	171.7585832	4_EWS-FLI1_loop_and_bridge_target	0.187006164
LIAS	8.102388748	4.952579886	4_EWS-FLI1_loop_and_bridge_target	-0.612730125

SMIM14	9.247627805	22.55514233	4_EWS-FLI1_loop_and_bridge_target	1.200752067
UBE2K	23.07081099	16.21243159	4_EWS-FLI1_loop_and_bridge_target	-0.483833829
N4BP2	15.07671334	13.41414351	4_EWS-FLI1_loop_and_bridge_target	-0.157487383
RHOH	30.56937173	5.762297143	1_EWS-FLI1_in_promoter	-2.222940233
LOC101060498	0.330736106	0.15428823	4_EWS-FLI1_loop_and_bridge_target	-0.205220988
CHRNA9	0.728410971	0.517546654	4_EWS-FLI1_loop_and_bridge_target	-0.187705423
UCHL1-AS1	0.193876953	0.180887006	4_EWS-FLI1_loop_and_bridge_target	-0.015783227
UCHL1	317.1066474	319.4769227	4_EWS-FLI1_loop_and_bridge_target	0.010709953
TMEM33	14.87798558	27.14555751	3_EWS-FLI1_bridge_target	0.825879334
EPHA5	2.471361941	0.750942396	4_EWS-FLI1_loop_and_bridge_target	-0.987370174
EPHA5-AS1	1.083500013	0.192071844	4_EWS-FLI1_loop_and_bridge_target	-0.805537922
SULT1B1	0.623513285	0.136344675	2_EWS-FLI1_loop_target	-0.514718692
PTPN13	93.4089833	35.94057712	3_EWS-FLI1_bridge_target	-1.353717737
C4orf36	2.528087145	0.366909326	3_EWS-FLI1_bridge_target	-1.367968651
LOC100506746	2.694806585	2.398021263	3_EWS-FLI1_bridge_target	-0.120803964
AFF1	11.22985798	9.072457693	3_EWS-FLI1_bridge_target	-0.279991905
SMARCAD1	15.27829363	16.56657632	2_EWS-FLI1_loop_target	0.109883564
PDLIM5	29.59537559	19.37434265	4_EWS-FLI1_loop_and_bridge_target	-0.586560096
EMCN	2.931531806	4.568351551	4_EWS-FLI1_loop_and_bridge_target	0.502158771
PPP3CA	61.73760133	38.3617309	4_EWS-FLI1_loop_and_bridge_target	-0.672536708
FLJ20021	5.355209061	3.030852144	4_EWS-FLI1_loop_and_bridge_target	-0.65685472
TBCK	6.236433469	5.587384172	4_EWS-FLI1_loop_and_bridge_target	-0.13557314
AIMP1	34.58511102	32.80439495	4_EWS-FLI1_loop_and_bridge_target	-0.074062912
DKK2	57.07726133	4.400130444	4_EWS-FLI1_loop_and_bridge_target	-3.426907262
PAPSS1	129.0831704	61.79463203	4_EWS-FLI1_loop_and_bridge_target	-1.050721184
RPL34-AS1	0.060840263	0.059917456	3_EWS-FLI1_bridge_target	-0.001255523
RPL34	129.9227964	166.466494	3_EWS-FLI1_bridge_target	0.355156154
SEC24B-AS1	2.038848128	0.944905165	3_EWS-FLI1_bridge_target	-0.643824765
SEC24B	20.68013713	18.23970354	3_EWS-FLI1_bridge_target	-0.172287313
ELOVL6	22.93397713	30.03350013	3_EWS-FLI1_bridge_target	0.37476627
ENPEP	24.19329066	12.88451446	4_EWS-FLI1_loop_and_bridge_target	-0.859562847
NDST4	20.71899793	6.025740869	4_EWS-FLI1_loop_and_bridge_target	-1.62823527
SNHG8	87.58018124	73.58136639	2_EWS-FLI1_loop_target	-0.24816872
SNORA24	68.82615398	62.48901958	2_EWS-FLI1_loop_target	-0.137260412
QRFPR	16.00212259	2.271179673	3_EWS-FLI1_bridge_target	-2.377831959
ANXA5	297.8363866	469.9288309	4_EWS-FLI1_loop_and_bridge_target	0.656153226
BBS7	13.99174022	16.7943949	3_EWS-FLI1_bridge_target	0.247255016
FAT4	10.91299317	15.37718639	4_EWS-FLI1_loop_and_bridge_target	0.459151583
SLC7A11	23.67356563	87.51721331	4_EWS-FLI1_loop_and_bridge_target	1.842991816
LOC105377448	0.271336067	0.042192714	4_EWS-FLI1_loop_and_bridge_target	-0.286723371
NOCT	20.18978305	50.69990481	4_EWS-FLI1_loop_and_bridge_target	1.286792807
ELF2	27.10327194	21.06274496	4_EWS-FLI1_loop_and_bridge_target	-0.34912581
NDUFC1	44.30565033	26.58088702	4_EWS-FLI1_loop_and_bridge_target	-0.716022133
NAA15	61.44027596	34.29825499	4_EWS-FLI1_loop_and_bridge_target	-0.822880049
RAB33B	8.156777457	9.019117997	4_EWS-FLI1_loop_and_bridge_target	0.129843645
SETD7	35.76751281	45.8483273	4_EWS-FLI1_loop_and_bridge_target	0.349565951
MGST2	9.311261227	5.396176091	3_EWS-FLI1_bridge_target	-0.688939245
MAML3	0.606333517	1.139810448	3_EWS-FLI1_bridge_target	0.413711538
TBC1D9	19.17800948	15.64179116	3_EWS-FLI1_bridge_target	-0.277973143
ANAPC10	12.02092742	8.14846029	3_EWS-FLI1_bridge_target	-0.50923135
ABCE1	99.58978226	69.46091709	3_EWS-FLI1_bridge_target	-0.513588609
SMAD1	17.40877234	10.84860357	4_EWS-FLI1_loop_and_bridge_target	-0.635676379
MMAA	2.407645324	2.08616453	4_EWS-FLI1_loop_and_bridge_target	-0.142960207
SLC10A7	9.139706976	11.21387673	4_EWS-FLI1_loop_and_bridge_target	0.268505229
EDNRA	23.55118603	18.74777829	4_EWS-FLI1_loop_and_bridge_target	-0.314102368
CTSO	1.969661167	9.6914923	4_EWS-FLI1_loop_and_bridge_target	1.848092999

PDGFC	28.06113576	16.80268117	4_EWS-FLI1_loop_and_bridge_target	-0.706996553
TMEM144	1.329857481	0.713613838	4_EWS-FLI1_loop_and_bridge_target	-0.443199672
C4orf46	28.85601667	4.986853345	4_EWS-FLI1_loop_and_bridge_target	-2.318151861
ETFDH	22.68076657	22.01888709	4_EWS-FLI1_loop_and_bridge_target	-0.040897699
RAPGEF2	24.48648864	37.78335251	3_EWS-FLI1_bridge_target	0.605704896
NAF1	4.984231222	2.525762015	4_EWS-FLI1_loop_and_bridge_target	-0.763230822
NPY1R	65.08323043	4.312044002	4_EWS-FLI1_loop_and_bridge_target	-3.636945211
NPY5R	5.060632997	0.315915662	3_EWS-FLI1_bridge_target	-2.203401454
TMA16	42.88609423	24.77064274	3_EWS-FLI1_bridge_target	-0.768035359
1-Mar	8.7843691	6.660001754	4_EWS-FLI1_loop_and_bridge_target	-0.353134106
APELA	19.30428831	0.235970235	4_EWS-FLI1_loop_and_bridge_target	-4.038068555
LOC101928131	0.082011544	0.07651669	3_EWS-FLI1_bridge_target	-0.007345205
LOC101930370	1.295717923	0.78794606	4_EWS-FLI1_loop_and_bridge_target	-0.360642175
GALNT7	59.22188433	47.67073286	4_EWS-FLI1_loop_and_bridge_target	-0.307233352
HMGB2	191.2842123	28.58338123	4_EWS-FLI1_loop_and_bridge_target	-2.700381454
SAP30	40.88024054	13.8724621	4_EWS-FLI1_loop_and_bridge_target	-1.493626227
HAND2	8.417491056	1.904338204	4_EWS-FLI1_loop_and_bridge_target	-1.697133296
HAND2-AS1	0.824465828	0.092044113	4_EWS-FLI1_loop_and_bridge_target	-0.740442994
DCTD	43.17964039	57.5688793	3_EWS-FLI1_bridge_target	0.406752614
WWC2-AS2	1.270012183	0.2402603	4_EWS-FLI1_loop_and_bridge_target	-0.872057103
WWC2	26.50027358	27.61556232	4_EWS-FLI1_loop_and_bridge_target	0.057353987
LOC389247	0.506192893	0.228521357	4_EWS-FLI1_loop_and_bridge_target	-0.293983606
ING2	6.42831495	3.509615545	4_EWS-FLI1_loop_and_bridge_target	-0.720030539
FLJ38576	0.114095768	0.106451239	3_EWS-FLI1_bridge_target	-0.009933379
CYP4V2	6.314043348	22.63316193	3_EWS-FLI1_bridge_target	1.692071581
F11-AS1	4.517905569	1.242679367	4_EWS-FLI1_loop_and_bridge_target	-1.298897393
ZNF622	27.66874173	37.30539114	4_EWS-FLI1_loop_and_bridge_target	0.418068867
MYO10	54.69978435	40.10044248	1_EWS-FLI1_in_promoter	-0.438517816
LOC285696	0.561492187	0.107644861	4_EWS-FLI1_loop_and_bridge_target	-0.49542996
BASP1	90.41296491	44.71426045	4_EWS-FLI1_loop_and_bridge_target	-0.999754514
LOC101929544	0.247500621	0.072921422	4_EWS-FLI1_loop_and_bridge_target	-0.217496113
LOC102723526	1.194674941	0.120863553	4_EWS-FLI1_loop_and_bridge_target	-0.969396611
CDH12	32.91270806	1.993995075	4_EWS-FLI1_loop_and_bridge_target	-3.50168224
IL7R	6.049260657	40.9458274	4_EWS-FLI1_loop_and_bridge_target	2.572983447
UGT3A2	190.6609714	26.80495148	1_EWS-FLI1_in_promoter	-2.785142861
LMBRD2	8.415190128	7.803579569	4_EWS-FLI1_loop_and_bridge_target	-0.09689998
SKP2	39.08819062	20.57761772	4_EWS-FLI1_loop_and_bridge_target	-0.89364171
NADK2	19.65711621	17.22932938	4_EWS-FLI1_loop_and_bridge_target	-0.180377376
NIPBL-AS1	5.159454806	3.84565941	2_EWS-FLI1_loop_target	-0.346109654
NIPBL	16.87729142	12.76876039	2_EWS-FLI1_loop_target	-0.376729492
NUP155	52.5156182	20.28007411	4_EWS-FLI1_loop_and_bridge_target	-1.330456819
GDNF	3.025776797	0.668766119	4_EWS-FLI1_loop_and_bridge_target	-1.270485414
GDNF-AS1	0.44297735	0.133487369	4_EWS-FLI1_loop_and_bridge_target	-0.34828034
EGFLAM	10.81545464	8.355224332	4_EWS-FLI1_loop_and_bridge_target	-0.336830989
LIFR	15.57966679	27.22334084	4_EWS-FLI1_loop_and_bridge_target	0.76747376
LIFR-AS1	0.648074861	0.386875568	4_EWS-FLI1_loop_and_bridge_target	-0.248943422
OSMR-AS1	0.524087472	2.589746389	4_EWS-FLI1_loop_and_bridge_target	1.235936217
LINC01265	3.331667733	2.424287854	1_EWS-FLI1_in_promoter	-0.3391186
OSMR	18.55867804	85.518792	4_EWS-FLI1_loop_and_bridge_target	2.145204659
RICTOR	9.583500694	8.326242465	4_EWS-FLI1_loop_and_bridge_target	-0.182449063
LOC101926940	11.13474435	9.077702505	4_EWS-FLI1_loop_and_bridge_target	-0.267976941
RPL37	140.4107115	167.0163909	4_EWS-FLI1_loop_and_bridge_target	0.248710578
SNORD72	4.463478303	5.354255384	4_EWS-FLI1_loop_and_bridge_target	0.217903345
C6	2.015629584	0.017995985	4_EWS-FLI1_loop_and_bridge_target	-1.566727359
ARL15	4.878482779	1.474131276	4_EWS-FLI1_loop_and_bridge_target	-1.248521797
HSPB3	0.211462164	0.925764111	4_EWS-FLI1_loop_and_bridge_target	0.668681648

SNX18	43.25102618	37.09694493	4_EWS-FLI1_loop_and_bridge_target	-0.216035602
LOC102467080	0.990002484	1.677192698	4_EWS-FLI1_loop_and_bridge_target	0.427950753
GPX8	25.98028516	61.72816309	4_EWS-FLI1_loop_and_bridge_target	1.217207719
DHX29	30.0929472	31.9523639	2_EWS-FLI1_loop_target	0.083794592
SKIV2L2	58.43413249	43.46971585	2_EWS-FLI1_loop_target	-0.41846851
SMIM15	36.78012027	32.71015688	4_EWS-FLI1_loop_and_bridge_target	-0.164443953
CTC-436P18.1	0.345838513	0.242000243	4_EWS-FLI1_loop_and_bridge_target	-0.115839855
ZSWIM6	24.9609952	9.911323795	4_EWS-FLI1_loop_and_bridge_target	-1.250519545
DIMT1	60.70887454	36.88716297	4_EWS-FLI1_loop_and_bridge_target	-0.703768869
LRRC70	1.33255085	0.27510625	3_EWS-FLI1_bridge_target	-0.871291066
PIK3R1	13.83813942	9.407163225	4_EWS-FLI1_loop_and_bridge_target	-0.511733328
LINC01336	3.15237633	1.069514184	3_EWS-FLI1_bridge_target	-1.004645064
HMGCR	60.24874654	155.0212609	3_EWS-FLI1_bridge_target	1.348990413
POC5	14.15270038	7.624285518	4_EWS-FLI1_loop_and_bridge_target	-0.813098074
IQGAP2	21.04836441	8.663229308	4_EWS-FLI1_loop_and_bridge_target	-1.190094336
LOC101929109	0.265683232	0.240370612	4_EWS-FLI1_loop_and_bridge_target	-0.029145131
F2RL2	15.59790693	23.66483375	4_EWS-FLI1_loop_and_bridge_target	0.57145424
F2R	74.89598187	126.4991505	1_EWS-FLI1_in_promoter	0.748392222
COX7C	446.4418312	285.4141158	2_EWS-FLI1_loop_target	-0.643597543
LUCAT1	6.066741004	7.2168358	4_EWS-FLI1_loop_and_bridge_target	0.217537902
ARRDC3	53.20867018	54.42756654	4_EWS-FLI1_loop_and_bridge_target	0.032080054
ARRDC3-AS1	2.308959314	2.477633053	4_EWS-FLI1_loop_and_bridge_target	0.07172816
PAM	74.13619206	79.4354864	4_EWS-FLI1_loop_and_bridge_target	0.098324126
GIN1	3.819729852	3.811895915	3_EWS-FLI1_bridge_target	-0.002346849
PPIP5K2	17.30940726	27.04377265	3_EWS-FLI1_bridge_target	0.615095358
EFNA5	2.11550585	3.479178756	4_EWS-FLI1_loop_and_bridge_target	0.523767817
FER	5.069134921	4.853532603	4_EWS-FLI1_loop_and_bridge_target	-0.05218334
PJA2	61.28622997	85.12363115	4_EWS-FLI1_loop_and_bridge_target	0.467495895
MAN2A1	63.36601111	28.30080135	4_EWS-FLI1_loop_and_bridge_target	-1.135358943
ALDH7A1	20.43392562	4.789710718	4_EWS-FLI1_loop_and_bridge_target	-1.888332932
PHAX	27.22534378	27.22469505	4_EWS-FLI1_loop_and_bridge_target	-3.31594E-05
LOC102723557	0.237196409	0.03905365	4_EWS-FLI1_loop_and_bridge_target	-0.251804403
LMNB1	54.98612537	10.34311774	4_EWS-FLI1_loop_and_bridge_target	-2.303252108
HINT1	140.8269049	114.6436568	3_EWS-FLI1_bridge_target	-0.294445107
LYRM7	13.90416004	8.717580667	3_EWS-FLI1_bridge_target	-0.617045987
CDC42SE2	20.7267803	12.34746227	3_EWS-FLI1_bridge_target	-0.702908926
GDF9	0.499701361	0.257392776	3_EWS-FLI1_bridge_target	-0.254239863
UQCRQ	45.50776617	30.9582183	3_EWS-FLI1_bridge_target	-0.541284668
HSPA4	135.2248379	103.6228122	4_EWS-FLI1_loop_and_bridge_target	-0.38079232
C5orf15	39.50515149	93.4445233	4_EWS-FLI1_loop_and_bridge_target	1.221361736
LOC105379183	0.44203796	0.515526226	4_EWS-FLI1_loop_and_bridge_target	0.071709675
VDAC1	202.3944276	210.0448185	4_EWS-FLI1_loop_and_bridge_target	0.053269255
CDKL3	1.223886863	2.219508533	3_EWS-FLI1_bridge_target	0.533757078
UBE2B	16.33558498	18.76902988	3_EWS-FLI1_bridge_target	0.189505556
TGFBI	104.0091603	531.6503058	4_EWS-FLI1_loop_and_bridge_target	2.342673505
KIAA0141	6.535871819	4.823653499	3_EWS-FLI1_bridge_target	-0.371849906
SPRY4	8.489566398	11.29600636	4_EWS-FLI1_loop_and_bridge_target	0.373775742
SPRY4-IT1	1.443712659	2.175894786	4_EWS-FLI1_loop_and_bridge_target	0.378088461
LOC101926941	1.791001688	0.583844355	4_EWS-FLI1_loop_and_bridge_target	-0.817352429
FGF1	1.014168873	1.591153595	4_EWS-FLI1_loop_and_bridge_target	0.363409889
PRELID2	12.82555357	5.751374069	4_EWS-FLI1_loop_and_bridge_target	-1.034084186
SH3RF2	11.65897999	4.383189313	4_EWS-FLI1_loop_and_bridge_target	-1.233628096
LARS	104.2973247	99.589514	4_EWS-FLI1_loop_and_bridge_target	-0.065988863
RBM27	44.80833024	25.60873217	4_EWS-FLI1_loop_and_bridge_target	-0.783710204
JAKMIP2	23.90426845	12.56578214	4_EWS-FLI1_loop_and_bridge_target	-0.876420804
CSNK1A1	138.451097	139.3830489	4_EWS-FLI1_loop_and_bridge_target	0.009609459

LOC644762	0.584700645	0.051603728	1_EWS-FLI1_in_promoter	-0.591619175
SLC26A2	51.08856599	12.52752096	4_EWS-FLI1_loop_and_bridge_target	-1.945069244
TIGD6	4.344420429	1.87359033	4_EWS-FLI1_loop_and_bridge_target	-0.895179104
HMGXB3	37.74176279	27.88825994	4_EWS-FLI1_loop_and_bridge_target	-0.423406292
PDGFRB	17.22319219	19.73617724	4_EWS-FLI1_loop_and_bridge_target	0.186374253
RPS14	336.667302	451.773813	3_EWS-FLI1_bridge_target	0.423188037
NDST1	7.931767635	13.00984038	3_EWS-FLI1_bridge_target	0.649422894
CNOT8	17.25680958	11.63420443	3_EWS-FLI1_bridge_target	-0.531099852
MRPL22	8.154875941	4.683590695	4_EWS-FLI1_loop_and_bridge_target	-0.687737674
UBLCP1	9.191395836	9.530614377	3_EWS-FLI1_bridge_target	0.047237949
CCNG1	66.91083647	46.82683463	3_EWS-FLI1_bridge_target	-0.505821491
TENM2	10.4537214	6.722702902	4_EWS-FLI1_loop_and_bridge_target	-0.56863864
KCNIP1	0.973883379	0.869875407	2_EWS-FLI1_loop_target	-0.078094611
CAGE1	0.074468877	0.208438166	3_EWS-FLI1_bridge_target	0.16951996
RIOK1	42.08224529	25.10283168	3_EWS-FLI1_bridge_target	-0.722887118
BLOC1S5	0.027454887	0.021346152	4_EWS-FLI1_loop_and_bridge_target	-0.008603147
EEF1E1	17.9563453	23.0703659	4_EWS-FLI1_loop_and_bridge_target	0.344577227
SCARNA27	3.441230438	1.322038366	4_EWS-FLI1_loop_and_bridge_target	-0.935567619
SLC35B3	10.71153142	20.95899	4_EWS-FLI1_loop_and_bridge_target	0.906881961
LOC100506207	0.782598308	0.514433339	4_EWS-FLI1_loop_and_bridge_target	-0.235203565
TFAP2A	1.229690972	1.315198093	4_EWS-FLI1_loop_and_bridge_target	0.054291867
TFAP2A-AS1	0.33251153	0.656963851	4_EWS-FLI1_loop_and_bridge_target	0.314394112
LINC00518	0.431008981	0.074906746	4_EWS-FLI1_loop_and_bridge_target	-0.412821223
RANBP9	30.04465135	24.39294561	4_EWS-FLI1_loop_and_bridge_target	-0.28991697
MCUR1	25.32843577	25.65832166	4_EWS-FLI1_loop_and_bridge_target	0.017964145
RNF182	17.43139501	2.036851315	4_EWS-FLI1_loop_and_bridge_target	-2.601517087
JARID2	18.67821181	7.865137869	4_EWS-FLI1_loop_and_bridge_target	-1.150384154
JARID2-AS1	0.518005219	0.345213113	4_EWS-FLI1_loop_and_bridge_target	-0.174342003
LOC100506885	0.33714286	0.235915467	4_EWS-FLI1_loop_and_bridge_target	-0.113573541
ID4	29.71374949	23.67390065	4_EWS-FLI1_loop_and_bridge_target	-0.315898838
CDKAL1	23.70047133	12.07294651	4_EWS-FLI1_loop_and_bridge_target	-0.917954224
DCDC2	36.3851533	3.28315337	1_EWS-FLI1_in_promoter	-3.125720206
KAAG1	2.020884326	0.24106633	4_EWS-FLI1_loop_and_bridge_target	-1.283390718
MRS2	14.38310794	15.12600333	4_EWS-FLI1_loop_and_bridge_target	0.068041916
C6orf62	54.19937939	81.83481664	4_EWS-FLI1_loop_and_bridge_target	0.585585233
TRIM38	13.59104958	8.615678705	4_EWS-FLI1_loop_and_bridge_target	-0.601623069
HIST1H1A	182.6376617	6.642354097	4_EWS-FLI1_loop_and_bridge_target	-4.586701052
HIST1H3A	162.7408933	22.80727595	4_EWS-FLI1_loop_and_bridge_target	-2.781940211
HIST1H4A	92.45776485	11.32261891	4_EWS-FLI1_loop_and_bridge_target	-2.923005631
HIST1H4B	310.9610134	60.55824483	4_EWS-FLI1_loop_and_bridge_target	-2.34134174
HIST1H3B	343.7040405	49.55963317	4_EWS-FLI1_loop_and_bridge_target	-2.769300308
HIST1H2AB	104.2948719	10.00259217	4_EWS-FLI1_loop_and_bridge_target	-3.258519813
HIST1H2BB	172.3550621	15.48717334	4_EWS-FLI1_loop_and_bridge_target	-3.394313984
HIST1H3C	227.7443613	20.81562369	4_EWS-FLI1_loop_and_bridge_target	-3.390302556
HIST1H1C	470.9300625	115.3261747	4_EWS-FLI1_loop_and_bridge_target	-2.02039732
HFE	5.779507074	4.658668051	4_EWS-FLI1_loop_and_bridge_target	-0.260717873
HIST1H4C	432.7775483	90.0918299	4_EWS-FLI1_loop_and_bridge_target	-2.251561815
HIST1H2BC	312.7637569	111.241379	1_EWS-FLI1_in_promoter	-1.483074076
HIST1H2AC	181.6222255	95.73155841	1_EWS-FLI1_in_promoter	-0.916803808
HIST1H1E	322.8000505	91.77640863	4_EWS-FLI1_loop_and_bridge_target	-1.803273305
HIST1H2BD	207.318088	54.36479721	4_EWS-FLI1_loop_and_bridge_target	-1.911747253
HIST1H2BE	104.3383861	7.850172644	4_EWS-FLI1_loop_and_bridge_target	-3.573181852
HIST1H4D	362.7075635	55.92546495	4_EWS-FLI1_loop_and_bridge_target	-2.675632852
HIST1H3D	76.75216452	14.55964729	4_EWS-FLI1_loop_and_bridge_target	-2.321073482
HIST1H2AD	6.251370172	0.97986373	4_EWS-FLI1_loop_and_bridge_target	-1.872852487
HIST1H2BF	198.0835321	22.33015036	4_EWS-FLI1_loop_and_bridge_target	-3.093106378

HIST1H4E	389.2139549	123.5913002	4_EWS-FLI1_loop_and_bridge_target	-1.647062039
HIST1H2BG	400.2769217	81.46614798	4_EWS-FLI1_loop_and_bridge_target	-2.28272426
HIST1H2AE	234.7961434	51.79155621	4_EWS-FLI1_loop_and_bridge_target	-2.159161022
HIST1H3E	22.05515871	4.919012883	4_EWS-FLI1_loop_and_bridge_target	-1.961661096
HIST1H1D	356.1099053	50.793837	4_EWS-FLI1_loop_and_bridge_target	-2.785515805
HIST1H4F	308.2503067	60.59103013	4_EWS-FLI1_loop_and_bridge_target	-2.327982863
HIST1H3F	173.093196	27.03937268	4_EWS-FLI1_loop_and_bridge_target	-2.634333842
HIST1H2BH	282.9920261	45.38168877	4_EWS-FLI1_loop_and_bridge_target	-2.614223167
HIST1H3G	245.1431308	27.97512449	4_EWS-FLI1_loop_and_bridge_target	-3.086610713
HIST1H2BI	283.8007617	53.44725817	4_EWS-FLI1_loop_and_bridge_target	-2.387021699
HIST1H4H	344.0185836	76.32120419	4_EWS-FLI1_loop_and_bridge_target	-2.15773806
BTN3A2	12.33492444	18.41066778	3_EWS-FLI1_bridge_target	0.541640102
BTN2A2	7.573410839	5.789628049	4_EWS-FLI1_loop_and_bridge_target	-0.336536737
BTN2A3P	1.997920787	2.285747365	3_EWS-FLI1_bridge_target	0.132259294
HCG11	8.867897174	7.5697696	3_EWS-FLI1_bridge_target	-0.203486265
HMGN4	69.49236545	67.58115027	4_EWS-FLI1_loop_and_bridge_target	-0.039654915
LOC105374988	0.106125896	0.024753839	4_EWS-FLI1_loop_and_bridge_target	-0.110238203
ABT1	11.0734611	13.90681543	4_EWS-FLI1_loop_and_bridge_target	0.304132772
HIST1H2BJ	222.4448052	35.12609215	4_EWS-FLI1_loop_and_bridge_target	-2.628805396
HIST1H2AG	144.6853265	31.75281305	4_EWS-FLI1_loop_and_bridge_target	-2.15316485
HIST1H2BK	118.915861	89.08332708	4_EWS-FLI1_loop_and_bridge_target	-0.412690476
HIST1H4I	32.84706427	3.537345899	4_EWS-FLI1_loop_and_bridge_target	-2.899110156
HIST1H2AH	123.3123706	19.78934769	4_EWS-FLI1_loop_and_bridge_target	-2.580053473
HIST1H2BL	126.9649142	15.81192907	4_EWS-FLI1_loop_and_bridge_target	-2.928191122
HIST1H2AI	100.4996394	20.06735849	4_EWS-FLI1_loop_and_bridge_target	-2.268393262
HIST1H3H	138.2855948	24.95382354	4_EWS-FLI1_loop_and_bridge_target	-2.42402706
HIST1H2AJ	95.63143881	11.13945311	4_EWS-FLI1_loop_and_bridge_target	-2.992789214
HIST1H2BM	186.859902	13.65908698	4_EWS-FLI1_loop_and_bridge_target	-3.679790007
HIST1H4J	6.770444617	1.771382867	4_EWS-FLI1_loop_and_bridge_target	-1.487391117
HIST1H4K	8.177639829	2.487224723	4_EWS-FLI1_loop_and_bridge_target	-1.396043853
HIST1H2AK	38.59106707	15.51958082	4_EWS-FLI1_loop_and_bridge_target	-1.260997872
HIST1H2BN	91.1725444	37.4175135	4_EWS-FLI1_loop_and_bridge_target	-1.26257293
HIST1H2AL	120.0390761	12.68312977	4_EWS-FLI1_loop_and_bridge_target	-3.145002716
HIST1H1B	370.1216294	47.05117811	4_EWS-FLI1_loop_and_bridge_target	-2.949248377
HIST1H3I	147.1784849	39.81081571	4_EWS-FLI1_loop_and_bridge_target	-1.860312536
HIST1H4L	149.7052024	22.8487455	4_EWS-FLI1_loop_and_bridge_target	-2.659743936
HIST1H3J	153.4870931	12.81934692	4_EWS-FLI1_loop_and_bridge_target	-3.482724968
HIST1H2AM	119.9338846	28.14557245	4_EWS-FLI1_loop_and_bridge_target	-2.052869888
HIST1H2BO	174.0333266	27.46555938	4_EWS-FLI1_loop_and_bridge_target	-2.620340283
KIF6	1.144018451	0.511213136	4_EWS-FLI1_loop_and_bridge_target	-0.504610174
LINC00951	1.339819606	0.529746956	4_EWS-FLI1_loop_and_bridge_target	-0.613104278
LRFN2	7.76412024	3.498209561	4_EWS-FLI1_loop_and_bridge_target	-0.962258403
OARD1	19.1509181	10.41865329	4_EWS-FLI1_loop_and_bridge_target	-0.819453061
NFYA	12.54773332	8.253651972	4_EWS-FLI1_loop_and_bridge_target	-0.549956748
MDFI	45.89377705	37.34888251	4_EWS-FLI1_loop_and_bridge_target	-0.290211947
TBCC	29.32911156	12.16499507	2_EWS-FLI1_loop_target	-1.203996256
GLTSCR1L	2.8933524	2.240152305	2_EWS-FLI1_loop_target	-0.264951304
MUT	17.51458253	15.22657518	3_EWS-FLI1_bridge_target	-0.190303486
CENPQ	20.98159526	4.124484278	3_EWS-FLI1_bridge_target	-2.100817364
MLIP	0.112004518	0.273166941	4_EWS-FLI1_loop_and_bridge_target	0.195258952
COL21A1	42.28543785	27.53580063	4_EWS-FLI1_loop_and_bridge_target	-0.601108712
FAM46A	29.96861412	17.98108811	4_EWS-FLI1_loop_and_bridge_target	-0.706244124
IBTK	26.12952871	52.96631992	4_EWS-FLI1_loop_and_bridge_target	0.992195325
TPBG	28.45813563	20.7374094	4_EWS-FLI1_loop_and_bridge_target	-0.438486112
UBE3D	22.46540909	7.926023445	4_EWS-FLI1_loop_and_bridge_target	-1.394446111
DOPEY1	13.63350427	5.099290842	4_EWS-FLI1_loop_and_bridge_target	-1.262561874

ME1	36.71006486	56.71420545	4_EWS-FLI1_loop_and_bridge_target	0.613976828
PRSS35	158.1669578	12.97586237	4_EWS-FLI1_loop_and_bridge_target	-3.50953166
NT5E	16.78428349	338.7986551	4_EWS-FLI1_loop_and_bridge_target	4.256005387
RNGTT	22.88963232	11.77532684	4_EWS-FLI1_loop_and_bridge_target	-0.903024449
PNRC1	18.95879673	37.3740804	4_EWS-FLI1_loop_and_bridge_target	0.943107431
PM20D2	39.57642689	23.17046189	4_EWS-FLI1_loop_and_bridge_target	-0.747396786
GABRR2	0.242397374	1.010165746	4_EWS-FLI1_loop_and_bridge_target	0.694187778
UBE2J1	29.00658796	64.5283821	4_EWS-FLI1_loop_and_bridge_target	1.126840632
ANKRD6	5.083071652	4.353639822	4_EWS-FLI1_loop_and_bridge_target	-0.184279916
MAP3K7	22.07489836	26.85705462	2_EWS-FLI1_loop_target	0.271718434
EPHA7	4.231632521	0.959620852	3_EWS-FLI1_bridge_target	-1.416686659
FUT9	1.287436423	0.392334874	4_EWS-FLI1_loop_and_bridge_target	-0.716225406
UFL1	25.535918	33.71150675	2_EWS-FLI1_loop_target	0.387467532
NDUFAF4	19.82475165	20.23360254	2_EWS-FLI1_loop_target	0.028049872
KLHL32	0.112670697	0.114030263	2_EWS-FLI1_loop_target	0.001761745
ASCC3	19.53807978	27.71507574	4_EWS-FLI1_loop_and_bridge_target	0.483507065
WASF1	26.21847947	13.11774093	4_EWS-FLI1_loop_and_bridge_target	-0.947077222
CDC40	28.8063527	8.722982118	4_EWS-FLI1_loop_and_bridge_target	-1.616149075
METTL24	8.24703919	1.577800557	4_EWS-FLI1_loop_and_bridge_target	-1.842850855
TSPYL4	8.168457025	5.420645328	3_EWS-FLI1_bridge_target	-0.513960653
DSE	11.61834864	23.32053337	3_EWS-FLI1_bridge_target	0.946651751
TSPYL1	21.36378119	22.88265152	3_EWS-FLI1_bridge_target	0.094798883
PKIB	25.5532544	24.83927816	4_EWS-FLI1_loop_and_bridge_target	-0.039322923
SMPDL3A	15.49616661	13.44374174	4_EWS-FLI1_loop_and_bridge_target	-0.191686279
CLVS2	21.37873823	10.14630177	4_EWS-FLI1_loop_and_bridge_target	-1.005563578
RNF217-AS1	0.138461647	0.149230463	4_EWS-FLI1_loop_and_bridge_target	0.013582451
RNF217	4.02928364	6.627508203	4_EWS-FLI1_loop_and_bridge_target	0.600858906
NCOA7	11.78970752	18.97576579	3_EWS-FLI1_bridge_target	0.643267538
PTPRK	23.98162255	29.98948136	4_EWS-FLI1_loop_and_bridge_target	0.310911428
ARHGAP18	32.48882995	42.1865881	4_EWS-FLI1_loop_and_bridge_target	0.366903372
L3MBTL3	15.46976076	7.302732926	4_EWS-FLI1_loop_and_bridge_target	-0.988161402
EPB41L2	54.19040244	23.60083306	4_EWS-FLI1_loop_and_bridge_target	-1.165710235
AKAP7	40.14495902	6.28092368	4_EWS-FLI1_loop_and_bridge_target	-2.498522294
MED23	21.38707214	16.13497415	2_EWS-FLI1_loop_target	-0.385721844
CTGF	20.89542492	96.22780411	4_EWS-FLI1_loop_and_bridge_target	2.15073949
SLC18B1	41.00352321	13.78736094	4_EWS-FLI1_loop_and_bridge_target	-1.506145743
RPS12	552.4774536	616.0646221	3_EWS-FLI1_bridge_target	0.156897035
SNORD101	26.90315689	19.39593274	3_EWS-FLI1_bridge_target	-0.452146868
SNORD100	26.00899011	20.19587557	3_EWS-FLI1_bridge_target	-0.349656134
SNORA33	22.62901997	16.81871365	3_EWS-FLI1_bridge_target	-0.407166602
SGK1	42.82559078	28.58603715	4_EWS-FLI1_loop_and_bridge_target	-0.566857069
LINC01010	0.018554018	0.103865284	4_EWS-FLI1_loop_and_bridge_target	0.116041621
LOC101928304	0.207515897	0.193612128	4_EWS-FLI1_loop_and_bridge_target	-0.016708084
HECA	9.910017652	7.317662283	3_EWS-FLI1_bridge_target	-0.391403421
CITED2	72.76382269	25.2860914	4_EWS-FLI1_loop_and_bridge_target	-1.488613786
LINC01625	0.460307991	0.028161764	4_EWS-FLI1_loop_and_bridge_target	-0.506205411
LOC100507477	1.402433484	0.462325734	4_EWS-FLI1_loop_and_bridge_target	-0.71623178
SF3B5	71.04881532	80.86677285	3_EWS-FLI1_bridge_target	0.184303317
FBXO5	35.03526522	8.307120591	4_EWS-FLI1_loop_and_bridge_target	-1.953002658
MTRF1L	5.898717718	5.145785115	4_EWS-FLI1_loop_and_bridge_target	-0.166730905
TFB1M	12.84837285	9.728447208	4_EWS-FLI1_loop_and_bridge_target	-0.368275191
ANKMY2	34.41507733	22.68835158	4_EWS-FLI1_loop_and_bridge_target	-0.580185884
BZW2	148.941481	96.16505665	4_EWS-FLI1_loop_and_bridge_target	-0.62589008
TSPAN13	141.1389789	72.12824462	4_EWS-FLI1_loop_and_bridge_target	-0.958801604
AHR	20.87971647	27.93930321	4_EWS-FLI1_loop_and_bridge_target	0.403436143
HDAC9	12.53204104	7.716538467	3_EWS-FLI1_bridge_target	-0.634552231

TWIST1	16.03667818	3.770904152	4_EWS-FLI1_loop_and_bridge_target	-1.836309458
TRIL	4.190384322	3.43651516	4_EWS-FLI1_loop_and_bridge_target	-0.226414468
LOC100506497	0.103150136	0.054535419	4_EWS-FLI1_loop_and_bridge_target	-0.065021599
CPVL	94.54559598	71.58403019	4_EWS-FLI1_loop_and_bridge_target	-0.396537212
CHN2	17.31424833	6.496136885	4_EWS-FLI1_loop_and_bridge_target	-1.288747287
FKBP14	25.70034592	88.13469845	2_EWS-FLI1_loop_target	1.739128722
PLEKHA8	6.590778957	8.619678136	2_EWS-FLI1_loop_target	0.341740683
NEUROD6	0.253757608	0.011011888	1_EWS-FLI1_in_promoter	-0.310458494
CCDC129	0.258849385	0.012491701	4_EWS-FLI1_loop_and_bridge_target	-0.3141956
SEPT7-AS1	1.313253099	1.786228016	3_EWS-FLI1_bridge_target	0.268390205
7-Sep	89.55993559	50.63090724	3_EWS-FLI1_bridge_target	-0.810637983
CDK13	18.97103978	17.37243706	3_EWS-FLI1_bridge_target	-0.120366439
GLI3	4.285088217	2.798588156	4_EWS-FLI1_loop_and_bridge_target	-0.47646425
HUS1	14.86374563	7.773585594	2_EWS-FLI1_loop_target	-0.85449498
ABCA13	8.640642106	0.685484279	4_EWS-FLI1_loop_and_bridge_target	-2.515966068
SEMA3C	13.90138474	29.18015143	4_EWS-FLI1_loop_and_bridge_target	1.018153642
CACNA2D1	26.27426081	15.0075899	4_EWS-FLI1_loop_and_bridge_target	-0.768783985
SEMA3A	13.67674744	46.55051509	4_EWS-FLI1_loop_and_bridge_target	1.695928685
STEAP2-AS1	3.722107676	0.550322191	1_EWS-FLI1_in_promoter	-1.606862869
STEAP1	148.4005766	27.5392775	4_EWS-FLI1_loop_and_bridge_target	-2.388164999
STEAP2	39.12704116	10.62763204	1_EWS-FLI1_in_promoter	-1.787017458
CFAP69	1.258284504	1.338640078	4_EWS-FLI1_loop_and_bridge_target	0.050442593
GTPBP10	7.502539391	5.679401096	4_EWS-FLI1_loop_and_bridge_target	-0.348175034
CLDN12	20.1977266	36.81537681	4_EWS-FLI1_loop_and_bridge_target	0.835063447
CDK14	43.06887464	27.29786276	4_EWS-FLI1_loop_and_bridge_target	-0.639066961
FZD1	42.85818674	20.19709924	4_EWS-FLI1_loop_and_bridge_target	-1.048979317
MTERF1	19.0929632	6.422402291	4_EWS-FLI1_loop_and_bridge_target	-1.436732238
AKAP9	17.07501345	21.72977913	4_EWS-FLI1_loop_and_bridge_target	0.330586944
CYP51A1	83.95202283	239.6532286	4_EWS-FLI1_loop_and_bridge_target	1.502235573
CYP51A1-AS1	0.089329119	0.091281502	4_EWS-FLI1_loop_and_bridge_target	0.002583399
GATAD1	6.508169986	9.12111569	4_EWS-FLI1_loop_and_bridge_target	0.430835114
BET1	5.274220688	13.52348514	4_EWS-FLI1_loop_and_bridge_target	1.210879512
COL1A2	109.811677	586.1611734	4_EWS-FLI1_loop_and_bridge_target	2.405646655
SHFM1	120.7162176	101.6615647	4_EWS-FLI1_loop_and_bridge_target	-0.245625252
DLX6-AS1	1.121476961	0.346785943	4_EWS-FLI1_loop_and_bridge_target	-0.655548442
DLX6	2.883832077	0.698036257	3_EWS-FLI1_bridge_target	-1.193613561
DLX5	13.83642451	2.690496619	4_EWS-FLI1_loop_and_bridge_target	-2.00725658
FBXL13	0.347701539	0.638345702	4_EWS-FLI1_loop_and_bridge_target	0.281738774
LRRC17	11.45504375	42.16312316	4_EWS-FLI1_loop_and_bridge_target	1.793069169
ARMC10	12.57365566	11.97062074	4_EWS-FLI1_loop_and_bridge_target	-0.065561795
KMT2E-AS1	2.993364417	2.891958926	4_EWS-FLI1_loop_and_bridge_target	-0.037108247
KMT2E	8.72236929	8.22117928	4_EWS-FLI1_loop_and_bridge_target	-0.076356668
ATXN7L1	4.516512488	1.234344344	4_EWS-FLI1_loop_and_bridge_target	-1.303904948
SYPL1	38.76457173	47.50181839	4_EWS-FLI1_loop_and_bridge_target	0.286555203
LAMB1	81.39232636	164.2728781	4_EWS-FLI1_loop_and_bridge_target	1.00426811
THAP5	18.47792172	21.68017311	4_EWS-FLI1_loop_and_bridge_target	0.219591901
DNAJB9	23.33992651	37.38743252	4_EWS-FLI1_loop_and_bridge_target	0.657309259
MDFIC	20.52139405	6.860035285	4_EWS-FLI1_loop_and_bridge_target	-1.453163837
TFEC	1.624678838	1.266671113	3_EWS-FLI1_bridge_target	-0.211565826
TES	103.4413216	140.0615923	4_EWS-FLI1_loop_and_bridge_target	0.433632611
CAPZA2	73.19866249	79.89466155	4_EWS-FLI1_loop_and_bridge_target	0.124651317
ST7-AS1	0.810559014	2.022970784	3_EWS-FLI1_bridge_target	0.739531835
ST7	8.376717296	8.135620716	3_EWS-FLI1_bridge_target	-0.037580181
ST7-OT4	0.908596092	0.840150336	3_EWS-FLI1_bridge_target	-0.052688188
CTTNBP2	11.09486769	7.138209836	4_EWS-FLI1_loop_and_bridge_target	-0.571611602
LSM8	6.525112279	6.999306159	4_EWS-FLI1_loop_and_bridge_target	0.088161762

AASS	29.27864518	6.856089617	2_EWS-FLI1_loop_target	-1.946417361
FEZF1	34.90422563	1.287629149	1_EWS-FLI1_in_promoter	-3.972228549
FEZF1-AS1	65.42717482	3.090071583	1_EWS-FLI1_in_promoter	-4.021575559
CADPS2	43.53331008	39.92295366	4_EWS-FLI1_loop_and_bridge_target	-0.121974572
IQUB	0.137514094	0.189395997	4_EWS-FLI1_loop_and_bridge_target	0.064344705
NDUFA5	15.83612543	16.05709679	4_EWS-FLI1_loop_and_bridge_target	0.01881195
WASL	20.15727392	18.54183335	4_EWS-FLI1_loop_and_bridge_target	-0.114587928
TMEM229A	14.78505342	1.001622646	1_EWS-FLI1_in_promoter	-2.979317221
POT1	13.40541551	8.920299347	2_EWS-FLI1_loop_target	-0.538155714
POT1-AS1	0.02454821	0.040081048	2_EWS-FLI1_loop_target	0.021708082
RBM28	28.32945594	13.66890718	2_EWS-FLI1_loop_target	-0.999588914
PRRT4	15.85375691	1.872856608	1_EWS-FLI1_in_promoter	-2.55251233
METTL2B	24.99711497	23.03873001	2_EWS-FLI1_loop_target	-0.11299085
FAM71F1	0.494131387	0.14713535	3_EWS-FLI1_bridge_target	-0.381271393
UBE2H	21.70520299	29.72165618	3_EWS-FLI1_bridge_target	0.436233058
LINC-PINT	4.105056342	5.294543211	4_EWS-FLI1_loop_and_bridge_target	0.302174803
MKLN1	17.48204975	13.15486057	4_EWS-FLI1_loop_and_bridge_target	-0.384827229
MKLN1-AS	0.953654769	0.790896853	4_EWS-FLI1_loop_and_bridge_target	-0.125493304
NUP205	71.56424886	33.33815696	2_EWS-FLI1_loop_target	-1.079446325
KDM7A	5.309264575	3.091910387	2_EWS-FLI1_loop_target	-0.6246973
JHDM1D-AS1	1.553912014	0.634911703	2_EWS-FLI1_loop_target	-0.643496101
CUL1	57.64787986	45.51330275	4_EWS-FLI1_loop_and_bridge_target	-0.334435571
EZH2	60.44976121	19.08594914	4_EWS-FLI1_loop_and_bridge_target	-1.613220768
GHET1	0.44345863	1.617372543	4_EWS-FLI1_loop_and_bridge_target	0.858589524
PDIA4	234.0820479	427.60283	3_EWS-FLI1_bridge_target	0.866477005
LOC101927752	4.640625247	2.40631336	3_EWS-FLI1_bridge_target	-0.72764393
CLN8	11.30656521	16.69817995	3_EWS-FLI1_bridge_target	0.524172845
ARHGEF10	18.76386661	21.36683519	3_EWS-FLI1_bridge_target	0.178495912
LOC101928058	1.194346804	0.608479766	4_EWS-FLI1_loop_and_bridge_target	-0.448093766
KBTBD11-OT1	1.567565089	0.746503098	4_EWS-FLI1_loop_and_bridge_target	-0.555931648
KBTBD11	15.699399	14.98762834	4_EWS-FLI1_loop_and_bridge_target	-0.062840242
LOC100287015	1.090978323	1.077175779	4_EWS-FLI1_loop_and_bridge_target	-0.009554798
MCPH1	7.295517947	7.080517467	4_EWS-FLI1_loop_and_bridge_target	-0.037884377
MCPH1-AS1	0.756155569	0.466488852	4_EWS-FLI1_loop_and_bridge_target	-0.260054547
AGPAT5	35.96385282	33.49603024	4_EWS-FLI1_loop_and_bridge_target	-0.099684792
ERI1	13.32204235	12.80844625	3_EWS-FLI1_bridge_target	-0.052686244
PPP1R3B	7.526739313	10.97637077	3_EWS-FLI1_bridge_target	0.490124736
LOC101929128	0.50081103	0.150189492	3_EWS-FLI1_bridge_target	-0.383870773
TNKS	13.94849425	13.49859155	3_EWS-FLI1_bridge_target	-0.044087412
LONRF1	62.15920159	38.43723266	4_EWS-FLI1_loop_and_bridge_target	-0.679434614
LOC340357	0.313864129	0.184948372	4_EWS-FLI1_loop_and_bridge_target	-0.148991887
LINC00681	0.15181899	0	3_EWS-FLI1_bridge_target	-0.203914013
KIAA1456	54.93462747	9.154402357	4_EWS-FLI1_loop_and_bridge_target	-2.461636358
DLC1	34.8218488	43.58351561	4_EWS-FLI1_loop_and_bridge_target	0.315670586
LOC102725080	0.013452316	0.150611966	4_EWS-FLI1_loop_and_bridge_target	0.183123169
SLC7A2	17.80909819	4.252438916	3_EWS-FLI1_bridge_target	-1.840371296
XPO7	26.55717798	16.76729597	3_EWS-FLI1_bridge_target	-0.633204022
C8orf58	8.501863434	10.84764232	4_EWS-FLI1_loop_and_bridge_target	0.318317614
DOCK5	2.232667433	6.611309362	4_EWS-FLI1_loop_and_bridge_target	1.235419565
PPP2R2A	33.98093183	15.71995009	4_EWS-FLI1_loop_and_bridge_target	-1.06499818
BNIP3L	44.80239119	63.81932465	4_EWS-FLI1_loop_and_bridge_target	0.501001071
PNMA2	57.40336786	21.49608194	4_EWS-FLI1_loop_and_bridge_target	-1.376377811
DPYSL2	184.3639719	150.3867237	4_EWS-FLI1_loop_and_bridge_target	-0.292122174
TRIM35	20.36226441	15.04592175	4_EWS-FLI1_loop_and_bridge_target	-0.412857915
PTK2B	2.593024113	2.889775018	4_EWS-FLI1_loop_and_bridge_target	0.114488096
CLU	54.61346879	33.55888126	4_EWS-FLI1_loop_and_bridge_target	-0.68637781

CCDC25	14.73228121	26.82684611	4_EWS-FLI1_loop_and_bridge_target	0.822749524
ESCO2	25.00808698	2.260614741	4_EWS-FLI1_loop_and_bridge_target	-2.995744392
PBK	53.48462971	4.338745448	3_EWS-FLI1_bridge_target	-3.351276631
ELP3	33.55816827	28.46502661	4_EWS-FLI1_loop_and_bridge_target	-0.230023181
ZNF395	19.81171425	10.40423821	2_EWS-FLI1_loop_target	-0.867825724
LEPROTL1	39.71723714	29.27136616	3_EWS-FLI1_bridge_target	-0.427685885
NRG1	4.953310977	4.827738435	4_EWS-FLI1_loop_and_bridge_target	-0.03075613
NRG1-IT1	1.255036983	0.188862624	4_EWS-FLI1_loop_and_bridge_target	-0.923569077
FUT10	8.912934643	8.976592054	4_EWS-FLI1_loop_and_bridge_target	0.009234865
TTI2	7.038730585	4.125448713	4_EWS-FLI1_loop_and_bridge_target	-0.649289387
RNF122	6.580769293	2.154668299	4_EWS-FLI1_loop_and_bridge_target	-1.26485594
LINC01605	4.387617624	11.5266928	4_EWS-FLI1_loop_and_bridge_target	1.217286209
ZNF703	5.334925652	2.213397767	4_EWS-FLI1_loop_and_bridge_target	-0.979228113
LOC102723701	1.693444035	0.758619331	1_EWS-FLI1_in_promoter	-0.615008857
ERLIN2	23.09846124	35.4506689	1_EWS-FLI1_in_promoter	0.59700426
LOC728024	1.804721514	0.989533747	4_EWS-FLI1_loop_and_bridge_target	-0.495427159
PROSC	26.86079197	32.95781438	4_EWS-FLI1_loop_and_bridge_target	0.285507337
ADGRA2	23.84016064	22.47947239	4_EWS-FLI1_loop_and_bridge_target	-0.081274514
BRF2	20.01551199	10.58357814	4_EWS-FLI1_loop_and_bridge_target	-0.859373637
ADRB3	23.18733843	6.767577352	1_EWS-FLI1_in_promoter	-1.638715416
EIF4EBP1	143.9625781	137.3642495	4_EWS-FLI1_loop_and_bridge_target	-0.06720929
ASH2L	29.40753611	11.99206144	4_EWS-FLI1_loop_and_bridge_target	-1.226798561
STAR	2.313958007	0.401762309	4_EWS-FLI1_loop_and_bridge_target	-1.241313583
LSM1	52.54898536	46.94773454	4_EWS-FLI1_loop_and_bridge_target	-0.159396587
BAG4	30.41644489	18.43923673	4_EWS-FLI1_loop_and_bridge_target	-0.69254836
DDHD2	29.53824285	15.5476649	4_EWS-FLI1_loop_and_bridge_target	-0.883989406
PLPP5	31.27228281	52.20314011	4_EWS-FLI1_loop_and_bridge_target	0.721215766
WHSC1L1	15.87227496	11.38542741	4_EWS-FLI1_loop_and_bridge_target	-0.446010857
LETM2	2.578269402	1.489400805	4_EWS-FLI1_loop_and_bridge_target	-0.52346348
C8orf86	0.113358179	0.132203837	4_EWS-FLI1_loop_and_bridge_target	0.02421592
TACC1	24.29924482	25.81372427	4_EWS-FLI1_loop_and_bridge_target	0.083877294
TM2D2	15.54783237	14.54205893	4_EWS-FLI1_loop_and_bridge_target	-0.090464611
ADAM9	77.59661959	211.9026195	4_EWS-FLI1_loop_and_bridge_target	1.437654531
SNTG1	0.447876727	0.119151272	3_EWS-FLI1_bridge_target	-0.371533722
TCEA1	41.53226141	51.62219175	4_EWS-FLI1_loop_and_bridge_target	0.307113775
RP1	0.669970788	0.294944898	4_EWS-FLI1_loop_and_bridge_target	-0.366932156
XKR4	11.09222397	6.440751765	4_EWS-FLI1_loop_and_bridge_target	-0.700559312
TMEM68	11.68768899	5.074997659	4_EWS-FLI1_loop_and_bridge_target	-1.062473554
TGS1	31.52993352	16.49180156	4_EWS-FLI1_loop_and_bridge_target	-0.895088988
RPS20	247.4679416	209.3334164	4_EWS-FLI1_loop_and_bridge_target	-0.240381647
SNORD54	23.07648646	8.121092822	4_EWS-FLI1_loop_and_bridge_target	-1.400346279
LOC286178	1.695878005	0.067741698	4_EWS-FLI1_loop_and_bridge_target	-1.336192533
FAM110B	2.309400404	1.198551266	4_EWS-FLI1_loop_and_bridge_target	-0.59001668
SDCBP	155.9701569	141.4129594	4_EWS-FLI1_loop_and_bridge_target	-0.140409864
LINC01301	1.006318125	0.174122126	4_EWS-FLI1_loop_and_bridge_target	-0.772967902
RAB2A	47.79287291	59.35438987	4_EWS-FLI1_loop_and_bridge_target	0.306788278
CHD7	5.900645632	8.226092612	4_EWS-FLI1_loop_and_bridge_target	0.418988426
SLCO5A1	40.601644	24.85867205	4_EWS-FLI1_loop_and_bridge_target	-0.685992353
LACTB2	19.62600155	11.63785351	4_EWS-FLI1_loop_and_bridge_target	-0.706712727
XKR9	0.142447447	0.0874364	4_EWS-FLI1_loop_and_bridge_target	-0.071196777
MSC	33.39398108	20.58795316	4_EWS-FLI1_loop_and_bridge_target	-0.671929654
MSC-AS1	6.881060578	6.766200428	4_EWS-FLI1_loop_and_bridge_target	-0.02118085
STAU2	18.89911096	9.586401869	3_EWS-FLI1_bridge_target	-0.91049165
TCEB1	37.38813033	29.72033031	3_EWS-FLI1_bridge_target	-0.321466568
TMEM70	30.32861365	19.35652342	3_EWS-FLI1_bridge_target	-0.621989737
LY96	41.07851576	31.41937035	4_EWS-FLI1_loop_and_bridge_target	-0.376227746

PI15	26.20958224	12.40062231	4_EWS-FLI1_loop_and_bridge_target	-1.021814807
ZFHX4-AS1	0.335599872	0.911510663	4_EWS-FLI1_loop_and_bridge_target	0.517225387
ZFHX4	3.946989186	1.513931885	4_EWS-FLI1_loop_and_bridge_target	-0.976605185
PEX2	7.416897598	6.833491419	3_EWS-FLI1_bridge_target	-0.103633099
MRPS28	49.38776962	44.90068635	4_EWS-FLI1_loop_and_bridge_target	-0.134557871
TPD52	17.66747641	16.02471561	4_EWS-FLI1_loop_and_bridge_target	-0.132896209
ZBTB10	6.008000144	5.430688154	4_EWS-FLI1_loop_and_bridge_target	-0.124029675
ZNF704	35.35518761	10.50802704	1_EWS-FLI1_in_promoter	-1.659520725
PAG1	3.146947775	6.082631702	4_EWS-FLI1_loop_and_bridge_target	0.772235645
GDF6	46.44898099	12.68921072	4_EWS-FLI1_loop_and_bridge_target	-1.793337837
UQCRB	18.02678281	18.03545849	4_EWS-FLI1_loop_and_bridge_target	0.000657679
RPL30	442.7107515	499.6445938	4_EWS-FLI1_loop_and_bridge_target	0.17416729
RIDA	55.59007738	27.68042348	4_EWS-FLI1_loop_and_bridge_target	-0.980482784
POP1	19.24279444	9.000446065	4_EWS-FLI1_loop_and_bridge_target	-1.01734411
MED30	10.30581212	4.74725858	2_EWS-FLI1_loop_target	-0.976118762
EXT1	54.09414892	71.38299575	4_EWS-FLI1_loop_and_bridge_target	0.393751708
RNF139-AS1	0.635339391	1.122176871	4_EWS-FLI1_loop_and_bridge_target	0.375954825
RNF139	32.24393315	39.30190143	4_EWS-FLI1_loop_and_bridge_target	0.277756826
TRIB1	26.76353064	28.8846527	4_EWS-FLI1_loop_and_bridge_target	0.106213732
FAM84B	125.69449	30.84756188	1_EWS-FLI1_in_promoter	-1.992098947
CASC8	0.092411559	0.030792819	4_EWS-FLI1_loop_and_bridge_target	-0.083762093
CASC11	0.268479898	0.234835762	4_EWS-FLI1_loop_and_bridge_target	-0.038781486
MYC	82.91472858	85.88835136	4_EWS-FLI1_loop_and_bridge_target	0.050238724
PVT1	14.96152668	13.97748914	1_EWS-FLI1_in_promoter	-0.091802861
TMEM75	0.618495839	0.978247327	4_EWS-FLI1_loop_and_bridge_target	0.289569152
FAM49B	50.94919176	33.48939919	3_EWS-FLI1_bridge_target	-0.590948306
ASAP1	31.66495323	29.57336911	4_EWS-FLI1_loop_and_bridge_target	-0.095468032
TMEM71	22.70808753	12.93903312	2_EWS-FLI1_loop_target	-0.766248797
AGO2	26.76265154	14.44119166	3_EWS-FLI1_bridge_target	-0.846361267
PUM3	49.30429455	57.33082826	2_EWS-FLI1_loop_target	0.21357699
RFX3	0.757995536	1.826210808	4_EWS-FLI1_loop_and_bridge_target	0.684937674
GLIS3	0.144488988	0.856337714	4_EWS-FLI1_loop_and_bridge_target	0.697755614
SPATA6L	6.267457521	1.883534106	1_EWS-FLI1_in_promoter	-1.333612647
PLPP6	31.24627452	11.47497349	1_EWS-FLI1_in_promoter	-1.370095743
CDC37L1-AS1	2.701526699	1.593859339	4_EWS-FLI1_loop_and_bridge_target	-0.513020188
CDC37L1	22.69380759	14.96517342	4_EWS-FLI1_loop_and_bridge_target	-0.569581834
AK3	78.28852907	39.62424492	4_EWS-FLI1_loop_and_bridge_target	-0.964771164
RCL1	25.66063948	18.09292455	4_EWS-FLI1_loop_and_bridge_target	-0.481673282
JAK2	7.33527977	6.662989264	4_EWS-FLI1_loop_and_bridge_target	-0.121323339
PLGRKT	38.10489143	32.15059401	4_EWS-FLI1_loop_and_bridge_target	-0.238314356
PDCD1LG2	2.067450661	31.36970689	4_EWS-FLI1_loop_and_bridge_target	3.399532258
RIC1	13.68762205	18.17096314	4_EWS-FLI1_loop_and_bridge_target	0.384321979
NFIB	9.486445723	4.695043759	4_EWS-FLI1_loop_and_bridge_target	-0.880746941
ZDHHC21	13.18664883	6.743822795	4_EWS-FLI1_loop_and_bridge_target	-0.873415992
CER1	69.29900635	1.885961786	1_EWS-FLI1_in_promoter	-4.606380196
FREM1	3.972386558	2.449488201	3_EWS-FLI1_bridge_target	-0.527556132
LOC389705	0.690833974	0.180473276	4_EWS-FLI1_loop_and_bridge_target	-0.518369623
MLLT3	7.822882499	3.038020325	4_EWS-FLI1_loop_and_bridge_target	-1.127601899
FOCAD-AS1	0.904453557	0.731340373	4_EWS-FLI1_loop_and_bridge_target	-0.137487727
LINGO2	6.750160622	8.822630012	4_EWS-FLI1_loop_and_bridge_target	0.341883148
EBLN3	30.61253821	27.0683956	4_EWS-FLI1_loop_and_bridge_target	-0.171550275
ZCCHC7	21.10509942	20.45179041	4_EWS-FLI1_loop_and_bridge_target	-0.04328116
GRHPR	65.41615974	36.59102465	4_EWS-FLI1_loop_and_bridge_target	-0.821146066
ZBTB5	13.5647606	12.06470203	4_EWS-FLI1_loop_and_bridge_target	-0.156807767
VPS13A	12.34170862	8.638227026	2_EWS-FLI1_loop_target	-0.46910375
GNAQ	25.33469521	22.65431439	4_EWS-FLI1_loop_and_bridge_target	-0.154861417

GAS1	26.83137594	6.60972435	4_EWS-FLI1_loop_and_bridge_target	-1.870796134
GAS1RR	0.235409391	0.075923808	4_EWS-FLI1_loop_and_bridge_target	-0.199413286
DAPK1	100.1668549	23.28662607	4_EWS-FLI1_loop_and_bridge_target	-2.058502712
CDK20	4.766566938	7.226092107	4_EWS-FLI1_loop_and_bridge_target	0.512494544
SPIN1	72.32303082	31.67731056	4_EWS-FLI1_loop_and_bridge_target	-1.165977171
NXNL2	0.422485238	0.284684335	4_EWS-FLI1_loop_and_bridge_target	-0.146999768
LOC286238	0.112194679	0.069785016	4_EWS-FLI1_loop_and_bridge_target	-0.056088437
C9orf47	0.705883882	0.658588966	4_EWS-FLI1_loop_and_bridge_target	-0.040563048
S1PR3	15.34520474	27.9508075	4_EWS-FLI1_loop_and_bridge_target	0.82473614
SHC3	0.557477839	1.220530507	4_EWS-FLI1_loop_and_bridge_target	0.511692756
CKS2	154.8553697	41.62489304	4_EWS-FLI1_loop_and_bridge_target	-1.870439742
SECISBP2	16.41943773	16.80781019	4_EWS-FLI1_loop_and_bridge_target	0.031812063
TNC	170.8192424	536.7772457	4_EWS-FLI1_loop_and_bridge_target	1.646117098
PAPPA	20.7532269	16.64792178	1_EWS-FLI1_in_promoter	-0.301731126
SPTAN1	79.3940931	52.52155633	2_EWS-FLI1_loop_target	-0.586969436
DOLK	15.63432509	15.86086138	3_EWS-FLI1_bridge_target	0.019514909
NUP188	41.95322514	25.54958115	3_EWS-FLI1_bridge_target	-0.694077359
CRAT	8.301557751	8.588404214	3_EWS-FLI1_bridge_target	0.043818382
PTPA	52.93208614	41.93441208	3_EWS-FLI1_bridge_target	-0.329009403
LINC00963	9.737622725	9.012196414	4_EWS-FLI1_loop_and_bridge_target	-0.100916122
NTMT1	14.61966095	11.92141528	4_EWS-FLI1_loop_and_bridge_target	-0.27359904
ASB6	10.48201402	11.55596539	3_EWS-FLI1_bridge_target	0.128997236
PRRX2	9.596259147	8.480638779	4_EWS-FLI1_loop_and_bridge_target	-0.16049886
TOR1B	46.04595298	31.07467435	4_EWS-FLI1_loop_and_bridge_target	-0.552636006
TOR1A	32.05154071	18.59328318	4_EWS-FLI1_loop_and_bridge_target	-0.75435836
C9orf78	55.70404087	40.66249688	4_EWS-FLI1_loop_and_bridge_target	-0.444702244
USP20	5.93878813	5.682561101	4_EWS-FLI1_loop_and_bridge_target	-0.054282592
FNBP1	50.13435248	14.75394817	4_EWS-FLI1_loop_and_bridge_target	-1.698579397
GPR107	27.89530602	38.04874285	4_EWS-FLI1_loop_and_bridge_target	0.434440953
ASS1	4.664983508	60.35448529	4_EWS-FLI1_loop_and_bridge_target	3.437025154
OLFM1	93.12352361	15.2595121	4_EWS-FLI1_loop_and_bridge_target	-2.533271363
C9orf62	0.371956525	0	4_EWS-FLI1_loop_and_bridge_target	-0.456234766
NLGN4X	20.98989027	9.416776949	4_EWS-FLI1_loop_and_bridge_target	-1.077931442
LOC105373156	0.337135408	0.154249011	4_EWS-FLI1_loop_and_bridge_target	-0.212191074
ADGRG2	77.39446173	46.91179454	4_EWS-FLI1_loop_and_bridge_target	-0.710370886
PDHA1	47.46308252	40.09416755	4_EWS-FLI1_loop_and_bridge_target	-0.237952523
EIF2S3	114.252531	167.682539	2_EWS-FLI1_loop_target	0.549512209
POLA1	22.67319895	5.398991762	4_EWS-FLI1_loop_and_bridge_target	-1.887338156
ARX	4.463922033	0.500939381	4_EWS-FLI1_loop_and_bridge_target	-1.864071186
TMEM47	48.94443507	15.540636	3_EWS-FLI1_bridge_target	-1.59430923
LINC01204	0.010807454	0.07310424	4_EWS-FLI1_loop_and_bridge_target	0.086282017
LOC401585	1.666365233	1.173970069	4_EWS-FLI1_loop_and_bridge_target	-0.294542333
LINC01186	1.226604724	0.236150365	1_EWS-FLI1_in_promoter	-0.848991225
ZNF674	2.465768996	2.477973648	4_EWS-FLI1_loop_and_bridge_target	0.005071504
ZNF674-AS1	6.30515291	9.200722318	4_EWS-FLI1_loop_and_bridge_target	0.481684937
RGN	0.632836977	0.110706802	3_EWS-FLI1_bridge_target	-0.555902727
SSX7	2.921722471	0.612675989	1_EWS-FLI1_in_promoter	-1.282030835
MSN	245.2584156	246.5175304	4_EWS-FLI1_loop_and_bridge_target	0.00735768
EFNB1	44.07565147	27.04819036	4_EWS-FLI1_loop_and_bridge_target	-0.68444065
PJA1	27.51149871	27.1720198	4_EWS-FLI1_loop_and_bridge_target	-0.017280875
COX7B	279.5267271	224.9465676	4_EWS-FLI1_loop_and_bridge_target	-0.312156589
PGK1	269.461351	205.0512607	4_EWS-FLI1_loop_and_bridge_target	-0.392419157
GPR174	19.69688472	0.945814412	4_EWS-FLI1_loop_and_bridge_target	-3.410967611
ITM2A	409.3271752	162.1522586	4_EWS-FLI1_loop_and_bridge_target	-1.330555745
KLHL13	22.42972738	6.969815104	4_EWS-FLI1_loop_and_bridge_target	-1.555722008
XIAP	13.46916047	7.7280066	3_EWS-FLI1_bridge_target	-0.729257119

LOC101928402	1.33004612	0.255485942	4_EWS-FLI1_loop_and_bridge_target	-0.892112637
STAG2	50.05988268	42.79191547	4_EWS-FLI1_loop_and_bridge_target	-0.221525668
FGF13	20.66972447	3.398542428	4_EWS-FLI1_loop_and_bridge_target	-2.300583376
ATP11C	74.65742255	60.67417074	4_EWS-FLI1_loop_and_bridge_target	-0.294815216
PFKP	78.89178539	63.76935022	4_EWS-FLI1_loop_and_bridge_target	-0.3027359
KLF6	28.99058838	47.81287018	4_EWS-FLI1_loop_and_bridge_target	0.702751758
LINC00702	0.541711652	2.000606731	4_EWS-FLI1_loop_and_bridge_target	0.960721285
LINC00703	0.333791405	0.073276953	3_EWS-FLI1_bridge_target	-0.313510654
LINC00705	0.075684244	0.164764425	4_EWS-FLI1_loop_and_bridge_target	0.114783546
USP6NL	25.00068533	16.44165098	3_EWS-FLI1_bridge_target	-0.576013042
UPF2	20.36427652	12.60914987	3_EWS-FLI1_bridge_target	-0.650623514
DHTKD1	25.13891994	7.667670479	3_EWS-FLI1_bridge_target	-1.592483318
SEC61A2	5.034860318	3.077333954	3_EWS-FLI1_bridge_target	-0.565694254
NUDT5	35.87690438	26.75029958	4_EWS-FLI1_loop_and_bridge_target	-0.410214208
CDC123	123.318211	109.7676158	4_EWS-FLI1_loop_and_bridge_target	-0.166501494
CAMK1D	6.408757775	7.840535871	4_EWS-FLI1_loop_and_bridge_target	0.254902155
TRDMT1	2.539827365	1.514191134	4_EWS-FLI1_loop_and_bridge_target	-0.493584672
STAM-AS1	0.817921207	0.38381759	4_EWS-FLI1_loop_and_bridge_target	-0.393635886
STAM	16.40504646	16.2882986	4_EWS-FLI1_loop_and_bridge_target	-0.00970977
CACNB2	6.040125607	1.52502156	1_EWS-FLI1_in_promoter	-1.479305463
ARL5B	43.36447813	36.6100382	4_EWS-FLI1_loop_and_bridge_target	-0.238287226
PLXDC2	10.77170775	15.98703706	4_EWS-FLI1_loop_and_bridge_target	0.529110603
CASC10	7.277236047	1.040880886	4_EWS-FLI1_loop_and_bridge_target	-2.019957117
SKIDA1	3.01612499	0.517711507	4_EWS-FLI1_loop_and_bridge_target	-1.403906586
MLLT10	14.32182198	9.078981396	4_EWS-FLI1_loop_and_bridge_target	-0.60423802
LOC100130992	0.56705342	0.209419695	4_EWS-FLI1_loop_and_bridge_target	-0.373739384
COMMD3	25.4920523	21.83889492	3_EWS-FLI1_bridge_target	-0.214066765
C10orf67	0.480992274	0.614525479	4_EWS-FLI1_loop_and_bridge_target	0.124546094
OTUD1	19.54909684	12.34935871	4_EWS-FLI1_loop_and_bridge_target	-0.622304549
KIAA1217	11.98450453	8.596615716	4_EWS-FLI1_loop_and_bridge_target	-0.436193336
PDSS1	25.01434242	12.72334613	4_EWS-FLI1_loop_and_bridge_target	-0.922674947
ABI1	34.76622704	30.7478955	4_EWS-FLI1_loop_and_bridge_target	-0.171936975
ANKRD26	13.46201536	6.632814114	4_EWS-FLI1_loop_and_bridge_target	-0.921981651
YME1L1	91.63860509	118.0960821	4_EWS-FLI1_loop_and_bridge_target	0.362440518
MASTL	26.91589941	12.66442058	4_EWS-FLI1_loop_and_bridge_target	-1.030662753
ACBD5	14.48513724	14.64712871	4_EWS-FLI1_loop_and_bridge_target	0.015013774
WAC-AS1	5.56748702	3.594291809	4_EWS-FLI1_loop_and_bridge_target	-0.515498951
WAC	34.87219513	27.38935981	4_EWS-FLI1_loop_and_bridge_target	-0.337515715
BAMBI	39.77481411	31.98197918	4_EWS-FLI1_loop_and_bridge_target	-0.306000325
SVIL-AS1	12.84945525	4.528408505	4_EWS-FLI1_loop_and_bridge_target	-1.324893103
SVIL	11.38201512	13.7743835	4_EWS-FLI1_loop_and_bridge_target	0.254851805
KIAA1462	17.38219692	9.736172872	4_EWS-FLI1_loop_and_bridge_target	-0.775829391
MTPAP	19.07019403	16.23213761	4_EWS-FLI1_loop_and_bridge_target	-0.219952888
MAP3K8	5.24467367	7.644315862	4_EWS-FLI1_loop_and_bridge_target	0.469125607
EPC1	25.55423578	12.20888232	4_EWS-FLI1_loop_and_bridge_target	-1.007433613
LOC102031319	2.781649949	0.479127999	4_EWS-FLI1_loop_and_bridge_target	-1.354268922
LOC101929431	0.113066019	0.095677416	4_EWS-FLI1_loop_and_bridge_target	-0.022716056
CCDC7	2.247701198	1.629969022	4_EWS-FLI1_loop_and_bridge_target	-0.304373099
ITGB1	64.43172671	146.7394657	4_EWS-FLI1_loop_and_bridge_target	1.174993017
NRP1	14.60727669	73.22290786	4_EWS-FLI1_loop_and_bridge_target	2.249645699
CUL2	20.63879922	26.56167709	4_EWS-FLI1_loop_and_bridge_target	0.349043233
CREM	11.45060368	11.21595211	4_EWS-FLI1_loop_and_bridge_target	-0.027449383
CCNY	28.19196026	29.39195303	4_EWS-FLI1_loop_and_bridge_target	0.058118296
FZD8	16.17915819	11.95809844	4_EWS-FLI1_loop_and_bridge_target	-0.406805321
ARID5B	20.15388774	28.6594548	4_EWS-FLI1_loop_and_bridge_target	0.487569247
ADO	31.81007362	17.25323969	4_EWS-FLI1_loop_and_bridge_target	-0.845986286

EGR2	18.60085112	3.292552857	4_EWS-FLI1_loop_and_bridge_target	-2.191008497
JMJD1C	23.27065933	20.90714508	4_EWS-FLI1_loop_and_bridge_target	-0.147811815
LOC105378330	0.186366526	0.298079632	3_EWS-FLI1_bridge_target	0.129829092
JMJD1C-AS1	0.191196329	0.205143884	3_EWS-FLI1_bridge_target	0.01679419
REEP3	43.68536744	37.27919599	4_EWS-FLI1_loop_and_bridge_target	-0.22324196
DNAJC12	126.3094253	79.94006075	4_EWS-FLI1_loop_and_bridge_target	-0.653413395
SIRT1	21.49124717	8.780324662	4_EWS-FLI1_loop_and_bridge_target	-1.201409401
HERC4	18.266841	23.08843259	4_EWS-FLI1_loop_and_bridge_target	0.322220476
MYPN	1.876632439	4.289603476	4_EWS-FLI1_loop_and_bridge_target	0.878778685
PBLD	2.993823669	2.030306293	4_EWS-FLI1_loop_and_bridge_target	-0.398307014
HNRNPH3	37.64352644	16.64034972	4_EWS-FLI1_loop_and_bridge_target	-1.131347592
DNA2	18.56289213	3.46383201	3_EWS-FLI1_bridge_target	-2.131765031
TET1	5.506163015	2.178797579	3_EWS-FLI1_bridge_target	-1.03332582
CCAR1	45.03272169	29.28208029	3_EWS-FLI1_bridge_target	-0.604195427
DDX50	48.05160758	40.58939069	4_EWS-FLI1_loop_and_bridge_target	-0.23808487
KIAA1279	22.37376438	14.44164582	3_EWS-FLI1_bridge_target	-0.598063573
HKDC1	0.667196927	3.296315775	4_EWS-FLI1_loop_and_bridge_target	1.36567551
H2AFY2	96.18732827	23.03768647	4_EWS-FLI1_loop_and_bridge_target	-2.015470172
AIFM2	2.562330841	1.691395547	3_EWS-FLI1_bridge_target	-0.404467073
TYSND1	16.20128726	4.547982059	4_EWS-FLI1_loop_and_bridge_target	-1.632481506
PPA1	257.7102867	219.6533238	3_EWS-FLI1_bridge_target	-0.229555939
LRRC20	13.08700633	2.002204312	3_EWS-FLI1_bridge_target	-2.230270987
EIF4EBP2	26.89076975	30.5302914	3_EWS-FLI1_bridge_target	0.176950752
KAT6B	9.144079733	5.581680515	3_EWS-FLI1_bridge_target	-0.624110088
DUSP13	0.069573351	0.064911867	4_EWS-FLI1_loop_and_bridge_target	-0.006301388
SAMD8	14.11923104	16.41730594	4_EWS-FLI1_loop_and_bridge_target	0.204136723
VDAC2	51.7492456	45.58962759	3_EWS-FLI1_bridge_target	-0.179141663
COMTD1	0.523191596	0.68339214	4_EWS-FLI1_loop_and_bridge_target	0.144273863
PAPSS2	5.778093811	22.89922598	3_EWS-FLI1_bridge_target	1.818012385
KLLN	0.274317782	0.211427225	3_EWS-FLI1_bridge_target	-0.073017354
PTEN	13.85810456	26.42987592	3_EWS-FLI1_bridge_target	0.884498012
ACTA2-AS1	0.730336286	0.594043671	3_EWS-FLI1_bridge_target	-0.118361294
ACTA2	31.91630649	48.23376576	2_EWS-FLI1_loop_target	0.58084563
FAS	10.97229499	12.93903312	2_EWS-FLI1_loop_target	0.219430761
FAS-AS1	1.972081724	0.482986955	2_EWS-FLI1_loop_target	-1.00296788
IFIT3	16.29698514	36.54162474	3_EWS-FLI1_bridge_target	1.117970491
IFIT5	19.76778088	22.97967482	3_EWS-FLI1_bridge_target	0.207465028
SH3PXD2A	17.50376978	2.875406999	4_EWS-FLI1_loop_and_bridge_target	-2.255399485
SLK	32.29003799	33.84499762	4_EWS-FLI1_loop_and_bridge_target	0.065861039
SFR1	12.05218378	4.038745068	4_EWS-FLI1_loop_and_bridge_target	-1.373154835
GSTO1	65.09766502	68.67434259	3_EWS-FLI1_bridge_target	0.076028177
ITPRIP	9.229580346	9.223908602	4_EWS-FLI1_loop_and_bridge_target	-0.000800118
CFAP58-AS1	1.01082063	0.460581108	3_EWS-FLI1_bridge_target	-0.46124192
ADD3-AS1	1.514394424	0.260276303	3_EWS-FLI1_bridge_target	-0.996470913
ADD3	42.07908174	30.98367497	3_EWS-FLI1_bridge_target	-0.429651782
MXI1	13.91002402	10.24467697	3_EWS-FLI1_bridge_target	-0.407040365
ZDHHC6	24.53121944	18.45981482	3_EWS-FLI1_bridge_target	-0.391764465
VTI1A	8.958294669	7.829181974	3_EWS-FLI1_bridge_target	-0.173618928
CASP7	55.40642135	51.99034487	4_EWS-FLI1_loop_and_bridge_target	-0.090129893
DCLRE1A	52.90374382	11.25671753	4_EWS-FLI1_loop_and_bridge_target	-2.136812814
NHLRC2	9.416957621	9.22531278	4_EWS-FLI1_loop_and_bridge_target	-0.026789011
ADRB1	26.02247508	10.90473785	4_EWS-FLI1_loop_and_bridge_target	-1.182623971
ENO4	0.725551696	0.631256016	4_EWS-FLI1_loop_and_bridge_target	-0.081074474
SHTN1	14.91621576	8.581000037	4_EWS-FLI1_loop_and_bridge_target	-0.732249208
VAX1	5.230673063	1.354701028	4_EWS-FLI1_loop_and_bridge_target	-1.403844122
EIF3A	108.2172628	56.6410579	4_EWS-FLI1_loop_and_bridge_target	-0.922032187

SFXN4	43.91220915	30.64080174	4_EWS-FLI1_loop_and_bridge_target	-0.505321531
PRDX3	63.93620503	184.7932081	4_EWS-FLI1_loop_and_bridge_target	1.516602784
GRK5	52.60154091	39.63479292	4_EWS-FLI1_loop_and_bridge_target	-0.399558933
RGS10	26.55304781	43.67205794	4_EWS-FLI1_loop_and_bridge_target	0.697160805
TIAL1	13.58590587	13.73969599	4_EWS-FLI1_loop_and_bridge_target	0.01513178
BAG3	17.63747807	17.09489782	4_EWS-FLI1_loop_and_bridge_target	-0.042623695
SEC23IP	15.8458629	30.65297771	3_EWS-FLI1_bridge_target	0.909946895
PTPRE	4.098572588	5.015523337	2_EWS-FLI1_loop_target	0.238596852
EBF3	31.59004946	7.613107156	4_EWS-FLI1_loop_and_bridge_target	-1.919825856
LINC00959	0.420159481	0.240550598	4_EWS-FLI1_loop_and_bridge_target	-0.195072372
GLRX3	76.4412659	90.42460641	3_EWS-FLI1_bridge_target	0.239479975
TRIM5	14.70806109	15.83302631	4_EWS-FLI1_loop_and_bridge_target	0.099789459
FAM160A2	9.440428255	11.22710417	4_EWS-FLI1_loop_and_bridge_target	0.22790187
PRKCDBP	1.291266626	1.491595842	4_EWS-FLI1_loop_and_bridge_target	0.120924721
SMPD1	114.1153553	70.96244563	1_EWS-FLI1_in_promoter	-0.677764167
APBB1	40.15578694	16.4161628	4_EWS-FLI1_loop_and_bridge_target	-1.240668505
TRIM3	12.15711524	6.574849443	4_EWS-FLI1_loop_and_bridge_target	-0.796554087
ARFIP2	26.61640319	36.66954935	4_EWS-FLI1_loop_and_bridge_target	0.447873341
TIMM10B	18.77469912	15.7960791	4_EWS-FLI1_loop_and_bridge_target	-0.235531256
RRP8	16.53274985	13.76022229	4_EWS-FLI1_loop_and_bridge_target	-0.248337839
ILK	75.11081316	108.9792633	4_EWS-FLI1_loop_and_bridge_target	0.531058189
TAF10	47.5615261	20.0393184	4_EWS-FLI1_loop_and_bridge_target	-1.206725794
TPP1	3.115103939	6.738915635	4_EWS-FLI1_loop_and_bridge_target	0.911202562
MRPL17	45.25463571	27.51303767	4_EWS-FLI1_loop_and_bridge_target	-0.697976215
RPL27A	44.01400237	51.58550893	3_EWS-FLI1_bridge_target	0.224291443
SNORA3A	19.32538958	13.54580849	3_EWS-FLI1_bridge_target	-0.482679517
SNORA3B	11.68194976	11.17172879	3_EWS-FLI1_bridge_target	-0.059242473
DENND5A	50.389469	71.04012045	3_EWS-FLI1_bridge_target	0.487327848
TMEM41B	38.76664903	66.67096961	3_EWS-FLI1_bridge_target	0.766978063
IPO7	71.75300877	78.42493001	3_EWS-FLI1_bridge_target	0.126585
WEE1	23.05712926	8.138033057	4_EWS-FLI1_loop_and_bridge_target	-1.396508929
SWAP70	20.36215449	12.07625992	3_EWS-FLI1_bridge_target	-0.708107198
LOC440028	0.56286677	1.565165284	4_EWS-FLI1_loop_and_bridge_target	0.714856989
SBF2-AS1	4.09238515	4.12136185	4_EWS-FLI1_loop_and_bridge_target	0.008185958
SBF2	17.78774062	17.84671822	4_EWS-FLI1_loop_and_bridge_target	0.004521747
ADM	12.64050639	40.63397168	4_EWS-FLI1_loop_and_bridge_target	1.609863987
CAND1.11	0.275330219	0.723942501	4_EWS-FLI1_loop_and_bridge_target	0.434840807
RNF141	47.77926509	18.2408276	4_EWS-FLI1_loop_and_bridge_target	-1.342097168
MRVI1-AS1	0.453432716	0.026440767	4_EWS-FLI1_loop_and_bridge_target	-0.50181391
MRVI1	0.960237463	0.119572392	4_EWS-FLI1_loop_and_bridge_target	-0.808080617
CTR9	45.11460764	21.91024133	4_EWS-FLI1_loop_and_bridge_target	-1.009231167
EIF4G2	234.6262704	218.8105427	3_EWS-FLI1_bridge_target	-0.100239813
LOC101928053	0.229386465	0.315930368	3_EWS-FLI1_bridge_target	0.098144644
ZBED5	17.48449623	10.01440445	2_EWS-FLI1_loop_target	-0.746924235
ZBED5-AS1	3.345746796	5.174123202	2_EWS-FLI1_loop_target	0.506630153
MICALCL	0.13406355	0.199348098	4_EWS-FLI1_loop_and_bridge_target	0.080748959
TEAD1	23.75302903	18.36939736	4_EWS-FLI1_loop_and_bridge_target	-0.35382601
CYP2R1	16.87499037	20.48750927	3_EWS-FLI1_bridge_target	0.265555797
CALCB	47.51065584	36.71060756	3_EWS-FLI1_bridge_target	-0.36333129
RPS13	394.0830668	446.8289748	3_EWS-FLI1_bridge_target	0.18079186
RCN1	196.3453998	370.2674258	3_EWS-FLI1_bridge_target	0.911735852
QSER1	21.95168937	14.92486025	4_EWS-FLI1_loop_and_bridge_target	-0.527319636
TCP11L1	25.00548069	23.27077503	4_EWS-FLI1_loop_and_bridge_target	-0.099595527
CSTF3	39.91412403	50.92985525	4_EWS-FLI1_loop_and_bridge_target	0.34396524
CSTF3-AS1	0.105241513	0.063534854	4_EWS-FLI1_loop_and_bridge_target	-0.055494343
HIPK3	63.02204068	50.72745784	4_EWS-FLI1_loop_and_bridge_target	-0.307638371

C11orf91	1.214551919	19.02681131	4_EWS-FLI1_loop_and_bridge_target	3.176846006
CD59	54.21674087	172.4023111	4_EWS-FLI1_loop_and_bridge_target	1.650945486
FBXO3	41.70570387	25.66685658	4_EWS-FLI1_loop_and_bridge_target	-0.679380999
LMO2	30.65738172	27.54939718	4_EWS-FLI1_loop_and_bridge_target	-0.149081656
CAPRIN1	282.4611226	171.000778	1_EWS-FLI1_in_promoter	-0.720735789
NAT10	69.31511821	42.7262778	4_EWS-FLI1_loop_and_bridge_target	-0.685334367
ABTB2	16.81558651	13.59221538	4_EWS-FLI1_loop_and_bridge_target	-0.287941051
APIP	53.64762377	52.02847309	4_EWS-FLI1_loop_and_bridge_target	-0.043391557
PDHX	53.90488714	67.41201055	4_EWS-FLI1_loop_and_bridge_target	0.317315059
FJX1	26.27920609	30.11561085	4_EWS-FLI1_loop_and_bridge_target	0.189836911
PRR5L	22.48232444	7.352465865	1_EWS-FLI1_in_promoter	-1.491301137
TRAF6	12.93245677	14.38897262	4_EWS-FLI1_loop_and_bridge_target	0.143447242
LRRC4C	14.89250294	7.908843832	4_EWS-FLI1_loop_and_bridge_target	-0.835036236
HNRNPKP3	0.474333507	0	2_EWS-FLI1_loop_target	-0.560062912
API5	133.0785806	100.8376997	2_EWS-FLI1_loop_target	-0.396807044
ALKBH3-AS1	0.714738908	0.598930718	4_EWS-FLI1_loop_and_bridge_target	-0.100881496
ACCS	12.10837199	6.471307482	4_EWS-FLI1_loop_and_bridge_target	-0.811055877
EXT2	103.1943471	106.4796882	4_EWS-FLI1_loop_and_bridge_target	0.044787034
ALX4	68.0919002	16.36947615	4_EWS-FLI1_loop_and_bridge_target	-1.991962346
CD82	7.420905044	72.24146136	4_EWS-FLI1_loop_and_bridge_target	3.120613375
CHST1	3.094333037	0.603976919	4_EWS-FLI1_loop_and_bridge_target	-1.351975076
DKFZp779M0652	1.788142309	0.018333352	4_EWS-FLI1_loop_and_bridge_target	-1.453094294
SLC35C1	12.74307009	23.2583313	4_EWS-FLI1_loop_and_bridge_target	0.819775986
CRY2	27.4558804	28.36752929	4_EWS-FLI1_loop_and_bridge_target	0.04549508
PEX16	17.47828057	12.69655714	4_EWS-FLI1_loop_and_bridge_target	-0.432017225
HARBI1	11.45405451	10.8742433	3_EWS-FLI1_bridge_target	-0.06877992
ATG13	44.17287738	75.66242791	3_EWS-FLI1_bridge_target	0.763062877
ARFGAP2	99.21185109	72.62242853	3_EWS-FLI1_bridge_target	-0.444835888
MADD	41.98955259	37.68242326	3_EWS-FLI1_bridge_target	-0.152307921
LOC101928943	3.116821203	3.152173568	3_EWS-FLI1_bridge_target	0.012335959
CELF1	55.22280384	34.56466689	3_EWS-FLI1_bridge_target	-0.660710752
KBTBD4	16.18291996	11.61010608	3_EWS-FLI1_bridge_target	-0.446394808
NDUFS3	146.411131	107.8543438	3_EWS-FLI1_bridge_target	-0.437446488
FNBP4	52.31955243	53.73981136	4_EWS-FLI1_loop_and_bridge_target	0.037925793
FADS1	245.7336675	112.7273999	3_EWS-FLI1_bridge_target	-1.117374709
FADS2	267.7987661	343.4210878	3_EWS-FLI1_bridge_target	0.357646961
ANKRD13D	11.61585294	11.81996358	3_EWS-FLI1_bridge_target	0.023154415
MRGPRF	3.524754157	1.662537078	4_EWS-FLI1_loop_and_bridge_target	-0.765037792
MRGPRF-AS1	0.105731082	0.045529383	4_EWS-FLI1_loop_and_bridge_target	-0.080766953
TPCN2	8.530620234	6.621502546	4_EWS-FLI1_loop_and_bridge_target	-0.322494658
MYEOV	41.07586345	16.32086578	4_EWS-FLI1_loop_and_bridge_target	-1.280481833
CCND1	347.2780281	114.1203615	4_EWS-FLI1_loop_and_bridge_target	-1.597096433
ORAOV1	16.81027331	11.22503173	4_EWS-FLI1_loop_and_bridge_target	-0.542871446
FADD	50.77030212	36.94073421	4_EWS-FLI1_loop_and_bridge_target	-0.44837714
PPFIA1	46.33755822	29.81310928	1_EWS-FLI1_in_promoter	-0.619441022
CTTN	120.4810702	97.47504518	4_EWS-FLI1_loop_and_bridge_target	-0.302901444
FLJ42102	0.053087491	0	3_EWS-FLI1_bridge_target	-0.074625302
DHCR7	98.64394719	204.9328133	3_EWS-FLI1_bridge_target	1.04731965
NADSYN1	15.07768744	24.78577115	3_EWS-FLI1_bridge_target	0.681515283
PGM2L1	8.869881369	16.69514091	4_EWS-FLI1_loop_and_bridge_target	0.842248601
KCNE3	51.65920537	3.898936602	1_EWS-FLI1_in_promoter	-3.426145225
LIPT2	7.73138206	1.883904672	4_EWS-FLI1_loop_and_bridge_target	-1.598186554
LOC100287896	3.649546874	2.149781871	4_EWS-FLI1_loop_and_bridge_target	-0.561838202
POLD3	37.68584867	9.043661439	4_EWS-FLI1_loop_and_bridge_target	-1.94552062
PRCP	44.84579102	47.77179485	4_EWS-FLI1_loop_and_bridge_target	0.089257776
DDIAS	35.45584866	2.811789711	4_EWS-FLI1_loop_and_bridge_target	-3.257609851

RAB30	5.552254006	5.075134416	4_EWS-FLI1_loop_and_bridge_target	-0.109074956
RAB30-AS1	28.04477983	9.175692733	4_EWS-FLI1_loop_and_bridge_target	-1.513151881
PCF11	29.03892563	35.85805449	4_EWS-FLI1_loop_and_bridge_target	0.295146708
ANKRD42	7.671374145	8.109486739	4_EWS-FLI1_loop_and_bridge_target	0.071109135
CCDC90B	27.3854959	17.80851139	4_EWS-FLI1_loop_and_bridge_target	-0.593768275
DLG2	23.7568382	7.809203845	4_EWS-FLI1_loop_and_bridge_target	-1.490743531
CREBZF	22.84560135	17.50588229	4_EWS-FLI1_loop_and_bridge_target	-0.365739246
PICALM	87.68171515	104.3773219	4_EWS-FLI1_loop_and_bridge_target	0.248855842
EED	28.06924462	12.32127226	4_EWS-FLI1_loop_and_bridge_target	-1.125761708
FZD4	50.70245051	10.58202849	4_EWS-FLI1_loop_and_bridge_target	-2.15834471
LOC100506368	8.406929475	1.043196527	4_EWS-FLI1_loop_and_bridge_target	-2.20289591
TMEM135	10.57583122	15.4796424	4_EWS-FLI1_loop_and_bridge_target	0.509569145
CHORDC1	56.71885702	27.68078854	3_EWS-FLI1_bridge_target	-1.008958041
DISC1FP1	4.102643401	0.3535684	4_EWS-FLI1_loop_and_bridge_target	-1.914477029
YAP1	59.69265038	46.66929862	4_EWS-FLI1_loop_and_bridge_target	-0.348461424
BIRC3	4.695582127	130.787121	4_EWS-FLI1_loop_and_bridge_target	4.532222276
BIRC2	25.26638195	154.711966	4_EWS-FLI1_loop_and_bridge_target	2.56759042
TMEM123	102.9743805	62.69997112	4_EWS-FLI1_loop_and_bridge_target	-0.706863465
LOC101928424	0.264947268	0.049439108	4_EWS-FLI1_loop_and_bridge_target	-0.269458785
MMP10	17.05206037	20.68512787	4_EWS-FLI1_loop_and_bridge_target	0.264542442
MMP1	672.6835698	652.4404087	4_EWS-FLI1_loop_and_bridge_target	-0.044015441
DCUN1D5	8.383956797	10.82948732	4_EWS-FLI1_loop_and_bridge_target	0.334119274
DYNC2H1	18.67815767	15.58970726	4_EWS-FLI1_loop_and_bridge_target	-0.246306729
NPAT	23.72801615	8.583335171	3_EWS-FLI1_bridge_target	-1.36754677
ATM	20.29660651	24.0003209	3_EWS-FLI1_bridge_target	0.23132305
ZC3H12C	16.53359998	30.35449326	4_EWS-FLI1_loop_and_bridge_target	0.838549964
RDX	106.5552581	77.64592492	4_EWS-FLI1_loop_and_bridge_target	-0.451634138
LOC105369486	81.50145831	20.14741208	1_EWS-FLI1_in_promoter	-1.963938497
FDX1	55.87381533	24.95446936	4_EWS-FLI1_loop_and_bridge_target	-1.131781597
ARHGAP20	7.934288538	1.299324253	2_EWS-FLI1_loop_target	-1.958142917
SIK2	13.24140799	18.79035095	3_EWS-FLI1_bridge_target	0.47470541
PPP2R1B	20.80981887	27.42875312	3_EWS-FLI1_bridge_target	0.382373036
FDXACB1	6.155948083	2.801893446	3_EWS-FLI1_bridge_target	-0.912424822
C11orf1	10.43056694	6.257827588	3_EWS-FLI1_bridge_target	-0.65528727
DIXDC1	14.71363144	8.959615746	4_EWS-FLI1_loop_and_bridge_target	-0.65785464
TIMM8B	59.31168412	37.07273903	4_EWS-FLI1_loop_and_bridge_target	-0.663679157
SDHD	122.8435809	84.84070279	4_EWS-FLI1_loop_and_bridge_target	-0.5287853
BCO2	1.474090579	2.160092667	3_EWS-FLI1_bridge_target	0.353068545
PTS	65.29321886	49.83546767	4_EWS-FLI1_loop_and_bridge_target	-0.383025895
ANKK1	0.285847158	0.004678862	4_EWS-FLI1_loop_and_bridge_target	-0.355984739
USP28	13.60789902	9.719021126	4_EWS-FLI1_loop_and_bridge_target	-0.446575535
ZBTB16	7.493910553	4.646487834	4_EWS-FLI1_loop_and_bridge_target	-0.589075139
NNMT	0.501060393	53.20324741	4_EWS-FLI1_loop_and_bridge_target	5.174325361
LOC101928940	0.003328842	0.052798713	4_EWS-FLI1_loop_and_bridge_target	0.069435102
C11orf71	6.21377804	3.753169322	4_EWS-FLI1_loop_and_bridge_target	-0.601865238
RBM7	13.62338597	19.34512301	4_EWS-FLI1_loop_and_bridge_target	0.476405605
ZPR1	70.84755236	91.04103578	4_EWS-FLI1_loop_and_bridge_target	0.35733821
SIK3	22.17903107	14.30925326	4_EWS-FLI1_loop_and_bridge_target	-0.598416347
PAFAH1B2	58.43373698	44.88229534	4_EWS-FLI1_loop_and_bridge_target	-0.373344529
SIDT2	15.26820996	21.97505059	4_EWS-FLI1_loop_and_bridge_target	0.498012522
PCSK7	11.19249148	12.74627335	4_EWS-FLI1_loop_and_bridge_target	0.17304759
RNF214	16.63289121	13.74882088	4_EWS-FLI1_loop_and_bridge_target	-0.257669428
HSPA8	838.2785505	567.1060919	4_EWS-FLI1_loop_and_bridge_target	-0.562989337
CLMP	75.74426657	58.84345793	4_EWS-FLI1_loop_and_bridge_target	-0.358865433
BARX2	23.57138029	8.477655195	4_EWS-FLI1_loop_and_bridge_target	-1.374376822
PRDM10	6.32014095	5.140143085	3_EWS-FLI1_bridge_target	-0.253599152

LINC00167	0.605791177	0.581826176	3_EWS-FLI1_bridge_target	-0.021693219
NTM	5.070645432	5.868144491	4_EWS-FLI1_loop_and_bridge_target	0.178070479
OPCML	0.229342732	0.035923069	3_EWS-FLI1_bridge_target	-0.246970317
LINC01252	0.68089018	0.816775542	4_EWS-FLI1_loop_and_bridge_target	0.11215472
ETV6	7.805700478	8.333618561	4_EWS-FLI1_loop_and_bridge_target	0.083998737
LRP6	33.87591643	21.13924007	4_EWS-FLI1_loop_and_bridge_target	-0.655625424
LOH12CR2	0.903806678	0.216614869	4_EWS-FLI1_loop_and_bridge_target	-0.646014447
BORCS5	5.102337257	2.616270954	4_EWS-FLI1_loop_and_bridge_target	-0.754859137
GPR19	1.331617164	0.061437239	4_EWS-FLI1_loop_and_bridge_target	-1.135311858
CDKN1B	23.82679253	26.57548733	4_EWS-FLI1_loop_and_bridge_target	0.151488496
APOLD1	4.326699619	8.522421005	4_EWS-FLI1_loop_and_bridge_target	0.838086487
DDX47	74.2076517	60.67042883	3_EWS-FLI1_bridge_target	-0.286300572
LMO3	6.059803747	2.806126432	2_EWS-FLI1_loop_target	-0.891304593
AEBP2	12.41020241	7.486921597	4_EWS-FLI1_loop_and_bridge_target	-0.660017758
SOX5	2.25229582	7.443017763	4_EWS-FLI1_loop_and_bridge_target	1.376300262
LOC101928471	0.26942525	0.687087545	4_EWS-FLI1_loop_and_bridge_target	0.410359395
BCAT1	59.97941202	93.01813857	4_EWS-FLI1_loop_and_bridge_target	0.624616878
DDX11-AS1	0.891628218	0.322210237	3_EWS-FLI1_bridge_target	-0.516676979
DDX11	13.22367758	3.605559181	3_EWS-FLI1_bridge_target	-1.626846291
ETFBKMT	1.788926325	0.834533248	4_EWS-FLI1_loop_and_bridge_target	-0.604296772
AMN1	19.74934112	3.491368155	4_EWS-FLI1_loop_and_bridge_target	-2.207838637
TWF1	41.39861366	46.37806988	2_EWS-FLI1_loop_target	0.160202334
NELL2	207.6079148	163.6233243	4_EWS-FLI1_loop_and_bridge_target	-0.341625141
LINC00938	10.57520615	3.204231953	4_EWS-FLI1_loop_and_bridge_target	-1.461123716
ARID2	22.21872531	8.894947719	4_EWS-FLI1_loop_and_bridge_target	-1.230524782
SCAF11	26.17546461	23.86091284	4_EWS-FLI1_loop_and_bridge_target	-0.128425431
SLC38A1	17.07618835	40.06997144	3_EWS-FLI1_bridge_target	1.183993449
SLC38A2	184.7980285	231.5012613	4_EWS-FLI1_loop_and_bridge_target	0.323503349
LOC100288798	4.011640859	6.395688918	4_EWS-FLI1_loop_and_bridge_target	0.56140151
AMIGO2	11.17443098	9.790311456	3_EWS-FLI1_bridge_target	-0.174117836
PCED1B	1.006655645	0.709510938	3_EWS-FLI1_bridge_target	-0.231209408
SP1	28.64326165	21.79508535	3_EWS-FLI1_bridge_target	-0.378981385
ATF7	2.163343158	1.61050926	2_EWS-FLI1_loop_target	-0.277118791
HOXC5	0.38698137	0.42669928	3_EWS-FLI1_bridge_target	0.040732865
HOXC4	2.054798196	1.450522913	3_EWS-FLI1_bridge_target	-0.31798744
HOXC6	4.160002696	3.939603634	3_EWS-FLI1_bridge_target	-0.062976538
CBX5	47.25422671	24.76925274	3_EWS-FLI1_bridge_target	-0.905004615
HNRNPA1	22.96810497	18.30770558	3_EWS-FLI1_bridge_target	-0.311939115
COPZ1	104.2951366	110.4248907	3_EWS-FLI1_bridge_target	0.081632744
LOC102724050	0.930552217	0.907668132	3_EWS-FLI1_bridge_target	-0.017203362
ITGA5	54.58796456	174.5277284	3_EWS-FLI1_bridge_target	1.658854492
NCKAP1L	138.4510681	44.45064968	4_EWS-FLI1_loop_and_bridge_target	-1.617386162
PPP1R1A	49.50921564	1.686788319	4_EWS-FLI1_loop_and_bridge_target	-4.232592071
GLYCAM1	0.849097757	0.045704271	4_EWS-FLI1_loop_and_bridge_target	-0.822346588
LOC100507065	0.167733057	0.227628766	4_EWS-FLI1_loop_and_bridge_target	0.072163844
RPSAP52	4.302953609	2.329745932	4_EWS-FLI1_loop_and_bridge_target	-0.671384027
HMGA2	23.65389639	41.68368392	4_EWS-FLI1_loop_and_bridge_target	0.791869024
LOC100129940	0.645317164	0.59433648	4_EWS-FLI1_loop_and_bridge_target	-0.045409578
LLPH	45.20588191	33.51481321	4_EWS-FLI1_loop_and_bridge_target	-0.420860839
LLPH-AS1	0.593629309	0.362870884	4_EWS-FLI1_loop_and_bridge_target	-0.225667196
TMBIM4	23.76744099	15.06450508	4_EWS-FLI1_loop_and_bridge_target	-0.62456828
YEATS4	39.88965572	21.989347	3_EWS-FLI1_bridge_target	-0.830770428
LOC101928002	0.015074226	0.051568872	3_EWS-FLI1_bridge_target	0.050958116
RAB3IP	2.758406386	3.689860555	3_EWS-FLI1_bridge_target	0.319423957
TSPAN8	53.85516678	7.149536687	4_EWS-FLI1_loop_and_bridge_target	-2.750837566
ZFC3H1	15.899126	10.22612745	4_EWS-FLI1_loop_and_bridge_target	-0.590088291

THAP2	2.052567649	3.626045313	4_EWS-FLI1_loop_and_bridge_target	0.59975613
TMEM19	23.35913444	9.160039982	4_EWS-FLI1_loop_and_bridge_target	-1.261556791
RAB21	9.051452343	19.53406895	4_EWS-FLI1_loop_and_bridge_target	1.030615562
TBC1D15	37.69958431	12.81173729	4_EWS-FLI1_loop_and_bridge_target	-1.486423271
TRHDE-AS1	2.15718923	0.372933211	4_EWS-FLI1_loop_and_bridge_target	-1.201379288
TRHDE	11.03465921	3.328193488	4_EWS-FLI1_loop_and_bridge_target	-1.475358388
ATXN7L3B	31.43865334	29.19974147	4_EWS-FLI1_loop_and_bridge_target	-0.103177729
SYT1	47.65240989	41.25221312	4_EWS-FLI1_loop_and_bridge_target	-0.203484357
ALX1	14.37031905	3.156328257	4_EWS-FLI1_loop_and_bridge_target	-1.886765608
LOC728084	4.4677987	3.523503609	4_EWS-FLI1_loop_and_bridge_target	-0.273519508
DUSP6	105.9477429	117.7392961	4_EWS-FLI1_loop_and_bridge_target	0.15089143
POC1B	12.53801865	14.22414002	4_EWS-FLI1_loop_and_bridge_target	0.169344126
GALNT4	0.203325989	0.540432814	4_EWS-FLI1_loop_and_bridge_target	0.35630823
LOC643339	0.110318297	0.041170745	4_EWS-FLI1_loop_and_bridge_target	-0.092766636
NUDT4	1.006401358	0.691873718	4_EWS-FLI1_loop_and_bridge_target	-0.245988341
USP44	3.963077928	4.660592162	4_EWS-FLI1_loop_and_bridge_target	0.189717876
LOC105369921	2.853009184	0.101819581	4_EWS-FLI1_loop_and_bridge_target	-1.806097618
LOC105369920	0.191291927	0	4_EWS-FLI1_loop_and_bridge_target	-0.25252699
SNRPF	83.15851668	55.35056217	4_EWS-FLI1_loop_and_bridge_target	-0.578679274
CFAP54	0.164266223	0.468295128	3_EWS-FLI1_bridge_target	0.334720995
SLC25A3	200.3760244	180.6597554	3_EWS-FLI1_bridge_target	-0.148653085
FICD	5.036234063	30.25393916	3_EWS-FLI1_bridge_target	2.37231738
SART3	37.87317517	26.9768433	4_EWS-FLI1_loop_and_bridge_target	-0.474541764
ISCU	13.1127682	24.20951175	4_EWS-FLI1_loop_and_bridge_target	0.836967178
SELPLG	0.302329195	1.897580931	4_EWS-FLI1_loop_and_bridge_target	1.153754786
CORO1C	74.14242146	97.1233783	4_EWS-FLI1_loop_and_bridge_target	0.384969298
SSH1	7.301186823	7.680653101	1_EWS-FLI1_in_promoter	0.064485976
ALKBH2	29.04270642	15.96386178	4_EWS-FLI1_loop_and_bridge_target	-0.824550152
UNG	31.77957744	9.06205335	4_EWS-FLI1_loop_and_bridge_target	-1.703872513
ACACB	2.756280409	0.578901004	4_EWS-FLI1_loop_and_bridge_target	-1.250384048
GIT2	13.41310831	7.251829916	4_EWS-FLI1_loop_and_bridge_target	-0.804595509
ANKRD13A	25.57738188	22.41139195	4_EWS-FLI1_loop_and_bridge_target	-0.182988279
IFT81	21.40195114	10.70307773	4_EWS-FLI1_loop_and_bridge_target	-0.936736405
ATP2A2	63.45719695	65.69850103	4_EWS-FLI1_loop_and_bridge_target	0.049312888
ANAPC7	46.97256216	17.95853434	4_EWS-FLI1_loop_and_bridge_target	-1.339362059
ARPC3	183.677719	175.5911104	4_EWS-FLI1_loop_and_bridge_target	-0.064597099
GPN3	42.74066039	14.79873559	4_EWS-FLI1_loop_and_bridge_target	-1.469165901
FAM216A	19.72029862	14.19039998	4_EWS-FLI1_loop_and_bridge_target	-0.447884937
VPS29	72.02170815	37.6254207	4_EWS-FLI1_loop_and_bridge_target	-0.918774771
RAD9B	0.529091929	0.227232146	4_EWS-FLI1_loop_and_bridge_target	-0.317266966
PPTC7	19.24147721	11.93644032	4_EWS-FLI1_loop_and_bridge_target	-0.645873891
TCTN1	6.883142089	7.173504063	4_EWS-FLI1_loop_and_bridge_target	0.05218393
CCDC63	1.03020573	0	2_EWS-FLI1_loop_target	-1.021625929
CUX2	2.455900644	0.565014936	4_EWS-FLI1_loop_and_bridge_target	-1.142885315
SH2B3	62.90112512	48.12509116	1_EWS-FLI1_in_promoter	-0.379381248
ATXN2	6.409767879	4.802179136	4_EWS-FLI1_loop_and_bridge_target	-0.352833511
BRAP	15.60605928	15.60110092	4_EWS-FLI1_loop_and_bridge_target	-0.000430835
ACAD10	11.4175865	8.914343433	4_EWS-FLI1_loop_and_bridge_target	-0.324795657
MAPKAPK5-AS1	14.6615286	5.639347027	4_EWS-FLI1_loop_and_bridge_target	-1.238111764
MAPKAPK5	25.1883012	22.73721332	4_EWS-FLI1_loop_and_bridge_target	-0.141771901
TMEM116	8.947977697	5.746508525	4_EWS-FLI1_loop_and_bridge_target	-0.560262206
ERP29	74.05631315	80.10366977	4_EWS-FLI1_loop_and_bridge_target	0.11179377
NAA25	22.63023934	19.90759406	4_EWS-FLI1_loop_and_bridge_target	-0.176607189
TRAFD1	33.46054242	28.07844807	3_EWS-FLI1_bridge_target	-0.244995136
HECTD4	7.779617754	5.216789064	4_EWS-FLI1_loop_and_bridge_target	-0.4979885
TPCN1	3.650653816	8.936273238	4_EWS-FLI1_loop_and_bridge_target	1.095271294

PLBD2	4.593167745	7.184382224	3_EWS-FLI1_bridge_target	0.549207924
WSB2	59.64669402	50.65847217	3_EWS-FLI1_bridge_target	-0.231424028
TMEM233	5.415681603	8.827316568	3_EWS-FLI1_bridge_target	0.615194985
GCN1	36.51936793	37.16654329	3_EWS-FLI1_bridge_target	0.024673005
RPLP0	511.9076902	629.0026354	3_EWS-FLI1_bridge_target	0.296658661
PXN-AS1	3.50994362	0.592578141	3_EWS-FLI1_bridge_target	-1.501745237
PXN	20.05479883	14.64715657	3_EWS-FLI1_bridge_target	-0.428248579
SIRT4	2.729730532	1.475001483	3_EWS-FLI1_bridge_target	-0.591642012
LINC01089	6.917196581	2.792708265	3_EWS-FLI1_bridge_target	-1.06176127
SETD1B	0.631804128	0.641741137	3_EWS-FLI1_bridge_target	0.008758772
UBC	124.3804531	169.223468	4_EWS-FLI1_loop_and_bridge_target	0.4411175
LOC101927464	0.199262424	0	1_EWS-FLI1_in_promoter	-0.262147386
LINC00944	8.625078846	15.65370066	4_EWS-FLI1_loop_and_bridge_target	0.790972533
TPTE2	10.99287405	0.50332806	4_EWS-FLI1_loop_and_bridge_target	-2.995945659
MPHOSPH8	20.78753418	20.15024363	4_EWS-FLI1_loop_and_bridge_target	-0.042828646
SAP18	88.96474045	75.14934907	4_EWS-FLI1_loop_and_bridge_target	-0.240527978
SKA3	30.87932721	2.563912022	4_EWS-FLI1_loop_and_bridge_target	-3.161087554
MRPL57	18.99229832	12.42267916	4_EWS-FLI1_loop_and_bridge_target	-0.574771673
LINC01046	0.787209577	0.117514521	4_EWS-FLI1_loop_and_bridge_target	-0.677415244
MIPEPP3	3.707349931	3.829352509	4_EWS-FLI1_loop_and_bridge_target	0.036914671
SPATA13	19.53413706	8.227063602	1_EWS-FLI1_in_promoter	-1.154080814
PARP4	46.00240313	45.92904874	4_EWS-FLI1_loop_and_bridge_target	-0.002253304
AMER2	42.79551851	2.967574098	4_EWS-FLI1_loop_and_bridge_target	-3.464454178
LINC00463	6.465536405	0.121977445	4_EWS-FLI1_loop_and_bridge_target	-2.734202249
LINC01053	0.617186485	0	4_EWS-FLI1_loop_and_bridge_target	-0.693486052
MTMR6	38.11897055	25.04038031	4_EWS-FLI1_loop_and_bridge_target	-0.587117886
NUP58	52.3938366	21.51034189	3_EWS-FLI1_bridge_target	-1.246085248
UBL3	13.39835772	18.29103657	4_EWS-FLI1_loop_and_bridge_target	0.422026399
KATNAL1	25.62199344	10.92866662	4_EWS-FLI1_loop_and_bridge_target	-1.158185816
LINC01058	2.836603034	0.266878773	4_EWS-FLI1_loop_and_bridge_target	-1.598551019
HMGB1	96.86849263	27.57436534	4_EWS-FLI1_loop_and_bridge_target	-1.776123024
USPL1	14.27601273	12.29458787	4_EWS-FLI1_loop_and_bridge_target	-0.200428973
LINC00398	0.056303731	0	4_EWS-FLI1_loop_and_bridge_target	-0.079024729
HSPH1	86.45491038	36.03783788	4_EWS-FLI1_loop_and_bridge_target	-1.239539508
BRCA2	13.9377993	3.003323765	4_EWS-FLI1_loop_and_bridge_target	-1.899697418
N4BP2L2	21.06721213	16.51447093	4_EWS-FLI1_loop_and_bridge_target	-0.333356969
PDS5B	18.4080424	9.183212088	4_EWS-FLI1_loop_and_bridge_target	-0.930461905
STARD13	25.04425764	16.09496875	1_EWS-FLI1_in_promoter	-0.60739353
STARD13-AS	1.364252505	1.494210805	4_EWS-FLI1_loop_and_bridge_target	0.077199278
RFC3	29.01316537	3.858137096	4_EWS-FLI1_loop_and_bridge_target	-2.627120374
CCDC169	1.392117989	0.533454078	4_EWS-FLI1_loop_and_bridge_target	-0.641503588
SPG20	56.30376788	49.19894104	4_EWS-FLI1_loop_and_bridge_target	-0.190973073
SPG20-AS1	1.47715774	0.888164771	4_EWS-FLI1_loop_and_bridge_target	-0.391701072
RFXAP	5.019861313	2.202773306	4_EWS-FLI1_loop_and_bridge_target	-0.910408563
SMAD9	10.64101255	4.734230738	4_EWS-FLI1_loop_and_bridge_target	-1.021544688
ALG5	58.19185586	77.81498206	4_EWS-FLI1_loop_and_bridge_target	0.413071209
EXOSC8	38.17704232	28.51049288	4_EWS-FLI1_loop_and_bridge_target	-0.408780469
SUPT20H	26.36056643	14.2880693	4_EWS-FLI1_loop_and_bridge_target	-0.839691875
TRPC4	24.19372641	5.140664398	4_EWS-FLI1_loop_and_bridge_target	-2.036597864
UFM1	53.92641408	120.8762951	4_EWS-FLI1_loop_and_bridge_target	1.149845537
LHFP	57.26938731	37.0238131	4_EWS-FLI1_loop_and_bridge_target	-0.615834926
TSC22D1	4.46159736	14.66234144	3_EWS-FLI1_bridge_target	1.51990504
GTF2F2	56.56694064	49.10831159	4_EWS-FLI1_loop_and_bridge_target	-0.200190616
TPT1	137.560115	178.5987601	4_EWS-FLI1_loop_and_bridge_target	0.374265357
SNORA31	2.256263758	2.379669059	4_EWS-FLI1_loop_and_bridge_target	0.053664421
TPT1-AS1	2.248035318	1.738749018	4_EWS-FLI1_loop_and_bridge_target	-0.246050258

SLC25A30	14.9913177	5.337378298	4_EWS-FLI1_loop_and_bridge_target	-1.335330781
SLC25A30-AS1	0.163730608	0.050920165	4_EWS-FLI1_loop_and_bridge_target	-0.147104051
COG3	17.20905908	31.67455498	4_EWS-FLI1_loop_and_bridge_target	0.843511212
SIAH3	6.274221951	0.598790036	4_EWS-FLI1_loop_and_bridge_target	-2.185812459
ZC3H13	60.04217265	18.54998616	4_EWS-FLI1_loop_and_bridge_target	-1.642638726
CPB2-AS1	2.988254736	0.630870076	4_EWS-FLI1_loop_and_bridge_target	-1.290115707
CPB2	6.253635674	0.768069855	1_EWS-FLI1_in_promoter	-2.03652901
LCP1	35.64725906	40.96295764	4_EWS-FLI1_loop_and_bridge_target	0.195411066
LRRC63	1.906283476	0.478410289	4_EWS-FLI1_loop_and_bridge_target	-0.975128727
LINC00563	0.74694943	0.807387717	4_EWS-FLI1_loop_and_bridge_target	0.049068176
KIAA0226L	38.43359277	8.224329216	1_EWS-FLI1_in_promoter	-2.095909251
LINC01198	1.037700676	0.025478255	4_EWS-FLI1_loop_and_bridge_target	-0.990645245
LRCH1	10.69202424	7.444930978	4_EWS-FLI1_loop_and_bridge_target	-0.469367189
INTS6	20.26307957	21.37091385	4_EWS-FLI1_loop_and_bridge_target	0.073273631
INTS6-AS1	2.851959271	1.950300563	4_EWS-FLI1_loop_and_bridge_target	-0.384730512
WDFY2	15.24710662	32.73060174	4_EWS-FLI1_loop_and_bridge_target	1.053875238
DHRS12	1.86771775	2.581756824	4_EWS-FLI1_loop_and_bridge_target	0.320764356
NEK3	8.046295405	3.864568626	2_EWS-FLI1_loop_target	-0.89501523
PCDH17	16.55522677	5.851864418	4_EWS-FLI1_loop_and_bridge_target	-1.357332123
DIAPH3	34.37893453	14.14462691	4_EWS-FLI1_loop_and_bridge_target	-1.224084563
DIAPH3-AS1	0.494496139	0.157835193	4_EWS-FLI1_loop_and_bridge_target	-0.368229256
TDRD3	19.88330286	16.49534705	4_EWS-FLI1_loop_and_bridge_target	-0.255378621
KLF12	3.88697808	2.971041579	3_EWS-FLI1_bridge_target	-0.299425167
MYCBP2	14.48598701	14.30731477	4_EWS-FLI1_loop_and_bridge_target	-0.016742111
SLAIN1	77.99447004	23.69270167	1_EWS-FLI1_in_promoter	-1.677666969
RNF219	13.4348207	3.743904915	2_EWS-FLI1_loop_target	-1.605406189
SPRY2	8.235949374	33.19338553	4_EWS-FLI1_loop_and_bridge_target	1.888385103
GPC6	11.38988731	23.34434573	4_EWS-FLI1_loop_and_bridge_target	0.974423662
FARP1	33.9367781	15.07123738	4_EWS-FLI1_loop_and_bridge_target	-1.120265557
UBAC2-AS1	1.127512366	0.561920755	3_EWS-FLI1_bridge_target	-0.445846258
UBAC2	18.49944884	19.82937764	3_EWS-FLI1_bridge_target	0.095186387
LINC01232	1.003659717	0.766156522	4_EWS-FLI1_loop_and_bridge_target	-0.182024311
LINC00449	0.992043817	0.710949606	4_EWS-FLI1_loop_and_bridge_target	-0.219452113
TM9SF2	91.0727351	110.9812657	4_EWS-FLI1_loop_and_bridge_target	0.282411483
LOC101927437	0.093943242	0.087648952	4_EWS-FLI1_loop_and_bridge_target	-0.008324898
ZIC5	12.49202449	9.462769286	4_EWS-FLI1_loop_and_bridge_target	-0.366842087
ZIC2	22.74927189	7.338979368	4_EWS-FLI1_loop_and_bridge_target	-1.50994056
LINC00554	0.114393844	0.039529386	4_EWS-FLI1_loop_and_bridge_target	-0.100328651
LOC105370333	10.88036094	2.101096689	4_EWS-FLI1_loop_and_bridge_target	-1.937728254
PCCA	36.67672053	25.4440824	4_EWS-FLI1_loop_and_bridge_target	-0.51072848
GGACT	2.288737952	1.073843082	2_EWS-FLI1_loop_target	-0.665227322
TMTC4	11.07566975	8.13363448	2_EWS-FLI1_loop_target	-0.402842246
FGF14	3.299033432	1.178511575	4_EWS-FLI1_loop_and_bridge_target	-0.980669551
FGF14-IT1	0.395157033	0.134065863	4_EWS-FLI1_loop_and_bridge_target	-0.298923085
FGF14-AS2	4.951883224	0.88604699	4_EWS-FLI1_loop_and_bridge_target	-1.6579806
TPP2	33.14914459	36.74593008	4_EWS-FLI1_loop_and_bridge_target	0.144471655
ERCC5	0.615006278	0.774775973	4_EWS-FLI1_loop_and_bridge_target	0.136097154
TNFSF13B	1.838197729	5.973673172	4_EWS-FLI1_loop_and_bridge_target	1.296943651
LINC00399	0.061906773	0	3_EWS-FLI1_bridge_target	-0.086657114
IRS2	17.33235706	12.13978071	4_EWS-FLI1_loop_and_bridge_target	-0.480451091
SOX1	9.415586099	0.590862327	3_EWS-FLI1_bridge_target	-2.710863128
OSGEP	25.28584983	16.20371578	3_EWS-FLI1_bridge_target	-0.611566177
APEX1	141.3893283	58.68726657	3_EWS-FLI1_bridge_target	-1.254345934
TMEM55B	20.90271456	20.39072487	3_EWS-FLI1_bridge_target	-0.034124313
METTL17	31.66777519	19.45972375	3_EWS-FLI1_bridge_target	-0.675081539
NDRG2	3.246967429	0.654546069	3_EWS-FLI1_bridge_target	-1.359997582

ARHGEF40	12.89981986	13.23429986	3_EWS-FLI1_bridge_target	0.034305347
ZNF219	2.933362887	1.956829646	4_EWS-FLI1_loop_and_bridge_target	-0.411712167
TMEM253	0.860771888	0.717331433	4_EWS-FLI1_loop_and_bridge_target	-0.115732709
HNRNPC	153.8063196	112.1284543	4_EWS-FLI1_loop_and_bridge_target	-0.452502523
SUPT16H	82.14989071	64.04886386	4_EWS-FLI1_loop_and_bridge_target	-0.354190508
CHD8	43.1583918	26.33447437	4_EWS-FLI1_loop_and_bridge_target	-0.691965998
RAB2B	17.94822102	9.896791541	4_EWS-FLI1_loop_and_bridge_target	-0.798158996
TOX4	31.96651812	21.76193159	4_EWS-FLI1_loop_and_bridge_target	-0.534378529
METTL3	48.4694514	25.91509035	4_EWS-FLI1_loop_and_bridge_target	-0.878122632
SALL2	31.49751794	5.053525496	4_EWS-FLI1_loop_and_bridge_target	-2.424482035
OR4E1	1.037750058	0.645479853	4_EWS-FLI1_loop_and_bridge_target	-0.308468744
DAD1	204.6851067	264.5632072	4_EWS-FLI1_loop_and_bridge_target	0.368617946
ABHD4	9.926747282	13.89958153	4_EWS-FLI1_loop_and_bridge_target	0.447407814
OXA1L	42.98411583	26.862989	4_EWS-FLI1_loop_and_bridge_target	-0.65863258
MRPL52	36.66916098	32.84780189	3_EWS-FLI1_bridge_target	-0.154321753
MMP14	32.84004428	114.3552152	4_EWS-FLI1_loop_and_bridge_target	1.769279868
LRP10	6.906129067	21.90027741	4_EWS-FLI1_loop_and_bridge_target	1.534321662
RBM23	57.59326843	35.51226561	4_EWS-FLI1_loop_and_bridge_target	-0.682353736
PRMT5-AS1	0.339428008	0.316685997	4_EWS-FLI1_loop_and_bridge_target	-0.024705708
PRMT5	89.87893127	34.11222849	4_EWS-FLI1_loop_and_bridge_target	-1.371972305
LOC101926933	0.48167032	0.192599115	4_EWS-FLI1_loop_and_bridge_target	-0.313115305
HAUS4	44.01972148	6.573872096	3_EWS-FLI1_bridge_target	-2.571454168
AJUBA	19.31565145	17.47440708	4_EWS-FLI1_loop_and_bridge_target	-0.137063564
C14orf93	5.280374217	2.18375253	4_EWS-FLI1_loop_and_bridge_target	-0.980122324
PSMB5	217.5430386	173.2870426	4_EWS-FLI1_loop_and_bridge_target	-0.326452107
CDH24	13.98392623	5.843684926	4_EWS-FLI1_loop_and_bridge_target	-1.130570455
ACIN1	54.78519465	33.06226962	3_EWS-FLI1_bridge_target	-0.711707716
C14orf119	26.89995364	23.0048143	3_EWS-FLI1_bridge_target	-0.216938949
PPP1R3E	1.68524145	0.566461823	3_EWS-FLI1_bridge_target	-0.777542208
PRKD1	15.84995209	9.290222831	4_EWS-FLI1_loop_and_bridge_target	-0.711470266
NPAS3	4.520166864	2.65048367	1_EWS-FLI1_in_promoter	-0.596624251
LRFN5	28.57760607	10.03006278	4_EWS-FLI1_loop_and_bridge_target	-1.423064287
C14orf28	1.522787308	2.952067089	2_EWS-FLI1_loop_target	0.647588857
SAMD4A	17.60359597	30.41831972	3_EWS-FLI1_bridge_target	0.756024513
WDHD1	24.49923578	2.386770194	3_EWS-FLI1_bridge_target	-2.912472007
SOCS4	21.73358558	13.32370326	3_EWS-FLI1_bridge_target	-0.66642071
MAPK1IP1L	18.11492265	10.34046951	4_EWS-FLI1_loop_and_bridge_target	-0.753218992
ATG14	10.22267071	7.739067097	3_EWS-FLI1_bridge_target	-0.360864857
KTN1-AS1	2.479710168	0.991528775	3_EWS-FLI1_bridge_target	-0.805090822
KTN1	102.372784	142.8455324	3_EWS-FLI1_bridge_target	0.476664011
LOC101927690	0.045832148	0.021380674	4_EWS-FLI1_loop_and_bridge_target	-0.034130656
TMEM260	8.190746686	9.070988087	4_EWS-FLI1_loop_and_bridge_target	0.131951256
OTX2	75.18260027	7.439504824	1_EWS-FLI1_in_promoter	-3.174231272
OTX2-AS1	1.119219352	0.029414945	1_EWS-FLI1_in_promoter	-1.04170829
EXOC5	22.84744689	31.50524141	4_EWS-FLI1_loop_and_bridge_target	0.446837549
AP5M1	35.77787409	28.64829375	4_EWS-FLI1_loop_and_bridge_target	-0.310889008
NAA30	26.69680111	14.59707613	4_EWS-FLI1_loop_and_bridge_target	-0.828443757
RHOJ	3.463939661	4.241189675	4_EWS-FLI1_loop_and_bridge_target	0.231576794
WDR89	16.00187062	12.826585	2_EWS-FLI1_loop_target	-0.298248614
GPHN	2.276649623	2.104751458	3_EWS-FLI1_bridge_target	-0.077743629
FAM71D	0.092618065	0.026588481	4_EWS-FLI1_loop_and_bridge_target	-0.089931204
MPP5	87.76415408	37.37327967	4_EWS-FLI1_loop_and_bridge_target	-1.209875112
ATP6V1D	85.97191637	54.86020005	4_EWS-FLI1_loop_and_bridge_target	-0.638728883
EIF2S1	74.81435696	65.50231916	4_EWS-FLI1_loop_and_bridge_target	-0.189066424
PLEK2	9.098581473	15.97427556	4_EWS-FLI1_loop_and_bridge_target	0.749197349
RDH11	102.5839613	111.4716372	4_EWS-FLI1_loop_and_bridge_target	0.118760595

ZFP36L1	5.505167016	6.205399391	4_EWS-FLI1_loop_and_bridge_target	0.147492303
MED6	20.62593748	20.4303536	4_EWS-FLI1_loop_and_bridge_target	-0.01310702
PCNX1	28.37025564	24.70110851	4_EWS-FLI1_loop_and_bridge_target	-0.192525238
SIPA1L1	18.10200103	12.51343875	4_EWS-FLI1_loop_and_bridge_target	-0.499328934
DPF3	7.609261463	1.761746579	4_EWS-FLI1_loop_and_bridge_target	-1.640308541
RBM25	51.64244841	31.85150813	3_EWS-FLI1_bridge_target	-0.680266988
FLRT2	15.31969709	16.40310954	4_EWS-FLI1_loop_and_bridge_target	0.092730827
LOC283585	0.028934306	0	4_EWS-FLI1_loop_and_bridge_target	-0.041150873
PSMC1	56.44679821	44.09739827	3_EWS-FLI1_bridge_target	-0.349182282
CALM1	204.5541151	123.5935026	4_EWS-FLI1_loop_and_bridge_target	-0.722289418
LOC105370622	0.254533782	0.237479768	4_EWS-FLI1_loop_and_bridge_target	-0.019746382
MOAP1	32.55999126	20.4147447	4_EWS-FLI1_loop_and_bridge_target	-0.648137862
TMEM251	17.1160863	7.093580309	4_EWS-FLI1_loop_and_bridge_target	-1.162421371
BTBD7	10.84539974	14.8589606	4_EWS-FLI1_loop_and_bridge_target	0.420971334
UNC79	0.302598492	0.148887333	4_EWS-FLI1_loop_and_bridge_target	-0.181155136
LOC730202	1.183249035	0.64836344	4_EWS-FLI1_loop_and_bridge_target	-0.405442331
PAPOLA	36.58324964	35.88521495	4_EWS-FLI1_loop_and_bridge_target	-0.027047172
VRK1	89.6889181	24.76085481	4_EWS-FLI1_loop_and_bridge_target	-1.815745803
LOC101929241	0.689559534	0.264912268	4_EWS-FLI1_loop_and_bridge_target	-0.417609861
LINC01550	3.533696324	1.028252063	4_EWS-FLI1_loop_and_bridge_target	-1.160450804
BCL11B	23.20016146	4.173222937	4_EWS-FLI1_loop_and_bridge_target	-2.225881403
PPP2R5C	34.66430078	29.06410105	3_EWS-FLI1_bridge_target	-0.246438868
DYNC1H1	106.2741822	99.17113156	4_EWS-FLI1_loop_and_bridge_target	-0.098836107
HSP90AA1	253.7630939	126.5360109	3_EWS-FLI1_bridge_target	-0.998251636
WDR20	6.48800063	6.814215592	3_EWS-FLI1_bridge_target	0.061520503
ZNF839	5.261083435	3.966115099	3_EWS-FLI1_bridge_target	-0.334294628
ANKRD9	5.043079509	2.312347549	4_EWS-FLI1_loop_and_bridge_target	-0.867429868
RCOR1	39.35287482	14.30033926	4_EWS-FLI1_loop_and_bridge_target	-1.399107815
TRAF3	11.61298128	16.10163163	4_EWS-FLI1_loop_and_bridge_target	0.439224656
CDC42BPB	24.05541422	24.20863151	4_EWS-FLI1_loop_and_bridge_target	0.008795413
EIF5	80.95960144	69.00243127	3_EWS-FLI1_bridge_target	-0.227507939
BAG5	25.17548369	20.36125287	3_EWS-FLI1_bridge_target	-0.293219931
APOPT1	8.233959056	9.208783643	3_EWS-FLI1_bridge_target	0.144789742
MEIS2	1.865911005	1.872738201	3_EWS-FLI1_bridge_target	0.003432713
GPR176	10.56149234	36.80817117	4_EWS-FLI1_loop_and_bridge_target	1.709370435
EIF2AK4	31.55014789	44.7273721	4_EWS-FLI1_loop_and_bridge_target	0.490393913
SRP14	132.1427342	109.091693	4_EWS-FLI1_loop_and_bridge_target	-0.274268086
SRP14-AS1	1.870666036	0.860121347	4_EWS-FLI1_loop_and_bridge_target	-0.625988762
BMF	13.5855436	14.68989931	4_EWS-FLI1_loop_and_bridge_target	0.105296938
BUB1B	78.03674596	5.767931152	4_EWS-FLI1_loop_and_bridge_target	-3.545736753
ANKRD63	3.049778769	0.395200081	4_EWS-FLI1_loop_and_bridge_target	-1.53737107
INAFM2	12.29733818	27.0575529	4_EWS-FLI1_loop_and_bridge_target	1.077251706
C15orf52	1.165874083	4.232657094	4_EWS-FLI1_loop_and_bridge_target	1.272594348
CHAC1	5.54256097	21.07380004	3_EWS-FLI1_bridge_target	1.754407644
SERF2	30.09659656	39.03566685	4_EWS-FLI1_loop_and_bridge_target	0.364529147
SERINC4	1.413623447	1.933774462	4_EWS-FLI1_loop_and_bridge_target	0.28155735
HYPK	0	0.062481933	4_EWS-FLI1_loop_and_bridge_target	0.08743831
CTDSPL2	37.09666181	13.37526341	4_EWS-FLI1_loop_and_bridge_target	-1.406076196
EIF3J-AS1	3.63086064	2.416951827	4_EWS-FLI1_loop_and_bridge_target	-0.438570433
EIF3J	74.95133788	63.66405476	4_EWS-FLI1_loop_and_bridge_target	-0.232111403
SPG11	18.68215715	14.3061255	4_EWS-FLI1_loop_and_bridge_target	-0.362779214
B2M	783.1146518	713.7922223	4_EWS-FLI1_loop_and_bridge_target	-0.133540682
LOC100419583	46.23043461	27.88638474	4_EWS-FLI1_loop_and_bridge_target	-0.709327156
TRIM69	2.635978066	5.497770224	4_EWS-FLI1_loop_and_bridge_target	0.837601231
SORD	79.57515346	20.49570039	4_EWS-FLI1_loop_and_bridge_target	-1.906286913
SHF	7.692617043	2.097011739	4_EWS-FLI1_loop_and_bridge_target	-1.488913736

BLOC1S6	23.19303844	9.236769711	4_EWS-FLI1_loop_and_bridge_target	-1.240831437
SEMA6D	5.581982186	7.090449482	4_EWS-FLI1_loop_and_bridge_target	0.297697735
TRPM7	27.73754406	24.82530993	3_EWS-FLI1_bridge_target	-0.15415111
AP4E1	9.791183184	6.942163424	3_EWS-FLI1_bridge_target	-0.442249103
MNS1	27.71195625	1.617422124	4_EWS-FLI1_loop_and_bridge_target	-3.455433111
ZNF280D	10.32860672	8.877794063	4_EWS-FLI1_loop_and_bridge_target	-0.197709642
LOC145783	4.768032128	0.78480965	4_EWS-FLI1_loop_and_bridge_target	-1.692308981
TCF12	122.1266167	54.49817211	4_EWS-FLI1_loop_and_bridge_target	-1.149630508
FAM96A	19.32346817	12.74637752	4_EWS-FLI1_loop_and_bridge_target	-0.56409513
SNX1	16.3478104	10.20267011	4_EWS-FLI1_loop_and_bridge_target	-0.630910947
ANP32A	67.4150193	31.7964702	2_EWS-FLI1_loop_target	-1.060772528
EWSAT1	7.618356248	0.249648575	1_EWS-FLI1_in_promoter	-2.785890296
GLCE	31.41248375	10.67677303	4_EWS-FLI1_loop_and_bridge_target	-1.472907949
LOC145694	0.619959433	0.381329756	4_EWS-FLI1_loop_and_bridge_target	-0.22989992
KIF23	64.61690243	8.024911373	4_EWS-FLI1_loop_and_bridge_target	-2.862082821
RPLP1	359.3199665	436.2881535	4_EWS-FLI1_loop_and_bridge_target	0.279305667
TLE3	8.154452263	7.046397384	4_EWS-FLI1_loop_and_bridge_target	-0.186130579
LINGO1	4.774794933	1.435743848	2_EWS-FLI1_loop_target	-1.245407294
LINGO1-AS2	0.172854151	0	2_EWS-FLI1_loop_target	-0.23002362
LINC00924	0.038011312	0.008866129	4_EWS-FLI1_loop_and_bridge_target	-0.041087416
NR2F2-AS1	0.59735315	0.511965838	4_EWS-FLI1_loop_and_bridge_target	-0.079257762
NR2F2	7.595322677	2.880139557	4_EWS-FLI1_loop_and_bridge_target	-1.147443258
TPSAB1	4.688841768	0.308918513	1_EWS-FLI1_in_promoter	-2.119759669
UBE2I	43.49667897	22.1476587	2_EWS-FLI1_loop_target	-0.942831387
TMEM114	0.952208705	0.126915683	1_EWS-FLI1_in_promoter	-0.792727719
METTL22	26.24684652	11.46703028	4_EWS-FLI1_loop_and_bridge_target	-1.127971418
TMEM186	9.5910179	6.146382792	4_EWS-FLI1_loop_and_bridge_target	-0.567556155
PMM2	34.62414077	43.24994735	4_EWS-FLI1_loop_and_bridge_target	0.312820521
CARHSP1	49.49633635	18.14329783	4_EWS-FLI1_loop_and_bridge_target	-1.399339335
C16orf72	37.6782759	42.06733391	4_EWS-FLI1_loop_and_bridge_target	0.155070529
USP31	13.27849927	10.51587725	4_EWS-FLI1_loop_and_bridge_target	-0.310220038
SCNN1G	57.13062704	2.217125217	1_EWS-FLI1_in_promoter	-4.175454476
COG7	18.72320487	13.15826542	4_EWS-FLI1_loop_and_bridge_target	-0.478249471
GGA2	38.73450689	23.34051764	4_EWS-FLI1_loop_and_bridge_target	-0.707032591
PALB2	13.86842903	9.142198284	4_EWS-FLI1_loop_and_bridge_target	-0.551881838
DCTN5	18.52102747	20.15733934	4_EWS-FLI1_loop_and_bridge_target	0.116129222
PLK1	52.11451861	3.365188569	4_EWS-FLI1_loop_and_bridge_target	-3.604990387
PRKCB	68.84186062	12.80252452	4_EWS-FLI1_loop_and_bridge_target	-2.33915983
CACNG3	1.339916324	0.167272335	4_EWS-FLI1_loop_and_bridge_target	-1.003315746
RBBP6	17.4489733	17.33426242	4_EWS-FLI1_loop_and_bridge_target	-0.008998303
ARHGAP17	21.14441784	12.10179378	4_EWS-FLI1_loop_and_bridge_target	-0.757178724
ZKSCAN2	7.518171826	4.541608084	4_EWS-FLI1_loop_and_bridge_target	-0.620239148
HS3ST4	16.80199673	2.27714195	4_EWS-FLI1_loop_and_bridge_target	-2.441528998
SALL1	6.588151077	2.064451703	4_EWS-FLI1_loop_and_bridge_target	-1.308119435
LINC01571	0.437738964	0.108909339	4_EWS-FLI1_loop_and_bridge_target	-0.374660344
LOC105371267	0.577173918	0.179500899	4_EWS-FLI1_loop_and_bridge_target	-0.41916524
CHD9	26.36870302	18.2175968	4_EWS-FLI1_loop_and_bridge_target	-0.510099133
RBL2	21.28000702	17.15575368	2_EWS-FLI1_loop_target	-0.295322866
AKTIP	5.911689986	5.174429735	3_EWS-FLI1_bridge_target	-0.162732612
IRX3	3.928612371	2.015707544	4_EWS-FLI1_loop_and_bridge_target	-0.708684993
CRNDE	20.58408908	10.39848346	4_EWS-FLI1_loop_and_bridge_target	-0.921126317
IRX5	3.13562907	1.834953348	4_EWS-FLI1_loop_and_bridge_target	-0.5447818
CES1P1	0.119185001	0.052952138	4_EWS-FLI1_loop_and_bridge_target	-0.088008674
CES1	304.3176942	122.9160572	1_EWS-FLI1_in_promoter	-1.300948056
ADGRG1	0.177898697	5.882780925	4_EWS-FLI1_loop_and_bridge_target	2.546776123
KATNB1	20.16541758	11.38122145	4_EWS-FLI1_loop_and_bridge_target	-0.773555304

KIFC3	24.92380666	22.40091001	4_EWS-FLI1_loop_and_bridge_target	-0.147712946
LOC388282	1.632436928	1.006144017	4_EWS-FLI1_loop_and_bridge_target	-0.391973788
ZNF319	2.89740122	2.517228702	4_EWS-FLI1_loop_and_bridge_target	-0.148073311
USB1	4.595222055	1.836376846	4_EWS-FLI1_loop_and_bridge_target	-0.980146163
CFAP20	43.20619122	32.89924984	4_EWS-FLI1_loop_and_bridge_target	-0.38299509
CDH8	18.01667102	2.895296059	4_EWS-FLI1_loop_and_bridge_target	-2.287459827
CDH11	67.88202776	63.12060237	4_EWS-FLI1_loop_and_bridge_target	-0.103339635
LOC101927650	0.034007454	0.142780144	4_EWS-FLI1_loop_and_bridge_target	0.144301288
TK2	4.442802026	5.042789004	2_EWS-FLI1_loop_target	0.150865007
CKLF	5.902476134	2.706605574	2_EWS-FLI1_loop_target	-0.897015391
CMTM3	6.848359154	20.062977	3_EWS-FLI1_bridge_target	1.42424639
PDP2	7.678887891	4.439453729	3_EWS-FLI1_bridge_target	-0.674048416
FAM96B	64.12194895	53.13202613	3_EWS-FLI1_bridge_target	-0.266661488
CES2	10.61367534	9.095737197	3_EWS-FLI1_bridge_target	-0.202078348
NQO1	9.216550319	12.62247434	3_EWS-FLI1_bridge_target	0.41508063
PDXDC2P	5.922029695	9.428770042	4_EWS-FLI1_loop_and_bridge_target	0.591301982
PDPR	11.39611482	6.798327517	4_EWS-FLI1_loop_and_bridge_target	-0.668651371
AARS	162.338811	103.373991	4_EWS-FLI1_loop_and_bridge_target	-0.64610538
DDX19B	10.0398981	8.503174576	4_EWS-FLI1_loop_and_bridge_target	-0.216245419
SNORD111B	2.272775373	0.706832394	3_EWS-FLI1_bridge_target	-0.93919319
PSMD7	118.471176	91.99487094	4_EWS-FLI1_loop_and_bridge_target	-0.361439538
GLG1	126.2321335	73.92909143	4_EWS-FLI1_loop_and_bridge_target	-0.763865218
RFWD3	54.26376144	12.90749413	4_EWS-FLI1_loop_and_bridge_target	-1.990471263
MLKL	7.458148632	7.989010658	4_EWS-FLI1_loop_and_bridge_target	0.087820427
BCAR1	3.244961971	10.18597216	4_EWS-FLI1_loop_and_bridge_target	1.397867111
ADAT1	6.770240192	5.308123833	3_EWS-FLI1_bridge_target	-0.300748214
MYH13	0.34904179	0.007942821	2_EWS-FLI1_loop_target	-0.420521241
MYHAS	0.197441752	0.045298269	3_EWS-FLI1_bridge_target	-0.196040816
SHISA6	8.165916572	7.116542332	4_EWS-FLI1_loop_and_bridge_target	-0.175413888
DNAH9	0.75583573	0.904617081	4_EWS-FLI1_loop_and_bridge_target	0.117343098
ZNF18	11.881153	6.16812222	2_EWS-FLI1_loop_target	-0.845594594
KIAA0100	65.98520907	58.55074101	3_EWS-FLI1_bridge_target	-0.169723111
SDF2	38.5082822	40.83193212	3_EWS-FLI1_bridge_target	0.082449514
SUPT6H	38.27599441	35.51066448	3_EWS-FLI1_bridge_target	-0.105329877
RAB34	74.44011533	66.57111774	3_EWS-FLI1_bridge_target	-0.158925162
RPL23A	177.1455277	186.4605638	3_EWS-FLI1_bridge_target	0.073530861
SNORD42B	16.17891922	12.78628151	3_EWS-FLI1_bridge_target	-0.317405895
SNORD4A	44.10341656	26.27551253	3_EWS-FLI1_bridge_target	-0.725630415
SNORD42A	6.477610236	4.53270297	3_EWS-FLI1_bridge_target	-0.434592801
SNORD4B	13.95914064	8.843364748	3_EWS-FLI1_bridge_target	-0.603803839
TLCD1	6.030143739	4.512822694	3_EWS-FLI1_bridge_target	-0.350762986
NEK8	1.606370278	3.139031614	3_EWS-FLI1_bridge_target	0.667251211
ERAL1	42.62083164	22.45453727	3_EWS-FLI1_bridge_target	-0.895150237
FLOT2	32.53610161	23.69271569	4_EWS-FLI1_loop_and_bridge_target	-0.441629481
DHRS13	19.56402255	3.183628049	4_EWS-FLI1_loop_and_bridge_target	-2.297296001
PHF12	6.598766362	5.079434472	3_EWS-FLI1_bridge_target	-0.321828094
LOC101927018	0.766663638	0.697850164	3_EWS-FLI1_bridge_target	-0.05731824
PIPOX	6.741216342	2.614478859	3_EWS-FLI1_bridge_target	-1.098772616
NUFIP2	21.3949237	30.40858358	3_EWS-FLI1_bridge_target	0.487987133
TAOK1	27.62947275	26.13972209	3_EWS-FLI1_bridge_target	-0.077095154
ABHD15	3.774523274	1.925723248	3_EWS-FLI1_bridge_target	-0.706563383
TP53I13	8.750541477	6.845847673	3_EWS-FLI1_bridge_target	-0.313553013
GIT1	20.03816855	19.2810154	4_EWS-FLI1_loop_and_bridge_target	-0.052879234
ANKRD13B	9.489237111	7.514744398	4_EWS-FLI1_loop_and_bridge_target	-0.300874626
SSH2	13.19087799	3.108255572	4_EWS-FLI1_loop_and_bridge_target	-1.788366013
EFCAB5	0.197156322	0.10698938	3_EWS-FLI1_bridge_target	-0.112970166

NSRP1	19.11651599	13.49740721	4_EWS-FLI1_loop_and_bridge_target	-0.47258556
TMEM98	33.62721507	9.521225428	3_EWS-FLI1_bridge_target	-1.718603614
ASIC2	2.962016575	0.963902475	4_EWS-FLI1_loop_and_bridge_target	-1.012511626
SLFN5	6.32013928	27.1841472	4_EWS-FLI1_loop_and_bridge_target	1.944940911
SLFN11	56.66485533	46.86721228	4_EWS-FLI1_loop_and_bridge_target	-0.268654528
SLFN12	11.01874271	5.014969949	4_EWS-FLI1_loop_and_bridge_target	-0.998656547
SLFN12L	2.381676514	0.552158639	4_EWS-FLI1_loop_and_bridge_target	-1.123462644
AP2B1	25.12940165	43.59010312	4_EWS-FLI1_loop_and_bridge_target	0.771049448
RARA	2.751749653	1.907118405	3_EWS-FLI1_bridge_target	-0.36797373
SMARCE1	46.21242126	23.43792182	2_EWS-FLI1_loop_target	-0.950044868
TMEM99	3.915331845	3.768358668	4_EWS-FLI1_loop_and_bridge_target	-0.043796062
KRT40	1.066023124	0.024557988	4_EWS-FLI1_loop_and_bridge_target	-1.011854761
KRTAP2-3	0.029936139	0.642398924	4_EWS-FLI1_loop_and_bridge_target	0.673249702
KRTAP4-7	0.122361574	0.228326456	4_EWS-FLI1_loop_and_bridge_target	0.130156519
KRTAP4-9	0.278242803	0.151433486	4_EWS-FLI1_loop_and_bridge_target	-0.15073083
KRTAP4-5	0.029016599	0	1_EWS-FLI1_in_promoter	-0.041266254
KRT34	0.268009705	5.681755081	4_EWS-FLI1_loop_and_bridge_target	2.397661314
EIF1	64.19676292	63.35913736	4_EWS-FLI1_loop_and_bridge_target	-0.018655346
DNAJC7	31.6621121	26.52294485	3_EWS-FLI1_bridge_target	-0.246983245
NKIRAS2	13.09909301	9.013897952	3_EWS-FLI1_bridge_target	-0.493598699
NFE2L1	64.2349448	110.693182	3_EWS-FLI1_bridge_target	0.77582423
SNX11	21.50773237	15.91564673	3_EWS-FLI1_bridge_target	-0.412062378
HOXB2	5.214157818	5.481260193	3_EWS-FLI1_bridge_target	0.060715472
HOXB-AS1	2.544158631	2.403555324	3_EWS-FLI1_bridge_target	-0.058400618
PRAC1	73.71981322	13.79338202	4_EWS-FLI1_loop_and_bridge_target	-2.336538934
PRAC2	9.006540259	2.879508223	4_EWS-FLI1_loop_and_bridge_target	-1.366997562
TTLL6	3.460198964	0.572451858	3_EWS-FLI1_bridge_target	-1.50409222
CALCOCO2	43.84462425	41.85773661	3_EWS-FLI1_bridge_target	-0.065379392
LOC105371814	0.72576883	0.806120955	4_EWS-FLI1_loop_and_bridge_target	0.065655287
ATP5G1	174.1763338	54.75817425	4_EWS-FLI1_loop_and_bridge_target	-1.651552653
UBE2Z	57.05713147	54.81764842	4_EWS-FLI1_loop_and_bridge_target	-0.056751947
SNF8	57.38757818	18.09540014	4_EWS-FLI1_loop_and_bridge_target	-1.61243632
IGF2BP1	33.82711999	10.60932397	4_EWS-FLI1_loop_and_bridge_target	-1.584927211
GNGT2	4.35095364	0.349951332	4_EWS-FLI1_loop_and_bridge_target	-1.986888633
ABI3	5.393785337	0.4344109	4_EWS-FLI1_loop_and_bridge_target	-2.156211952
PHOSPHO1	20.44672725	1.215569561	1_EWS-FLI1_in_promoter	-3.275007982
ZNF652	6.071147405	8.141455761	4_EWS-FLI1_loop_and_bridge_target	0.370479596
LOC102724596	0.775212719	0.132430213	4_EWS-FLI1_loop_and_bridge_target	-0.648569763
KAT7	40.09470401	18.39250524	3_EWS-FLI1_bridge_target	-1.08345329
COX11	26.71117804	11.49475821	2_EWS-FLI1_loop_target	-1.149145059
STXBP4	3.239876506	1.997489328	2_EWS-FLI1_loop_target	-0.500267627
CEP112	8.680108255	6.381365103	4_EWS-FLI1_loop_and_bridge_target	-0.39113553
APOH	2.059985228	0.059442191	4_EWS-FLI1_loop_and_bridge_target	-1.53021982
ABCA10	0.467327196	0.432257055	4_EWS-FLI1_loop_and_bridge_target	-0.034900165
PRO1804	0.231527684	0.099250182	4_EWS-FLI1_loop_and_bridge_target	-0.163929289
LINC01152	0.630867731	0.119715045	4_EWS-FLI1_loop_and_bridge_target	-0.54250815
LOC146795	0.214496555	0.309284205	4_EWS-FLI1_loop_and_bridge_target	0.108419898
METTL4	9.152128009	6.244452983	4_EWS-FLI1_loop_and_bridge_target	-0.486833502
NDC80	46.69566291	4.398881802	4_EWS-FLI1_loop_and_bridge_target	-3.143125547
EMILIN2	10.57378176	19.97996022	3_EWS-FLI1_bridge_target	0.858151597
LPIN2	11.16611731	14.60694277	4_EWS-FLI1_loop_and_bridge_target	0.359319135
MYL12A	158.1121651	184.2629092	4_EWS-FLI1_loop_and_bridge_target	0.21952993
LOC104968399	0.977404008	0.82506774	4_EWS-FLI1_loop_and_bridge_target	-0.115657649
MYL12B	309.9210693	240.4920217	4_EWS-FLI1_loop_and_bridge_target	-0.364572856
TGIF1	22.13756815	19.27756598	4_EWS-FLI1_loop_and_bridge_target	-0.190352751
GAPLINC	0.636367024	0.664977382	4_EWS-FLI1_loop_and_bridge_target	0.02500621

DLGAP1	19.4546681	2.562350725	1_EWS-FLI1_in_promoter	-2.521528662
DLGAP1-AS1	3.724381263	4.289635062	4_EWS-FLI1_loop_and_bridge_target	0.163042796
DLGAP1-AS2	7.143223286	24.1189643	4_EWS-FLI1_loop_and_bridge_target	1.625105114
DLGAP1-AS3	1.104971917	0.094581441	4_EWS-FLI1_loop_and_bridge_target	-0.943421685
DLGAP1-AS5	1.002005333	0.399074935	1_EWS-FLI1_in_promoter	-0.516972581
LINC00526	1.562363878	1.257252413	4_EWS-FLI1_loop_and_bridge_target	-0.182907611
LINC00667	6.913509265	8.598401907	4_EWS-FLI1_loop_and_bridge_target	0.27847662
NDUFV2	91.07964021	91.02873848	4_EWS-FLI1_loop_and_bridge_target	-0.000797744
NDUFV2-AS1	1.162089026	0.56323528	4_EWS-FLI1_loop_and_bridge_target	-0.467890996
ANKRD12	7.546565005	7.636725107	4_EWS-FLI1_loop_and_bridge_target	0.015139676
RALBP1	27.94250476	26.42011997	4_EWS-FLI1_loop_and_bridge_target	-0.077954899
PPP4R1	35.44275247	37.60794381	4_EWS-FLI1_loop_and_bridge_target	0.083265793
PPP4R1-AS1	0.195528375	0.222967288	4_EWS-FLI1_loop_and_bridge_target	0.032737443
RAB31	13.94377978	19.61707656	4_EWS-FLI1_loop_and_bridge_target	0.464294678
VAPA	33.21853332	29.22018571	4_EWS-FLI1_loop_and_bridge_target	-0.179265398
APCDD1	115.8304228	43.34861632	1_EWS-FLI1_in_promoter	-1.397455005
NAPG	28.71181121	42.19348612	4_EWS-FLI1_loop_and_bridge_target	0.539777207
GNAL	0.237423702	0.208485722	4_EWS-FLI1_loop_and_bridge_target	-0.034139145
SEH1L	33.57338826	31.60561602	4_EWS-FLI1_loop_and_bridge_target	-0.084541521
ABHD3	34.35207799	9.807581747	4_EWS-FLI1_loop_and_bridge_target	-1.709751271
MIB1	36.16157906	30.19172472	4_EWS-FLI1_loop_and_bridge_target	-0.252648478
NOL4	7.174998959	2.627776454	4_EWS-FLI1_loop_and_bridge_target	-1.172132988
DTNA	1.457258538	1.82428726	2_EWS-FLI1_loop_target	0.200837176
SETBP1	1.507507775	1.18636436	4_EWS-FLI1_loop_and_bridge_target	-0.197720327
PIAS2	5.313013861	3.196207881	4_EWS-FLI1_loop_and_bridge_target	-0.589242767
SMAD7	5.154439852	8.713015923	4_EWS-FLI1_loop_and_bridge_target	0.658291771
MEX3C	25.18846382	30.77425716	4_EWS-FLI1_loop_and_bridge_target	0.278926962
LINC01630	0.938216537	0.1845478	4_EWS-FLI1_loop_and_bridge_target	-0.710393341
DCC	21.52362057	17.96871559	4_EWS-FLI1_loop_and_bridge_target	-0.247816759
TCF4	17.63544185	15.69935813	4_EWS-FLI1_loop_and_bridge_target	-0.158256374
FECH	13.96184003	12.45418995	4_EWS-FLI1_loop_and_bridge_target	-0.153232079
NARS	176.2066837	155.5007829	4_EWS-FLI1_loop_and_bridge_target	-0.179263145
LOC100505549	6.479346477	0.786419391	4_EWS-FLI1_loop_and_bridge_target	-2.0658414
ATP8B1	39.78562772	35.21605585	4_EWS-FLI1_loop_and_bridge_target	-0.17143142
LOC101927322	0.306913742	0.167860473	3_EWS-FLI1_bridge_target	-0.162296002
MALT1	12.31164478	17.26087985	3_EWS-FLI1_bridge_target	0.456067438
ZNF532	23.26112765	19.65464533	4_EWS-FLI1_loop_and_bridge_target	-0.232180321
SEC11C	203.8587764	219.3025514	4_EWS-FLI1_loop_and_bridge_target	0.104856502
GRP	230.7191941	27.78144783	4_EWS-FLI1_loop_and_bridge_target	-3.009166479
RAX	3.223102434	0.573145191	4_EWS-FLI1_loop_and_bridge_target	-1.424651413
LMAN1	100.078048	196.3448499	4_EWS-FLI1_loop_and_bridge_target	0.965249161
BCL2	8.40297469	4.265071509	4_EWS-FLI1_loop_and_bridge_target	-0.836664109
KDSR	63.94062086	70.4653062	4_EWS-FLI1_loop_and_bridge_target	0.138121859
VPS4B	50.61100408	43.37366015	4_EWS-FLI1_loop_and_bridge_target	-0.21797514
SERPINB13	0.759521008	0.029680936	1_EWS-FLI1_in_promoter	-0.772985376
SERPINB4	0.482732924	1.694001703	4_EWS-FLI1_loop_and_bridge_target	0.861492006
SERPINB3	0.107648805	0.448615158	4_EWS-FLI1_loop_and_bridge_target	0.387173848
SERPINB8	11.48290509	15.8361759	4_EWS-FLI1_loop_and_bridge_target	0.431610762
DSEL	16.32539157	12.96497856	4_EWS-FLI1_loop_and_bridge_target	-0.311074601
LOC643542	0.144966578	0.095473182	4_EWS-FLI1_loop_and_bridge_target	-0.06375132
TMX3	45.95575222	43.23023665	4_EWS-FLI1_loop_and_bridge_target	-0.086268942
CCDC102B	3.324726656	7.952611057	4_EWS-FLI1_loop_and_bridge_target	1.04969956
RTTN	6.32875214	1.846902322	2_EWS-FLI1_loop_target	-1.364174578
CBARP	0.646592578	0.603270237	3_EWS-FLI1_bridge_target	-0.03846601
ATP5D	11.49800205	10.77540909	3_EWS-FLI1_bridge_target	-0.0859203
MIDN	6.475582247	7.989740435	3_EWS-FLI1_bridge_target	0.266093511

CIRBP-AS1	3.498554422	2.957037142	3_EWS-FLI1_bridge_target	-0.185040869
CIRBP	57.3618669	42.28847378	3_EWS-FLI1_bridge_target	-0.431043097
C19orf24	15.26060738	14.38436828	3_EWS-FLI1_bridge_target	-0.079915943
TIMM44	41.93756685	43.68477476	3_EWS-FLI1_bridge_target	0.057542912
NDUFA7	43.47265781	26.63574421	4_EWS-FLI1_loop_and_bridge_target	-0.686383163
RPS28	99.48515589	114.4359179	4_EWS-FLI1_loop_and_bridge_target	0.200109792
ANGPTL4	3.119581386	11.29874364	4_EWS-FLI1_loop_and_bridge_target	1.577941298
RAB11B-AS1	2.595480814	0.640321909	4_EWS-FLI1_loop_and_bridge_target	-1.132205743
RAB11B	3.407534331	3.610431558	4_EWS-FLI1_loop_and_bridge_target	0.064929993
HNRNPM	85.32181157	47.61595013	4_EWS-FLI1_loop_and_bridge_target	-0.828295425
ADAMTS10	2.507309404	2.429290939	4_EWS-FLI1_loop_and_bridge_target	-0.032454399
ZNF558	12.60269019	15.93400793	4_EWS-FLI1_loop_and_bridge_target	0.31603147
FBXL12	8.687139106	13.28512599	4_EWS-FLI1_loop_and_bridge_target	0.560371197
OLFM2	16.34055213	8.206464604	4_EWS-FLI1_loop_and_bridge_target	-0.91343068
COL5A3	11.4700796	56.65107845	4_EWS-FLI1_loop_and_bridge_target	2.208876921
RDH8	0.119045929	0.249906902	4_EWS-FLI1_loop_and_bridge_target	0.159551392
PPAN	2.649452233	2.134541112	4_EWS-FLI1_loop_and_bridge_target	-0.219425686
SNORD105	3.750822103	2.939591191	4_EWS-FLI1_loop_and_bridge_target	-0.270131256
SNORD105B	3.188198788	2.715926644	4_EWS-FLI1_loop_and_bridge_target	-0.172607898
P2RY11	0.864815056	1.348800277	4_EWS-FLI1_loop_and_bridge_target	0.332891486
EIF3G	89.56377429	79.78331447	4_EWS-FLI1_loop_and_bridge_target	-0.164876745
S1PR2	4.604385303	7.403267303	4_EWS-FLI1_loop_and_bridge_target	0.584394231
CDC37	137.2203811	167.5523418	4_EWS-FLI1_loop_and_bridge_target	0.28622631
PDE4A	3.509245897	0.784573118	4_EWS-FLI1_loop_and_bridge_target	-1.337307172
KRI1	22.28450161	14.43243548	3_EWS-FLI1_bridge_target	-0.593404243
CDKN2D	10.11799419	4.924894856	3_EWS-FLI1_bridge_target	-0.908035076
SMARCA4	36.70200177	25.07462351	3_EWS-FLI1_bridge_target	-0.531994703
LDLR	60.28308789	175.0428345	3_EWS-FLI1_bridge_target	1.522365609
SWSAP1	2.225216964	1.632263976	4_EWS-FLI1_loop_and_bridge_target	-0.293092039
EPOR	5.250872177	1.476792021	4_EWS-FLI1_loop_and_bridge_target	-1.335584772
ZNF653	1.935692121	2.353110253	3_EWS-FLI1_bridge_target	0.191799246
ZNF627	15.98429177	13.57727709	3_EWS-FLI1_bridge_target	-0.220479798
SYCE2	2.309991833	0.790672463	3_EWS-FLI1_bridge_target	-0.886326183
STX10	25.74361807	16.93784546	4_EWS-FLI1_loop_and_bridge_target	-0.57618804
IER2	33.3405187	48.54129433	4_EWS-FLI1_loop_and_bridge_target	0.52871973
LOC284454	5.326843972	10.7111936	4_EWS-FLI1_loop_and_bridge_target	0.888330199
BRD4	7.35942197	4.059773526	4_EWS-FLI1_loop_and_bridge_target	-0.724330376
AKAP8	11.83853888	13.34575902	4_EWS-FLI1_loop_and_bridge_target	0.160143279
AKAP8L	42.71372367	32.83436989	4_EWS-FLI1_loop_and_bridge_target	-0.369596756
RASAL3	0.887656562	0.104879031	1_EWS-FLI1_in_promoter	-0.772707883
CYP4F22	49.18795615	5.413313863	1_EWS-FLI1_in_promoter	-2.968199277
TPM4	177.1801929	190.7301185	4_EWS-FLI1_loop_and_bridge_target	0.105740014
CHERP	4.132590761	2.55109821	4_EWS-FLI1_loop_and_bridge_target	-0.531421974
SLC35E1	16.43866094	44.87636262	4_EWS-FLI1_loop_and_bridge_target	1.395461745
MED26	5.363063036	3.863972688	4_EWS-FLI1_loop_and_bridge_target	-0.387586286
SMIM7	46.47456222	22.90085762	4_EWS-FLI1_loop_and_bridge_target	-0.99009231
TMEM38A	9.911864785	5.872291033	4_EWS-FLI1_loop_and_bridge_target	-0.667034633
NWD1	3.390425368	0.420541301	4_EWS-FLI1_loop_and_bridge_target	-1.627919945
ANO8	2.309754806	2.676554554	4_EWS-FLI1_loop_and_bridge_target	0.151630049
GTPBP3	5.493719081	3.780312595	4_EWS-FLI1_loop_and_bridge_target	-0.441940012
RPL18A	151.5511007	202.6442475	4_EWS-FLI1_loop_and_bridge_target	0.416758473
SNORA68	29.05336038	31.13251251	4_EWS-FLI1_loop_and_bridge_target	0.096507479
CCDC124	15.9792641	15.64473103	3_EWS-FLI1_bridge_target	-0.028708375
KCNN1	22.57297538	2.892860769	4_EWS-FLI1_loop_and_bridge_target	-2.598231216
ARRDC2	9.050370753	8.767201798	4_EWS-FLI1_loop_and_bridge_target	-0.041231513
PIK3R2	8.045536767	9.936525973	4_EWS-FLI1_loop_and_bridge_target	0.273876513

MPV17L2	10.7619317	31.63838356	4_EWS-FLI1_loop_and_bridge_target	1.472444591
JUND	17.38485982	15.41390326	3_EWS-FLI1_bridge_target	-0.163599821
LSM4	58.59297937	26.05248647	3_EWS-FLI1_bridge_target	-1.139381175
SSBP4	19.55381014	15.84670498	4_EWS-FLI1_loop_and_bridge_target	-0.286939412
ISYNA1	17.27330575	7.929322489	4_EWS-FLI1_loop_and_bridge_target	-1.033115029
COPE	67.44136907	145.8124379	3_EWS-FLI1_bridge_target	1.101033674
DDX49	27.9366787	37.18556934	3_EWS-FLI1_bridge_target	0.400128193
TSHZ3	2.59053319	4.012936731	4_EWS-FLI1_loop_and_bridge_target	0.481457927
ZFP36	1.601378387	6.030668164	3_EWS-FLI1_bridge_target	1.43438554
PLEKHG2	5.212099399	2.307093376	3_EWS-FLI1_bridge_target	-0.909517133
DYRK1B	3.175793345	5.069528444	3_EWS-FLI1_bridge_target	0.539534118
FBL	116.0121228	88.86480895	3_EWS-FLI1_bridge_target	-0.380829832
ZNF546	1.425097452	0.89382113	3_EWS-FLI1_bridge_target	-0.356742647
ZNF780B	4.36427027	2.164334278	3_EWS-FLI1_bridge_target	-0.761479914
ZNF780A	6.535234174	4.098723983	3_EWS-FLI1_bridge_target	-0.563516107
MAP3K10	1.759262195	2.91571791	3_EWS-FLI1_bridge_target	0.504994283
AKT2	19.37221769	12.20366133	3_EWS-FLI1_bridge_target	-0.625664999
SERTAD1	14.85001562	15.3769313	3_EWS-FLI1_bridge_target	0.047180789
LTBP4	3.444612866	5.040301616	3_EWS-FLI1_bridge_target	0.442562828
C19orf54	1.85756297	1.421923575	3_EWS-FLI1_bridge_target	-0.238631949
SNRPA	56.20937335	23.76747428	3_EWS-FLI1_bridge_target	-1.207804788
MIA	0.07613609	0.53276173	3_EWS-FLI1_bridge_target	0.510272909
HNRNPUL1	52.76045782	30.80934366	4_EWS-FLI1_loop_and_bridge_target	-0.75709482
CCDC97	5.40515821	2.163335509	4_EWS-FLI1_loop_and_bridge_target	-1.017787628
TGFB1	12.97003208	10.22161371	4_EWS-FLI1_loop_and_bridge_target	-0.316055178
EXOSC5	17.08078303	10.78399876	4_EWS-FLI1_loop_and_bridge_target	-0.617627975
BCKDHA	19.84806506	17.11644712	4_EWS-FLI1_loop_and_bridge_target	-0.20261344
B3GNT8	0.102076802	0.139681748	4_EWS-FLI1_loop_and_bridge_target	0.048406247
ATP5SL	37.0700426	20.05621029	4_EWS-FLI1_loop_and_bridge_target	-0.854410382
LINC01480	0.987141047	1.418840339	4_EWS-FLI1_loop_and_bridge_target	0.283621265
CEACAM21	1.535568747	0.263408292	4_EWS-FLI1_loop_and_bridge_target	-1.004988444
CEACAM6	6.432782483	0.182981089	1_EWS-FLI1_in_promoter	-2.651475378
RPS19	326.5647816	323.4632634	4_EWS-FLI1_loop_and_bridge_target	-0.013725111
CD79A	25.7894363	0.662070088	4_EWS-FLI1_loop_and_bridge_target	-4.010611099
ARHGEF1	20.7003003	16.42457881	4_EWS-FLI1_loop_and_bridge_target	-0.316591224
RABAC1	33.77041293	62.6764137	3_EWS-FLI1_bridge_target	0.872898884
ZNF574	8.476067298	9.052014851	3_EWS-FLI1_bridge_target	0.085124358
DEDD2	13.51707028	16.9887135	3_EWS-FLI1_bridge_target	0.309341684
ZNF526	7.660660808	6.751158459	3_EWS-FLI1_bridge_target	-0.160065161
CIC	3.99232572	5.740730314	3_EWS-FLI1_bridge_target	0.433192842
BCL3	1.067294015	6.351316293	3_EWS-FLI1_bridge_target	1.830259009
LIG1	25.57402127	5.092358987	3_EWS-FLI1_bridge_target	-2.124943699
C19orf68	0.97032137	0.550480858	3_EWS-FLI1_bridge_target	-0.345715245
CARD8	23.43099407	19.98740419	4_EWS-FLI1_loop_and_bridge_target	-0.219188828
CARD8-AS1	6.873386281	6.297697791	4_EWS-FLI1_loop_and_bridge_target	-0.109542853
CCDC114	0.241590219	0.077597904	3_EWS-FLI1_bridge_target	-0.204370147
EMP3	116.9823709	165.9711492	3_EWS-FLI1_bridge_target	0.501027536
GRIN2D	0.27043037	0.35977718	3_EWS-FLI1_bridge_target	0.098052957
GRWD1	23.88552838	22.17374854	3_EWS-FLI1_bridge_target	-0.102815588
CYTH2	21.69110111	14.56107462	3_EWS-FLI1_bridge_target	-0.544184925
SULT2B1	0.363068901	0.067748585	3_EWS-FLI1_bridge_target	-0.352286504
RPL18	228.8170629	292.2002882	4_EWS-FLI1_loop_and_bridge_target	0.351400605
SPHK2	2.806797573	0.849619092	4_EWS-FLI1_loop_and_bridge_target	-1.041349661
DBP	3.668805231	0.591303742	3_EWS-FLI1_bridge_target	-1.552844167
CA11	12.01075852	4.292380587	4_EWS-FLI1_loop_and_bridge_target	-1.297716354
SEC1P	0.033699345	0.071866185	3_EWS-FLI1_bridge_target	0.052308173

MAMSTR	0.720591579	0.203256884	4_EWS-FLI1_loop_and_bridge_target	-0.515960004
IZUMO1	0.423078512	0.211463483	3_EWS-FLI1_bridge_target	-0.23226434
HSD17B14	0.484544359	0.836827842	3_EWS-FLI1_bridge_target	0.307196213
PLEKHA4	6.192323821	8.498266735	4_EWS-FLI1_loop_and_bridge_target	0.401206294
PPP1R15A	41.36229699	52.29679758	4_EWS-FLI1_loop_and_bridge_target	0.331268031
TULP2	0.35504941	0.385344137	3_EWS-FLI1_bridge_target	0.031898946
NUCB1	30.36638814	58.91041919	3_EWS-FLI1_bridge_target	0.933587516
FTL	486.6706751	561.5172026	4_EWS-FLI1_loop_and_bridge_target	0.205989995
GYS1	19.16444092	7.889477714	4_EWS-FLI1_loop_and_bridge_target	-1.181642842
RUVBL2	63.00948138	48.90397334	4_EWS-FLI1_loop_and_bridge_target	-0.359130933
SNRNP70	58.67182501	38.61733165	4_EWS-FLI1_loop_and_bridge_target	-0.590918188
LIN7B	6.367952016	4.948450892	4_EWS-FLI1_loop_and_bridge_target	-0.308749658
C19orf73	1.079873783	0.495764387	4_EWS-FLI1_loop_and_bridge_target	-0.475613041
PPFIA3	6.685670339	5.477454193	4_EWS-FLI1_loop_and_bridge_target	-0.246744188
TRPM4	15.95494328	6.461084928	4_EWS-FLI1_loop_and_bridge_target	-1.184248622
TEAD2	43.23789181	13.93251258	4_EWS-FLI1_loop_and_bridge_target	-1.566825697
DKKL1	0.692831412	0.175297905	4_EWS-FLI1_loop_and_bridge_target	-0.526411818
SLC17A7	0.364243141	0.0159924	4_EWS-FLI1_loop_and_bridge_target	-0.425211181
PIH1D1	40.04950837	26.69965767	4_EWS-FLI1_loop_and_bridge_target	-0.567496797
ALDH16A1	4.643725472	3.669869541	4_EWS-FLI1_loop_and_bridge_target	-0.273265566
RPL13A	104.7134171	128.0987955	1_EWS-FLI1_in_promoter	0.288317045
SNORD32A	73.56185496	78.35495683	1_EWS-FLI1_in_promoter	0.089882591
SNORD33	15.97940597	18.06254828	1_EWS-FLI1_in_promoter	0.166955005
SNORD34	34.20067791	24.69808039	1_EWS-FLI1_in_promoter	-0.453942617
SNORD35A	61.68793934	35.55668304	1_EWS-FLI1_in_promoter	-0.778052733
RPS11	657.1045502	695.7032485	1_EWS-FLI1_in_promoter	0.082227493
SNORD35B	29.75652202	31.18187043	1_EWS-FLI1_in_promoter	0.065355808
FCGRT	169.403979	31.51560761	1_EWS-FLI1_in_promoter	-2.389754735
RCN3	27.78926236	60.88435913	4_EWS-FLI1_loop_and_bridge_target	1.104043999
NOSIP	43.26807894	48.94249861	4_EWS-FLI1_loop_and_bridge_target	0.174001233
PRRG2	0.384218828	0.160170033	4_EWS-FLI1_loop_and_bridge_target	-0.254735774
PRR12	0.701651699	0.613511107	4_EWS-FLI1_loop_and_bridge_target	-0.076732262
RRAS	24.22513724	24.43625682	4_EWS-FLI1_loop_and_bridge_target	0.012024262
SCAF1	5.960963372	6.20987138	4_EWS-FLI1_loop_and_bridge_target	0.050686539
IRF3	16.17257455	12.11736956	4_EWS-FLI1_loop_and_bridge_target	-0.388627903
BCL2L12	14.14595765	4.511872534	4_EWS-FLI1_loop_and_bridge_target	-1.458318369
PRMT1	166.3637913	163.1697187	4_EWS-FLI1_loop_and_bridge_target	-0.027799394
ADM5	1.280110119	3.075992676	4_EWS-FLI1_loop_and_bridge_target	0.838047957
CPT1C	4.300879152	1.193990827	4_EWS-FLI1_loop_and_bridge_target	-1.272674157
AP2A1	17.65855745	28.14592856	4_EWS-FLI1_loop_and_bridge_target	0.643456914
FUZ	11.48847792	2.509221243	4_EWS-FLI1_loop_and_bridge_target	-1.831374842
MED25	7.059485835	4.555192374	4_EWS-FLI1_loop_and_bridge_target	-0.536850931
PTOV1-AS1	6.80386557	4.243078444	4_EWS-FLI1_loop_and_bridge_target	-0.573774795
PTOV1	30.02262103	21.31398944	4_EWS-FLI1_loop_and_bridge_target	-0.475372111
PNKP	11.08552094	4.155087463	4_EWS-FLI1_loop_and_bridge_target	-1.229210852
AKT1S1	8.232119927	13.21072092	4_EWS-FLI1_loop_and_bridge_target	0.622245876
TBC1D17	5.623703089	11.44187421	4_EWS-FLI1_loop_and_bridge_target	0.909493915
IL4I1	0.270792525	5.720358316	4_EWS-FLI1_loop_and_bridge_target	2.402809647
NUP62	27.78551803	15.06444271	4_EWS-FLI1_loop_and_bridge_target	-0.841472243
ATF5	20.15274656	14.6947306	4_EWS-FLI1_loop_and_bridge_target	-0.430564736
VRK3	9.628755163	14.22828443	4_EWS-FLI1_loop_and_bridge_target	0.518780783
ZNF473	12.20199247	13.50888801	4_EWS-FLI1_loop_and_bridge_target	0.136181272
NR1H2	16.39557922	24.29198057	3_EWS-FLI1_bridge_target	0.539959299
CLEC11A	28.32443841	15.57693819	4_EWS-FLI1_loop_and_bridge_target	-0.822925918
C19orf48	43.24935025	14.3405257	3_EWS-FLI1_bridge_target	-1.528308348
SNORD88C	23.79645281	9.93573164	3_EWS-FLI1_bridge_target	-1.181084009

MYADM	4.406119632	7.338874396	4_EWS-FLI1_loop_and_bridge_target	0.62525922
MBOAT7	12.54224398	12.51078845	4_EWS-FLI1_loop_and_bridge_target	-0.003354948
TSEN34	18.63085558	11.57828803	4_EWS-FLI1_loop_and_bridge_target	-0.642187475
CDC42EP5	11.85108236	16.23837024	2_EWS-FLI1_loop_target	0.423733511
U2AF2	63.58068742	56.70239865	3_EWS-FLI1_bridge_target	-0.162471506
ZIM2	0.378069591	0.184266409	4_EWS-FLI1_loop_and_bridge_target	-0.218655082
PEG3	137.1271485	30.83819195	4_EWS-FLI1_loop_and_bridge_target	-2.11716659
MIMT1	3.247606219	0.526557206	4_EWS-FLI1_loop_and_bridge_target	-1.476368371
MZF1	1.392722237	1.824874595	4_EWS-FLI1_loop_and_bridge_target	0.239533895
CENPBD1P1	15.84461319	20.46686334	4_EWS-FLI1_loop_and_bridge_target	0.349824106
SDCBP2	0.177714537	0.053058396	4_EWS-FLI1_loop_and_bridge_target	-0.161404449
SDCBP2-AS1	2.447362692	2.044890079	4_EWS-FLI1_loop_and_bridge_target	-0.179102943
FKBP1A	68.24054531	84.98647838	3_EWS-FLI1_bridge_target	0.312492723
NSFL1C	26.56178151	21.53620474	3_EWS-FLI1_bridge_target	-0.290424567
SIRPB2	0.252084902	0.00839982	4_EWS-FLI1_loop_and_bridge_target	-0.312264627
SIRPA	25.53976483	9.530898745	4_EWS-FLI1_loop_and_bridge_target	-1.333527021
LOC727993	0.1595834	0	1_EWS-FLI1_in_promoter	-0.213606585
STK35	15.03839204	7.870984621	4_EWS-FLI1_loop_and_bridge_target	-0.85436336
SNORD119	7.571972121	3.470350712	4_EWS-FLI1_loop_and_bridge_target	-0.939239139
LOC101929125	1.354758692	1.044897179	4_EWS-FLI1_loop_and_bridge_target	-0.203550921
PANK2	8.196637459	5.218003903	3_EWS-FLI1_bridge_target	-0.564654947
ADRA1D	23.48695581	10.59515069	4_EWS-FLI1_loop_and_bridge_target	-1.078491862
PRNP	67.86431779	326.004224	4_EWS-FLI1_loop_and_bridge_target	2.247480726
PRND	0.10106227	0.05598528	3_EWS-FLI1_bridge_target	-0.060306337
SLC23A2	10.93638938	6.736662339	4_EWS-FLI1_loop_and_bridge_target	-0.625583289
TMEM230	60.22587437	41.45529331	4_EWS-FLI1_loop_and_bridge_target	-0.528197033
PCNA	80.69989997	32.66681625	4_EWS-FLI1_loop_and_bridge_target	-1.279007015
CDS2	25.15541165	26.44232717	4_EWS-FLI1_loop_and_bridge_target	0.069293354
MKKS	28.95465982	22.89744026	4_EWS-FLI1_loop_and_bridge_target	-0.325924355
SLX4IP	4.234803141	1.786125737	4_EWS-FLI1_loop_and_bridge_target	-0.909874916
JAG1	30.99267905	30.94722448	4_EWS-FLI1_loop_and_bridge_target	-0.00205121
BTBD3	15.38342759	10.12230184	3_EWS-FLI1_bridge_target	-0.55878182
TASP1	10.0630114	12.79083165	4_EWS-FLI1_loop_and_bridge_target	0.317965314
ESF1	37.94181285	17.58679723	4_EWS-FLI1_loop_and_bridge_target	-1.067041852
NDUFAF5	14.34557164	8.309093417	4_EWS-FLI1_loop_and_bridge_target	-0.721109809
SNRPB2	61.35148982	58.64942839	2_EWS-FLI1_loop_target	-0.063915719
PCSK2	58.1942537	12.83589062	4_EWS-FLI1_loop_and_bridge_target	-2.097041619
BFSP1	4.950237533	2.720525611	4_EWS-FLI1_loop_and_bridge_target	-0.677440812
DSTN	122.4370591	199.7504567	4_EWS-FLI1_loop_and_bridge_target	0.701627675
RRBP1	37.7404713	104.3678168	3_EWS-FLI1_bridge_target	1.443520872
SNX5	53.24367228	40.07617326	4_EWS-FLI1_loop_and_bridge_target	-0.401153068
SNORD17	167.8848982	137.1572968	4_EWS-FLI1_loop_and_bridge_target	-0.289728566
MGME1	21.56777341	11.81912202	4_EWS-FLI1_loop_and_bridge_target	-0.81596663
DZANK1	0.688650938	1.111645403	4_EWS-FLI1_loop_and_bridge_target	0.322496453
POLR3F	14.82704497	12.23625011	4_EWS-FLI1_loop_and_bridge_target	-0.25789746
SEC23B	44.43227667	127.0554022	4_EWS-FLI1_loop_and_bridge_target	1.494978609
SLC24A3	45.56771443	7.804751569	1_EWS-FLI1_in_promoter	-2.402975872
RALGAPA2	10.96826971	5.87722212	3_EWS-FLI1_bridge_target	-0.799316745
NKX2-2	8.497776	0.926698047	4_EWS-FLI1_loop_and_bridge_target	-2.301459242
LOC101929625	0.494817449	0.062954208	1_EWS-FLI1_in_promoter	-0.491889862
APMAP	77.48141277	121.0245469	2_EWS-FLI1_loop_target	0.636748477
MAFB	11.98980741	4.551739521	4_EWS-FLI1_loop_and_bridge_target	-1.226368255
SRSF6	69.55261371	53.06827998	3_EWS-FLI1_bridge_target	-0.383917068
TOX2	8.200805947	2.152993784	3_EWS-FLI1_bridge_target	-1.545037914
OSER1	28.45773155	19.02567703	3_EWS-FLI1_bridge_target	-0.556795319
OSER1-AS1	1.384825405	1.16347947	3_EWS-FLI1_bridge_target	-0.140530219

PKIG	57.04663315	67.92479716	3_EWS-FLI1_bridge_target	0.247810727
SDC4	30.00715122	34.20090984	3_EWS-FLI1_bridge_target	0.183011734
WFDC3	0.178539132	0.904274243	3_EWS-FLI1_bridge_target	0.6922416
DNTTIP1	25.53524797	47.29822844	3_EWS-FLI1_bridge_target	0.864060242
UBE2C	84.85318902	15.82035745	3_EWS-FLI1_bridge_target	-2.35166336
NEURL2	0.412229279	0.392792692	3_EWS-FLI1_bridge_target	-0.019993795
CTSA	22.70088011	27.46864043	3_EWS-FLI1_bridge_target	0.264432963
PLTP	38.15517039	36.99507991	3_EWS-FLI1_bridge_target	-0.043390214
PCIF1	7.376477729	4.116112778	3_EWS-FLI1_bridge_target	-0.711295658
ZNF335	12.91034316	11.17140237	3_EWS-FLI1_bridge_target	-0.192662608
MMP9	6.938699759	12.03077551	3_EWS-FLI1_bridge_target	0.714948307
SLC12A5	0.464775782	0.564867096	3_EWS-FLI1_bridge_target	0.095360291
NCOA5	20.61841062	12.75909245	3_EWS-FLI1_bridge_target	-0.651875148
PREX1	73.44056523	15.252771	4_EWS-FLI1_loop_and_bridge_target	-2.1954033
ARFGEF2	16.14778071	29.72736293	4_EWS-FLI1_loop_and_bridge_target	0.841502085
CSE1L-AS1	0.291877354	0.167582328	4_EWS-FLI1_loop_and_bridge_target	-0.145944832
CSE1L	123.2313109	54.19045105	4_EWS-FLI1_loop_and_bridge_target	-1.17053825
STAU1	34.22284225	33.20260747	3_EWS-FLI1_bridge_target	-0.042405015
DDX27	40.07548131	34.96656577	3_EWS-FLI1_bridge_target	-0.191621069
ZNFX1	15.26562709	30.08413212	3_EWS-FLI1_bridge_target	0.934351855
ZFAS1	111.3512542	119.0413243	3_EWS-FLI1_bridge_target	0.095514911
SNORD12C	25.66701809	9.940389742	3_EWS-FLI1_bridge_target	-1.285392378
SNORD12B	41.47753763	26.45360265	3_EWS-FLI1_bridge_target	-0.629704655
SNORD12	40.38385131	23.53228292	3_EWS-FLI1_bridge_target	-0.754386419
UBE2V1	1.330391502	0.660712018	4_EWS-FLI1_loop_and_bridge_target	-0.488770426
CEBPB-AS1	0.304557166	0.196104581	4_EWS-FLI1_loop_and_bridge_target	-0.125216629
CEBPB	21.62772422	8.968913983	4_EWS-FLI1_loop_and_bridge_target	-1.182583243
LINC01270	0.005598242	0.031338925	4_EWS-FLI1_loop_and_bridge_target	0.036464486
PTPN1	31.5222753	22.93178141	4_EWS-FLI1_loop_and_bridge_target	-0.442500403
FAM65C	9.362976285	5.308380491	4_EWS-FLI1_loop_and_bridge_target	-0.716096826
LOC100506175	0.339808021	0.074597776	4_EWS-FLI1_loop_and_bridge_target	-0.318229537
PARD6B	11.2246704	8.685920976	4_EWS-FLI1_loop_and_bridge_target	-0.33583443
BCAS4	19.3698115	8.565427093	4_EWS-FLI1_loop_and_bridge_target	-1.090531339
ADNP	21.22791496	12.98466039	4_EWS-FLI1_loop_and_bridge_target	-0.668527407
ADNP-AS1	2.449130398	1.731088065	4_EWS-FLI1_loop_and_bridge_target	-0.336756837
DPM1	65.09325734	65.90598104	4_EWS-FLI1_loop_and_bridge_target	0.017632087
MOCS3	7.106264009	5.951123837	4_EWS-FLI1_loop_and_bridge_target	-0.221790915
NFATC2	0.94701403	1.068211069	3_EWS-FLI1_bridge_target	0.087120146
SALL4	7.982060385	9.153711449	3_EWS-FLI1_bridge_target	0.176888841
ZFP64	7.445701647	7.374878442	3_EWS-FLI1_bridge_target	-0.012149033
ZNF217	21.39572766	29.41118929	4_EWS-FLI1_loop_and_bridge_target	0.441378695
LOC105372672	0.368190936	0.312292527	4_EWS-FLI1_loop_and_bridge_target	-0.060180226
PFDN4	37.71833395	31.9116096	4_EWS-FLI1_loop_and_bridge_target	-0.234420289
LINC00659	23.37410329	3.30322334	3_EWS-FLI1_bridge_target	-2.50185952
LIPI	94.49360263	11.49559006	1_EWS-FLI1_in_promoter	-2.933985057
RBM11	103.0993484	21.33781745	1_EWS-FLI1_in_promoter	-2.220400901
HSPA13	20.70969468	118.4162965	4_EWS-FLI1_loop_and_bridge_target	2.45958839
NRIP1	10.44898819	16.98458872	4_EWS-FLI1_loop_and_bridge_target	0.651541062
MRPL39	53.29818913	32.99225207	4_EWS-FLI1_loop_and_bridge_target	-0.675698137
ATP5J	32.2120437	27.90476466	4_EWS-FLI1_loop_and_bridge_target	-0.200399176
GABPA	12.2136368	7.044744911	4_EWS-FLI1_loop_and_bridge_target	-0.715909014
APP	194.1537625	253.0865467	4_EWS-FLI1_loop_and_bridge_target	0.380708712
CYYR1-AS1	1.082201097	0.455162994	4_EWS-FLI1_loop_and_bridge_target	-0.51692865
CYYR1	34.4073497	15.30188258	4_EWS-FLI1_loop_and_bridge_target	-1.119010279
ADAMTS1	4.389857028	8.58799079	4_EWS-FLI1_loop_and_bridge_target	0.830981519
CBR3	17.79886518	8.741859809	4_EWS-FLI1_loop_and_bridge_target	-0.948376447

MORC3	22.6735984	28.70489536	4_EWS-FLI1_loop_and_bridge_target	0.327421693
CHAF1B	39.99307896	5.309617011	3_EWS-FLI1_bridge_target	-2.699756011
CLDN14	0.168845419	0.268732098	4_EWS-FLI1_loop_and_bridge_target	0.118303321
SIM2	14.67120606	6.77693313	4_EWS-FLI1_loop_and_bridge_target	-1.010842975
HLCS	9.973073515	9.711814416	4_EWS-FLI1_loop_and_bridge_target	-0.034764805
PIGP	22.47358569	11.70118251	4_EWS-FLI1_loop_and_bridge_target	-0.886075414
TTC3	50.68324381	57.40456476	4_EWS-FLI1_loop_and_bridge_target	0.17638451
DYRK1A	29.79219761	22.06126416	4_EWS-FLI1_loop_and_bridge_target	-0.417093235
ETS2	26.63534712	23.30323775	4_EWS-FLI1_loop_and_bridge_target	-0.185366208
RIPK4	8.454655257	7.030840403	3_EWS-FLI1_bridge_target	-0.235473885
PRDM15	5.100790141	2.932518377	3_EWS-FLI1_bridge_target	-0.633542597
CSTB	50.48207527	67.76993694	2_EWS-FLI1_loop_target	0.417707812
RRP1	43.09273226	24.46831828	3_EWS-FLI1_bridge_target	-0.791837179
C21orf33	30.8554563	27.81092468	4_EWS-FLI1_loop_and_bridge_target	-0.144924539
C21orf2	3.248654793	5.495800994	4_EWS-FLI1_loop_and_bridge_target	0.612501306
UBE2G2	69.21800136	45.9538722	4_EWS-FLI1_loop_and_bridge_target	-0.580596793
LINC01424	1.204326921	0.322283145	4_EWS-FLI1_loop_and_bridge_target	-0.737307065
SUMO3	81.31649548	50.16382018	4_EWS-FLI1_loop_and_bridge_target	-0.686057575
PTTG1IP	147.9206908	179.195143	4_EWS-FLI1_loop_and_bridge_target	0.275015912
ITGB2	4.499122761	8.71313609	1_EWS-FLI1_in_promoter	0.820735682
ITGB2-AS1	17.3374117	3.780643328	1_EWS-FLI1_in_promoter	-1.93951334
LINC01547	93.69364786	6.409314606	1_EWS-FLI1_in_promoter	-3.675855653
FAM207A	16.10999998	11.6417772	1_EWS-FLI1_in_promoter	-0.436640464
SSR4P1	1.915461921	0.722230087	3_EWS-FLI1_bridge_target	-0.759446583
ADARB1	8.235324958	6.461242793	3_EWS-FLI1_bridge_target	-0.30774677
APOL6	10.81578908	23.33784327	4_EWS-FLI1_loop_and_bridge_target	1.042485349
RBFOX2	53.49074577	32.30200251	4_EWS-FLI1_loop_and_bridge_target	-0.710402304
TOMM22	102.1055919	37.93837873	3_EWS-FLI1_bridge_target	-1.404857858
JOSD1	43.7084883	54.74970765	3_EWS-FLI1_bridge_target	0.318415474
GTPBP1	21.87204177	19.03452347	3_EWS-FLI1_bridge_target	-0.191096965
SUN2	36.92111318	9.978635419	3_EWS-FLI1_bridge_target	-1.788302569
DNAL4	65.41918051	13.8987396	2_EWS-FLI1_loop_target	-2.156409636
RPL3	276.3772803	363.0862769	3_EWS-FLI1_bridge_target	0.392430741
SNORD83B	9.87313173	14.20125084	3_EWS-FLI1_bridge_target	0.483422509
SNORD83A	12.08159541	11.02162512	3_EWS-FLI1_bridge_target	-0.121906563
SNORD139	15.53522318	22.71502284	3_EWS-FLI1_bridge_target	0.520258739
SNORD43	23.24299761	11.7064365	3_EWS-FLI1_bridge_target	-0.932008611
MIEF1	15.60522787	17.23079949	3_EWS-FLI1_bridge_target	0.134740309
CACNA1I	1.167922451	0.046394268	3_EWS-FLI1_bridge_target	-1.050886608
RRP7BP	5.95791207	1.957370276	3_EWS-FLI1_bridge_target	-1.234339561
POLDIP3	57.96824669	42.52670322	4_EWS-FLI1_loop_and_bridge_target	-0.438037547
RNU12	38.85751006	23.39741726	4_EWS-FLI1_loop_and_bridge_target	-0.708123156
CYB5R3	22.63738986	23.84405392	4_EWS-FLI1_loop_and_bridge_target	0.071829869
KIAA1644	4.674700611	3.080049331	4_EWS-FLI1_loop_and_bridge_target	-0.475957684
FAM19A5	19.0894231	8.58775234	4_EWS-FLI1_loop_and_bridge_target	-1.067171586

SKNMC_EWS-FLI1_target genes				
**Target Name**	**shGFP_RPKM**	**shFLI1_RPKM**	**Loop_Target_Catagory**	**lof2 Fold Change**
PARK7	206.7419089	176.0917304	4_EWS-FLI1_loop_and_bridge_target	-0.230295444
RERE	2.396852026	2.649694548	4_EWS-FLI1_loop_and_bridge_target	0.103577353
LOC102724552	0.19991686	0.301385795	4_EWS-FLI1_loop_and_bridge_target	0.117114265
ENO1	524.8543465	499.6265491	4_EWS-FLI1_loop_and_bridge_target	-0.070928449
ENO1-AS1	0.985091073	0.551732371	4_EWS-FLI1_loop_and_bridge_target	-0.355325441

SLC2A5	0.563993092	1.155746568	3_EWS-FLI1_bridge_target	0.462953443
H6PD	5.507128454	7.217954118	3_EWS-FLI1_bridge_target	0.336758242
PGD	89.90991719	46.48790734	3_EWS-FLI1_bridge_target	-0.936877502
APITD1	3.545311685	1.34834322	2_EWS-FLI1_loop_target	-0.952735948
CORT	0.33812096	0.509736171	2_EWS-FLI1_loop_target	0.174087923
DFFA	26.1632622	19.6393959	3_EWS-FLI1_bridge_target	-0.396256008
PEX14	9.532830275	9.109542608	3_EWS-FLI1_bridge_target	-0.059175429
TARDBP	55.94491826	43.27423952	4_EWS-FLI1_loop_and_bridge_target	-0.363099575
DHRS3	4.871569543	8.772673856	4_EWS-FLI1_loop_and_bridge_target	0.73500714
PRDM2	3.779954326	2.996627028	4_EWS-FLI1_loop_and_bridge_target	-0.258213889
RCC2	103.1124468	66.65772603	4_EWS-FLI1_loop_and_bridge_target	-0.621815961
PAX7	24.04676074	2.139931539	4_EWS-FLI1_loop_and_bridge_target	-2.995819025
ALDH4A1	31.35681893	8.21845503	4_EWS-FLI1_loop_and_bridge_target	-1.811472893
UBR4	16.0862854	20.12568018	4_EWS-FLI1_loop_and_bridge_target	0.306159007
LOC101927895	1.071226495	0.668248377	4_EWS-FLI1_loop_and_bridge_target	-0.312151225
OTUD3	18.98814261	14.18298615	2_EWS-FLI1_loop_target	-0.396688852
RAP1GAP	9.819220105	2.52162867	1_EWS-FLI1_in_promoter	-1.619281807
LOC101928043	0.695699215	0.387793555	4_EWS-FLI1_loop_and_bridge_target	-0.289087314
LINC00339	20.01667557	11.44379594	4_EWS-FLI1_loop_and_bridge_target	-0.756107839
CDC42	113.9548489	132.4268562	4_EWS-FLI1_loop_and_bridge_target	0.214981761
TCEA3	31.18398375	14.62374532	3_EWS-FLI1_bridge_target	-1.042602577
E2F2	12.10956051	1.452364018	3_EWS-FLI1_bridge_target	-2.418374274
ID3	88.44495056	142.7876698	4_EWS-FLI1_loop_and_bridge_target	0.68486802
TCEB3-AS1	1.845204336	0.590211123	3_EWS-FLI1_bridge_target	-0.83931395
PITHD1	94.92556601	64.0744627	3_EWS-FLI1_bridge_target	-0.559823878
LYPLA2	32.92709053	17.14477768	4_EWS-FLI1_loop_and_bridge_target	-0.902883335
HMGCL	17.55849389	29.56544378	4_EWS-FLI1_loop_and_bridge_target	0.71982188
FUCA1	56.94638514	17.95272503	1_EWS-FLI1_in_promoter	-1.61231337
GRHL3	1.790702789	0.305613299	4_EWS-FLI1_loop_and_bridge_target	-1.095900827
SRRM1	8.85666212	5.902778573	3_EWS-FLI1_bridge_target	-0.513921966
CLIC4	210.1264763	297.2961479	4_EWS-FLI1_loop_and_bridge_target	0.498637836
RUNX3	6.211436819	4.746460883	4_EWS-FLI1_loop_and_bridge_target	-0.327613027
SYF2	27.2682884	29.32238662	3_EWS-FLI1_bridge_target	0.101198777
RSRP1	16.14558261	10.20250483	4_EWS-FLI1_loop_and_bridge_target	-0.61401558
MTFR1L	12.64442939	16.50724477	3_EWS-FLI1_bridge_target	0.359639993
SNHG3	53.62514111	23.29089053	4_EWS-FLI1_loop_and_bridge_target	-1.16914972
RCC1	64.31403087	34.18352961	4_EWS-FLI1_loop_and_bridge_target	-0.892492726
SNORA73B	521.8935062	527.0315226	4_EWS-FLI1_loop_and_bridge_target	0.014106905
TRNAU1AP	21.98187566	11.35719866	4_EWS-FLI1_loop_and_bridge_target	-0.895144822
SNHG12	25.58002497	16.68232631	3_EWS-FLI1_bridge_target	-0.58803437
SNORA61	32.37320347	20.53760091	3_EWS-FLI1_bridge_target	-0.63183262
SNORA44	29.51777481	19.01470382	3_EWS-FLI1_bridge_target	-0.608589506
SNORA16A	45.03947817	29.19817118	3_EWS-FLI1_bridge_target	-0.6084103
TAF12	35.09223361	30.75059444	4_EWS-FLI1_loop_and_bridge_target	-0.184904826
RNU11	99.43198208	68.26237244	4_EWS-FLI1_loop_and_bridge_target	-0.536075052
GMEB1	9.244336657	10.59098825	3_EWS-FLI1_bridge_target	0.178177006
YTHDF2	80.27998827	44.69556381	4_EWS-FLI1_loop_and_bridge_target	-0.830846081
EPB41	101.2324126	13.23833246	4_EWS-FLI1_loop_and_bridge_target	-2.844000574
SRSF4	34.31541288	36.53643728	4_EWS-FLI1_loop_and_bridge_target	0.087993762
ZBTB8OS	33.32163716	27.1929489	3_EWS-FLI1_bridge_target	-0.283783984
RBBP4	34.76344249	33.30788646	3_EWS-FLI1_bridge_target	-0.059945362
YARS	49.35210965	50.90001063	3_EWS-FLI1_bridge_target	0.043682609
S100PBP	21.46982485	21.26189834	3_EWS-FLI1_bridge_target	-0.013412258
ZNF362	6.037776657	5.387295351	4_EWS-FLI1_loop_and_bridge_target	-0.139914568
PHC2	29.85313374	32.46980026	4_EWS-FLI1_loop_and_bridge_target	0.117442911
LOC101929464	0.802251398	0.150656717	4_EWS-FLI1_loop_and_bridge_target	-0.647342779

ZSCAN20	2.606763234	4.217888028	3_EWS-FLI1_bridge_target	0.532761265
PSMB2	34.13910134	24.2847537	3_EWS-FLI1_bridge_target	-0.474809571
AGO3	11.39691555	11.31799114	4_EWS-FLI1_loop_and_bridge_target	-0.009214216
TRAPPC3	63.36927287	40.46231477	4_EWS-FLI1_loop_and_bridge_target	-0.63457151
MAP7D1	26.54273733	33.76177228	4_EWS-FLI1_loop_and_bridge_target	0.335829689
THRAP3	83.26715219	66.35663606	4_EWS-FLI1_loop_and_bridge_target	-0.323150281
EVA1B	5.813432407	6.265463946	4_EWS-FLI1_loop_and_bridge_target	0.092673157
STK40	17.80863375	19.84822273	4_EWS-FLI1_loop_and_bridge_target	0.148529346
LSM10	22.02615092	9.609309043	4_EWS-FLI1_loop_and_bridge_target	-1.117942568
MRPS15	116.9964219	74.08953872	4_EWS-FLI1_loop_and_bridge_target	-0.652059277
GRIK3	23.54134328	3.371187128	2_EWS-FLI1_loop_target	-2.489117172
SNIP1	10.42952437	7.10431209	3_EWS-FLI1_bridge_target	-0.496003731
MTF1	2.812147943	4.498881954	4_EWS-FLI1_loop_and_bridge_target	0.528534207
SF3A3	102.0292522	79.35993291	4_EWS-FLI1_loop_and_bridge_target	-0.358505743
UTP11	57.81493184	39.85176375	4_EWS-FLI1_loop_and_bridge_target	-0.525784103
CDC20	108.8845265	50.99318877	3_EWS-FLI1_bridge_target	-1.079593702
KDM4A	27.06485146	32.35126627	4_EWS-FLI1_loop_and_bridge_target	0.248977115
KDM4A-AS1	2.927521834	0.820242125	4_EWS-FLI1_loop_and_bridge_target	-1.109488928
ST3GAL3	9.386218248	7.104010061	4_EWS-FLI1_loop_and_bridge_target	-0.357962576
ARTN	6.268620748	0.335438524	4_EWS-FLI1_loop_and_bridge_target	-2.444368069
IPO13	12.38298863	11.31983018	4_EWS-FLI1_loop_and_bridge_target	-0.119417959
SLC6A9	4.717163787	2.445544856	4_EWS-FLI1_loop_and_bridge_target	-0.730567482
DMRTA2	0.127506709	0.079815971	2_EWS-FLI1_loop_target	-0.062350559
FAF1	61.34096294	59.0936342	3_EWS-FLI1_bridge_target	-0.052968267
CDKN2C	37.42440116	22.92407926	4_EWS-FLI1_loop_and_bridge_target	-0.683559374
C1orf185	0.101531138	0.135056332	4_EWS-FLI1_loop_and_bridge_target	0.043253622
RNF11	26.08000196	70.83593918	4_EWS-FLI1_loop_and_bridge_target	1.407477955
OSBPL9	38.37027261	47.08494164	3_EWS-FLI1_bridge_target	0.288478466
NRDC	58.82895605	59.94780365	4_EWS-FLI1_loop_and_bridge_target	0.026730342
TXNDC12	22.93500597	29.78893126	4_EWS-FLI1_loop_and_bridge_target	0.363289624
BTF3L4	18.09855411	6.598329396	4_EWS-FLI1_loop_and_bridge_target	-1.329709259
PARS2	5.727626407	3.748843411	3_EWS-FLI1_bridge_target	-0.502521408
OMA1	12.60804515	5.394767237	2_EWS-FLI1_loop_target	-1.089496081
JUN	27.9356488	38.35859955	4_EWS-FLI1_loop_and_bridge_target	0.443830895
LINC01135	0.051477553	0.197208703	4_EWS-FLI1_loop_and_bridge_target	0.18725662
FGGY	1.188261612	0.799037884	4_EWS-FLI1_loop_and_bridge_target	-0.282559659
NFIA	2.28745537	3.906031792	4_EWS-FLI1_loop_and_bridge_target	0.577585274
ATG4C	16.31138831	6.549290099	4_EWS-FLI1_loop_and_bridge_target	-1.197308537
ALG6	36.86692843	11.32182323	4_EWS-FLI1_loop_and_bridge_target	-1.619722659
ITGB3BP	45.43141801	19.80508091	4_EWS-FLI1_loop_and_bridge_target	-1.158165442
EFCAB7	19.37886838	6.254260836	4_EWS-FLI1_loop_and_bridge_target	-1.490173417
PGM1	0.965499656	0.579771499	4_EWS-FLI1_loop_and_bridge_target	-0.315180212
RAVER2	26.7876673	12.75503778	2_EWS-FLI1_loop_target	-1.014484627
JAK1	124.5595331	115.9996829	4_EWS-FLI1_loop_and_bridge_target	-0.10186695
LINC01359	0.492905918	0.228056198	4_EWS-FLI1_loop_and_bridge_target	-0.281746668
LEPROT	10.60457895	30.43352959	4_EWS-FLI1_loop_and_bridge_target	1.437610099
LEPR	2.006672852	3.710194029	4_EWS-FLI1_loop_and_bridge_target	0.64761859
PDE4B	5.59770632	3.610779752	4_EWS-FLI1_loop_and_bridge_target	-0.516953809
WDR78	2.382532132	4.109506376	4_EWS-FLI1_loop_and_bridge_target	0.595080282
MIER1	12.900348	13.25532919	4_EWS-FLI1_loop_and_bridge_target	0.036380353
LRRC40	36.15387227	34.06163353	4_EWS-FLI1_loop_and_bridge_target	-0.083619367
SRSF11	76.42338698	103.5849466	4_EWS-FLI1_loop_and_bridge_target	0.433833887
ANKRD13C	17.15406834	19.79393186	4_EWS-FLI1_loop_and_bridge_target	0.195869686
HHLA3	2.164049203	2.289950413	4_EWS-FLI1_loop_and_bridge_target	0.056293805
CTH	3.598223216	3.173647102	4_EWS-FLI1_loop_and_bridge_target	-0.139767882
ZRANB2-AS1	3.17330242	1.064455026	4_EWS-FLI1_loop_and_bridge_target	-1.01542848

ZRANB2	54.36095386	24.58379102	4_EWS-FLI1_loop_and_bridge_target	-1.113638741
ZRANB2-AS2	0.44298308	0.298555563	4_EWS-FLI1_loop_and_bridge_target	-0.152146637
CRYZ	23.88680946	15.67470948	4_EWS-FLI1_loop_and_bridge_target	-0.577719659
TYW3	32.00768064	15.89869141	4_EWS-FLI1_loop_and_bridge_target	-0.965890236
ZZZ3	35.92647998	45.0490991	4_EWS-FLI1_loop_and_bridge_target	0.318517188
USP33	63.38898227	44.13079985	4_EWS-FLI1_loop_and_bridge_target	-0.512701499
FUBP1	111.5983437	52.22574998	4_EWS-FLI1_loop_and_bridge_target	-1.080989328
DNAJB4	21.26910536	51.20462678	4_EWS-FLI1_loop_and_bridge_target	1.229134075
GTF2B	52.76986792	31.04703705	3_EWS-FLI1_bridge_target	-0.746606951
KYAT3	48.10635685	24.7959347	4_EWS-FLI1_loop_and_bridge_target	-0.928766072
RBMXL1	30.62631054	8.549899489	4_EWS-FLI1_loop_and_bridge_target	-1.727567809
GBP3	0.387567487	16.75279452	3_EWS-FLI1_bridge_target	3.677416296
GBP4	2.344357289	1.053166862	4_EWS-FLI1_loop_and_bridge_target	-0.703878103
GBP6	9.841951772	1.253873313	4_EWS-FLI1_loop_and_bridge_target	-2.266146164
GEMIN8P4	3.213136906	0.571085146	4_EWS-FLI1_loop_and_bridge_target	-1.423133427
ZNF326	5.87237298	8.854140907	4_EWS-FLI1_loop_and_bridge_target	0.519921764
ZNF644	60.29275058	44.92537607	2_EWS-FLI1_loop_target	-0.416424913
PTBP2	31.94944847	12.76924366	4_EWS-FLI1_loop_and_bridge_target	-1.258805001
OLFM3	1.829342926	12.22981009	4_EWS-FLI1_loop_and_bridge_target	2.2252534
PRMT6	32.95784084	10.64612374	4_EWS-FLI1_loop_and_bridge_target	-1.543894878
NTNG1	5.35426654	5.757325627	4_EWS-FLI1_loop_and_bridge_target	0.088726771
AMPD2	21.57793603	25.06104398	3_EWS-FLI1_bridge_target	0.20698127
STRIP1	24.3775331	28.14037726	4_EWS-FLI1_loop_and_bridge_target	0.19946772
LOC440600	0.518488858	0.179201998	4_EWS-FLI1_loop_and_bridge_target	-0.364825449
RBM15	43.57516489	20.92083602	4_EWS-FLI1_loop_and_bridge_target	-1.023937313
LAMTOR5	36.55346972	34.93767167	2_EWS-FLI1_loop_target	-0.063449268
LAMTOR5-AS1	0.412148798	0.461674742	2_EWS-FLI1_loop_target	0.0497302
KCNA2	15.97340435	0.584640664	1_EWS-FLI1_in_promoter	-3.42104832
ST7L	6.806887946	7.363769705	4_EWS-FLI1_loop_and_bridge_target	0.099405776
CAPZA1	138.0798145	177.7156347	4_EWS-FLI1_loop_and_bridge_target	0.361752804
RHOC	28.84401931	140.9870217	4_EWS-FLI1_loop_and_bridge_target	2.250245315
LOC100996251	0.169473494	0.280538838	4_EWS-FLI1_loop_and_bridge_target	0.130891846
LRIG2	12.58172971	10.55920559	4_EWS-FLI1_loop_and_bridge_target	-0.232624975
PHTF1	11.56728521	11.20349488	4_EWS-FLI1_loop_and_bridge_target	-0.04237866
RSBN1	13.65668389	7.847011401	4_EWS-FLI1_loop_and_bridge_target	-0.72829664
AP4B1-AS1	0.699740032	0.406684294	1_EWS-FLI1_in_promoter	-0.273015533
PTPN22	8.796378298	0.912502529	4_EWS-FLI1_loop_and_bridge_target	-2.356786831
AP4B1	13.77187155	5.329569775	4_EWS-FLI1_loop_and_bridge_target	-1.222673276
DCLRE1B	18.23720674	9.156338734	4_EWS-FLI1_loop_and_bridge_target	-0.921518916
HIPK1-AS1	0.055756441	0.16181892	4_EWS-FLI1_loop_and_bridge_target	0.13810818
HIPK1	21.88620899	12.88538441	4_EWS-FLI1_loop_and_bridge_target	-0.720911387
OLFML3	229.1797806	54.29203202	4_EWS-FLI1_loop_and_bridge_target	-2.057617612
TRIM33	23.65473868	15.10252739	4_EWS-FLI1_loop_and_bridge_target	-0.614577815
BCAS2	97.53374	68.67715198	4_EWS-FLI1_loop_and_bridge_target	-0.499932162
DENND2C	58.21398257	7.103071485	4_EWS-FLI1_loop_and_bridge_target	-2.869397115
NRAS	112.5295377	66.56226175	4_EWS-FLI1_loop_and_bridge_target	-0.748778171
CSDE1	310.2427531	339.8746371	4_EWS-FLI1_loop_and_bridge_target	0.131201013
SIKE1	37.95029583	39.57413453	3_EWS-FLI1_bridge_target	0.058926036
ATP1A1	117.1807791	83.12104795	4_EWS-FLI1_loop_and_bridge_target	-0.490456687
CD58	13.63344208	10.04903965	4_EWS-FLI1_loop_and_bridge_target	-0.40534818
PFDN2	63.64424848	49.65531185	3_EWS-FLI1_bridge_target	-0.351808455
NIT1	12.33964297	3.795801325	3_EWS-FLI1_bridge_target	-1.475876255
DEDD	12.89555387	13.04104167	3_EWS-FLI1_bridge_target	0.015026629
PPOX	8.344418153	2.331287715	3_EWS-FLI1_bridge_target	-1.488024873
ADAMTS4	16.0387886	21.75838878	4_EWS-FLI1_loop_and_bridge_target	0.417575655
NDUFS2	78.68162262	31.07545881	4_EWS-FLI1_loop_and_bridge_target	-1.312777125

DUSP12	38.03433947	46.12413333	4_EWS-FLI1_loop_and_bridge_target	0.271722229
ATF6	20.31215963	19.94734064	4_EWS-FLI1_loop_and_bridge_target	-0.024909695
UHMK1	57.20435753	30.97508248	4_EWS-FLI1_loop_and_bridge_target	-0.864179085
UAP1	137.2640127	90.84942732	4_EWS-FLI1_loop_and_bridge_target	-0.59008307
DDR2	93.72803691	56.73172802	4_EWS-FLI1_loop_and_bridge_target	-0.714427079
HSD17B7	18.04020099	12.81012788	4_EWS-FLI1_loop_and_bridge_target	-0.463322029
RGS5	1.80498301	11.57608684	2_EWS-FLI1_loop_target	2.164619147
LOC101928404	0.02634681	0.126167133	4_EWS-FLI1_loop_and_bridge_target	0.133902641
NUF2	32.83126	21.30402625	2_EWS-FLI1_loop_target	-0.601052745
PBX1	11.88846455	10.59256617	4_EWS-FLI1_loop_and_bridge_target	-0.152880439
LOC100505795	0.047119138	0.300852945	4_EWS-FLI1_loop_and_bridge_target	0.313032285
MGST3	59.86574893	51.9702817	4_EWS-FLI1_loop_and_bridge_target	-0.200447426
ALDH9A1	67.70161794	71.51362995	3_EWS-FLI1_bridge_target	0.07790812
LOC440700	0.133773568	0.183029235	3_EWS-FLI1_bridge_target	0.061353185
UCK2	23.72295942	25.50502877	4_EWS-FLI1_loop_and_bridge_target	0.100414658
CACYBP	70.4942026	25.98051981	4_EWS-FLI1_loop_and_bridge_target	-1.405908116
MRPS14	34.34445639	15.29566395	4_EWS-FLI1_loop_and_bridge_target	-1.116995818
LOC284648	1.070829837	0.495448264	2_EWS-FLI1_loop_target	-0.46963101
NMNAT2	0.084232099	0.10670973	4_EWS-FLI1_loop_and_bridge_target	0.029603255
SMG7-AS1	0.381626132	0.245671541	4_EWS-FLI1_loop_and_bridge_target	-0.149443567
SMG7	10.50766655	5.993869383	4_EWS-FLI1_loop_and_bridge_target	-0.718432566
ARPC5	123.9768208	126.1092777	4_EWS-FLI1_loop_and_bridge_target	0.02440879
RGL1	18.12518474	40.20555469	4_EWS-FLI1_loop_and_bridge_target	1.107365149
APOBEC4	0.162315506	0.186016033	4_EWS-FLI1_loop_and_bridge_target	0.029121776
COLGALT2	14.02203847	8.505929066	4_EWS-FLI1_loop_and_bridge_target	-0.660181056
TSEN15	43.3688313	45.90438489	4_EWS-FLI1_loop_and_bridge_target	0.080176248
EDEM3	27.00701	34.45714849	4_EWS-FLI1_loop_and_bridge_target	0.340288547
TRMT1L	10.9864507	4.022288857	4_EWS-FLI1_loop_and_bridge_target	-1.254987614
SWT1	6.588354282	4.721850106	4_EWS-FLI1_loop_and_bridge_target	-0.407305332
IVNS1ABP	66.51994147	57.49023704	4_EWS-FLI1_loop_and_bridge_target	-0.207117818
HMCN1	16.76888465	58.79895678	4_EWS-FLI1_loop_and_bridge_target	1.750767189
TPR	76.666104	60.14911733	4_EWS-FLI1_loop_and_bridge_target	-0.344953423
C1orf27	23.04359095	22.94614714	4_EWS-FLI1_loop_and_bridge_target	-0.005858831
ZBTB41	6.610249407	8.792209839	3_EWS-FLI1_bridge_target	0.363690739
CRB1	0.049683904	0.160817391	3_EWS-FLI1_bridge_target	0.145186091
C1orf53	6.838932337	9.925770376	4_EWS-FLI1_loop_and_bridge_target	0.47900593
LHX9	6.203814284	19.88209463	3_EWS-FLI1_bridge_target	1.535433539
PTPRC	28.10241047	10.82852349	3_EWS-FLI1_bridge_target	-1.298868654
ZNF281	27.69880796	29.9237411	4_EWS-FLI1_loop_and_bridge_target	0.10772405
CSRP1	119.4891199	195.0316243	4_EWS-FLI1_loop_and_bridge_target	0.702183535
RPS10P7	0.604102905	0.28571569	1_EWS-FLI1_in_promoter	-0.319195041
NAV1	1.617701406	2.664782088	4_EWS-FLI1_loop_and_bridge_target	0.485426874
IPO9-AS1	0.096119963	0.321472567	4_EWS-FLI1_loop_and_bridge_target	0.269740776
IPO9	36.07713028	37.39049587	4_EWS-FLI1_loop_and_bridge_target	0.050219609
TIMM17A	99.8752215	80.40746044	4_EWS-FLI1_loop_and_bridge_target	-0.309338923
RNPEP	12.98498589	14.50806993	4_EWS-FLI1_loop_and_bridge_target	0.149140348
TRAF3IP3	0.750526347	0.487193473	4_EWS-FLI1_loop_and_bridge_target	-0.235196433
AIDA	3.334707657	3.3972104	3_EWS-FLI1_bridge_target	0.020653867
BROX	17.04214868	21.87703959	3_EWS-FLI1_bridge_target	0.342529209
CAPN2	34.5863051	107.8837376	4_EWS-FLI1_loop_and_bridge_target	1.613394443
TP53BP2	31.59050259	27.19953033	4_EWS-FLI1_loop_and_bridge_target	-0.208780467
FBXO28	27.25128536	38.24711837	4_EWS-FLI1_loop_and_bridge_target	0.474270224
NVL	33.75478016	27.31684603	3_EWS-FLI1_bridge_target	-0.295550834
WDR26	12.50403543	18.66671508	3_EWS-FLI1_bridge_target	0.54236541
CNIH3	0.617074795	0.759047591	3_EWS-FLI1_bridge_target	0.121408106
C1orf198	10.46499282	46.58526641	4_EWS-FLI1_loop_and_bridge_target	2.053279496

LOC101927765	2.288284471	0.720915931	2_EWS-FLI1_loop_target	-0.934158492
COA6	38.31904318	12.19756609	2_EWS-FLI1_loop_target	-1.574956324
IRF2BP2	13.97192193	43.08777796	3_EWS-FLI1_bridge_target	1.558119335
GPR137B	60.02314432	14.3401782	1_EWS-FLI1_in_promoter	-1.992041277
ERO1B	4.282508684	5.818298417	4_EWS-FLI1_loop_and_bridge_target	0.368188509
LGALS8	1.483800716	0.759984458	4_EWS-FLI1_loop_and_bridge_target	-0.496986737
HEATR1	67.19478445	34.37736843	4_EWS-FLI1_loop_and_bridge_target	-0.94683467
ACTN2	4.341919028	0.520075242	4_EWS-FLI1_loop_and_bridge_target	-1.813215371
MTR	20.41229781	13.86751462	4_EWS-FLI1_loop_and_bridge_target	-0.526276128
FH	122.0590406	80.91928006	4_EWS-FLI1_loop_and_bridge_target	-0.587075708
KMO	25.95599882	1.065411344	4_EWS-FLI1_loop_and_bridge_target	-3.706105328
EXO1	56.75062377	14.85178441	2_EWS-FLI1_loop_target	-1.865191278
CEP170	16.74818687	15.28999998	4_EWS-FLI1_loop_and_bridge_target	-0.123685047
SDCCAG8	7.108532723	8.236898379	4_EWS-FLI1_loop_and_bridge_target	0.18796762
LINC01304	0.086139673	0.010185126	2_EWS-FLI1_loop_target	-0.104589936
LINC01249	1.673313087	0.142637468	2_EWS-FLI1_loop_target	-1.226261067
LINC01248	0.032939767	0.122685788	4_EWS-FLI1_loop_and_bridge_target	0.120198079
SOX11	26.19220401	9.73819975	4_EWS-FLI1_loop_and_bridge_target	-1.340440944
CMPK2	9.288490565	4.992606287	4_EWS-FLI1_loop_and_bridge_target	-0.779775841
ID2-AS1	1.639093718	0.727423881	4_EWS-FLI1_loop_and_bridge_target	-0.611420444
ID2	272.1868288	99.51148601	4_EWS-FLI1_loop_and_bridge_target	-1.442527553
PDIA6	132.077112	143.6921286	3_EWS-FLI1_bridge_target	0.120723978
ROCK2	32.95157076	21.09340022	3_EWS-FLI1_bridge_target	-0.619862852
E2F6	18.03314781	12.19307751	3_EWS-FLI1_bridge_target	-0.528733046
TRIB2	28.7235819	10.94928293	4_EWS-FLI1_loop_and_bridge_target	-1.314683935
ATAD2B	10.37418	6.564341524	4_EWS-FLI1_loop_and_bridge_target	-0.588476134
WDCP	28.43552073	12.20884273	3_EWS-FLI1_bridge_target	-1.156054077
FKBP1B	6.871670548	1.04464799	3_EWS-FLI1_bridge_target	-1.944817353
PTRHD1	43.19526462	29.03829203	2_EWS-FLI1_loop_target	-0.557089014
CENPO	13.52229074	13.46325265	2_EWS-FLI1_loop_target	-0.005877003
ASXL2	4.436826892	3.797078476	3_EWS-FLI1_bridge_target	-0.180608853
HADHA	81.53832164	89.26513826	3_EWS-FLI1_bridge_target	0.129104802
HADHB	71.41776723	63.83152393	3_EWS-FLI1_bridge_target	-0.159648211
ADGRF3	0.890307611	0.14642054	4_EWS-FLI1_loop_and_bridge_target	-0.72148466
EPT1	24.87314299	20.92631497	4_EWS-FLI1_loop_and_bridge_target	-0.238791952
MAPRE3	0.959048161	2.092699851	4_EWS-FLI1_loop_and_bridge_target	0.658713961
AGBL5-AS1	0.056815029	0.060460108	3_EWS-FLI1_bridge_target	0.004967462
AGBL5	29.48866369	13.76277417	3_EWS-FLI1_bridge_target	-1.046309066
OST4	102.9362979	80.74734078	3_EWS-FLI1_bridge_target	-0.346455872
EMILIN1	20.10387227	41.87052345	3_EWS-FLI1_bridge_target	1.022478295
ABHD1	0.615672957	0.995522082	3_EWS-FLI1_bridge_target	0.304631045
PREB	57.10938274	25.97180704	3_EWS-FLI1_bridge_target	-1.107318948
SLC5A6	78.65207601	33.95447356	4_EWS-FLI1_loop_and_bridge_target	-1.188234857
ATRAID	99.73963669	51.18592491	4_EWS-FLI1_loop_and_bridge_target	-0.948898779
CAD	59.21511247	33.08871031	4_EWS-FLI1_loop_and_bridge_target	-0.820831594
GTF3C2	22.82698713	17.40101191	3_EWS-FLI1_bridge_target	-0.372811432
CCDC121	1.491983835	0.605664017	3_EWS-FLI1_bridge_target	-0.634124668
GPN1	51.58503092	44.10936387	3_EWS-FLI1_bridge_target	-0.221225233
PPP1CB	65.78091335	77.67190739	4_EWS-FLI1_loop_and_bridge_target	0.236412737
TRMT61B	17.82897532	8.031073876	4_EWS-FLI1_loop_and_bridge_target	-1.059985038
WDR43	102.8484769	67.6612642	4_EWS-FLI1_loop_and_bridge_target	-0.596911731
YPEL5	215.0761902	69.98542766	4_EWS-FLI1_loop_and_bridge_target	-1.605945313
LBH	457.5788445	126.2608922	4_EWS-FLI1_loop_and_bridge_target	-1.84938066
LCLAT1	22.09027813	15.87442326	4_EWS-FLI1_loop_and_bridge_target	-0.452447357
DPY30	52.96863433	34.10366638	3_EWS-FLI1_bridge_target	-0.620499461
SPAST	44.24467924	12.0298507	4_EWS-FLI1_loop_and_bridge_target	-1.795927588

SLC30A6	17.22751829	25.9479012	4_EWS-FLI1_loop_and_bridge_target	0.564054766
YIPF4	35.60346314	46.23410576	2_EWS-FLI1_loop_target	0.367848793
RASGRP3	1.275670753	8.089295097	2_EWS-FLI1_loop_target	1.997876572
FAM98A	24.0854924	39.69703625	4_EWS-FLI1_loop_and_bridge_target	0.698070478
LOC100288911	1.098053589	1.804303558	4_EWS-FLI1_loop_and_bridge_target	0.418590997
CRIM1	17.52514802	84.85142387	4_EWS-FLI1_loop_and_bridge_target	2.212356992
CYP1B1	13.19528219	21.11172387	3_EWS-FLI1_bridge_target	0.639399975
HNRNPLL	34.83414598	34.90826166	4_EWS-FLI1_loop_and_bridge_target	0.002980841
GALM	5.825165057	2.794703335	3_EWS-FLI1_bridge_target	-0.846876834
SRSF7	149.9511251	78.95283585	4_EWS-FLI1_loop_and_bridge_target	-0.916860399
GEMIN6	65.49492031	24.5361445	4_EWS-FLI1_loop_and_bridge_target	-1.380703414
SOS1	6.159003906	4.623846269	4_EWS-FLI1_loop_and_bridge_target	-0.348201709
LOC388942	0	0.054380693	4_EWS-FLI1_loop_and_bridge_target	0.076395859
LOC101929723	0.141626328	0.931678138	4_EWS-FLI1_loop_and_bridge_target	0.758764217
COX7A2L	43.84227005	11.18978784	3_EWS-FLI1_bridge_target	-1.879186297
SLC1A4	47.82757031	5.302046472	4_EWS-FLI1_loop_and_bridge_target	-2.953803692
ACTR2	74.35769729	97.70402519	4_EWS-FLI1_loop_and_bridge_target	0.389354038
SPRED2	5.037489265	3.18836052	3_EWS-FLI1_bridge_target	-0.527563089
MEIS1-AS3	1.650082253	0.675364034	4_EWS-FLI1_loop_and_bridge_target	-0.661562531
MEIS1	94.46848828	28.90831332	4_EWS-FLI1_loop_and_bridge_target	-1.674478063
MEIS1-AS2	6.844752109	2.141288288	4_EWS-FLI1_loop_and_bridge_target	-1.320371509
LOC100507073	1.22419677	0.916741199	4_EWS-FLI1_loop_and_bridge_target	-0.21462887
LOC101927577	0.147884235	0.028613097	3_EWS-FLI1_bridge_target	-0.158276724
ETAA1	30.56866608	19.77275718	4_EWS-FLI1_loop_and_bridge_target	-0.603800583
LOC101927701	0.558158681	0.10182316	4_EWS-FLI1_loop_and_bridge_target	-0.499949469
C1D	13.93906291	11.8820121	3_EWS-FLI1_bridge_target	-0.213731702
WDR92	10.73202176	7.446711636	3_EWS-FLI1_bridge_target	-0.473989947
PNO1	50.30371398	29.25295617	3_EWS-FLI1_bridge_target	-0.761987147
PPP3R1	31.97462463	22.05745762	3_EWS-FLI1_bridge_target	-0.51612279
GKN1	1.142086011	0.540159448	1_EWS-FLI1_in_promoter	-0.475936693
ANTXR1	9.584834163	29.1863636	4_EWS-FLI1_loop_and_bridge_target	1.511898307
PCBP1-AS1	3.454989533	2.480321431	3_EWS-FLI1_bridge_target	-0.356201487
PCBP1	45.02517993	43.63922462	3_EWS-FLI1_bridge_target	-0.044111394
ZNF638	57.45770323	47.98056679	4_EWS-FLI1_loop_and_bridge_target	-0.255183679
CYP26B1	4.864811791	2.057806737	4_EWS-FLI1_loop_and_bridge_target	-0.939587584
SPR	58.68098788	11.51937022	2_EWS-FLI1_loop_target	-2.253109426
SMYD5	48.13462428	29.97063115	4_EWS-FLI1_loop_and_bridge_target	-0.66583924
PRADC1	36.54315904	16.84790959	4_EWS-FLI1_loop_and_bridge_target	-1.07279494
CCT7	373.592549	205.2926373	4_EWS-FLI1_loop_and_bridge_target	-0.860629872
ALMS1	14.63082404	7.972319281	4_EWS-FLI1_loop_and_bridge_target	-0.800840973
TPRKB	98.41416955	44.06074556	2_EWS-FLI1_loop_target	-1.14158031
GCFC2	23.82681837	11.843384	2_EWS-FLI1_loop_target	-0.950874011
IWS1	57.49326778	51.15029894	4_EWS-FLI1_loop_and_bridge_target	-0.165595066
LIMS2	0.804465874	1.68030183	1_EWS-FLI1_in_promoter	0.570823613
GPR17	0.760707112	0.260369278	1_EWS-FLI1_in_promoter	-0.482308448
FAM201B	1.880500157	0.426099805	4_EWS-FLI1_loop_and_bridge_target	-1.014244384
GPR39	5.77068295	3.10098627	4_EWS-FLI1_loop_and_bridge_target	-0.72333045
NCKAP5	2.710048449	14.02588112	4_EWS-FLI1_loop_and_bridge_target	2.017939661
HNMT	5.551241722	20.55807903	4_EWS-FLI1_loop_and_bridge_target	1.718388344
LRP1B	2.080029449	2.138305874	4_EWS-FLI1_loop_and_bridge_target	0.027041826
GTDC1	13.55510757	6.096564144	4_EWS-FLI1_loop_and_bridge_target	-1.036332895
ZEB2	14.05059403	10.21150779	4_EWS-FLI1_loop_and_bridge_target	-0.424840116
ZEB2-AS1	1.778747443	0.803034249	4_EWS-FLI1_loop_and_bridge_target	-0.624007915
LINC01412	0.218663988	0.0581732	4_EWS-FLI1_loop_and_bridge_target	-0.203724614
LOC105373656	0.514622777	0.073018587	4_EWS-FLI1_loop_and_bridge_target	-0.497283462
NMI	21.60715116	18.18508404	4_EWS-FLI1_loop_and_bridge_target	-0.236794118

TNFAIP6	39.28108908	28.05469994	4_EWS-FLI1_loop_and_bridge_target	-0.471331134
RIF1	46.17473887	39.61481789	4_EWS-FLI1_loop_and_bridge_target	-0.216008356
NR4A2	0.225932662	0.160285207	4_EWS-FLI1_loop_and_bridge_target	-0.079400262
FAP	4.486133852	42.10880474	4_EWS-FLI1_loop_and_bridge_target	2.974120835
IFIH1	8.986133265	7.177118598	2_EWS-FLI1_loop_target	-0.288333595
CSRNP3	4.529674916	2.630361168	2_EWS-FLI1_loop_target	-0.607081586
SCN1A	0.408864568	0.081119606	4_EWS-FLI1_loop_and_bridge_target	-0.382006794
LOC101929680	0.095345186	0.048061062	4_EWS-FLI1_loop_and_bridge_target	-0.063662818
STK39	9.601922095	36.05783381	4_EWS-FLI1_loop_and_bridge_target	1.805452708
DYNC1I2	36.34186706	36.07308945	4_EWS-FLI1_loop_and_bridge_target	-0.010421717
DLX2	0.105381522	1.153465489	4_EWS-FLI1_loop_and_bridge_target	0.962115803
DLX2-AS1	0.085192641	0.09065834	4_EWS-FLI1_loop_and_bridge_target	0.007248064
ATF2	31.21844737	27.27237446	4_EWS-FLI1_loop_and_bridge_target	-0.188493918
ATP5G3	66.05068551	44.54324627	4_EWS-FLI1_loop_and_bridge_target	-0.558014953
KIAA1715	13.54857151	29.36601894	4_EWS-FLI1_loop_and_bridge_target	1.061580274
HOXD13	45.98409941	17.82361112	4_EWS-FLI1_loop_and_bridge_target	-1.319629174
HOXD11	17.496893	12.58115741	4_EWS-FLI1_loop_and_bridge_target	-0.445676521
HOXD8	13.21983503	3.29937637	4_EWS-FLI1_loop_and_bridge_target	-1.725705412
HOXD-AS2	7.568673189	2.275816302	4_EWS-FLI1_loop_and_bridge_target	-1.387217371
HNRNPA3	31.17800034	36.8558174	4_EWS-FLI1_loop_and_bridge_target	0.234440346
NFE2L2	21.33653218	28.7508139	4_EWS-FLI1_loop_and_bridge_target	0.413523917
TTC30B	5.194965113	2.745515435	4_EWS-FLI1_loop_and_bridge_target	-0.72593189
PDE11A	0.159433185	0.612039706	2_EWS-FLI1_loop_target	0.475467596
UBE2E3	39.76904364	32.34617194	4_EWS-FLI1_loop_and_bridge_target	-0.289952961
SSFA2	14.969518	65.47622042	4_EWS-FLI1_loop_and_bridge_target	2.057517589
PPP1R1C	0.120069958	0.177636023	4_EWS-FLI1_loop_and_bridge_target	0.072304864
NCKAP1	98.39452719	191.9489242	4_EWS-FLI1_loop_and_bridge_target	0.956980678
CALCRL	13.0871596	6.420676784	4_EWS-FLI1_loop_and_bridge_target	-0.924758075
NABP1	10.62044212	8.97037703	4_EWS-FLI1_loop_and_bridge_target	-0.220944994
STK17B	18.84711454	39.62803463	4_EWS-FLI1_loop_and_bridge_target	1.033546299
GTF3C3	38.91780552	29.01655454	2_EWS-FLI1_loop_target	-0.411274024
PGAP1	4.723656197	6.677011158	4_EWS-FLI1_loop_and_bridge_target	0.423607729
SF3B1	172.8719613	137.7433742	4_EWS-FLI1_loop_and_bridge_target	-0.325606425
COQ10B	10.08849633	23.67647119	3_EWS-FLI1_bridge_target	1.154072359
HSPD1	370.2927205	186.8424689	4_EWS-FLI1_loop_and_bridge_target	-0.983033755
HSPE1	55.55272773	38.57634005	4_EWS-FLI1_loop_and_bridge_target	-0.514958412
MARS2	14.03752679	4.643255958	3_EWS-FLI1_bridge_target	-1.413967616
SGO2	26.23671499	10.07498	3_EWS-FLI1_bridge_target	-1.298248618
BZW1	34.51277563	52.68829751	4_EWS-FLI1_loop_and_bridge_target	0.596269533
CLK1	31.50713068	33.54474649	3_EWS-FLI1_bridge_target	0.087710106
CFLAR	4.307102699	5.195485029	4_EWS-FLI1_loop_and_bridge_target	0.223292765
CASP8	8.16728914	9.377253082	4_EWS-FLI1_loop_and_bridge_target	0.178857522
MPP4	0.333967431	8.984954934	4_EWS-FLI1_loop_and_bridge_target	2.904032473
ALS2	14.32722314	18.24967434	4_EWS-FLI1_loop_and_bridge_target	0.328737693
CDK15	0.535964201	0.429047092	4_EWS-FLI1_loop_and_bridge_target	-0.104091133
NOP58	137.6659884	93.84759487	4_EWS-FLI1_loop_and_bridge_target	-0.547930876
CYP20A1	4.344545524	16.67505843	4_EWS-FLI1_loop_and_bridge_target	1.725575807
ABI2	13.12079814	19.96964534	3_EWS-FLI1_bridge_target	0.570480825
RAPH1	1.093317339	3.592318384	4_EWS-FLI1_loop_and_bridge_target	1.133431632
IKZF2	5.771949804	7.291465803	4_EWS-FLI1_loop_and_bridge_target	0.292055891
FN1	34.05946317	694.2077683	4_EWS-FLI1_loop_and_bridge_target	4.309568392
LINC01614	0.255746176	2.579793782	4_EWS-FLI1_loop_and_bridge_target	1.5113316
XRCC5	361.5153789	242.7617769	3_EWS-FLI1_bridge_target	-0.572570278
IGFBP5	13.86162858	68.82855585	4_EWS-FLI1_loop_and_bridge_target	2.232224917
TNS1	7.140088876	5.149661417	4_EWS-FLI1_loop_and_bridge_target	-0.404537564
PNKD	7.026646109	7.214626332	4_EWS-FLI1_loop_and_bridge_target	0.03339766

TMBIM1	11.72004738	50.37792963	4_EWS-FLI1_loop_and_bridge_target	2.01404471
CTDSP1	29.38292427	21.73494107	4_EWS-FLI1_loop_and_bridge_target	-0.418349467
USP37	18.49436709	10.32144816	3_EWS-FLI1_bridge_target	-0.783998807
CNOT9	56.32584974	46.58350205	3_EWS-FLI1_bridge_target	-0.26872438
GLB1L	2.81153678	2.279122954	3_EWS-FLI1_bridge_target	-0.2170628
STK16	10.73363444	8.778190831	3_EWS-FLI1_bridge_target	-0.263010484
TUBA4B	0.083781582	0.115903779	3_EWS-FLI1_bridge_target	0.042138597
STK11IP	10.70684236	11.098392	3_EWS-FLI1_bridge_target	0.047463315
EPHA4	27.53625539	32.11177699	4_EWS-FLI1_loop_and_bridge_target	0.214548405
PAX3	4.19310064	5.183930233	4_EWS-FLI1_loop_and_bridge_target	0.251927858
CCDC140	0.279008943	0.325876065	4_EWS-FLI1_loop_and_bridge_target	0.051919576
FARSB	73.93367737	31.38897416	4_EWS-FLI1_loop_and_bridge_target	-1.210111478
KCNE4	11.7656557	61.15799644	4_EWS-FLI1_loop_and_bridge_target	2.283672359
SCG2	1.964437058	37.08789191	4_EWS-FLI1_loop_and_bridge_target	3.683502369
WDFY1	33.03211041	65.98165537	4_EWS-FLI1_loop_and_bridge_target	0.976869412
RHBDD1	6.68556558	4.62370343	4_EWS-FLI1_loop_and_bridge_target	-0.450630917
LOC654841	0.512936163	0.747462848	2_EWS-FLI1_loop_target	0.207910668
MFF	49.38139863	38.12461053	2_EWS-FLI1_loop_target	-0.364814782
TRIP12	48.49226564	53.02743523	4_EWS-FLI1_loop_and_bridge_target	0.12648911
FBXO36	6.928900565	5.55958073	4_EWS-FLI1_loop_and_bridge_target	-0.273517229
LOC151475	1.444678357	0.070737631	1_EWS-FLI1_in_promoter	-1.191039653
ITM2C	142.3982242	203.039645	4_EWS-FLI1_loop_and_bridge_target	0.508822337
PSMD1	133.3838573	87.64626309	3_EWS-FLI1_bridge_target	-0.600228127
HTR2B	0.713886558	0.451408418	2_EWS-FLI1_loop_target	-0.239818078
LINC00471	1.127326285	0.873346725	3_EWS-FLI1_bridge_target	-0.183423385
ATG16L1	40.92999734	33.20354578	4_EWS-FLI1_loop_and_bridge_target	-0.293836846
DGKD	28.53322781	20.51246428	4_EWS-FLI1_loop_and_bridge_target	-0.457166246
USP40	14.61853627	16.00107369	4_EWS-FLI1_loop_and_bridge_target	0.122366607
UGT1A6	0.017584575	0.028069122	4_EWS-FLI1_loop_and_bridge_target	0.01478856
HJURP	34.00397296	19.46435335	2_EWS-FLI1_loop_target	-0.774405597
LRRFIP1	9.375323991	17.11713305	4_EWS-FLI1_loop_and_bridge_target	0.804198285
RAMP1	50.39023494	3.97910066	4_EWS-FLI1_loop_and_bridge_target	-3.367537161
ESPNL	0.896751355	0.88794357	4_EWS-FLI1_loop_and_bridge_target	-0.006714925
ERFE	18.21132139	2.959316007	4_EWS-FLI1_loop_and_bridge_target	-2.27863363
PDCD6IP	14.61672196	16.65044616	2_EWS-FLI1_loop_target	0.176612996
CCK	179.8393877	3.41892513	1_EWS-FLI1_in_promoter	-5.35486964
SEC22C	14.11560423	17.48043592	2_EWS-FLI1_loop_target	0.289960137
NKTR	12.36621665	12.13513514	2_EWS-FLI1_loop_target	-0.02516012
HIGD1A	25.5382718	19.25085447	2_EWS-FLI1_loop_target	-0.390091642
KIAA1143	8.484481526	7.816452818	4_EWS-FLI1_loop_and_bridge_target	-0.105370587
KIF15	20.79317884	5.537524624	4_EWS-FLI1_loop_and_bridge_target	-1.737060269
LARS2-AS1	0.218265935	0.079635158	4_EWS-FLI1_loop_and_bridge_target	-0.17428523
ELP6	38.47381933	13.60787158	4_EWS-FLI1_loop_and_bridge_target	-1.434150127
SMARCC1	77.46866862	24.1370885	4_EWS-FLI1_loop_and_bridge_target	-1.642299163
MAP4	16.81604236	22.45264565	4_EWS-FLI1_loop_and_bridge_target	0.396573786
ARF4	61.17817823	85.95571791	2_EWS-FLI1_loop_target	0.48387255
ARF4-AS1	1.014568926	0.854731282	2_EWS-FLI1_loop_target	-0.119260985
ABHD6	70.85236829	11.87374595	1_EWS-FLI1_in_promoter	-2.480603805
RPP14	11.59399251	10.13220157	4_EWS-FLI1_loop_and_bridge_target	-0.177996777
PTPRG	5.015703907	16.66024281	2_EWS-FLI1_loop_target	1.553699714
FOXP1	6.939240371	7.245911883	4_EWS-FLI1_loop_and_bridge_target	0.054678068
ROBO2	2.078162045	18.0382369	4_EWS-FLI1_loop_and_bridge_target	2.628758792
VGLL3	3.210517766	28.21822776	4_EWS-FLI1_loop_and_bridge_target	2.794799117
CHMP2B	29.84613671	33.02504539	2_EWS-FLI1_loop_target	0.141507275
CGGBP1	55.42664416	37.59983756	4_EWS-FLI1_loop_and_bridge_target	-0.547781775
C3orf38	30.81696854	34.38467465	4_EWS-FLI1_loop_and_bridge_target	0.153328269

CLDND1	25.60255737	40.43935656	2_EWS-FLI1_loop_target	0.63943666
DCBLD2	22.243247	30.53594638	3_EWS-FLI1_bridge_target	0.440185607
COL8A1	0.209467509	76.87481156	4_EWS-FLI1_loop_and_bridge_target	6.008712849
HP09053	0.234903961	0.53043408	4_EWS-FLI1_loop_and_bridge_target	0.309542058
CMSS1	89.42416564	58.43041192	4_EWS-FLI1_loop_and_bridge_target	-0.605507
TBC1D23	23.86753164	34.35898435	4_EWS-FLI1_loop_and_bridge_target	0.507813523
TOMM70	53.65800151	55.05882288	4_EWS-FLI1_loop_and_bridge_target	0.03650874
LNP1	2.305070548	3.296507595	4_EWS-FLI1_loop_and_bridge_target	0.378483378
TMEM45A	9.38162223	62.46339081	4_EWS-FLI1_loop_and_bridge_target	2.611892709
ADGRG7	3.735315417	0.288747228	1_EWS-FLI1_in_promoter	-1.877491202
TFG	76.67251308	108.5268797	2_EWS-FLI1_loop_target	0.495808925
ABI3BP	1.45783325	19.51857184	4_EWS-FLI1_loop_and_bridge_target	3.061471373
SENP7	11.50374736	12.22514361	4_EWS-FLI1_loop_and_bridge_target	0.080922854
TRMT10C	80.24368378	29.49537334	4_EWS-FLI1_loop_and_bridge_target	-1.413665278
PCNP	88.54710876	83.82244648	4_EWS-FLI1_loop_and_bridge_target	-0.078200758
ZBTB11	31.30442609	25.94842934	4_EWS-FLI1_loop_and_bridge_target	-0.261530655
ZBTB11-AS1	2.848934932	1.732407937	4_EWS-FLI1_loop_and_bridge_target	-0.494286394
RPL24	567.9094317	586.7431282	4_EWS-FLI1_loop_and_bridge_target	0.046986767
CEP97	14.81865639	10.74588621	4_EWS-FLI1_loop_and_bridge_target	-0.429471499
NXPE3	4.439304069	6.702917535	4_EWS-FLI1_loop_and_bridge_target	0.501982902
NFKBIZ	0.338839309	3.570603677	4_EWS-FLI1_loop_and_bridge_target	1.771401911
LOC152225	0.174752613	1.757661697	4_EWS-FLI1_loop_and_bridge_target	1.231088505
LOC101929411	0	0.23610381	4_EWS-FLI1_loop_and_bridge_target	0.305799908
ALCAM	56.80785682	41.58484713	4_EWS-FLI1_loop_and_bridge_target	-0.440925416
NAA50	59.60175534	49.61998812	4_EWS-FLI1_loop_and_bridge_target	-0.259652413
ATP6V1A	39.74782143	37.51362092	4_EWS-FLI1_loop_and_bridge_target	-0.081354162
ZBTB20	0.224605189	0.595365064	4_EWS-FLI1_loop_and_bridge_target	0.381569889
LSAMP	27.58157795	20.01933777	4_EWS-FLI1_loop_and_bridge_target	-0.443368351
CCDC58	62.89278996	38.44472643	4_EWS-FLI1_loop_and_bridge_target	-0.695820706
FAM162A	168.1712313	28.82103745	4_EWS-FLI1_loop_and_bridge_target	-2.504081895
WDR5B	5.884579607	3.264889168	4_EWS-FLI1_loop_and_bridge_target	-0.690860311
LOC102723582	0.446835107	0.792504469	4_EWS-FLI1_loop_and_bridge_target	0.309076206
KPNA1	29.2212285	25.84780394	4_EWS-FLI1_loop_and_bridge_target	-0.170758222
HSPBAP1	18.81011822	9.707452678	4_EWS-FLI1_loop_and_bridge_target	-0.887622189
DIRC2	23.5083036	14.4089282	4_EWS-FLI1_loop_and_bridge_target	-0.669504113
SEMA5B	21.55363787	14.56299866	4_EWS-FLI1_loop_and_bridge_target	-0.535240093
PDIA5	51.03486254	163.8259642	4_EWS-FLI1_loop_and_bridge_target	1.663393085
HACD2	36.05897197	61.73583108	4_EWS-FLI1_loop_and_bridge_target	0.759466801
MYLK-AS1	1.616639374	1.029142742	4_EWS-FLI1_loop_and_bridge_target	-0.366844749
CCDC14	65.31040003	53.95878512	4_EWS-FLI1_loop_and_bridge_target	-0.270885045
UMPS	42.95105231	26.3890946	4_EWS-FLI1_loop_and_bridge_target	-0.682296132
ITGB5	46.39525637	187.3522904	4_EWS-FLI1_loop_and_bridge_target	1.990619
HEG1	1.179420678	33.66164031	3_EWS-FLI1_bridge_target	3.991323328
OSBPL11	14.54331216	19.92509251	4_EWS-FLI1_loop_and_bridge_target	0.428940037
FAM86JP	4.67912544	2.387181884	4_EWS-FLI1_loop_and_bridge_target	-0.74558332
SLC41A3	24.04882563	17.54130545	2_EWS-FLI1_loop_target	-0.434000142
ZXDC	24.2014513	16.66590378	4_EWS-FLI1_loop_and_bridge_target	-0.512539259
MCM2	202.7249521	77.93820892	2_EWS-FLI1_loop_target	-1.367827001
SEC61A1	112.7058604	245.4567793	3_EWS-FLI1_bridge_target	1.116028053
RUVBL1	122.0232203	56.34310523	3_EWS-FLI1_bridge_target	-1.10123871
EEFSEC	5.721278508	4.88502208	3_EWS-FLI1_bridge_target	-0.191687858
RPL32P3	7.563312842	8.072224092	3_EWS-FLI1_bridge_target	0.083287244
SNORA7B	28.94623537	18.7652515	3_EWS-FLI1_bridge_target	-0.599408336
FAM86HP	0.732178177	2.779364858	4_EWS-FLI1_loop_and_bridge_target	1.125556465
PIK3R4	35.01597951	30.09860492	4_EWS-FLI1_loop_and_bridge_target	-0.211787278
ATP2C1	34.08387715	35.11805257	4_EWS-FLI1_loop_and_bridge_target	0.041911917

ASTE1	17.3853391	8.366297179	4_EWS-FLI1_loop_and_bridge_target	-0.973005068
NEK11	8.139573145	5.099248991	4_EWS-FLI1_loop_and_bridge_target	-0.583495175
MRPL3	186.254722	141.2127727	4_EWS-FLI1_loop_and_bridge_target	-0.396951054
DNAJC13	37.65302177	48.961836	2_EWS-FLI1_loop_target	0.37024529
AMOTL2	32.83806169	63.92814916	2_EWS-FLI1_loop_target	0.940197154
COPB2	145.0034694	192.3816169	4_EWS-FLI1_loop_and_bridge_target	0.405448006
LOC100507291	3.588598667	1.990095649	4_EWS-FLI1_loop_and_bridge_target	-0.617861995
SLC25A36	22.34482306	21.65608152	4_EWS-FLI1_loop_and_bridge_target	-0.043204293
RASA2	10.7906587	12.16431263	3_EWS-FLI1_bridge_target	0.158987875
ATP1B3	93.177486	127.0899666	3_EWS-FLI1_bridge_target	0.443703356
TFDP2	16.61254694	17.09356765	3_EWS-FLI1_bridge_target	0.038873353
LOC100289361	1.763044216	0.73071329	3_EWS-FLI1_bridge_target	-0.674891901
U2SURP	81.71311547	47.67841198	3_EWS-FLI1_bridge_target	-0.764834005
ZIC4	0.153181303	0.555175402	3_EWS-FLI1_bridge_target	0.431457953
SERP1	110.9311469	144.338824	2_EWS-FLI1_loop_target	0.376808589
EIF2A	82.28981653	62.92807911	2_EWS-FLI1_loop_target	-0.38169037
LOC101928105	0.444054305	0.630057963	3_EWS-FLI1_bridge_target	0.174798269
GPR87	0.038057467	0.502189056	4_EWS-FLI1_loop_and_bridge_target	0.53318008
P2RY12	0.366430874	0.179577613	2_EWS-FLI1_loop_target	-0.212142131
IGSF10	1.839476274	0.929738428	4_EWS-FLI1_loop_and_bridge_target	-0.55721955
AADACL2-AS1	0.089859003	0.302809593	4_EWS-FLI1_loop_and_bridge_target	0.257484745
AADACP1	1.112234631	0.028635295	4_EWS-FLI1_loop_and_bridge_target	-1.038038538
LOC101928166	0	0.255438724	4_EWS-FLI1_loop_and_bridge_target	0.328191615
MBNL1-AS1	0.524591531	3.316213281	4_EWS-FLI1_loop_and_bridge_target	1.501343388
MBNL1	70.73321204	77.48757927	4_EWS-FLI1_loop_and_bridge_target	0.129823132
P2RY1	10.67419829	5.94484066	3_EWS-FLI1_bridge_target	-0.749309979
SSR3	138.317528	98.38893452	4_EWS-FLI1_loop_and_bridge_target	-0.487219636
TIPARP-AS1	0.441014653	0.159661759	4_EWS-FLI1_loop_and_bridge_target	-0.313380934
TIPARP	42.40879445	89.99033888	4_EWS-FLI1_loop_and_bridge_target	1.067726015
LINC00886	3.142524888	2.13847962	4_EWS-FLI1_loop_and_bridge_target	-0.400444525
PA2G4P4	0.340425492	0.424880054	4_EWS-FLI1_loop_and_bridge_target	0.08814945
LOC105374177	0.37072091	0.410942949	4_EWS-FLI1_loop_and_bridge_target	0.041724797
LINC00881	0.029233523	0.139990755	4_EWS-FLI1_loop_and_bridge_target	0.147451771
CCNL1	38.32386896	47.05592457	4_EWS-FLI1_loop_and_bridge_target	0.289309033
KPNA4	28.05047682	9.917157465	2_EWS-FLI1_loop_target	-1.411964577
SLITRK3	7.584219858	3.578918268	2_EWS-FLI1_loop_target	-0.906680213
BCHE	10.13493512	35.59354724	2_EWS-FLI1_loop_target	1.71649612
PRKCI	41.61700867	36.34912048	2_EWS-FLI1_loop_target	-0.190355063
NAALADL2-AS2	0	0.091476817	4_EWS-FLI1_loop_and_bridge_target	0.126281488
TBL1XR1	52.51784591	70.57906575	4_EWS-FLI1_loop_and_bridge_target	0.419517665
TTC14	20.82540816	18.24486438	3_EWS-FLI1_bridge_target	-0.18153513
FXR1	50.3224403	45.02032728	2_EWS-FLI1_loop_target	-0.157318526
SOX2	59.24992156	6.416304843	4_EWS-FLI1_loop_and_bridge_target	-3.022186912
EIF2B5	77.78521493	51.29909732	3_EWS-FLI1_bridge_target	-0.591138869
AP2M1	293.4416871	457.3775929	3_EWS-FLI1_bridge_target	0.638554579
ALG3	28.23288495	20.0966033	4_EWS-FLI1_loop_and_bridge_target	-0.47058148
ECE2	4.863826256	1.652895067	4_EWS-FLI1_loop_and_bridge_target	-1.144274745
PSMD2	236.7642108	162.6372976	4_EWS-FLI1_loop_and_bridge_target	-0.539029954
EIF4G1	134.8908155	149.4890289	4_EWS-FLI1_loop_and_bridge_target	0.147210362
FAM131A	9.595724176	7.474291881	4_EWS-FLI1_loop_and_bridge_target	-0.322317468
CLCN2	2.326428647	3.571418981	4_EWS-FLI1_loop_and_bridge_target	0.458667963
POLR2H	63.85150604	33.18505006	4_EWS-FLI1_loop_and_bridge_target	-0.92377454
MAGEF1	49.18257831	24.85596976	1_EWS-FLI1_in_promoter	-0.956689181
LOC101928992	0.01784926	0.033240225	4_EWS-FLI1_loop_and_bridge_target	0.021651796
MAP3K13	7.000165246	7.025960095	3_EWS-FLI1_bridge_target	0.004644183
TRA2B	107.7884447	71.31400811	3_EWS-FLI1_bridge_target	-0.589178277

LOC344887	0.66189771	0.397624299	4_EWS-FLI1_loop_and_bridge_target	-0.24985499
AHSG	3.145883908	0.039108811	2_EWS-FLI1_loop_target	-1.996332984
EIF4A2	323.3527568	281.4276772	4_EWS-FLI1_loop_and_bridge_target	-0.199682225
SNORD2	87.20944608	75.05215448	4_EWS-FLI1_loop_and_bridge_target	-0.213944039
SNORA81	14.03012028	11.88890272	4_EWS-FLI1_loop_and_bridge_target	-0.221727108
SNORA63	178.9946964	148.1904107	4_EWS-FLI1_loop_and_bridge_target	-0.27079959
SNORA4	60.42547884	45.44261118	4_EWS-FLI1_loop_and_bridge_target	-0.403388111
RFC4	145.6721211	59.27478426	3_EWS-FLI1_bridge_target	-1.282968187
MASP1	0.490517106	0.237602657	3_EWS-FLI1_bridge_target	-0.268264733
LOC101929130	0.527403575	0.09354003	3_EWS-FLI1_bridge_target	-0.482075274
BCL6	55.43583872	11.39362741	4_EWS-FLI1_loop_and_bridge_target	-2.187013113
LPP-AS2	0.348901033	1.788144904	4_EWS-FLI1_loop_and_bridge_target	1.047521038
LPP	4.167748453	23.99154966	4_EWS-FLI1_loop_and_bridge_target	2.27383261
TPRG1-AS1	1.210926344	0.282436313	4_EWS-FLI1_loop_and_bridge_target	-0.785763781
CLDN16	0.596991771	0.086459986	4_EWS-FLI1_loop_and_bridge_target	-0.555721838
UTS2B	0.364909152	0.378101644	2_EWS-FLI1_loop_target	0.013877372
CCDC50	11.28925227	23.82771753	2_EWS-FLI1_loop_target	1.014554499
LOC647323	0.0787495	0.022855044	4_EWS-FLI1_loop_and_bridge_target	-0.076758186
LOC285389	0.024321553	0.097057312	4_EWS-FLI1_loop_and_bridge_target	0.098970221
HES1	51.00354365	45.58491402	4_EWS-FLI1_loop_and_bridge_target	-0.158747105
LOC100505920	1.334977463	0.190262349	3_EWS-FLI1_bridge_target	-0.972129028
LOC101929337	7.682105067	0.875153732	3_EWS-FLI1_bridge_target	-2.211036004
CPN2	0.00763663	0.056886013	4_EWS-FLI1_loop_and_bridge_target	0.068844313
LINC00884	0.030886532	0.186252664	3_EWS-FLI1_bridge_target	0.202525782
TMEM44-AS1	2.620365393	2.539661811	4_EWS-FLI1_loop_and_bridge_target	-0.032523784
TMEM44	9.735818844	22.77148377	4_EWS-FLI1_loop_and_bridge_target	1.146799723
LSG1	70.48784714	53.40988689	4_EWS-FLI1_loop_and_bridge_target	-0.393829177
XXYLT1	38.57035956	29.25482697	4_EWS-FLI1_loop_and_bridge_target	-0.387254841
APOD	6.34367451	12.24919103	2_EWS-FLI1_loop_target	0.851330253
RUBCN	5.616125508	6.177356278	4_EWS-FLI1_loop_and_bridge_target	0.117465934
FYTTD1	53.03140441	27.52620206	4_EWS-FLI1_loop_and_bridge_target	-0.9215105
LRCH3	23.79556803	22.62246278	3_EWS-FLI1_bridge_target	-0.069922894
IQCG	9.508132619	5.250097344	4_EWS-FLI1_loop_and_bridge_target	-0.749555749
RPL35A	840.0445048	665.3205448	4_EWS-FLI1_loop_and_bridge_target	-0.335965767
LMLN	2.23835251	2.940901666	4_EWS-FLI1_loop_and_bridge_target	0.283265713
FAM86EP	1.384747925	1.419166794	2_EWS-FLI1_loop_target	0.020673465
STX18-IT1	0.021331842	0.181603423	4_EWS-FLI1_loop_and_bridge_target	0.21029422
LOC101928279	0.222902551	0.128914835	2_EWS-FLI1_loop_target	-0.11537279
MSX1	10.84558424	5.735766438	2_EWS-FLI1_loop_target	-0.814435337
BOD1L1	10.16131834	9.672974866	4_EWS-FLI1_loop_and_bridge_target	-0.064545091
CPEB2-AS1	0.361751397	0.150964808	4_EWS-FLI1_loop_and_bridge_target	-0.242619625
CPEB2	8.187481244	4.702070439	4_EWS-FLI1_loop_and_bridge_target	-0.688183538
FBXL5	19.62723819	23.45253212	3_EWS-FLI1_bridge_target	0.245433198
FAM200B	7.266700502	8.860859631	3_EWS-FLI1_bridge_target	0.254401801
LDB2	17.63643862	7.39623197	4_EWS-FLI1_loop_and_bridge_target	-1.15031226
DCAF16	23.0144028	9.622695384	2_EWS-FLI1_loop_target	-1.176750052
NCAPG	39.25241188	14.59243882	2_EWS-FLI1_loop_target	-1.368228632
KLF3-AS1	0.317365201	0.249397944	3_EWS-FLI1_bridge_target	-0.076422285
KLF3	5.271301417	6.056498401	3_EWS-FLI1_bridge_target	0.170187601
RPL9	311.5200608	234.827091	4_EWS-FLI1_loop_and_bridge_target	-0.40621934
LIAS	11.71538663	6.70478816	4_EWS-FLI1_loop_and_bridge_target	-0.722748134
SMIM14	16.02750666	17.96507784	4_EWS-FLI1_loop_and_bridge_target	0.155478097
UBE2K	19.23154052	13.26811647	4_EWS-FLI1_loop_and_bridge_target	-0.50381128
PDS5A	63.29637877	48.90183317	4_EWS-FLI1_loop_and_bridge_target	-0.365644671
N4BP2	16.04108608	8.359206287	4_EWS-FLI1_loop_and_bridge_target	-0.864559195
RHOH	31.23358998	3.452071159	1_EWS-FLI1_in_promoter	-2.856016319

LOC101060498	0.449774811	0.534940517	4_EWS-FLI1_loop_and_bridge_target	0.08235392
CHRNA9	0.253995131	0.248667428	4_EWS-FLI1_loop_and_bridge_target	-0.006142468
RBM47	2.676764088	0.729121478	4_EWS-FLI1_loop_and_bridge_target	-1.088397384
APBB2	7.297723214	3.296500348	4_EWS-FLI1_loop_and_bridge_target	-0.949553523
UCHL1-AS1	0.507784321	0.187048458	4_EWS-FLI1_loop_and_bridge_target	-0.345051245
UCHL1	524.0792907	362.2346853	4_EWS-FLI1_loop_and_bridge_target	-0.531633324
SRD5A3	46.60440343	6.213620512	4_EWS-FLI1_loop_and_bridge_target	-2.722299595
TMEM165	47.3490928	86.72935506	4_EWS-FLI1_loop_and_bridge_target	0.859570843
CEP135	8.185029648	6.167906131	4_EWS-FLI1_loop_and_bridge_target	-0.357732634
AASDH	10.70255049	8.129021512	4_EWS-FLI1_loop_and_bridge_target	-0.35829085
PPAT	58.51199805	34.43820464	4_EWS-FLI1_loop_and_bridge_target	-0.747875039
PAICS	137.5529307	68.80104239	4_EWS-FLI1_loop_and_bridge_target	-0.98911674
SRP72	61.14421391	76.98023137	4_EWS-FLI1_loop_and_bridge_target	0.327488363
NOA1	20.4388076	13.8159308	3_EWS-FLI1_bridge_target	-0.533075402
POLR2B	135.7033087	86.96159233	3_EWS-FLI1_bridge_target	-0.636102535
UTP3	11.69013756	10.54088289	4_EWS-FLI1_loop_and_bridge_target	-0.136954113
RUFY3	5.112766805	8.178407038	4_EWS-FLI1_loop_and_bridge_target	0.586418257
GRSF1	45.03473351	22.77263725	3_EWS-FLI1_bridge_target	-0.953420836
DCK	45.58612186	19.53467235	4_EWS-FLI1_loop_and_bridge_target	-1.181838308
COX18	8.0986524	4.067764563	4_EWS-FLI1_loop_and_bridge_target	-0.844303382
ANKRD17	32.65990359	32.00394853	4_EWS-FLI1_loop_and_bridge_target	-0.028392408
RCHY1	13.35740137	15.19570976	4_EWS-FLI1_loop_and_bridge_target	0.173817043
THAP6	7.11299432	6.719102536	4_EWS-FLI1_loop_and_bridge_target	-0.071801357
G3BP2	165.8469956	96.31762098	4_EWS-FLI1_loop_and_bridge_target	-0.777752751
USO1	69.20041155	57.56634065	4_EWS-FLI1_loop_and_bridge_target	-0.261407734
NAAA	14.02586099	10.99739149	4_EWS-FLI1_loop_and_bridge_target	-0.324726895
CXCL9	0.017078077	0.018173755	3_EWS-FLI1_bridge_target	0.001553349
ART3	7.226161489	0.185124091	4_EWS-FLI1_loop_and_bridge_target	-2.795181266
CXCL11	2.498690194	0.053794768	3_EWS-FLI1_bridge_target	-1.731220999
NUP54	63.37544274	53.37187518	4_EWS-FLI1_loop_and_bridge_target	-0.243649865
RASGEF1B	19.50917225	2.205681475	4_EWS-FLI1_loop_and_bridge_target	-2.677566278
HNRNPD	37.33399375	32.3917504	4_EWS-FLI1_loop_and_bridge_target	-0.199132589
HNRNPDL	117.7702606	114.6223157	4_EWS-FLI1_loop_and_bridge_target	-0.038753767
ENOPH1	80.53195836	35.32169993	4_EWS-FLI1_loop_and_bridge_target	-1.166533943
SEC31A	45.57070014	109.3839951	4_EWS-FLI1_loop_and_bridge_target	1.24503653
LIN54	12.10304392	10.7522107	4_EWS-FLI1_loop_and_bridge_target	-0.156969831
COPS4	74.23268685	66.89571934	3_EWS-FLI1_bridge_target	-0.148038997
PLAC8	0.682764697	0.414324353	3_EWS-FLI1_bridge_target	-0.250720439
HELQ	7.517921999	4.4814872	2_EWS-FLI1_loop_target	-0.63593415
MRPS18C	43.50661151	22.85279491	2_EWS-FLI1_loop_target	-0.899861345
FAM175A	13.15504397	3.172881335	2_EWS-FLI1_loop_target	-1.76220043
NKX6-1	21.32106142	5.104463765	4_EWS-FLI1_loop_and_bridge_target	-1.870469158
WDFY3	8.214515853	9.395516335	4_EWS-FLI1_loop_and_bridge_target	0.173981146
WDFY3-AS2	1.296932489	1.011579677	4_EWS-FLI1_loop_and_bridge_target	-0.191379571
MAPK10	0.768490316	2.354913807	4_EWS-FLI1_loop_and_bridge_target	0.923757383
PTPN13	130.5356323	117.6799283	4_EWS-FLI1_loop_and_bridge_target	-0.14837771
SLC10A6	2.30929262	0.303085472	4_EWS-FLI1_loop_and_bridge_target	-1.344591149
C4orf36	4.049197099	1.246623495	4_EWS-FLI1_loop_and_bridge_target	-1.168295627
LOC100506746	0.558773618	3.327357517	4_EWS-FLI1_loop_and_bridge_target	1.473074898
AFF1	6.420744024	20.44235402	4_EWS-FLI1_loop_and_bridge_target	1.530827551
KLHL8	24.524017	11.67100578	4_EWS-FLI1_loop_and_bridge_target	-1.010324355
HSD17B11	25.29884115	35.90026346	2_EWS-FLI1_loop_target	0.488631887
SMARCAD1	44.57671267	31.96276383	2_EWS-FLI1_loop_target	-0.467459657
PDLIM5	14.20924687	37.36256492	4_EWS-FLI1_loop_and_bridge_target	1.334750467
EIF4E	25.09427953	13.76834996	4_EWS-FLI1_loop_and_bridge_target	-0.821224924
EMCN	4.032473768	19.10918303	4_EWS-FLI1_loop_and_bridge_target	1.998514819

PPP3CA	28.75381761	44.77037991	4_EWS-FLI1_loop_and_bridge_target	0.62133948
FLJ20021	1.370510325	1.473953378	4_EWS-FLI1_loop_and_bridge_target	0.061620636
SLC39A8	20.11083588	12.32610987	4_EWS-FLI1_loop_and_bridge_target	-0.66372801
UBE2D3	67.12204815	81.83667593	3_EWS-FLI1_bridge_target	0.282147851
LOC105377348	2.135253846	3.350776342	3_EWS-FLI1_bridge_target	0.472690599
PPA2	88.76300601	53.59765042	3_EWS-FLI1_bridge_target	-0.717282123
INTS12	23.48007541	20.63246031	4_EWS-FLI1_loop_and_bridge_target	-0.178410246
GSTCD	19.04587188	28.46927634	4_EWS-FLI1_loop_and_bridge_target	0.555906466
TBCK	6.124165361	6.136320183	4_EWS-FLI1_loop_and_bridge_target	0.002459342
AIMP1	26.67739225	31.69883564	4_EWS-FLI1_loop_and_bridge_target	0.240531245
PAPSS1	86.29094305	55.12433655	4_EWS-FLI1_loop_and_bridge_target	-0.637205488
RPL34-AS1	0.079673554	0.035870647	3_EWS-FLI1_bridge_target	-0.059751311
RPL34	241.9323334	205.0161597	3_EWS-FLI1_bridge_target	-0.237797015
OSTC	140.7997493	216.4349827	3_EWS-FLI1_bridge_target	0.616729089
ELOVL6	0.923721587	0.823385742	4_EWS-FLI1_loop_and_bridge_target	-0.077280218
NDST4	47.12296486	31.65579756	4_EWS-FLI1_loop_and_bridge_target	-0.559386386
SNHG8	93.50349444	25.18368594	4_EWS-FLI1_loop_and_bridge_target	-1.851699474
SNORA24	79.25609792	30.89996099	4_EWS-FLI1_loop_and_bridge_target	-1.331056354
LOC101929741	0.059712632	0.039714758	3_EWS-FLI1_bridge_target	-0.027485311
METTL14	20.62590661	14.98627308	3_EWS-FLI1_bridge_target	-0.435926976
EXOSC9	79.11152368	33.77511599	2_EWS-FLI1_loop_target	-1.203954456
CCNA2	79.22641557	39.78232939	3_EWS-FLI1_bridge_target	-0.97613316
FAT4	32.48311143	39.16533998	4_EWS-FLI1_loop_and_bridge_target	0.262517491
PLK4	25.30585474	17.29591906	2_EWS-FLI1_loop_target	-0.523862039
PCDH18	8.887886074	75.34314119	3_EWS-FLI1_bridge_target	2.948764522
SLC7A11	19.1055875	50.17861809	4_EWS-FLI1_loop_and_bridge_target	1.347944699
ELF2	32.74669047	32.17261494	4_EWS-FLI1_loop_and_bridge_target	-0.024753282
NDUFC1	50.26583579	26.53790937	4_EWS-FLI1_loop_and_bridge_target	-0.896578679
NAA15	72.92880531	47.33154461	4_EWS-FLI1_loop_and_bridge_target	-0.613171497
RAB33B	6.868963434	7.424166704	4_EWS-FLI1_loop_and_bridge_target	0.098360382
SETD7	27.54647584	28.9146752	4_EWS-FLI1_loop_and_bridge_target	0.067540748
MAML3	0.541554315	0.78459955	4_EWS-FLI1_loop_and_bridge_target	0.211214659
SCOC	55.3936015	58.90560098	3_EWS-FLI1_bridge_target	0.087159415
SCOC-AS1	0.836901995	0.677096251	3_EWS-FLI1_bridge_target	-0.131309166
TBC1D9	34.75512333	31.40274401	4_EWS-FLI1_loop_and_bridge_target	-0.142033988
ANAPC10	17.0635557	10.23831634	3_EWS-FLI1_bridge_target	-0.68465599
ABCE1	119.2878336	73.83645943	3_EWS-FLI1_bridge_target	-0.684677519
MMAA	4.034880411	1.614812867	4_EWS-FLI1_loop_and_bridge_target	-0.94524981
ZNF827	2.316227112	2.908245935	4_EWS-FLI1_loop_and_bridge_target	0.236978442
LSM6	49.17095299	34.01868231	4_EWS-FLI1_loop_and_bridge_target	-0.518727544
SLC10A7	4.212374099	4.816858119	4_EWS-FLI1_loop_and_bridge_target	0.158299484
TIGD4	0.163699451	0.313563434	2_EWS-FLI1_loop_target	0.174767371
ARFIP1	13.49838017	20.10101097	2_EWS-FLI1_loop_target	0.541420397
TRIM2	45.43261881	34.07146697	2_EWS-FLI1_loop_target	-0.404840875
MND1	31.51808591	7.3310067	4_EWS-FLI1_loop_and_bridge_target	-1.964679596
CTSO	2.010415198	7.013867203	4_EWS-FLI1_loop_and_bridge_target	1.412536125
PDGFC	16.11652987	40.88782716	4_EWS-FLI1_loop_and_bridge_target	1.291140804
C4orf46	21.16990513	9.605004536	4_EWS-FLI1_loop_and_bridge_target	-1.063857359
ETFDH	29.14910732	18.18539227	4_EWS-FLI1_loop_and_bridge_target	-0.652107023
PPID	54.41764534	25.78798307	4_EWS-FLI1_loop_and_bridge_target	-1.048759451
NAF1	3.35908726	1.792617356	4_EWS-FLI1_loop_and_bridge_target	-0.642408175
NPY1R	91.44343369	8.930323749	4_EWS-FLI1_loop_and_bridge_target	-3.218658189
NPY5R	9.172741325	1.260545258	3_EWS-FLI1_bridge_target	-2.169965799
TMA16	62.75834599	27.55166785	4_EWS-FLI1_loop_and_bridge_target	-1.15903918
APELA	19.94001663	0.91033737	4_EWS-FLI1_loop_and_bridge_target	-3.454363239
TMEM192	6.281214276	15.96871822	2_EWS-FLI1_loop_target	1.22062662

LOC101928131	0.141270295	0.02373691	4_EWS-FLI1_loop_and_bridge_target	-0.156795511
LOC101930370	0.43002981	0.92639986	4_EWS-FLI1_loop_and_bridge_target	0.429861972
GALNT7	21.56406046	33.8771234	4_EWS-FLI1_loop_and_bridge_target	0.628254347
HMGB2	264.9854838	137.0816627	4_EWS-FLI1_loop_and_bridge_target	-0.945825771
SAP30	37.33364731	29.53315426	4_EWS-FLI1_loop_and_bridge_target	-0.328234636
HAND2	6.562135838	11.6385707	4_EWS-FLI1_loop_and_bridge_target	0.740967648
HAND2-AS1	0.273114765	0.396014122	4_EWS-FLI1_loop_and_bridge_target	0.132951059
LOC101928509	0.905446741	1.213686579	4_EWS-FLI1_loop_and_bridge_target	0.216321691
FBXO8	10.10478454	12.79808139	4_EWS-FLI1_loop_and_bridge_target	0.313286274
CEP44	11.27951752	7.81370761	4_EWS-FLI1_loop_and_bridge_target	-0.478432934
CDKN2AIP	14.28153744	19.10354883	3_EWS-FLI1_bridge_target	0.395660502
CMBL	16.25573862	8.451716489	2_EWS-FLI1_loop_target	-0.868427968
DAP	17.12063402	35.99153801	4_EWS-FLI1_loop_and_bridge_target	1.029561851
CTNND2	4.404611915	1.679878384	1_EWS-FLI1_in_promoter	-1.012023496
FAM134B	8.207203116	6.033926891	4_EWS-FLI1_loop_and_bridge_target	-0.388432634
MYO10	64.62401106	15.44466406	1_EWS-FLI1_in_promoter	-1.99660424
LOC285696	0.83680519	0.10389074	4_EWS-FLI1_loop_and_bridge_target	-0.734601239
BASP1	100.5463497	45.15301278	4_EWS-FLI1_loop_and_bridge_target	-1.137641647
LOC646241	0.283222133	0.029167045	4_EWS-FLI1_loop_and_bridge_target	-0.318293765
CDH12	4.718925653	0.240121793	4_EWS-FLI1_loop_and_bridge_target	-2.205262335
TARS	84.2927401	104.3787899	2_EWS-FLI1_loop_target	0.305089664
AMACR	1.041671012	0.796506002	2_EWS-FLI1_loop_target	-0.184556659
RAD1	16.2216676	14.85265429	2_EWS-FLI1_loop_target	-0.119500429
BRIX1	105.7369641	61.57614982	2_EWS-FLI1_loop_target	-0.770375082
IL7R	0.45744744	10.1451469	4_EWS-FLI1_loop_and_bridge_target	2.93489987
UGT3A2	106.3109242	31.46840859	1_EWS-FLI1_in_promoter	-1.724688371
LMBRD2	11.13681632	8.046582853	4_EWS-FLI1_loop_and_bridge_target	-0.423945176
SKP2	61.52466078	193.4837408	4_EWS-FLI1_loop_and_bridge_target	1.637152317
NADK2	26.48933573	19.05040386	4_EWS-FLI1_loop_and_bridge_target	-0.455240748
NIPBL-AS1	2.915391737	3.081674577	4_EWS-FLI1_loop_and_bridge_target	0.060004507
NIPBL	12.42546432	14.34061781	4_EWS-FLI1_loop_and_bridge_target	0.192384601
NUP155	72.52283446	38.82888334	2_EWS-FLI1_loop_target	-0.884377354
GDNF-AS1	0.331764221	0.3530492	4_EWS-FLI1_loop_and_bridge_target	0.022875613
EGFLAM	33.45247975	16.36200414	4_EWS-FLI1_loop_and_bridge_target	-0.988674335
LIFR	16.3019647	26.95255858	4_EWS-FLI1_loop_and_bridge_target	0.692044473
LIFR-AS1	0.393362107	0.230494396	4_EWS-FLI1_loop_and_bridge_target	-0.179332147
OSMR-AS1	0.299165285	0.411993762	4_EWS-FLI1_loop_and_bridge_target	0.120148726
LINC01265	8.54132288	0.707458833	1_EWS-FLI1_in_promoter	-2.48233851
OSMR	7.657557731	21.4318315	4_EWS-FLI1_loop_and_bridge_target	1.37351541
RICTOR	7.268106751	9.519859975	4_EWS-FLI1_loop_and_bridge_target	0.347486581
LOC101926940	5.790580158	8.585839944	4_EWS-FLI1_loop_and_bridge_target	0.497370014
PRKAA1	38.10454964	42.29043684	4_EWS-FLI1_loop_and_bridge_target	0.14671189
RPL37	234.4332482	189.7031955	4_EWS-FLI1_loop_and_bridge_target	-0.303989056
SNORD72	8.960440246	6.459406738	4_EWS-FLI1_loop_and_bridge_target	-0.417148615
CARD6	5.395722047	5.039173614	4_EWS-FLI1_loop_and_bridge_target	-0.082756094
C6	2.757125795	0.384585418	4_EWS-FLI1_loop_and_bridge_target	-1.440175361
OXCT1	45.11659409	71.56024681	4_EWS-FLI1_loop_and_bridge_target	0.653893396
OXCT1-AS1	0.14067151	0.242365866	4_EWS-FLI1_loop_and_bridge_target	0.123206712
C5orf51	10.39377128	10.97617179	4_EWS-FLI1_loop_and_bridge_target	0.07192147
ANXA2R	4.666753265	0.75804367	3_EWS-FLI1_bridge_target	-1.68855148
LOC153684	0.552513586	0.479079472	3_EWS-FLI1_bridge_target	-0.069906323
ARL15	7.720307689	2.652754098	3_EWS-FLI1_bridge_target	-1.255394405
SNX18	44.21272716	38.74146306	4_EWS-FLI1_loop_and_bridge_target	-0.186083956
LOC102467080	3.176853835	2.161618676	4_EWS-FLI1_loop_and_bridge_target	-0.401753285
GPX8	49.5987853	102.4891237	4_EWS-FLI1_loop_and_bridge_target	1.032304497
DHX29	36.01158305	22.87985915	4_EWS-FLI1_loop_and_bridge_target	-0.632182516

SKIV2L2	89.60440246	54.60248811	4_EWS-FLI1_loop_and_bridge_target	-0.70443171
IL6ST	12.9351546	59.63901994	3_EWS-FLI1_bridge_target	2.121517428
FLJ31104	0.101109387	0.043038502	3_EWS-FLI1_bridge_target	-0.078165383
PDE4D	8.028838755	13.34139354	4_EWS-FLI1_loop_and_bridge_target	0.667572864
PART1	0.373669915	0.199469345	3_EWS-FLI1_bridge_target	-0.195639087
DEPDC1B	39.27984395	19.0696467	3_EWS-FLI1_bridge_target	-1.005042874
ERCC8	23.91264357	17.62073084	4_EWS-FLI1_loop_and_bridge_target	-0.419968422
NDUFAF2	40.66751022	37.11624138	4_EWS-FLI1_loop_and_bridge_target	-0.128517034
SMIM15	54.02868424	46.69419878	4_EWS-FLI1_loop_and_bridge_target	-0.206370037
CTC-436P18.1	0.352734662	0.229389747	4_EWS-FLI1_loop_and_bridge_target	-0.137936526
ZSWIM6	48.17616077	14.06768159	4_EWS-FLI1_loop_and_bridge_target	-1.706501655
LOC100506526	0.058739331	0	4_EWS-FLI1_loop_and_bridge_target	-0.082347432
DIMT1	62.46522332	36.38145227	3_EWS-FLI1_bridge_target	-0.763643644
PIK3R1	15.119005	10.06324989	4_EWS-FLI1_loop_and_bridge_target	-0.542987444
SLC30A5	113.8525604	69.82498767	4_EWS-FLI1_loop_and_bridge_target	-0.697452669
CENPH	57.25991635	19.70884157	3_EWS-FLI1_bridge_target	-1.492256777
LOC102724392	4.512964781	3.317651478	4_EWS-FLI1_loop_and_bridge_target	-0.352581592
BDP1	49.0233235	38.39955178	3_EWS-FLI1_bridge_target	-0.344421694
LINC01336	1.017616749	2.926144661	4_EWS-FLI1_loop_and_bridge_target	0.960461174
HMGCR	107.4228361	117.4215449	4_EWS-FLI1_loop_and_bridge_target	0.127262929
COL4A3BP	7.923309856	10.0632233	4_EWS-FLI1_loop_and_bridge_target	0.310120937
POLK	14.58300888	15.04686186	4_EWS-FLI1_loop_and_bridge_target	0.042317364
POC5	18.93441333	8.647130841	4_EWS-FLI1_loop_and_bridge_target	-1.047089309
IQGAP2	15.63358481	21.9183364	2_EWS-FLI1_loop_target	0.462403199
F2R	37.39272768	191.2837097	1_EWS-FLI1_in_promoter	2.324331575
ZBED3	0.74960604	0.520238135	2_EWS-FLI1_loop_target	-0.202732777
ZBED3-AS1	3.482731422	2.165525401	2_EWS-FLI1_loop_target	-0.501933092
HOMER1	30.74403252	14.74895019	4_EWS-FLI1_loop_and_bridge_target	-1.011229746
PAPD4	30.248552	31.65487351	4_EWS-FLI1_loop_and_bridge_target	0.06350898
MTX3	18.48527522	10.37140031	4_EWS-FLI1_loop_and_bridge_target	-0.776974386
THBS4	1.726647168	1.935554851	4_EWS-FLI1_loop_and_bridge_target	0.106505188
CTD-2201I18.1	1.58358296	0.285393523	4_EWS-FLI1_loop_and_bridge_target	-1.007163102
EDIL3	5.066814409	56.19606318	4_EWS-FLI1_loop_and_bridge_target	3.236904766
COX7C	705.0176937	401.7876446	2_EWS-FLI1_loop_target	-0.80968491
CCNH	42.48524151	39.02910333	2_EWS-FLI1_loop_target	-0.119476548
CETN3	18.91940462	14.73854644	3_EWS-FLI1_bridge_target	-0.339872222
LOC731157	1.314400742	1.087900082	3_EWS-FLI1_bridge_target	-0.148586019
LUCAT1	0.171880947	0.304847172	4_EWS-FLI1_loop_and_bridge_target	0.155054832
ARRDC3	41.47375588	52.54398908	4_EWS-FLI1_loop_and_bridge_target	0.334152936
ARRDC3-AS1	0.80868314	1.494925696	4_EWS-FLI1_loop_and_bridge_target	0.464057163
NR2F1-AS1	1.876146255	2.100442194	4_EWS-FLI1_loop_and_bridge_target	0.108336952
NR2F1	4.645223295	6.597599705	4_EWS-FLI1_loop_and_bridge_target	0.428513055
ALDH7A1	24.27615484	14.04348906	4_EWS-FLI1_loop_and_bridge_target	-0.748637796
PHAX	28.80144343	34.64007976	4_EWS-FLI1_loop_and_bridge_target	0.258118357
LOC102723557	0.328893127	0.323071297	4_EWS-FLI1_loop_and_bridge_target	-0.006334277
LMNB1	79.72401228	51.64273641	4_EWS-FLI1_loop_and_bridge_target	-0.6167634
PRRC1	33.49690388	24.47893725	4_EWS-FLI1_loop_and_bridge_target	-0.437161782
HINT1	208.4187962	141.829875	4_EWS-FLI1_loop_and_bridge_target	-0.552093163
LYRM7	14.25660239	8.329127458	4_EWS-FLI1_loop_and_bridge_target	-0.709619654
CDC42SE2	41.02309271	11.01471202	4_EWS-FLI1_loop_and_bridge_target	-1.806380272
RAPGEF6	13.45144568	7.163127261	4_EWS-FLI1_loop_and_bridge_target	-0.82401997
GNPDA1	71.02715874	40.49432174	4_EWS-FLI1_loop_and_bridge_target	-0.79562707
NDFIP1	18.18654288	20.61893049	3_EWS-FLI1_bridge_target	0.17220037
FGF1	0.182976987	0.525733825	2_EWS-FLI1_loop_target	0.367081287
LARS	158.0073502	85.53344415	3_EWS-FLI1_bridge_target	-0.877763727
TCERG1	41.14766595	24.26753006	3_EWS-FLI1_bridge_target	-0.738168095

DPYSL3	47.37506903	7.510264549	4_EWS-FLI1_loop_and_bridge_target	-2.506987835
JAKMIP2	44.75075283	8.696159233	4_EWS-FLI1_loop_and_bridge_target	-2.238310187
CSNK1A1	148.1986184	156.003009	2_EWS-FLI1_loop_target	0.073558033
HAND1	1.051078035	0.644206272	4_EWS-FLI1_loop_and_bridge_target	-0.318991079
CNOT8	49.81749273	31.43571738	4_EWS-FLI1_loop_and_bridge_target	-0.647741849
MRPL22	13.01938859	7.634998795	4_EWS-FLI1_loop_and_bridge_target	-0.699155551
MED7	20.06668302	10.26831922	4_EWS-FLI1_loop_and_bridge_target	-0.902690838
CLINT1	77.32135885	63.39415873	4_EWS-FLI1_loop_and_bridge_target	-0.28247597
EBF1	2.997597803	6.282271193	4_EWS-FLI1_loop_and_bridge_target	0.865255138
LOC101927740	0.032306974	0.335201986	4_EWS-FLI1_loop_and_bridge_target	0.371185961
RNF145	21.72674825	28.29378797	4_EWS-FLI1_loop_and_bridge_target	0.366203482
TTC1	101.8098866	67.21336283	3_EWS-FLI1_bridge_target	-0.591852713
CCNG1	67.64717789	58.40236933	4_EWS-FLI1_loop_and_bridge_target	-0.208679936
NUDCD2	56.30917737	22.19326004	4_EWS-FLI1_loop_and_bridge_target	-1.305060568
HMMR	40.49409388	17.46309317	4_EWS-FLI1_loop_and_bridge_target	-1.168261732
MAT2B	86.92310866	36.87175884	4_EWS-FLI1_loop_and_bridge_target	-1.215119971
TENM2	5.658079351	13.99508697	4_EWS-FLI1_loop_and_bridge_target	1.171311919
BOD1	83.01940401	58.28638847	4_EWS-FLI1_loop_and_bridge_target	-0.503021637
CPEB4	7.10846169	6.77323321	3_EWS-FLI1_bridge_target	-0.060913437
MSX2	14.74555852	9.83510429	3_EWS-FLI1_bridge_target	-0.539231893
RNF44	3.324069085	1.256137712	3_EWS-FLI1_bridge_target	-0.938534442
NSD1	16.76774913	12.42988096	3_EWS-FLI1_bridge_target	-0.403814411
MXD3	1.905640056	0.860203305	2_EWS-FLI1_loop_target	-0.643395692
DBN1	87.59607	66.92789864	4_EWS-FLI1_loop_and_bridge_target	-0.383238479
PDLIM7	7.707905588	28.06546508	1_EWS-FLI1_in_promoter	1.738908323
DDX41	25.28761073	21.87362209	4_EWS-FLI1_loop_and_bridge_target	-0.200698184
RREB1	11.45019978	6.077897434	4_EWS-FLI1_loop_and_bridge_target	-0.814776132
SSR1	18.15528724	22.29655712	3_EWS-FLI1_bridge_target	0.282374103
CAGE1	0.317318157	0.147284352	3_EWS-FLI1_bridge_target	-0.19938082
RIOK1	55.30227917	17.69183995	3_EWS-FLI1_bridge_target	-1.590784736
SNRNP48	29.16370029	15.79452576	3_EWS-FLI1_bridge_target	-0.844822363
BLOC1S5	0.024890976	0.004414651	4_EWS-FLI1_loop_and_bridge_target	-0.029115472
EEF1E1	25.7054008	16.52343173	2_EWS-FLI1_loop_target	-0.607846202
SLC35B3	9.836138823	18.23635554	4_EWS-FLI1_loop_and_bridge_target	0.827984715
LOC100506207	0.395070475	0.386097235	4_EWS-FLI1_loop_and_bridge_target	-0.009309538
TFAP2A	1.88437626	2.994886876	4_EWS-FLI1_loop_and_bridge_target	0.469895278
TFAP2A-AS1	0.673850642	0.669906337	4_EWS-FLI1_loop_and_bridge_target	-0.003403616
SIRT5	6.013264314	2.879262675	2_EWS-FLI1_loop_target	-0.854303633
NOL7	58.95959551	36.66746596	4_EWS-FLI1_loop_and_bridge_target	-0.67067167
RANBP9	37.84252331	21.93548248	4_EWS-FLI1_loop_and_bridge_target	-0.760055668
MCUR1	31.28950378	16.16496179	4_EWS-FLI1_loop_and_bridge_target	-0.911598624
RNF182	53.53715935	3.868985111	4_EWS-FLI1_loop_and_bridge_target	-3.485546563
CD83	53.37441803	5.968798807	3_EWS-FLI1_bridge_target	-2.963946146
LINC01108	0.043783072	0.029952039	4_EWS-FLI1_loop_and_bridge_target	-0.019244752
JARID2	31.638177	6.192451426	4_EWS-FLI1_loop_and_bridge_target	-2.182005
JARID2-AS1	0.245996987	0.071394382	4_EWS-FLI1_loop_and_bridge_target	-0.217810943
MYLIP	21.04551884	118.5451526	4_EWS-FLI1_loop_and_bridge_target	2.438998296
LOC100506885	0.548191561	0.675285543	4_EWS-FLI1_loop_and_bridge_target	0.113823025
ID4	48.16247818	71.88863551	4_EWS-FLI1_loop_and_bridge_target	0.568136252
CDKAL1	29.28462143	21.89527398	4_EWS-FLI1_loop_and_bridge_target	-0.403535548
SOX4	55.36087049	41.10543025	4_EWS-FLI1_loop_and_bridge_target	-0.420687587
DCDC2	20.01496812	0.45947173	1_EWS-FLI1_in_promoter	-3.847899097
KAAG1	0.828237323	0.044513864	4_EWS-FLI1_loop_and_bridge_target	-0.807621717
TDP2	22.96155044	14.72600928	3_EWS-FLI1_bridge_target	-0.607568651
ACOT13	15.54652799	8.35459456	3_EWS-FLI1_bridge_target	-0.822781492
C6orf62	80.3015965	105.9147599	4_EWS-FLI1_loop_and_bridge_target	0.395105447

GMNN	88.46483094	43.39364936	4_EWS-FLI1_loop_and_bridge_target	-1.010967354
TRIM38	6.398150446	7.722283077	4_EWS-FLI1_loop_and_bridge_target	0.237541174
HIST1H3A	63.38067782	7.791425733	4_EWS-FLI1_loop_and_bridge_target	-2.87245871
HIST1H4A	53.79993779	12.435929	4_EWS-FLI1_loop_and_bridge_target	-2.028078179
HIST1H4B	519.3768148	145.058786	4_EWS-FLI1_loop_and_bridge_target	-1.83300754
HIST1H3B	527.3383204	226.2356379	4_EWS-FLI1_loop_and_bridge_target	-1.217272938
HIST1H2AB	248.9798937	36.52398898	4_EWS-FLI1_loop_and_bridge_target	-2.735926955
HIST1H2BB	24.00896346	0.428200646	4_EWS-FLI1_loop_and_bridge_target	-4.130174683
HIST1H1C	824.6195388	418.810923	4_EWS-FLI1_loop_and_bridge_target	-0.975737409
HFE	1.056281391	1.351073888	4_EWS-FLI1_loop_and_bridge_target	0.193282177
HIST1H4C	240.7831123	53.12645151	4_EWS-FLI1_loop_and_bridge_target	-2.159307766
HIST1H2BC	484.9909225	276.0451838	1_EWS-FLI1_in_promoter	-0.81080808
HIST1H2AC	279.729211	141.2213572	1_EWS-FLI1_in_promoter	-0.981041062
HIST1H1E	518.2069112	238.2359294	4_EWS-FLI1_loop_and_bridge_target	-1.117875512
HIST1H2BD	308.0207986	136.9261332	4_EWS-FLI1_loop_and_bridge_target	-1.163808108
HIST1H2BE	35.48294469	0.650536858	4_EWS-FLI1_loop_and_bridge_target	-4.466214919
HIST1H4D	605.2491096	142.2125516	4_EWS-FLI1_loop_and_bridge_target	-2.081752783
HIST1H3D	163.44777	49.66873327	4_EWS-FLI1_loop_and_bridge_target	-1.698461778
HIST1H2AD	25.82148372	11.62813837	4_EWS-FLI1_loop_and_bridge_target	-1.086747073
HIST1H2BF	339.2464354	119.5169078	4_EWS-FLI1_loop_and_bridge_target	-1.497344485
HIST1H4E	594.2549864	326.9437247	4_EWS-FLI1_loop_and_bridge_target	-0.860059532
HIST1H2BG	455.5997168	60.49285675	4_EWS-FLI1_loop_and_bridge_target	-2.892439228
HIST1H2AE	304.5852692	39.72502421	4_EWS-FLI1_loop_and_bridge_target	-2.907587538
HIST1H3F	155.1174648	51.77558395	4_EWS-FLI1_loop_and_bridge_target	-1.564689398
HIST1H2BH	188.0910326	60.50491914	4_EWS-FLI1_loop_and_bridge_target	-1.620307241
HIST1H3G	139.7078519	12.51440074	4_EWS-FLI1_loop_and_bridge_target	-3.380133392
HIST1H2BI	0.608518862	0.028154764	4_EWS-FLI1_loop_and_bridge_target	-0.64567541
HIST1H4H	513.5134481	205.3785397	4_EWS-FLI1_loop_and_bridge_target	-1.317915826
BTN3A2	7.445051041	5.327592058	4_EWS-FLI1_loop_and_bridge_target	-0.416449551
BTN2A2	7.393865082	4.114958884	3_EWS-FLI1_bridge_target	-0.714612631
BTN2A3P	2.216449694	2.611362868	4_EWS-FLI1_loop_and_bridge_target	0.167074264
BTN3A3	5.965795681	6.655928773	3_EWS-FLI1_bridge_target	0.136289251
BTN2A1	18.40612723	22.46764683	3_EWS-FLI1_bridge_target	0.274160949
HMGN4	56.22893627	59.68011684	4_EWS-FLI1_loop_and_bridge_target	0.084479072
ABT1	21.43260977	16.74011603	4_EWS-FLI1_loop_and_bridge_target	-0.338582026
ZNF322	4.029633798	4.42649062	4_EWS-FLI1_loop_and_bridge_target	0.109566128
GUSBP2	1.782453167	1.989087154	1_EWS-FLI1_in_promoter	0.103347558
LINC00240	0.17751062	0.163712604	1_EWS-FLI1_in_promoter	-0.017005264
HIST1H2BJ	363.4654066	157.0567562	4_EWS-FLI1_loop_and_bridge_target	-1.205339182
HIST1H2AG	216.6571535	86.42189623	4_EWS-FLI1_loop_and_bridge_target	-1.31599086
HIST1H2BK	63.88350387	64.88882745	4_EWS-FLI1_loop_and_bridge_target	0.022182122
HIST1H2AH	111.7486063	23.48177561	4_EWS-FLI1_loop_and_bridge_target	-2.203329498
LINC01012	0.492302048	0.597076688	3_EWS-FLI1_bridge_target	0.097894017
HIST1H2BL	133.892956	26.80725092	4_EWS-FLI1_loop_and_bridge_target	-2.278281985
HIST1H2AI	138.150649	48.45374858	4_EWS-FLI1_loop_and_bridge_target	-1.492495851
HIST1H3H	321.3121108	68.17720753	4_EWS-FLI1_loop_and_bridge_target	-2.220089716
HIST1H2AJ	9.138872115	0.112105985	4_EWS-FLI1_loop_and_bridge_target	-3.18853098
HIST1H2BM	18.84634763	0.38621266	4_EWS-FLI1_loop_and_bridge_target	-3.839653025
HIST1H4J	10.13286465	3.9053663	4_EWS-FLI1_loop_and_bridge_target	-1.182392091
HIST1H4K	15.97104166	4.970111184	4_EWS-FLI1_loop_and_bridge_target	-1.507245414
HIST1H2AK	92.01731204	26.77377286	4_EWS-FLI1_loop_and_bridge_target	-1.743776081
HIST1H2BN	82.83852654	40.47319441	4_EWS-FLI1_loop_and_bridge_target	-1.015434189
HIST1H2AL	81.76939843	16.02089939	4_EWS-FLI1_loop_and_bridge_target	-2.281790201
HIST1H1B	289.5001527	90.98457906	4_EWS-FLI1_loop_and_bridge_target	-1.659074999
HIST1H3I	0.53325133	0	4_EWS-FLI1_loop_and_bridge_target	-0.616594202
HIST1H3J	369.0603247	81.46651654	4_EWS-FLI1_loop_and_bridge_target	-2.165880095

HIST1H2AM	217.301211	88.37393085	4_EWS-FLI1_loop_and_bridge_target	-1.28839415
HIST1H2BO	340.8396239	140.9253253	4_EWS-FLI1_loop_and_bridge_target	-1.268187586
ZNF165	1.14305289	0.729832602	3_EWS-FLI1_bridge_target	-0.309035022
ZSCAN16-AS1	4.8183225	1.626600921	3_EWS-FLI1_bridge_target	-1.147406247
ZSCAN26	9.372560735	6.556899693	3_EWS-FLI1_bridge_target	-0.456905726
ZSCAN31	0.878442871	1.300473269	3_EWS-FLI1_bridge_target	0.292393453
PPIL1	98.94180191	66.59978617	3_EWS-FLI1_bridge_target	-0.564069546
C6orf89	19.59877942	33.93144176	3_EWS-FLI1_bridge_target	0.761967337
TMEM217	0.091792455	1.923902396	3_EWS-FLI1_bridge_target	1.421196521
TBC1D22B	7.508285644	7.133434023	3_EWS-FLI1_bridge_target	-0.065003867
ZFAND3	11.4994181	23.9243439	4_EWS-FLI1_loop_and_bridge_target	0.995694595
DAAM2	13.05159096	9.35449037	3_EWS-FLI1_bridge_target	-0.440476938
OARD1	21.24269892	14.98653628	4_EWS-FLI1_loop_and_bridge_target	-0.476474463
NFYA	21.8056017	9.345932949	4_EWS-FLI1_loop_and_bridge_target	-1.140324488
LINC01276	0	0.135701822	4_EWS-FLI1_loop_and_bridge_target	0.183584106
FOXP4-AS1	1.844222809	0.467990906	4_EWS-FLI1_loop_and_bridge_target	-0.954191456
FOXP4	0.775454949	1.385683873	4_EWS-FLI1_loop_and_bridge_target	0.42621413
MDFI	98.28715367	59.88653829	1_EWS-FLI1_in_promoter	-0.705483775
TOMM6	123.5792076	71.32936938	4_EWS-FLI1_loop_and_bridge_target	-0.784409823
MED20	28.23753472	7.090152628	4_EWS-FLI1_loop_and_bridge_target	-1.853582844
BYSL	44.6148294	25.02768938	4_EWS-FLI1_loop_and_bridge_target	-0.80945568
CCND3	17.92252644	24.69678718	4_EWS-FLI1_loop_and_bridge_target	0.441483271
RPL7L1	102.0226402	79.30854605	3_EWS-FLI1_bridge_target	-0.359335992
DEFB113	0.464331662	0.148236529	2_EWS-FLI1_loop_target	-0.350822494
TFAP2B	25.25775128	2.375432568	4_EWS-FLI1_loop_and_bridge_target	-2.959599066
MLIP	0.044537205	0.303325285	1_EWS-FLI1_in_promoter	0.319333318
COL21A1	2.553925192	2.139412813	4_EWS-FLI1_loop_and_bridge_target	-0.178918567
LMBRD1	14.60761657	32.03315597	4_EWS-FLI1_loop_and_bridge_target	1.08166457
COL19A1	2.292494556	8.86284543	4_EWS-FLI1_loop_and_bridge_target	1.582822869
FAM135A	7.361489634	12.94218211	3_EWS-FLI1_bridge_target	0.737624484
RIMS1	1.241824507	1.199261171	4_EWS-FLI1_loop_and_bridge_target	-0.027654406
KCNQ5	6.76923385	6.816200943	4_EWS-FLI1_loop_and_bridge_target	0.008695221
EEF1A1	335.6033506	341.989818	2_EWS-FLI1_loop_target	0.027116212
IBTK	28.33133066	28.19056945	4_EWS-FLI1_loop_and_bridge_target	-0.006940168
TPBG	21.94658192	21.51100898	4_EWS-FLI1_loop_and_bridge_target	-0.027648546
UBE3D	39.9990844	6.848187386	4_EWS-FLI1_loop_and_bridge_target	-2.385160298
DOPEY1	10.9925572	4.805195548	4_EWS-FLI1_loop_and_bridge_target	-1.046722751
ZNF292	16.45790475	17.24335736	2_EWS-FLI1_loop_target	0.063490835
AKIRIN2	9.048410908	9.998292989	3_EWS-FLI1_bridge_target	0.130312259
RNGTT	27.53711432	12.30617606	4_EWS-FLI1_loop_and_bridge_target	-1.100743429
PNRC1	17.21540657	27.99898226	4_EWS-FLI1_loop_and_bridge_target	0.670843072
PM20D2	39.74266134	40.44113977	4_EWS-FLI1_loop_and_bridge_target	0.024523461
GABRR2	0.205110054	0.27283661	2_EWS-FLI1_loop_target	0.078882334
UBE2J1	14.58413136	35.41473774	4_EWS-FLI1_loop_and_bridge_target	1.224444712
LYRM2	18.89593468	12.12560505	4_EWS-FLI1_loop_and_bridge_target	-0.600089748
LOC101929057	0.82408274	0.434892407	4_EWS-FLI1_loop_and_bridge_target	-0.346228608
EPHA7	6.593763807	6.875510672	4_EWS-FLI1_loop_and_bridge_target	0.052558349
NDUFAF4	33.76964194	30.01657517	4_EWS-FLI1_loop_and_bridge_target	-0.164788817
KLHL32	0.354923775	0.176871617	4_EWS-FLI1_loop_and_bridge_target	-0.203254743
POU3F2	83.81286476	12.25847542	4_EWS-FLI1_loop_and_bridge_target	-2.677368225
FBXL4	12.69392175	7.872162712	3_EWS-FLI1_bridge_target	-0.626177944
COQ3	35.37106082	11.73951279	3_EWS-FLI1_bridge_target	-1.513480901
PNISR	20.82326453	17.39561607	2_EWS-FLI1_loop_target	-0.246504936
LOC101927365	4.304216586	2.923635299	2_EWS-FLI1_loop_target	-0.434948736
LIN28B	13.3953738	42.38662646	4_EWS-FLI1_loop_and_bridge_target	1.591645162
SEC63	7.760675095	18.94380737	3_EWS-FLI1_bridge_target	1.1868269

FOXO3	9.866098691	10.39945949	2_EWS-FLI1_loop_target	0.069131365
MICAL1	5.366646344	18.19529253	4_EWS-FLI1_loop_and_bridge_target	1.592147014
ZBTB24	17.98825564	8.375843057	4_EWS-FLI1_loop_and_bridge_target	-1.018087053
AK9	0.545024044	0.286737184	4_EWS-FLI1_loop_and_bridge_target	-0.263911877
FIG4	12.9880071	7.786356408	4_EWS-FLI1_loop_and_bridge_target	-0.670853506
WASF1	23.42533046	20.25896156	4_EWS-FLI1_loop_and_bridge_target	-0.200306954
CDC40	24.57345134	10.50535962	4_EWS-FLI1_loop_and_bridge_target	-1.152340799
METTL24	1.218143117	0.413473551	4_EWS-FLI1_loop_and_bridge_target	-0.650107565
CDK19	9.047943144	16.0550184	4_EWS-FLI1_loop_and_bridge_target	0.763296106
AMD1	78.09527229	46.40217165	4_EWS-FLI1_loop_and_bridge_target	-0.738638309
GTF3C6	91.20480707	71.37401611	4_EWS-FLI1_loop_and_bridge_target	-0.349370137
RPF2	42.29848894	37.46076002	4_EWS-FLI1_loop_and_bridge_target	-0.170929406
REV3L	6.714044579	19.25061074	3_EWS-FLI1_bridge_target	1.39240603
TRAF3IP2-AS1	3.905439999	3.164582016	3_EWS-FLI1_bridge_target	-0.236210842
FYN	29.37893271	36.7902718	4_EWS-FLI1_loop_and_bridge_target	0.31494371
MARCKS	28.95031315	119.3686702	3_EWS-FLI1_bridge_target	2.006816941
HSF2	33.55944613	20.2700736	4_EWS-FLI1_loop_and_bridge_target	-0.700255071
SERINC1	123.2915848	235.0331828	4_EWS-FLI1_loop_and_bridge_target	0.925261074
PKIB	1.762127087	2.472989132	4_EWS-FLI1_loop_and_bridge_target	0.330398196
SMPDL3A	3.202828349	10.57450655	4_EWS-FLI1_loop_and_bridge_target	1.461518248
NKAIN2	2.524888693	2.63370743	4_EWS-FLI1_loop_and_bridge_target	0.043864563
RNF217-AS1	0.095230582	0.315536797	4_EWS-FLI1_loop_and_bridge_target	0.264416966
RNF217	2.49681778	9.093943867	4_EWS-FLI1_loop_and_bridge_target	1.529375445
RNF146	13.37088368	22.57217197	3_EWS-FLI1_bridge_target	0.713935919
THEMIS	0.17502368	0.010956039	3_EWS-FLI1_bridge_target	-0.216969568
PTPRK	18.32754321	27.02154014	4_EWS-FLI1_loop_and_bridge_target	0.535877989
ARHGAP18	11.82290063	11.90628681	4_EWS-FLI1_loop_and_bridge_target	0.009351345
TMEM244	0.403073962	0.112877357	2_EWS-FLI1_loop_target	-0.33429645
L3MBTL3	27.49061559	17.09081012	4_EWS-FLI1_loop_and_bridge_target	-0.655229779
EPB41L2	30.56367372	44.67361596	4_EWS-FLI1_loop_and_bridge_target	0.53309588
AKAP7	23.94550733	4.457889159	2_EWS-FLI1_loop_target	-2.192365009
MED23	29.9084951	25.78564598	4_EWS-FLI1_loop_and_bridge_target	-0.206543323
CTGF	6.18803688	128.1195095	4_EWS-FLI1_loop_and_bridge_target	4.166965381
RPS12	809.5829306	747.4639822	2_EWS-FLI1_loop_target	-0.115026903
SNORD101	26.13296758	19.88806249	2_EWS-FLI1_loop_target	-0.377368167
SNORD100	26.92695965	17.48410846	2_EWS-FLI1_loop_target	-0.595373059
SGK1	33.93033648	18.68972883	4_EWS-FLI1_loop_and_bridge_target	-0.827037297
FLJ46906	1.640143746	0.528527643	3_EWS-FLI1_bridge_target	-0.788473838
GVQW2	0.541429209	0.367475731	3_EWS-FLI1_bridge_target	-0.172753405
CCDC28A	15.16334635	9.680063319	3_EWS-FLI1_bridge_target	-0.597805715
REPS1	25.62940939	17.00895216	4_EWS-FLI1_loop_and_bridge_target	-0.56430619
ABRACL	117.5067926	62.50827417	4_EWS-FLI1_loop_and_bridge_target	-0.899953284
HECA	21.72502192	15.52126911	4_EWS-FLI1_loop_and_bridge_target	-0.459957172
CITED2	81.04223738	46.72706676	4_EWS-FLI1_loop_and_bridge_target	-0.781559163
LINC01625	0.718340314	0.3276115	4_EWS-FLI1_loop_and_bridge_target	-0.372182756
EPM2A	3.667094477	2.055931561	2_EWS-FLI1_loop_target	-0.610912438
LOC100507557	0.417395609	0.565582115	2_EWS-FLI1_loop_target	0.143456695
ZBTB2	22.05805842	17.02937952	4_EWS-FLI1_loop_and_bridge_target	-0.354921291
RMND1	33.56575461	20.2091607	3_EWS-FLI1_bridge_target	-0.704655891
ARMT1	60.63868253	47.27657552	3_EWS-FLI1_bridge_target	-0.352512683
SYNE1-AS1	0.258005208	0.394677173	4_EWS-FLI1_loop_and_bridge_target	0.148793325
FBXO5	57.97345623	33.64656492	4_EWS-FLI1_loop_and_bridge_target	-0.767353427
MTRF1L	4.495961687	4.763185236	3_EWS-FLI1_bridge_target	0.068494443
TFB1M	17.68939575	6.520661436	4_EWS-FLI1_loop_and_bridge_target	-1.313288469
ARID1B	7.345222958	7.607111681	4_EWS-FLI1_loop_and_bridge_target	0.044578594
RSPH3	2.373978271	6.601121745	4_EWS-FLI1_loop_and_bridge_target	1.171761659

PSMG3	48.23040286	38.00087012	4_EWS-FLI1_loop_and_bridge_target	-0.336043234
PSMG3-AS1	0.305162874	1.489750215	4_EWS-FLI1_loop_and_bridge_target	0.931771156
CHST12	3.610031731	4.753020474	4_EWS-FLI1_loop_and_bridge_target	0.319542924
BRAT1	12.73991697	6.627545484	3_EWS-FLI1_bridge_target	-0.849082503
TTYH3	5.224655623	6.968315776	4_EWS-FLI1_loop_and_bridge_target	0.356280798
GNA12	13.94753661	19.76017111	4_EWS-FLI1_loop_and_bridge_target	0.47391059
SDK1	8.200764416	3.766748964	4_EWS-FLI1_loop_and_bridge_target	-0.948748078
FOXK1	15.85455086	6.283196873	1_EWS-FLI1_in_promoter	-1.210494435
AP5Z1	2.884703568	4.030615409	4_EWS-FLI1_loop_and_bridge_target	0.372930385
TNRC18	2.836319658	4.028653732	4_EWS-FLI1_loop_and_bridge_target	0.390449278
ETV1	2.830672319	5.130540396	4_EWS-FLI1_loop_and_bridge_target	0.678416629
SOSTDC1	2.24556158	11.50050556	2_EWS-FLI1_loop_target	1.945446409
ANKMY2	6.013976434	7.637401239	4_EWS-FLI1_loop_and_bridge_target	0.300364726
BZW2	81.62437447	60.00073337	4_EWS-FLI1_loop_and_bridge_target	-0.437740857
AHR	27.96352142	27.72305465	4_EWS-FLI1_loop_and_bridge_target	-0.012027832
HDAC9	5.856690194	11.55260074	4_EWS-FLI1_loop_and_bridge_target	0.87240206
TWIST1	105.9582556	36.51948359	4_EWS-FLI1_loop_and_bridge_target	-1.511335965
TWISTNB	51.89468244	64.02820147	4_EWS-FLI1_loop_and_bridge_target	0.297942829
CCDC126	13.51143175	17.09970998	2_EWS-FLI1_loop_target	0.318776712
LOC100506497	0.062344882	0.344992644	4_EWS-FLI1_loop_and_bridge_target	0.34034608
FKBP14	17.63669524	38.82315546	2_EWS-FLI1_loop_target	1.095461484
PLEKHA8	8.512490462	11.17864055	2_EWS-FLI1_loop_target	0.356458093
NEUROD6	2.295250924	0.03416094	1_EWS-FLI1_in_promoter	-1.671927606
CCDC129	0.275137837	0.023681531	1_EWS-FLI1_in_promoter	-0.316886244
FKBP9	9.126804607	48.80572518	3_EWS-FLI1_bridge_target	2.298132569
YAE1D1	9.137708359	7.596726447	3_EWS-FLI1_bridge_target	-0.237872262
RALA	27.36580786	28.59171395	4_EWS-FLI1_loop_and_bridge_target	0.061040307
CDK13	20.86604635	17.75250914	4_EWS-FLI1_loop_and_bridge_target	-0.221608742
MRPS24	0.083059327	0.08838816	2_EWS-FLI1_loop_target	0.007080895
HUS1	14.79541877	6.457906295	4_EWS-FLI1_loop_and_bridge_target	-1.082663611
UPP1	0.510324077	3.836490815	4_EWS-FLI1_loop_and_bridge_target	1.679102511
FKBP9P1	1.921369288	1.678672051	1_EWS-FLI1_in_promoter	-0.125126776
GBAS	82.68690336	44.90457265	4_EWS-FLI1_loop_and_bridge_target	-0.866363994
PSPH	34.18881919	17.36334074	4_EWS-FLI1_loop_and_bridge_target	-0.938288559
CCT6A	251.1188768	187.2840002	4_EWS-FLI1_loop_and_bridge_target	-0.421193732
SNORA15	2.781800513	1.202610635	4_EWS-FLI1_loop_and_bridge_target	-0.779858778
SUMF2	53.42434172	30.01630933	4_EWS-FLI1_loop_and_bridge_target	-0.811225024
BAZ1B	51.36755004	64.13984908	3_EWS-FLI1_bridge_target	0.314867266
BCL7B	23.99715498	20.93702781	3_EWS-FLI1_bridge_target	-0.188395833
TBL2	22.42726512	22.98324915	4_EWS-FLI1_loop_and_bridge_target	0.033838577
STX1A	6.523608565	10.87930556	4_EWS-FLI1_loop_and_bridge_target	0.658953805
EIF4H	124.1878009	113.7385232	4_EWS-FLI1_loop_and_bridge_target	-0.12574413
RFC2	89.56995489	54.1999645	4_EWS-FLI1_loop_and_bridge_target	-0.7143652
GTF2IRD1	13.84867537	10.30939061	4_EWS-FLI1_loop_and_bridge_target	-0.392813042
GTF2I	21.80066892	23.17831933	4_EWS-FLI1_loop_and_bridge_target	0.084637814
GNAI1	10.01010181	48.43286115	4_EWS-FLI1_loop_and_bridge_target	2.166642602
SEMA3C	34.09563881	81.02704924	4_EWS-FLI1_loop_and_bridge_target	1.224807967
CACNA2D1	8.83017821	17.28046975	4_EWS-FLI1_loop_and_bridge_target	0.895013667
STEAP2-AS1	3.964592587	0.29434526	1_EWS-FLI1_in_promoter	-1.939452829
STEAP1	131.1077757	17.67300056	4_EWS-FLI1_loop_and_bridge_target	-2.822689707
STEAP2	26.06319975	3.626424366	1_EWS-FLI1_in_promoter	-2.548362912
GTPBP10	13.94873368	7.636343256	4_EWS-FLI1_loop_and_bridge_target	-0.791530787
CLDN12	21.32822074	29.98338676	4_EWS-FLI1_loop_and_bridge_target	0.472626559
CDK14	42.35202527	38.7614875	4_EWS-FLI1_loop_and_bridge_target	-0.124727663
FZD1	33.77923176	14.52111404	4_EWS-FLI1_loop_and_bridge_target	-1.163993953
AKAP9	15.96562487	18.4223762	4_EWS-FLI1_loop_and_bridge_target	0.195105148

CYP51A1	79.3225065	140.5338196	4_EWS-FLI1_loop_and_bridge_target	0.817270632
CYP51A1-AS1	0.134978333	0.242132849	4_EWS-FLI1_loop_and_bridge_target	0.130154724
GATAD1	8.254517992	14.25476254	4_EWS-FLI1_loop_and_bridge_target	0.721029966
PEX1	18.05869617	9.111428546	4_EWS-FLI1_loop_and_bridge_target	-0.91446259
RBM48	5.824079654	4.358356271	4_EWS-FLI1_loop_and_bridge_target	-0.348843979
CUX1	1.707538205	4.367485418	2_EWS-FLI1_loop_target	0.987264671
FBXL13	1.027042829	0.750265445	2_EWS-FLI1_loop_target	-0.211802834
LRRC17	10.0542328	18.84569518	2_EWS-FLI1_loop_target	0.844227199
ARMC10	19.06784845	8.024920111	2_EWS-FLI1_loop_target	-1.152899882
NAPEPLD	7.670537409	4.324509807	4_EWS-FLI1_loop_and_bridge_target	-0.703472704
PMPCB	31.28693752	23.58065299	4_EWS-FLI1_loop_and_bridge_target	-0.393427363
DNAJC2	56.62279017	37.2501911	4_EWS-FLI1_loop_and_bridge_target	-0.591172564
PSMC2	85.90712906	56.19425141	4_EWS-FLI1_loop_and_bridge_target	-0.603604378
KMT2E-AS1	1.808146186	2.324019355	4_EWS-FLI1_loop_and_bridge_target	0.243310742
KMT2E	5.847473473	6.604484898	4_EWS-FLI1_loop_and_bridge_target	0.151278756
SRPK2	25.17759732	15.8676487	4_EWS-FLI1_loop_and_bridge_target	-0.634073807
PUS7	44.63034461	27.97230012	4_EWS-FLI1_loop_and_bridge_target	-0.655319324
ATXN7L1	1.36441522	1.26848088	4_EWS-FLI1_loop_and_bridge_target	-0.059756912
SYPL1	21.31146922	45.17311584	4_EWS-FLI1_loop_and_bridge_target	1.049267573
LAMB1	65.94966295	148.4117703	4_EWS-FLI1_loop_and_bridge_target	1.158145107
THAP5	21.90009438	14.20324251	4_EWS-FLI1_loop_and_bridge_target	-0.590974494
DNAJB9	11.3158614	7.392844216	4_EWS-FLI1_loop_and_bridge_target	-0.553285829
FOXP2	8.283902488	16.52360903	4_EWS-FLI1_loop_and_bridge_target	0.916496657
MDFIC	17.48838445	18.28490464	4_EWS-FLI1_loop_and_bridge_target	0.060852847
LINC01393	0.155846448	0.176210398	3_EWS-FLI1_bridge_target	0.025196397
TFEC	2.55820296	0.479245289	4_EWS-FLI1_loop_and_bridge_target	-1.266287504
TES	10.9267248	89.64259156	4_EWS-FLI1_loop_and_bridge_target	2.925991191
CAV2	69.82579082	122.779528	4_EWS-FLI1_loop_and_bridge_target	0.805426015
CAV1	473.4388204	404.351563	1_EWS-FLI1_in_promoter	-0.227048356
CAPZA2	112.5956191	141.8625467	4_EWS-FLI1_loop_and_bridge_target	0.330720547
ST7-AS1	0.643005895	0.821111026	4_EWS-FLI1_loop_and_bridge_target	0.148481224
ST7	8.707078535	9.600649759	4_EWS-FLI1_loop_and_bridge_target	0.127043627
ST7-OT4	0.63251897	0.837460949	4_EWS-FLI1_loop_and_bridge_target	0.170613833
LSM8	13.29059631	13.97720098	4_EWS-FLI1_loop_and_bridge_target	0.067701912
FEZF1	36.42777298	0.679789707	1_EWS-FLI1_in_promoter	-4.47775667
FEZF1-AS1	84.33663151	2.110121563	1_EWS-FLI1_in_promoter	-4.778122288
CADPS2	20.38797983	11.82684089	4_EWS-FLI1_loop_and_bridge_target	-0.737634327
IQUB	0.825210945	0.669671363	4_EWS-FLI1_loop_and_bridge_target	-0.128499041
NDUFA5	22.57103648	16.97848248	4_EWS-FLI1_loop_and_bridge_target	-0.390743947
WASL	15.05875506	15.06205801	4_EWS-FLI1_loop_and_bridge_target	0.000296702
TMEM229A	4.649188942	0.187878482	1_EWS-FLI1_in_promoter	-2.249656495
GRM8	5.526515365	1.245699692	4_EWS-FLI1_loop_and_bridge_target	-1.539147899
ZNF800	8.272820032	11.43204468	4_EWS-FLI1_loop_and_bridge_target	0.422983534
SND1	118.2387883	144.171349	2_EWS-FLI1_loop_target	0.283903131
RBM28	31.97312777	16.14495803	4_EWS-FLI1_loop_and_bridge_target	-0.943506372
PRRT4	9.833287171	2.006064662	1_EWS-FLI1_in_promoter	-1.849523122
IMPDH1	36.36384124	40.8561553	2_EWS-FLI1_loop_target	0.163797018
HILPDA	59.98737571	22.91682142	2_EWS-FLI1_loop_target	-1.350484972
METTL2B	31.87724107	26.12971134	3_EWS-FLI1_bridge_target	-0.277215542
NRF1	11.95320209	7.92922932	3_EWS-FLI1_bridge_target	-0.536701215
KLHDC10	20.69209883	21.93364137	4_EWS-FLI1_loop_and_bridge_target	0.080295793
TMEM209	40.20410524	22.34564324	4_EWS-FLI1_loop_and_bridge_target	-0.819634743
LOC105375504	0.200881709	0.356282777	4_EWS-FLI1_loop_and_bridge_target	0.175563955
CEP41	3.329228566	2.899027005	3_EWS-FLI1_bridge_target	-0.150995824
LINC-PINT	2.706521706	2.206209487	4_EWS-FLI1_loop_and_bridge_target	-0.209197269
LOC100506860	1.0377592	1.07835422	4_EWS-FLI1_loop_and_bridge_target	0.028457978

MKLN1	19.64705987	18.81421397	4_EWS-FLI1_loop_and_bridge_target	-0.059400621
MKLN1-AS	1.531409395	0.631418058	4_EWS-FLI1_loop_and_bridge_target	-0.633814321
PODXL	8.818235571	3.010113148	4_EWS-FLI1_loop_and_bridge_target	-1.291820838
CNOT4	3.71447232	3.057639772	3_EWS-FLI1_bridge_target	-0.216455511
C7orf73	33.94502377	19.58828823	2_EWS-FLI1_loop_target	-0.763263143
CREB3L2	1.762505804	3.991788352	2_EWS-FLI1_loop_target	0.85357927
KDM7A	11.14236624	1.213204059	1_EWS-FLI1_in_promoter	-2.455841214
JHDM1D-AS1	2.166633966	0.310175594	1_EWS-FLI1_in_promoter	-1.273189935
MKRN1	39.28647331	25.44333817	2_EWS-FLI1_loop_target	-0.607391205
MRPS33	85.46152114	39.57643156	2_EWS-FLI1_loop_target	-1.091416223
WEE2-AS1	0.444686861	1.115050635	2_EWS-FLI1_loop_target	0.549935383
SSBP1	124.2446454	78.18641052	2_EWS-FLI1_loop_target	-0.661424155
CUL1	68.81269344	51.49965205	4_EWS-FLI1_loop_and_bridge_target	-0.411181512
EZH2	80.13708364	28.78547341	4_EWS-FLI1_loop_and_bridge_target	-1.445752559
GHET1	0.779724907	0.324120966	4_EWS-FLI1_loop_and_bridge_target	-0.426619333
PDIA4	124.3095607	94.3736913	4_EWS-FLI1_loop_and_bridge_target	-0.393833231
ZNF786	8.069950725	3.403452638	4_EWS-FLI1_loop_and_bridge_target	-1.042459564
ZNF425	0.477851822	1.823751525	4_EWS-FLI1_loop_and_bridge_target	0.934111521
ZNF398	6.265085754	7.737839288	4_EWS-FLI1_loop_and_bridge_target	0.266296744
ZNF783	7.098020802	3.720088024	3_EWS-FLI1_bridge_target	-0.778755585
LINC01003	0.910638635	0.899843682	2_EWS-FLI1_loop_target	-0.008174223
LOC101928058	6.496866817	1.326639297	4_EWS-FLI1_loop_and_bridge_target	-1.688040208
KBTBD11-OT1	4.692841366	1.260295199	4_EWS-FLI1_loop_and_bridge_target	-1.332637695
KBTBD11	30.5321191	16.14290722	4_EWS-FLI1_loop_and_bridge_target	-0.879210336
MYOM2	33.66575048	1.227662331	4_EWS-FLI1_loop_and_bridge_target	-3.959908523
LOC101927815	4.416371742	0.907564553	4_EWS-FLI1_loop_and_bridge_target	-1.50559488
LOC100287015	1.469239479	1.716785844	4_EWS-FLI1_loop_and_bridge_target	0.137834084
MCPH1	17.04841985	12.43375255	4_EWS-FLI1_loop_and_bridge_target	-0.426010174
MCPH1-AS1	0.165089459	0.181636385	4_EWS-FLI1_loop_and_bridge_target	0.020345423
AGPAT5	55.3372874	50.52982791	4_EWS-FLI1_loop_and_bridge_target	-0.128682326
PPP1R3B	8.459751986	14.60728168	4_EWS-FLI1_loop_and_bridge_target	0.72234502
TNKS	36.67177209	30.83149925	4_EWS-FLI1_loop_and_bridge_target	-0.243028791
MSRA	17.46929697	12.93578804	4_EWS-FLI1_loop_and_bridge_target	-0.406334365
FAM86B1	0.256087101	0.59549982	4_EWS-FLI1_loop_and_bridge_target	0.345071937
FAM86B2	0.091253815	0.106819219	3_EWS-FLI1_bridge_target	0.020432903
LOC100506990	2.12213085	3.619759873	3_EWS-FLI1_bridge_target	0.565286862
LONRF1	140.7230431	35.33174646	4_EWS-FLI1_loop_and_bridge_target	-1.963771726
LOC340357	0.059906002	0.079686735	4_EWS-FLI1_loop_and_bridge_target	0.026676459
LINC00681	0.458803904	0.097647872	4_EWS-FLI1_loop_and_bridge_target	-0.410370657
KIAA1456	87.90154682	15.12751702	4_EWS-FLI1_loop_and_bridge_target	-2.462684182
DLC1	13.17822805	50.6049875	4_EWS-FLI1_loop_and_bridge_target	1.863833265
LOC102725080	0.012196053	0.077871088	4_EWS-FLI1_loop_and_bridge_target	0.09069589
ASAH1	22.24878562	53.95376345	3_EWS-FLI1_bridge_target	1.241062926
LOC101929066	0.192850696	0.55214869	3_EWS-FLI1_bridge_target	0.379853291
PSD3	24.05429583	36.25452164	3_EWS-FLI1_bridge_target	0.572357548
XPO7	72.5879289	37.90520519	4_EWS-FLI1_loop_and_bridge_target	-0.919505942
FAM160B2	7.240102636	13.50366457	4_EWS-FLI1_loop_and_bridge_target	0.815683253
NUDT18	7.360314059	2.721540693	4_EWS-FLI1_loop_and_bridge_target	-1.167657128
HR	12.32482366	8.549641226	4_EWS-FLI1_loop_and_bridge_target	-0.480598002
REEP4	15.25410686	12.46137075	3_EWS-FLI1_bridge_target	-0.271978959
CCAR2	75.83720626	57.24029026	3_EWS-FLI1_bridge_target	-0.399787519
PPP2R2A	48.65964057	30.94791758	4_EWS-FLI1_loop_and_bridge_target	-0.636351923
BNIP3L	99.08182294	120.7408126	4_EWS-FLI1_loop_and_bridge_target	0.282632928
PNMA2	75.12344921	18.61032226	4_EWS-FLI1_loop_and_bridge_target	-1.956727689
DPYSL2	215.5027081	122.9352961	4_EWS-FLI1_loop_and_bridge_target	-0.804797953
TRIM35	27.18540035	16.98075719	4_EWS-FLI1_loop_and_bridge_target	-0.648494284

PTK2B	4.254186393	3.218660827	4_EWS-FLI1_loop_and_bridge_target	-0.316682279
EPHX2	0.020328542	0.037857329	4_EWS-FLI1_loop_and_bridge_target	0.024574365
CLU	25.98005235	46.26433787	4_EWS-FLI1_loop_and_bridge_target	0.808858897
PBK	107.3263114	42.95398182	4_EWS-FLI1_loop_and_bridge_target	-1.301317931
ELP3	76.51854423	46.96571556	4_EWS-FLI1_loop_and_bridge_target	-0.692537898
DCTN6	107.8661832	94.60465793	3_EWS-FLI1_bridge_target	-0.187403069
GTF2E2	112.690483	82.95339308	4_EWS-FLI1_loop_and_bridge_target	-0.437450951
GSR	93.24858314	109.9500825	4_EWS-FLI1_loop_and_bridge_target	0.235367905
UBXN8	87.8205843	66.73040982	3_EWS-FLI1_bridge_target	-0.391090337
PURG	2.39454028	1.722704225	3_EWS-FLI1_bridge_target	-0.318175938
WRN	33.71571511	22.01420804	3_EWS-FLI1_bridge_target	-0.593064093
NRG1	9.816591303	8.763699594	4_EWS-FLI1_loop_and_bridge_target	-0.147746113
NRG1-IT1	5.982802909	0.312473191	1_EWS-FLI1_in_promoter	-2.411518299
FUT10	18.57003304	16.73321686	4_EWS-FLI1_loop_and_bridge_target	-0.142191923
MAK16	73.52811392	61.80733263	4_EWS-FLI1_loop_and_bridge_target	-0.246851749
TTI2	16.37825324	9.685269397	4_EWS-FLI1_loop_and_bridge_target	-0.701659797
RNF122	17.16985379	8.984543153	4_EWS-FLI1_loop_and_bridge_target	-0.863778488
LINC01605	2.421776492	8.285312053	4_EWS-FLI1_loop_and_bridge_target	1.440204869
ZNF703	4.519102007	2.766388417	4_EWS-FLI1_loop_and_bridge_target	-0.551251762
LOC101929622	0.539154018	0.089249143	4_EWS-FLI1_loop_and_bridge_target	-0.498803626
LOC102723701	2.133900037	0.5544302	1_EWS-FLI1_in_promoter	-1.011573327
ERLIN2	51.64673659	43.10984041	1_EWS-FLI1_in_promoter	-0.255243565
LOC728024	5.947649483	2.698382043	4_EWS-FLI1_loop_and_bridge_target	-0.909630707
PROSC	62.16130322	55.68961912	4_EWS-FLI1_loop_and_bridge_target	-0.155956363
ADGRA2	39.14246653	64.36059608	4_EWS-FLI1_loop_and_bridge_target	0.703291876
BRF2	48.07998808	19.35361295	4_EWS-FLI1_loop_and_bridge_target	-1.26984999
GOT1L1	0.419820566	0.187637088	4_EWS-FLI1_loop_and_bridge_target	-0.257614565
ADRB3	28.93960244	2.459796397	1_EWS-FLI1_in_promoter	-3.113296019
EIF4EBP1	310.541556	117.5218854	4_EWS-FLI1_loop_and_bridge_target	-1.394271133
ASH2L	52.20874312	28.69605025	4_EWS-FLI1_loop_and_bridge_target	-0.841392268
STAR	4.062738363	0.38805518	1_EWS-FLI1_in_promoter	-1.866853008
LSM1	136.0136622	97.97336499	4_EWS-FLI1_loop_and_bridge_target	-0.469207523
BAG4	62.09059834	43.56158197	4_EWS-FLI1_loop_and_bridge_target	-0.501624583
DDHD2	44.42440192	25.08375297	4_EWS-FLI1_loop_and_bridge_target	-0.800316058
PLPP5	40.46430208	33.5174368	4_EWS-FLI1_loop_and_bridge_target	-0.264544473
WHSC1L1	42.76182506	34.37758971	4_EWS-FLI1_loop_and_bridge_target	-0.306837146
LETM2	4.137037187	2.240458238	4_EWS-FLI1_loop_and_bridge_target	-0.664738676
FGFR1	28.3644322	41.89051883	4_EWS-FLI1_loop_and_bridge_target	0.546589026
C8orf86	0.205544149	0.120302178	4_EWS-FLI1_loop_and_bridge_target	-0.105796564
PLEKHA2	76.56246221	39.39343697	4_EWS-FLI1_loop_and_bridge_target	-0.941237696
TM2D2	26.58497071	24.65457243	4_EWS-FLI1_loop_and_bridge_target	-0.104666468
ADAM9	61.01433611	254.8785313	4_EWS-FLI1_loop_and_bridge_target	2.044785435
IDO1	33.75751298	5.428635092	3_EWS-FLI1_bridge_target	-2.434740489
C8orf4	11.19132626	22.84960207	4_EWS-FLI1_loop_and_bridge_target	0.968110114
GINS4	68.05572928	12.47020019	3_EWS-FLI1_bridge_target	-2.357989823
PRKDC	169.7011707	159.8998508	3_EWS-FLI1_bridge_target	-0.085309965
MCM4	153.7809497	76.27500782	3_EWS-FLI1_bridge_target	-1.002154117
SNAI2	4.166435453	65.46768942	4_EWS-FLI1_loop_and_bridge_target	3.685412052
PCMTD1	22.78691251	29.10142152	4_EWS-FLI1_loop_and_bridge_target	0.339663593
RB1CC1	66.49585988	48.25673768	4_EWS-FLI1_loop_and_bridge_target	-0.454477932
ATP6V1H	36.88572444	37.79084888	4_EWS-FLI1_loop_and_bridge_target	0.034062013
TCEA1	59.7612465	58.21177715	4_EWS-FLI1_loop_and_bridge_target	-0.037267312
LYPLA1	103.4268321	49.01608185	4_EWS-FLI1_loop_and_bridge_target	-1.062028505
MRPL15	85.30919521	63.57228206	4_EWS-FLI1_loop_and_bridge_target	-0.418599255
TMEM68	15.23077325	10.65508808	4_EWS-FLI1_loop_and_bridge_target	-0.477771825
TGS1	52.77639598	34.71559829	4_EWS-FLI1_loop_and_bridge_target	-0.590418782

RPS20	430.1431431	357.4386682	4_EWS-FLI1_loop_and_bridge_target	-0.266440652
SNORD54	11.37833682	16.99072974	4_EWS-FLI1_loop_and_bridge_target	0.539436223
PLAG1	19.25715905	28.00090657	4_EWS-FLI1_loop_and_bridge_target	0.517666141
CHCHD7	96.16564719	59.6995142	4_EWS-FLI1_loop_and_bridge_target	-0.67876137
LINC00968	4.179703566	22.11236063	4_EWS-FLI1_loop_and_bridge_target	2.15772318
RAB2A	68.28229742	82.57505721	4_EWS-FLI1_loop_and_bridge_target	0.270585666
LOC401463	1.11723252	0.446410931	4_EWS-FLI1_loop_and_bridge_target	-0.549702233
BHLHE22	58.12922558	3.158306447	4_EWS-FLI1_loop_and_bridge_target	-3.829803395
CYP7B1	11.68850585	7.760666025	4_EWS-FLI1_loop_and_bridge_target	-0.534409734
ARMC1	104.0871314	36.70462174	4_EWS-FLI1_loop_and_bridge_target	-1.478772731
MTFR1	53.94778235	37.75526254	4_EWS-FLI1_loop_and_bridge_target	-0.50366903
VCPIP1	14.44525237	16.14099261	4_EWS-FLI1_loop_and_bridge_target	0.150287212
C8orf44	2.502513644	1.794959558	4_EWS-FLI1_loop_and_bridge_target	-0.325563263
C8orf44-SGK3	0.012669141	0.0296603	4_EWS-FLI1_loop_and_bridge_target	0.024005556
SLCO5A1	13.02330495	3.055202532	4_EWS-FLI1_loop_and_bridge_target	-1.789980523
NCOA2	22.48999257	30.02047085	4_EWS-FLI1_loop_and_bridge_target	0.401174328
TRAM1	128.0400489	371.5420201	4_EWS-FLI1_loop_and_bridge_target	1.52958427
LACTB2-AS1	0.140049429	0.195400883	4_EWS-FLI1_loop_and_bridge_target	0.068398138
LACTB2	4.012776348	1.513196194	4_EWS-FLI1_loop_and_bridge_target	-0.996086567
XKR9	0.394231503	0.593120408	4_EWS-FLI1_loop_and_bridge_target	0.192385179
EYA1	1.760975766	8.938162415	4_EWS-FLI1_loop_and_bridge_target	1.847800894
RPL7	577.8857173	532.0108065	4_EWS-FLI1_loop_and_bridge_target	-0.119113779
RDH10	122.0136364	281.6951136	4_EWS-FLI1_loop_and_bridge_target	1.200428695
STAU2-AS1	0.598219945	0.422416762	4_EWS-FLI1_loop_and_bridge_target	-0.168121734
STAU2	82.26684326	27.65034399	4_EWS-FLI1_loop_and_bridge_target	-1.53918967
UBE2W	20.68361505	17.94776055	4_EWS-FLI1_loop_and_bridge_target	-0.194577955
TCEB1	112.750449	55.91023793	4_EWS-FLI1_loop_and_bridge_target	-0.999112124
TMEM70	163.0580703	31.72451067	4_EWS-FLI1_loop_and_bridge_target	-2.325763039
LY96	127.3308404	25.94765943	4_EWS-FLI1_loop_and_bridge_target	-2.251636043
JPH1	76.58191257	7.765429871	4_EWS-FLI1_loop_and_bridge_target	-3.145823592
PI15	4.495045753	183.4539615	4_EWS-FLI1_loop_and_bridge_target	5.06898547
LINC01111	0.134030085	8.805466869	4_EWS-FLI1_loop_and_bridge_target	3.112127409
ZFHX4-AS1	6.335357488	11.38265827	4_EWS-FLI1_loop_and_bridge_target	0.755381878
ZFHX4	10.96628766	10.82804227	4_EWS-FLI1_loop_and_bridge_target	-0.016764345
PEX2	23.88754797	22.17146437	4_EWS-FLI1_loop_and_bridge_target	-0.103074876
PKIA	22.325588	22.25841521	3_EWS-FLI1_bridge_target	-0.004160652
PKIA-AS1	0.672669036	0.452744286	3_EWS-FLI1_bridge_target	-0.203371233
TPD52	21.2127822	29.39381375	4_EWS-FLI1_loop_and_bridge_target	0.452387608
ZNF704	9.047921706	14.66412291	1_EWS-FLI1_in_promoter	0.640566863
LOC102723322	0.997121257	0.303169574	4_EWS-FLI1_loop_and_bridge_target	-0.615897104
E2F5	46.26612188	36.57944005	4_EWS-FLI1_loop_and_bridge_target	-0.330862928
C8orf59	70.54296026	40.97400381	4_EWS-FLI1_loop_and_bridge_target	-0.769313729
CA13	3.931931605	3.906522808	4_EWS-FLI1_loop_and_bridge_target	-0.007451827
DPY19L4	22.88446753	48.34612874	4_EWS-FLI1_loop_and_bridge_target	1.046864194
INTS8	94.49701879	46.33458675	4_EWS-FLI1_loop_and_bridge_target	-1.012560969
CCNE2	64.50634781	26.01094297	4_EWS-FLI1_loop_and_bridge_target	-1.27809071
C8orf37	4.017343025	3.326686118	4_EWS-FLI1_loop_and_bridge_target	-0.213661112
C8orf37-AS1	0.76376741	0.084079489	4_EWS-FLI1_loop_and_bridge_target	-0.702189779
UQCRB	50.32275064	43.90250572	4_EWS-FLI1_loop_and_bridge_target	-0.192802539
MTERF3	94.98534569	40.62125983	4_EWS-FLI1_loop_and_bridge_target	-1.205493528
PTDSS1	176.546238	96.7722428	4_EWS-FLI1_loop_and_bridge_target	-0.860697939
MTDH	121.8813199	117.973327	4_EWS-FLI1_loop_and_bridge_target	-0.046627451
LAPTM4B	296.2325833	252.2574392	4_EWS-FLI1_loop_and_bridge_target	-0.230987629
RPL30	900.5101576	817.5918847	4_EWS-FLI1_loop_and_bridge_target	-0.139199383
SNORA72	25.92659152	19.29433181	4_EWS-FLI1_loop_and_bridge_target	-0.407954779
RIDA	147.0105732	62.5160801	4_EWS-FLI1_loop_and_bridge_target	-1.220506455

POP1	86.73938316	30.32970333	4_EWS-FLI1_loop_and_bridge_target	-1.485693455
STK3	23.0544426	28.24527595	4_EWS-FLI1_loop_and_bridge_target	0.281900233
OSR2	1.201867687	3.547566483	4_EWS-FLI1_loop_and_bridge_target	1.046366951
ZFPM2	1.202639733	2.301438285	4_EWS-FLI1_loop_and_bridge_target	0.583861132
CSMD3	4.313725639	0.921413307	4_EWS-FLI1_loop_and_bridge_target	-1.467555856
TRPS1	9.088057844	21.18245406	3_EWS-FLI1_bridge_target	1.136770528
LINC00536	0.300642677	0.091408855	4_EWS-FLI1_loop_and_bridge_target	-0.253033014
EIF3H	347.1005908	297.0241485	4_EWS-FLI1_loop_and_bridge_target	-0.224075028
UTP23	35.56579249	28.17401698	3_EWS-FLI1_bridge_target	-0.325810585
RAD21	152.5240358	117.6128779	4_EWS-FLI1_loop_and_bridge_target	-0.372203889
RAD21-AS1	0.385395279	0.263212025	4_EWS-FLI1_loop_and_bridge_target	-0.133200853
MED30	21.43345523	21.35960459	4_EWS-FLI1_loop_and_bridge_target	-0.004757168
EXT1	51.98195026	117.3180385	4_EWS-FLI1_loop_and_bridge_target	1.159097185
TAF2	57.58818864	50.08215416	4_EWS-FLI1_loop_and_bridge_target	-0.197790482
MRPL13	160.6056706	90.90277934	4_EWS-FLI1_loop_and_bridge_target	-0.814297425
MTBP	25.8877762	23.69455508	4_EWS-FLI1_loop_and_bridge_target	-0.122757464
SNTB1	38.50621739	20.01225913	4_EWS-FLI1_loop_and_bridge_target	-0.910848437
LOC101927543	1.025506952	0.199552063	4_EWS-FLI1_loop_and_bridge_target	-0.755787262
DERL1	33.47054153	52.18729128	3_EWS-FLI1_bridge_target	0.625717602
C8orf76	13.55201433	8.196309167	3_EWS-FLI1_bridge_target	-0.662091996
ZHX1-C8orf76	2.369915962	0.499558072	3_EWS-FLI1_bridge_target	-1.168175221
ZHX1	25.61455577	41.85466481	3_EWS-FLI1_bridge_target	0.687236767
ATAD2	133.5058011	61.2528043	4_EWS-FLI1_loop_and_bridge_target	-1.111457663
WDYHV1	41.03008587	16.66341271	3_EWS-FLI1_bridge_target	-1.250658295
FAM91A1	40.75337454	30.43485669	4_EWS-FLI1_loop_and_bridge_target	-0.409527616
TRMT12	28.47999906	15.69697245	4_EWS-FLI1_loop_and_bridge_target	-0.820149947
RNF139-AS1	0.840176275	1.213242519	4_EWS-FLI1_loop_and_bridge_target	0.266317573
RNF139	43.58038649	39.28840905	4_EWS-FLI1_loop_and_bridge_target	-0.146044288
TATDN1	52.09801247	38.72926373	4_EWS-FLI1_loop_and_bridge_target	-0.418455803
NDUFB9	359.4789209	181.0636008	4_EWS-FLI1_loop_and_bridge_target	-0.985472381
LOC105375744	0.255040994	1.296706301	4_EWS-FLI1_loop_and_bridge_target	0.871831891
SQLE	155.4294391	205.9592577	4_EWS-FLI1_loop_and_bridge_target	0.40383474
TRIB1	41.45747674	21.87822571	4_EWS-FLI1_loop_and_bridge_target	-0.892043463
FAM84B	261.4022059	114.0796519	4_EWS-FLI1_loop_and_bridge_target	-1.189147086
CASC11	0.136916744	0.323779786	4_EWS-FLI1_loop_and_bridge_target	0.219536537
MYC	101.0269908	25.06487983	4_EWS-FLI1_loop_and_bridge_target	-1.968771748
PVT1	19.08302605	9.050145719	4_EWS-FLI1_loop_and_bridge_target	-0.998760247
ASAP1	17.54866371	29.91570122	4_EWS-FLI1_loop_and_bridge_target	0.737024473
TMEM71	65.18551961	8.994656984	4_EWS-FLI1_loop_and_bridge_target	-2.727286652
PHF20L1	44.94257738	35.61538776	4_EWS-FLI1_loop_and_bridge_target	-0.32738172
ST3GAL1	9.186792939	3.577601754	4_EWS-FLI1_loop_and_bridge_target	-1.154036065
ZFAT	6.329917728	9.1311653	2_EWS-FLI1_loop_target	0.466931214
TRAPPC9	3.657943831	2.366607685	3_EWS-FLI1_bridge_target	-0.468397631
SPATA6L	4.077206333	0.604342837	4_EWS-FLI1_loop_and_bridge_target	-1.662052423
PLPP6	9.310808412	2.874564548	4_EWS-FLI1_loop_and_bridge_target	-1.412051366
CDC37L1-AS1	1.692857769	1.418175637	4_EWS-FLI1_loop_and_bridge_target	-0.155218997
CDC37L1	15.86754049	12.8146789	4_EWS-FLI1_loop_and_bridge_target	-0.288047596
AK3	49.23985565	34.76488238	4_EWS-FLI1_loop_and_bridge_target	-0.490288622
JAK2	8.644322816	10.21207969	3_EWS-FLI1_bridge_target	0.217302056
PLGRKT	31.42691697	29.67927249	4_EWS-FLI1_loop_and_bridge_target	-0.079927592
RIC1	14.79771919	35.20593102	4_EWS-FLI1_loop_and_bridge_target	1.196509766
MPDZ	16.64871551	24.39463603	4_EWS-FLI1_loop_and_bridge_target	0.52496061
NFIB	7.295596879	10.91228534	4_EWS-FLI1_loop_and_bridge_target	0.522032523
CER1	16.16741193	0.306314487	1_EWS-FLI1_in_promoter	-3.716098399
FREM1	12.13175341	8.420617693	4_EWS-FLI1_loop_and_bridge_target	-0.479166001
LOC389705	0.926952893	0.359911225	4_EWS-FLI1_loop_and_bridge_target	-0.502808828

SNAPC3	24.06883798	9.310211912	4_EWS-FLI1_loop_and_bridge_target	-1.281821139
CCDC171	18.13453218	3.996775119	4_EWS-FLI1_loop_and_bridge_target	-1.937109433
BNC2	2.369761581	4.445092206	4_EWS-FLI1_loop_and_bridge_target	0.692309959
CNTLN	6.092262495	6.232827049	4_EWS-FLI1_loop_and_bridge_target	0.028313721
MLLT3	15.73849078	10.0373124	4_EWS-FLI1_loop_and_bridge_target	-0.600780537
FOCAD-AS1	1.093319942	0.552645404	4_EWS-FLI1_loop_and_bridge_target	-0.431064447
DDX58	10.00358437	4.470285091	3_EWS-FLI1_bridge_target	-1.008285623
TOPORS	31.64064767	25.78956819	4_EWS-FLI1_loop_and_bridge_target	-0.284998357
TOPORS-AS1	3.489043642	1.723598486	4_EWS-FLI1_loop_and_bridge_target	-0.720894086
DNAJA1	235.0008186	154.8198432	3_EWS-FLI1_bridge_target	-0.598912896
SMU1	36.63075877	34.0288857	3_EWS-FLI1_bridge_target	-0.103367284
NFX1	16.14611118	16.8257712	3_EWS-FLI1_bridge_target	0.056083091
EBLN3	25.42023218	17.32070143	4_EWS-FLI1_loop_and_bridge_target	-0.528168405
ZCCHC7	23.80037492	16.24528932	4_EWS-FLI1_loop_and_bridge_target	-0.524159598
ZBTB5	13.93192744	11.14710636	4_EWS-FLI1_loop_and_bridge_target	-0.297787721
FBXO10	7.750867685	10.04729371	3_EWS-FLI1_bridge_target	0.336195013
TOMM5	204.7790365	110.9094242	4_EWS-FLI1_loop_and_bridge_target	-0.878764482
ZFAND5	43.58969012	65.61390085	4_EWS-FLI1_loop_and_bridge_target	0.579113094
ANXA1	306.8183005	615.6782251	4_EWS-FLI1_loop_and_bridge_target	1.0024389
C9orf40	14.64999346	8.138918639	3_EWS-FLI1_bridge_target	-0.77606668
C9orf41-AS1	0.517695151	0.265252429	3_EWS-FLI1_bridge_target	-0.262456792
GNAQ	23.1856911	36.80615087	4_EWS-FLI1_loop_and_bridge_target	0.64446721
CEP78	37.32588317	25.82420481	4_EWS-FLI1_loop_and_bridge_target	-0.514783634
PSAT1	60.64850713	25.63256642	4_EWS-FLI1_loop_and_bridge_target	-1.2108745
UBQLN1	56.05892419	44.29956901	4_EWS-FLI1_loop_and_bridge_target	-0.332955222
LOC105376114	3.409915653	2.172588723	4_EWS-FLI1_loop_and_bridge_target	-0.475090552
HNRNPK	281.5608284	221.1792429	4_EWS-FLI1_loop_and_bridge_target	-0.346837439
RMI1	17.88547354	5.525013859	4_EWS-FLI1_loop_and_bridge_target	-1.533224088
NAA35	10.6613729	14.29002061	2_EWS-FLI1_loop_target	0.390852703
ISCA1	53.0101389	19.34042434	2_EWS-FLI1_loop_target	-1.408880482
ZCCHC6	14.62737928	11.89856513	2_EWS-FLI1_loop_target	-0.276865273
GAS1	81.93874317	46.70420377	4_EWS-FLI1_loop_and_bridge_target	-0.797929781
GAS1RR	0.216060252	0.173843477	4_EWS-FLI1_loop_and_bridge_target	-0.050974663
DAPK1	25.75484565	15.89766491	4_EWS-FLI1_loop_and_bridge_target	-0.662976312
SPIN1	70.49316566	32.53651219	4_EWS-FLI1_loop_and_bridge_target	-1.092072679
NXNL2	0.028372659	0.256640174	4_EWS-FLI1_loop_and_bridge_target	0.289208449
LOC286238	0	0.018040516	4_EWS-FLI1_loop_and_bridge_target	0.025794979
CKS2	229.6230121	90.91904599	4_EWS-FLI1_loop_and_bridge_target	-1.327100746
SECISBP2	28.80997839	12.94771462	4_EWS-FLI1_loop_and_bridge_target	-1.095766578
PALM2	2.185842027	2.077637775	4_EWS-FLI1_loop_and_bridge_target	-0.049851289
PALM2-AKAP2	0.112265983	0.175111547	4_EWS-FLI1_loop_and_bridge_target	0.079295881
AKAP2	0.027691123	0.067602373	2_EWS-FLI1_loop_target	0.054967696
TXN	269.0130091	248.1704919	4_EWS-FLI1_loop_and_bridge_target	-0.11589569
ZFP37	7.781040578	6.208101617	2_EWS-FLI1_loop_target	-0.284772563
CDC26	10.40567254	8.018573882	4_EWS-FLI1_loop_and_bridge_target	-0.338780297
PRPF4	47.58584643	52.27486391	4_EWS-FLI1_loop_and_bridge_target	0.1329189
POLE3	101.8780655	53.90115156	4_EWS-FLI1_loop_and_bridge_target	-0.906027105
C9orf43	0.918513884	0.530138537	4_EWS-FLI1_loop_and_bridge_target	-0.326326927
RGS3	1.773757116	3.339003972	4_EWS-FLI1_loop_and_bridge_target	0.645522443
TNC	4.01304038	129.4570979	4_EWS-FLI1_loop_and_bridge_target	4.701745775
PAPPA	34.16492264	23.32306518	4_EWS-FLI1_loop_and_bridge_target	-0.531811996
MEGF9	11.0033145	7.217734452	3_EWS-FLI1_bridge_target	-0.546620218
PSMD5	12.67849662	16.85162573	4_EWS-FLI1_loop_and_bridge_target	0.384145791
PSMD5-AS1	5.561747042	2.854002024	4_EWS-FLI1_loop_and_bridge_target	-0.767722649
C5	3.859495391	0.902423623	4_EWS-FLI1_loop_and_bridge_target	-1.352967978
STOM	17.75620739	90.01097519	4_EWS-FLI1_loop_and_bridge_target	2.278672397

RC3H2	31.34480949	31.33352274	3_EWS-FLI1_bridge_target	-0.000503518
SPTAN1	63.18445207	80.94691212	2_EWS-FLI1_loop_target	0.352465724
WDR34	54.86477022	17.724539	3_EWS-FLI1_bridge_target	-1.577008569
IER5L	1.826005678	16.75745582	4_EWS-FLI1_loop_and_bridge_target	2.651588627
LINC01503	12.25372491	7.166211123	4_EWS-FLI1_loop_and_bridge_target	-0.698659109
LINC00963	8.266264959	6.909543152	4_EWS-FLI1_loop_and_bridge_target	-0.228393567
NTMT1	23.75701293	12.72707244	4_EWS-FLI1_loop_and_bridge_target	-0.85081328
C9orf50	0.085643395	0.030379338	3_EWS-FLI1_bridge_target	-0.075374726
ASB6	10.25108084	10.55737863	4_EWS-FLI1_loop_and_bridge_target	0.038750611
PRRX2	21.83415884	20.4433914	4_EWS-FLI1_loop_and_bridge_target	-0.09066055
TOR1B	27.20274672	35.51160964	4_EWS-FLI1_loop_and_bridge_target	0.372519596
TOR1A	38.76461635	27.37645726	4_EWS-FLI1_loop_and_bridge_target	-0.486790768
GPR107	17.14526152	30.89404941	4_EWS-FLI1_loop_and_bridge_target	0.81369443
ASS1	48.44969227	39.66057095	4_EWS-FLI1_loop_and_bridge_target	-0.282331069
LOC100272217	0.409650926	0.567859933	4_EWS-FLI1_loop_and_bridge_target	0.15345873
FUBP3	31.22470845	19.78246292	4_EWS-FLI1_loop_and_bridge_target	-0.632800668
EXOSC2	67.55865505	29.93616527	4_EWS-FLI1_loop_and_bridge_target	-1.148044431
ABL1	17.84457824	29.79710849	3_EWS-FLI1_bridge_target	0.708645397
RNU6ATAC	17.48152987	12.00834472	4_EWS-FLI1_loop_and_bridge_target	-0.506646792
RXRA	10.07668943	7.764248136	4_EWS-FLI1_loop_and_bridge_target	-0.337824521
COL5A1	7.900875016	109.9604562	4_EWS-FLI1_loop_and_bridge_target	3.639954644
OLFM1	45.01776766	15.25267361	4_EWS-FLI1_loop_and_bridge_target	-1.501513934
C9orf62	0.114414224	0	3_EWS-FLI1_bridge_target	-0.156285577
LINC01546	0.085770463	0.127782527	4_EWS-FLI1_loop_and_bridge_target	0.054769754
NLGN4X	21.44630386	5.425092962	4_EWS-FLI1_loop_and_bridge_target	-1.804688669
LOC105373156	0.264506165	0.071932774	4_EWS-FLI1_loop_and_bridge_target	-0.238359641
SCML1	9.754948951	51.84114557	2_EWS-FLI1_loop_target	2.296661067
NR0B1	35.58280453	5.728757305	1_EWS-FLI1_in_promoter	-2.442753688
TMEM47	28.73352817	23.88798385	4_EWS-FLI1_loop_and_bridge_target	-0.256641299
USP9X	27.77885416	44.89570569	3_EWS-FLI1_bridge_target	0.67335002
DDX3X	80.34444865	81.51840874	2_EWS-FLI1_loop_target	0.020672108
LOC401585	1.325759761	3.018491022	4_EWS-FLI1_loop_and_bridge_target	0.788951777
LINC01186	0.141213537	0.300546733	4_EWS-FLI1_loop_and_bridge_target	0.188549475
NDUFB11	126.5324021	84.51631829	3_EWS-FLI1_bridge_target	-0.576592191
RBM10	35.53764336	25.40648017	3_EWS-FLI1_bridge_target	-0.468491573
UBA1	177.6918891	66.64631318	4_EWS-FLI1_loop_and_bridge_target	-1.401390939
CDK16	46.36935119	43.18781756	4_EWS-FLI1_loop_and_bridge_target	-0.100305234
USP11	51.15683054	43.08141012	4_EWS-FLI1_loop_and_bridge_target	-0.242685828
ZNF41	5.84116812	5.224449453	4_EWS-FLI1_loop_and_bridge_target	-0.136296446
LINC01560	1.661556877	0.964894698	4_EWS-FLI1_loop_and_bridge_target	-0.437818399
ARAF	10.80437006	12.83840144	4_EWS-FLI1_loop_and_bridge_target	0.229356244
UXT	52.92090523	46.87654522	4_EWS-FLI1_loop_and_bridge_target	-0.171525666
UXT-AS1	0.204634662	0.217763396	4_EWS-FLI1_loop_and_bridge_target	0.015638178
ZNF630	1.256378008	0.097312082	1_EWS-FLI1_in_promoter	-1.040034887
SLC38A5	87.48434226	30.37073862	4_EWS-FLI1_loop_and_bridge_target	-1.496002688
FTSJ1	31.33332035	24.21525	4_EWS-FLI1_loop_and_bridge_target	-0.358725138
WDR13	14.4358296	11.69371575	3_EWS-FLI1_bridge_target	-0.282168581
HDAC6	10.02066499	11.30102204	3_EWS-FLI1_bridge_target	0.158566909
SLC35A2	11.35185105	12.47835321	3_EWS-FLI1_bridge_target	0.125916978
EFNB1	58.55230623	32.18157168	4_EWS-FLI1_loop_and_bridge_target	-0.843775157
AWAT2	0.152416212	1.656703728	2_EWS-FLI1_loop_target	1.204975488
FTX	2.483149551	3.613800196	4_EWS-FLI1_loop_and_bridge_target	0.405563111
RLIM	28.94217386	18.3449015	4_EWS-FLI1_loop_and_bridge_target	-0.630225585
ATRX	21.75525438	20.13102265	4_EWS-FLI1_loop_and_bridge_target	-0.106837125
COX7B	360.3699412	203.8733388	4_EWS-FLI1_loop_and_bridge_target	-0.818744256
ATP7A	12.58804279	7.520088287	3_EWS-FLI1_bridge_target	-0.673397381

PGK1	328.9904614	187.4234638	4_EWS-FLI1_loop_and_bridge_target	-0.808445693
ZCCHC5	0.034930086	0.715543546	4_EWS-FLI1_loop_and_bridge_target	0.729132436
LPAR4	4.511247709	5.456695496	4_EWS-FLI1_loop_and_bridge_target	0.228417018
GUCY2F	0.078162915	0.003327104	1_EWS-FLI1_in_promoter	-0.103783162
KLHL13	19.82344637	9.023867595	2_EWS-FLI1_loop_target	-1.054769597
CUL4B	26.65978284	37.44320191	4_EWS-FLI1_loop_and_bridge_target	0.474938673
MCTS1	8.42012473	5.305802305	4_EWS-FLI1_loop_and_bridge_target	-0.579066222
XIAP	16.53974919	7.410976395	4_EWS-FLI1_loop_and_bridge_target	-1.060282927
LOC101928402	1.950620279	0.830306446	4_EWS-FLI1_loop_and_bridge_target	-0.688933052
STAG2	79.59121031	82.13032649	4_EWS-FLI1_loop_and_bridge_target	0.044752378
ZNF280C	22.01555142	5.875344554	2_EWS-FLI1_loop_target	-1.743105088
HTATSF1	65.21950947	42.20856542	2_EWS-FLI1_loop_target	-0.615938991
ARHGEF6	14.75090305	15.69058673	4_EWS-FLI1_loop_and_bridge_target	0.083600126
RBMX	159.3019774	97.30236203	4_EWS-FLI1_loop_and_bridge_target	-0.705494234
SNORD61	8.077462705	7.078801905	4_EWS-FLI1_loop_and_bridge_target	-0.168147742
ATP11C	52.6973555	51.02849875	4_EWS-FLI1_loop_and_bridge_target	-0.04554896
ZNF275	4.910130996	3.005823991	3_EWS-FLI1_bridge_target	-0.561091075
HAUS7	15.01048166	6.448800159	3_EWS-FLI1_bridge_target	-1.103936748
BGN	0.05658332	0.396405758	3_EWS-FLI1_bridge_target	0.402311671
ARHGAP4	0.253729988	0.474339307	3_EWS-FLI1_bridge_target	0.233841914
ZMYND11	21.12484932	21.29711357	2_EWS-FLI1_loop_target	0.011189331
IDI1	52.04682307	40.25049605	2_EWS-FLI1_loop_target	-0.362854884
CACNB2	3.482836178	1.737128266	4_EWS-FLI1_loop_and_bridge_target	-0.711748735
PLXDC2	9.458416643	23.81564951	4_EWS-FLI1_loop_and_bridge_target	1.246585765
SKIDA1	0.907980995	0.818874226	4_EWS-FLI1_loop_and_bridge_target	-0.069001016
MLLT10	7.9112819	6.290170761	4_EWS-FLI1_loop_and_bridge_target	-0.289680373
COMMD3	19.66252349	10.30527129	3_EWS-FLI1_bridge_target	-0.870020847
KIAA1217	1.000967044	0.858547939	4_EWS-FLI1_loop_and_bridge_target	-0.106521505
PDSS1	19.70919772	9.851752493	4_EWS-FLI1_loop_and_bridge_target	-0.932343616
ANKRD26	5.853414658	4.933068514	3_EWS-FLI1_bridge_target	-0.208044535
YME1L1	59.75204165	68.68828413	4_EWS-FLI1_loop_and_bridge_target	0.19798324
MASTL	15.54111617	12.75893206	4_EWS-FLI1_loop_and_bridge_target	-0.265688093
ACBD5	8.562024856	11.63278756	4_EWS-FLI1_loop_and_bridge_target	0.401784959
RAB18	19.57565728	24.28093954	3_EWS-FLI1_bridge_target	0.297111563
WAC-AS1	3.041009626	2.26344716	2_EWS-FLI1_loop_target	-0.308319108
WAC	23.41480081	23.76265392	2_EWS-FLI1_loop_target	0.020409935
SVIL-AS1	7.915735547	3.182922661	2_EWS-FLI1_loop_target	-1.091842501
SVIL	3.765612494	2.984524035	2_EWS-FLI1_loop_target	-0.258254247
EPC1	6.996073311	7.136155894	4_EWS-FLI1_loop_and_bridge_target	0.025055623
LOC102031319	0.737165467	0.536735619	4_EWS-FLI1_loop_and_bridge_target	-0.176866195
LOC101929431	0.035758325	0.048199795	4_EWS-FLI1_loop_and_bridge_target	0.017226316
CCDC7	1.147995784	0.682468235	4_EWS-FLI1_loop_and_bridge_target	-0.352411894
ITGB1	38.57536112	98.71203558	4_EWS-FLI1_loop_and_bridge_target	1.333165138
FZD8	24.45554414	7.391447402	2_EWS-FLI1_loop_target	-1.600988324
ARID5B	3.389451544	26.29687866	4_EWS-FLI1_loop_and_bridge_target	2.636623398
ADO	13.95557289	7.542747472	4_EWS-FLI1_loop_and_bridge_target	-0.807911134
EGR2	1.543043718	0.346307159	4_EWS-FLI1_loop_and_bridge_target	-0.917548666
HERC4	15.50782706	9.603472604	4_EWS-FLI1_loop_and_bridge_target	-0.638613409
MYPN	2.298387726	0.782212602	4_EWS-FLI1_loop_and_bridge_target	-0.88809155
PBLD	0.668432597	1.750271389	4_EWS-FLI1_loop_and_bridge_target	0.721080582
HNRNPH3	9.207778996	7.161114914	4_EWS-FLI1_loop_and_bridge_target	-0.322830838
H2AFY2	34.95618312	11.68252151	4_EWS-FLI1_loop_and_bridge_target	-1.503398275
TYSND1	7.520070679	0.885788441	4_EWS-FLI1_loop_and_bridge_target	-2.175697564
SAR1A	55.60685389	67.59185069	4_EWS-FLI1_loop_and_bridge_target	0.277060438
OBFC1	2.055124341	1.824377448	2_EWS-FLI1_loop_target	-0.113298196
SFR1	10.91155111	1.722025653	3_EWS-FLI1_bridge_target	-2.129608723

ADD3-AS1	0.198719441	0.346039647	2_EWS-FLI1_loop_target	0.167226867
ADD3	17.16096803	35.58379019	2_EWS-FLI1_loop_target	1.010363446
MXI1	9.050508178	4.684042385	3_EWS-FLI1_bridge_target	-0.822279232
CASP7	57.03177282	33.72460107	4_EWS-FLI1_loop_and_bridge_target	-0.740884883
DCLRE1A	6.242306197	3.37314843	4_EWS-FLI1_loop_and_bridge_target	-0.727776858
NHLRC2	2.579350759	3.676460716	4_EWS-FLI1_loop_and_bridge_target	0.38571914
ADRB1	14.86236093	5.304920245	4_EWS-FLI1_loop_and_bridge_target	-1.331057489
ENO4	0.782897217	0.61840244	4_EWS-FLI1_loop_and_bridge_target	-0.139653134
SHTN1	4.144817156	3.851103106	4_EWS-FLI1_loop_and_bridge_target	-0.084806962
VAX1	1.298411878	0.561188462	4_EWS-FLI1_loop_and_bridge_target	-0.557992648
EIF3A	57.67634688	24.26865297	3_EWS-FLI1_bridge_target	-1.215430295
PRDX3	46.29711616	118.1193978	2_EWS-FLI1_loop_target	1.332584239
GRK5	26.07067989	8.753952316	4_EWS-FLI1_loop_and_bridge_target	-1.472672297
TIAL1	5.421607165	5.475506366	4_EWS-FLI1_loop_and_bridge_target	0.012058599
LINC00959	0.125531222	0.23031883	3_EWS-FLI1_bridge_target	0.128426153
BET1L	9.293293476	12.15795288	3_EWS-FLI1_bridge_target	0.354230386
RIC8A	40.74920904	47.23059535	3_EWS-FLI1_bridge_target	0.208199753
SIRT3	3.629515157	5.994946754	3_EWS-FLI1_bridge_target	0.595451966
PSMD13	104.8162178	63.59548271	3_EWS-FLI1_bridge_target	-0.712055574
IFITM2	0.744393622	8.578833737	3_EWS-FLI1_bridge_target	2.457124392
IFITM1	42.60310997	45.74823235	3_EWS-FLI1_bridge_target	0.100480777
ANO9	0.044236676	0.029956669	3_EWS-FLI1_bridge_target	-0.019865091
PTDSS2	11.88341518	13.48115739	3_EWS-FLI1_bridge_target	0.168661834
LOC143666	0.378347628	0.284203239	4_EWS-FLI1_loop_and_bridge_target	-0.102066248
PHRF1	12.09599421	9.426223096	4_EWS-FLI1_loop_and_bridge_target	-0.328908953
DRD4	0.016977766	0.045167518	3_EWS-FLI1_bridge_target	0.039446056
DEAF1	28.23641467	14.00939287	4_EWS-FLI1_loop_and_bridge_target	-0.96190078
TMEM80	4.351848349	7.601081476	4_EWS-FLI1_loop_and_bridge_target	0.684480835
PDDC1	13.65478262	4.957825958	3_EWS-FLI1_bridge_target	-1.298513683
LOC171391	1.535282523	2.211888878	3_EWS-FLI1_bridge_target	0.341275456
SLC25A22	5.099662868	5.27842172	4_EWS-FLI1_loop_and_bridge_target	0.041672431
PANO1	0.414156121	0.727199735	4_EWS-FLI1_loop_and_bridge_target	0.288493526
PIDD1	3.140305285	1.546134592	4_EWS-FLI1_loop_and_bridge_target	-0.701428465
RPLP2	515.4056017	487.9276003	4_EWS-FLI1_loop_and_bridge_target	-0.078883802
SNORA52	39.34483151	19.37362931	4_EWS-FLI1_loop_and_bridge_target	-0.985680863
PNPLA2	3.830133555	2.605713551	3_EWS-FLI1_bridge_target	-0.421778292
POLR2L	45.3367392	29.0455602	4_EWS-FLI1_loop_and_bridge_target	-0.625004702
TSPAN4	20.85053783	36.47296977	4_EWS-FLI1_loop_and_bridge_target	0.778181526
MRPL23	82.98983523	46.3624727	2_EWS-FLI1_loop_target	-0.826470335
SNORD131	3.721968109	0.926985964	2_EWS-FLI1_loop_target	-1.293042235
FAM160A2	6.707996873	4.908063921	2_EWS-FLI1_loop_target	-0.383670551
SMPD1	48.60364163	18.01325568	1_EWS-FLI1_in_promoter	-1.38344045
TRIM3	5.332584506	5.009645649	4_EWS-FLI1_loop_and_bridge_target	-0.075514498
ARFIP2	23.00948103	21.89764957	4_EWS-FLI1_loop_and_bridge_target	-0.068404706
TIMM10B	37.74893875	19.16111003	4_EWS-FLI1_loop_and_bridge_target	-0.942581726
RRP8	22.78696361	20.01184672	4_EWS-FLI1_loop_and_bridge_target	-0.178968159
ILK	56.84668522	118.8938334	4_EWS-FLI1_loop_and_bridge_target	1.051451261
TAF10	63.96523854	29.01970457	4_EWS-FLI1_loop_and_bridge_target	-1.113758191
TPP1	2.726019822	3.485970992	4_EWS-FLI1_loop_and_bridge_target	0.267784942
MRPL17	73.8962056	23.87403803	4_EWS-FLI1_loop_and_bridge_target	-1.590251898
RPL27A	55.48696741	58.56518689	2_EWS-FLI1_loop_target	0.076551339
SNORA3A	29.79401968	17.5090146	2_EWS-FLI1_loop_target	-0.734422111
SNORA3B	14.03309529	19.91122106	2_EWS-FLI1_loop_target	0.476135219
LOC644656	6.837984485	5.328344516	4_EWS-FLI1_loop_and_bridge_target	-0.308654575
ZNF143	19.11414117	12.87977752	4_EWS-FLI1_loop_and_bridge_target	-0.535225696
SBF2	20.42714138	24.90497229	4_EWS-FLI1_loop_and_bridge_target	0.273789649

ADM	5.25737334	199.4426466	4_EWS-FLI1_loop_and_bridge_target	5.001488498
CAND1.11	0.079423954	2.764996762	4_EWS-FLI1_loop_and_bridge_target	1.802387017
RNF141	56.61177328	26.83963184	4_EWS-FLI1_loop_and_bridge_target	-1.049223532
MRVI1-AS1	0.488167354	0.109365617	4_EWS-FLI1_loop_and_bridge_target	-0.423801859
LYVE1	2.557483074	1.781565897	4_EWS-FLI1_loop_and_bridge_target	-0.354959609
CTR9	36.37283199	21.57015585	4_EWS-FLI1_loop_and_bridge_target	-0.72757351
EIF4G2	279.0044325	351.7754847	4_EWS-FLI1_loop_and_bridge_target	0.333300642
LOC101928053	0.277286539	0.511114472	4_EWS-FLI1_loop_and_bridge_target	0.242530746
ZBED5	28.84770138	27.74040315	4_EWS-FLI1_loop_and_bridge_target	-0.054539532
ZBED5-AS1	5.703019094	6.455814033	4_EWS-FLI1_loop_and_bridge_target	0.153554833
COPB1	132.487686	178.0276579	4_EWS-FLI1_loop_and_bridge_target	0.423475825
PSMA1	165.8511207	103.7644171	4_EWS-FLI1_loop_and_bridge_target	-0.671412584
CYP2R1	16.88545819	6.387546836	4_EWS-FLI1_loop_and_bridge_target	-1.275619801
C11orf58	84.174977	53.39096137	4_EWS-FLI1_loop_and_bridge_target	-0.647062727
PIK3C2A	21.71807754	31.29505803	2_EWS-FLI1_loop_target	0.507472657
HPS5	26.56858195	18.89162166	3_EWS-FLI1_bridge_target	-0.470864165
GTF2H1	58.46386085	31.11786271	3_EWS-FLI1_bridge_target	-0.888637245
LDHA	209.3860252	117.7736608	3_EWS-FLI1_bridge_target	-0.824823938
TCP11L1	8.046039033	14.31130329	4_EWS-FLI1_loop_and_bridge_target	0.759238963
CSTF3	61.1749885	36.60146762	4_EWS-FLI1_loop_and_bridge_target	-0.725545363
CSTF3-AS1	0.12908871	0.370303484	4_EWS-FLI1_loop_and_bridge_target	0.279336605
HIPK3	21.00601412	16.40282047	4_EWS-FLI1_loop_and_bridge_target	-0.338576716
C11orf91	0.166449788	0.299756258	4_EWS-FLI1_loop_and_bridge_target	0.156116896
CD59	12.81248514	41.09991685	4_EWS-FLI1_loop_and_bridge_target	1.607844471
LMO2	6.948600319	17.26378811	4_EWS-FLI1_loop_and_bridge_target	1.200213286
CAPRIN1	151.9455013	102.8584343	1_EWS-FLI1_in_promoter	-0.558399292
NAT10	25.68729635	14.05128957	4_EWS-FLI1_loop_and_bridge_target	-0.826266056
ABTB2	13.63228703	2.268505654	4_EWS-FLI1_loop_and_bridge_target	-2.162452183
CAT	22.54059695	18.36450794	4_EWS-FLI1_loop_and_bridge_target	-0.281736063
PRR5L	32.30406279	9.319694582	1_EWS-FLI1_in_promoter	-1.69029791
TRAF6	5.380927233	5.163199715	4_EWS-FLI1_loop_and_bridge_target	-0.05008654
LRRC4C	3.526506255	0.511139893	2_EWS-FLI1_loop_target	-1.582760724
ALX4	31.25678399	14.46678414	4_EWS-FLI1_loop_and_bridge_target	-1.060429347
CD82	6.433890442	12.66742939	4_EWS-FLI1_loop_and_bridge_target	0.878552591
CRY2	9.822815123	12.66699464	4_EWS-FLI1_loop_and_bridge_target	0.336620223
PHF21A	3.174708049	3.065015352	4_EWS-FLI1_loop_and_bridge_target	-0.038414508
PACSIN3	8.895817137	8.194748269	3_EWS-FLI1_bridge_target	-0.106008764
ACP2	18.36618177	24.6763256	3_EWS-FLI1_bridge_target	0.40689922
NR1H3	3.216344189	3.098495632	3_EWS-FLI1_bridge_target	-0.040898182
ZDHHC5	31.0750299	35.77227006	3_EWS-FLI1_bridge_target	0.197167631
LPXN	1.381164604	1.509965089	3_EWS-FLI1_bridge_target	0.075999944
ZFP91	29.23686005	29.70709232	3_EWS-FLI1_bridge_target	0.022263578
GLYAT	0.438939696	0.02981494	2_EWS-FLI1_loop_target	-0.482621027
OSBP	18.1991794	12.13661425	3_EWS-FLI1_bridge_target	-0.547451157
PATL1	8.670693407	5.914806313	4_EWS-FLI1_loop_and_bridge_target	-0.483930496
TMEM258	166.0294116	106.1824387	4_EWS-FLI1_loop_and_bridge_target	-0.640033618
FEN1	109.1270086	45.99112615	4_EWS-FLI1_loop_and_bridge_target	-1.228708085
FADS1	145.8782168	63.88702609	3_EWS-FLI1_bridge_target	-1.178618498
FADS2	154.9955361	145.832534	4_EWS-FLI1_loop_and_bridge_target	-0.087333082
FADS3	9.891740523	44.57579069	4_EWS-FLI1_loop_and_bridge_target	2.065033167
RAB3IL1	3.768046083	1.579328612	4_EWS-FLI1_loop_and_bridge_target	-0.886402592
BEST1	1.878035607	1.174390763	4_EWS-FLI1_loop_and_bridge_target	-0.404473208
FTH1	138.6670403	148.884958	4_EWS-FLI1_loop_and_bridge_target	0.101864003
INCENP	22.77513203	8.656462663	3_EWS-FLI1_bridge_target	-1.299886647
WDR74	51.47679555	33.4339771	3_EWS-FLI1_bridge_target	-0.607846805
CD248	2.372938096	12.70285059	3_EWS-FLI1_bridge_target	2.022398301

LRFN4	10.3727432	5.674855528	3_EWS-FLI1_bridge_target	-0.76877177
KDM2A	21.47609198	11.52508256	4_EWS-FLI1_loop_and_bridge_target	-0.843571096
ANKRD13D	8.210595083	6.597599705	3_EWS-FLI1_bridge_target	-0.277750667
LOC100130987	1.618660173	1.085614578	4_EWS-FLI1_loop_and_bridge_target	-0.32835628
POLD4	3.435711326	4.711731755	3_EWS-FLI1_bridge_target	0.364762748
CPT1A	60.54113666	37.25185317	4_EWS-FLI1_loop_and_bridge_target	-0.686021447
MRGPRF	4.451543372	2.473660549	4_EWS-FLI1_loop_and_bridge_target	-0.650207946
MRGPRF-AS1	0.110604513	0.070620336	4_EWS-FLI1_loop_and_bridge_target	-0.052898201
TPCN2	8.670243582	8.746692542	4_EWS-FLI1_loop_and_bridge_target	0.011360506
CCND1	249.7458456	9.630051813	4_EWS-FLI1_loop_and_bridge_target	-4.560005264
ORAOV1	11.35596854	6.349005134	4_EWS-FLI1_loop_and_bridge_target	-0.749587238
PPFIA1	29.24974558	18.20989671	1_EWS-FLI1_in_promoter	-0.655073247
FCHSD2	22.37953479	6.946482588	3_EWS-FLI1_bridge_target	-1.556857907
RAB6A	29.40479376	33.23363039	4_EWS-FLI1_loop_and_bridge_target	0.171115491
COA4	82.49159252	25.31592884	4_EWS-FLI1_loop_and_bridge_target	-1.665694611
PAAF1	16.5082879	9.682485625	4_EWS-FLI1_loop_and_bridge_target	-0.712790637
UCP2	7.487813509	0.642838604	2_EWS-FLI1_loop_target	-2.369202205
PGM2L1	5.391821658	7.120873286	4_EWS-FLI1_loop_and_bridge_target	0.345407722
KCNE3	101.0852902	7.907187525	1_EWS-FLI1_in_promoter	-3.51866122
LIPT2	9.478315153	2.499175226	4_EWS-FLI1_loop_and_bridge_target	-1.582319943
LOC100287896	4.369578653	2.307552625	4_EWS-FLI1_loop_and_bridge_target	-0.699044775
POLD3	35.81022611	19.85568013	4_EWS-FLI1_loop_and_bridge_target	-0.819666251
PRCP	34.69155336	34.78925075	4_EWS-FLI1_loop_and_bridge_target	0.00394365
DDIAS	41.28910639	15.1179625	4_EWS-FLI1_loop_and_bridge_target	-1.391616693
RAB30	4.639620336	10.24790695	4_EWS-FLI1_loop_and_bridge_target	0.995986617
RAB30-AS1	19.32702924	6.892227666	4_EWS-FLI1_loop_and_bridge_target	-1.364894904
PCF11	24.52983587	34.53840912	4_EWS-FLI1_loop_and_bridge_target	0.477194837
ANKRD42	8.647646953	4.421301828	4_EWS-FLI1_loop_and_bridge_target	-0.831537784
CCDC90B	23.07927824	12.78765448	4_EWS-FLI1_loop_and_bridge_target	-0.804415099
CREBZF	17.88234096	15.90782451	4_EWS-FLI1_loop_and_bridge_target	-0.159346592
EED	23.16937971	20.87049721	4_EWS-FLI1_loop_and_bridge_target	-0.144194429
TMEM135	3.706670677	11.69251536	4_EWS-FLI1_loop_and_bridge_target	1.431199189
DISC1FP1	5.078852519	0.540469618	4_EWS-FLI1_loop_and_bridge_target	-1.980428789
DCUN1D5	10.36558663	9.20160206	4_EWS-FLI1_loop_and_bridge_target	-0.155876419
DYNC2H1	20.65419361	17.0080938	4_EWS-FLI1_loop_and_bridge_target	-0.266000972
ZC3H12C	3.633839446	4.19248317	4_EWS-FLI1_loop_and_bridge_target	0.164216577
RDX	62.35025972	61.99939375	4_EWS-FLI1_loop_and_bridge_target	-0.008012589
LOC105369486	164.8843056	9.074147907	1_EWS-FLI1_in_promoter	-4.041447676
FDX1	35.75197793	17.27188133	4_EWS-FLI1_loop_and_bridge_target	-1.008196709
FDXACB1	14.28074289	2.243225774	3_EWS-FLI1_bridge_target	-2.236213319
C11orf1	5.575097531	2.903807321	3_EWS-FLI1_bridge_target	-0.752130447
DIXDC1	13.29213393	10.05212089	4_EWS-FLI1_loop_and_bridge_target	-0.37089809
PIH1D2	3.011645585	1.386801119	3_EWS-FLI1_bridge_target	-0.749115796
C11orf57	46.16183542	34.18470751	3_EWS-FLI1_bridge_target	-0.422671345
TIMM8B	65.38535174	25.12531142	4_EWS-FLI1_loop_and_bridge_target	-1.345416704
SDHD	95.37251768	66.89466733	4_EWS-FLI1_loop_and_bridge_target	-0.50532353
PTS	61.49819319	32.99849878	4_EWS-FLI1_loop_and_bridge_target	-0.878343437
TTC12	0	0.021094954	4_EWS-FLI1_loop_and_bridge_target	0.030117032
ZW10	36.9289208	43.05853803	4_EWS-FLI1_loop_and_bridge_target	0.216123301
C11orf71	4.340223348	1.370740196	4_EWS-FLI1_loop_and_bridge_target	-1.171562512
RBM7	7.97305754	11.99193987	4_EWS-FLI1_loop_and_bridge_target	0.533945293
LOC100507548	0.806455592	0.008496983	1_EWS-FLI1_in_promoter	-0.840955021
PRMT8	2.398495251	0.172457796	4_EWS-FLI1_loop_and_bridge_target	-1.535360115
PARP11	4.778934411	2.101004889	4_EWS-FLI1_loop_and_bridge_target	-0.898067696
C12orf4	15.65537312	18.81075861	3_EWS-FLI1_bridge_target	0.250296452
RAD51AP1	42.0282979	20.68138034	3_EWS-FLI1_bridge_target	-0.988829163

RIMKLB	27.80204763	12.62627629	4_EWS-FLI1_loop_and_bridge_target	-1.079780016
M6PR	55.48880981	59.46595581	2_EWS-FLI1_loop_target	0.098157985
ETV6	1.901188824	3.513265243	3_EWS-FLI1_bridge_target	0.637527371
LRP6	16.56935968	18.40480806	4_EWS-FLI1_loop_and_bridge_target	0.14335255
CREBL2	15.88883764	17.75040099	3_EWS-FLI1_bridge_target	0.15085141
CDKN1B	20.79942565	46.68257473	4_EWS-FLI1_loop_and_bridge_target	1.129172015
APOLD1	4.500138869	6.979282997	4_EWS-FLI1_loop_and_bridge_target	0.53679107
MGST1	13.11391754	8.545640666	4_EWS-FLI1_loop_and_bridge_target	-0.564204552
BCAT1	42.51896878	74.85762067	4_EWS-FLI1_loop_and_bridge_target	0.80164975
CASC1	0.3570924	0.126667452	3_EWS-FLI1_bridge_target	-0.268457201
LYRM5	13.05929731	4.531205398	3_EWS-FLI1_bridge_target	-1.345858668
DNM1L	52.09295411	52.09612588	4_EWS-FLI1_loop_and_bridge_target	8.61842E-05
YARS2	31.05686488	15.48570327	4_EWS-FLI1_loop_and_bridge_target	-0.959417906
PKP2	41.9394106	1.984546165	1_EWS-FLI1_in_promoter	-3.846718914
ALG10	7.720230349	5.087494731	4_EWS-FLI1_loop_and_bridge_target	-0.518517627
LRRK2	2.471811007	2.479667354	2_EWS-FLI1_loop_target	0.00326098
YAF2	4.281307254	3.868607247	2_EWS-FLI1_loop_target	-0.117385954
DBX2	2.820379855	1.28370002	2_EWS-FLI1_loop_target	-0.742342936
PLEKHA8P1	3.847766674	1.994649791	4_EWS-FLI1_loop_and_bridge_target	-0.694932965
ANO6	11.40507098	44.65119251	4_EWS-FLI1_loop_and_bridge_target	1.879722557
LINC00938	3.071610878	1.491857272	4_EWS-FLI1_loop_and_bridge_target	-0.708378254
ARID2	20.1615539	9.801050486	4_EWS-FLI1_loop_and_bridge_target	-0.970273936
SCAF11	32.1878119	25.43333699	4_EWS-FLI1_loop_and_bridge_target	-0.328294949
SLC38A1	35.16437265	49.76722251	4_EWS-FLI1_loop_and_bridge_target	0.489328207
SLC38A2	179.8905853	320.4184165	4_EWS-FLI1_loop_and_bridge_target	0.829335272
LOC100288798	2.399149421	8.908589972	4_EWS-FLI1_loop_and_bridge_target	1.543505989
CSAD	6.675005484	4.387184006	3_EWS-FLI1_bridge_target	-0.510636438
ZNF740	23.02591909	14.00636665	3_EWS-FLI1_bridge_target	-0.679016908
SP1	24.06542697	19.86833566	3_EWS-FLI1_bridge_target	-0.264383266
MAP3K12	0.876668383	2.078772969	3_EWS-FLI1_bridge_target	0.714181745
TARBP2	39.46618259	30.80240235	3_EWS-FLI1_bridge_target	-0.347581007
ATF7	1.382515783	1.25195438	3_EWS-FLI1_bridge_target	-0.081308169
ATP5G2	175.5569825	79.7967485	4_EWS-FLI1_loop_and_bridge_target	-1.127764737
CALCOCO1	12.46904294	12.66312736	3_EWS-FLI1_bridge_target	0.020640399
HOXC13-AS	0.944327922	1.267064363	3_EWS-FLI1_bridge_target	0.221553792
HOXC13	5.97394853	2.793479025	3_EWS-FLI1_bridge_target	-0.878454156
HOXC12	1.87931267	1.768013062	3_EWS-FLI1_bridge_target	-0.056873711
HOTAIR	16.36323501	9.383964293	4_EWS-FLI1_loop_and_bridge_target	-0.741678439
HOXC11	15.69960741	9.44063654	4_EWS-FLI1_loop_and_bridge_target	-0.677604514
HOXC-AS2	0.642325466	0.537063296	3_EWS-FLI1_bridge_target	-0.095563483
HOXC-AS1	0.801734705	0.426585777	3_EWS-FLI1_bridge_target	-0.336820103
HOXC9	4.212637509	3.49211994	3_EWS-FLI1_bridge_target	-0.214617089
HOXC5	0.515632305	0.972976866	4_EWS-FLI1_loop_and_bridge_target	0.380454244
HOXC4	1.877693113	3.891430033	4_EWS-FLI1_loop_and_bridge_target	0.765343559
HOXC6	5.883976057	13.5098703	4_EWS-FLI1_loop_and_bridge_target	1.075720639
LOC100240735	0.357714955	0.04078552	3_EWS-FLI1_bridge_target	-0.38350783
CBX5	57.55597741	55.3440524	4_EWS-FLI1_loop_and_bridge_target	-0.055553119
HNRNPA1	29.96062184	29.5264232	4_EWS-FLI1_loop_and_bridge_target	-0.02037589
COPZ1	90.05085046	117.7006844	3_EWS-FLI1_bridge_target	0.382583855
LOC102724050	0.565629888	0.877373418	4_EWS-FLI1_loop_and_bridge_target	0.261972435
ITGA5	36.41849368	168.0560316	4_EWS-FLI1_loop_and_bridge_target	2.175678102
NCKAP1L	175.2771869	35.75694285	4_EWS-FLI1_loop_and_bridge_target	-2.261757094
PDE1B	14.0955038	1.938399313	4_EWS-FLI1_loop_and_bridge_target	-2.361016537
PPP1R1A	85.74939897	4.347193817	4_EWS-FLI1_loop_and_bridge_target	-4.019999887
MYL6	468.176661	760.407034	3_EWS-FLI1_bridge_target	0.698536649
RNF41	15.79837267	30.35843623	3_EWS-FLI1_bridge_target	0.900532138

NABP2	38.04437341	33.83718318	3_EWS-FLI1_bridge_target	-0.164486683
CS	53.56295078	52.33591263	3_EWS-FLI1_bridge_target	-0.032814399
CNPY2	49.56115764	40.26013211	3_EWS-FLI1_bridge_target	-0.293281055
RBMS2	4.009727695	6.596927299	3_EWS-FLI1_bridge_target	0.600683828
BAZ2A	8.107593505	8.704472966	3_EWS-FLI1_bridge_target	0.091579963
NACA	152.3246177	76.130165	3_EWS-FLI1_bridge_target	-0.991222252
PRIM1	67.67878525	22.52019078	3_EWS-FLI1_bridge_target	-1.54596476
NAB2	27.11556459	13.00254716	4_EWS-FLI1_loop_and_bridge_target	-1.005679731
LRP1	4.982595606	19.65457261	3_EWS-FLI1_bridge_target	1.787617756
SHMT2	89.04893232	44.05577202	3_EWS-FLI1_bridge_target	-0.998997232
DDIT3	37.33557664	33.98643306	3_EWS-FLI1_bridge_target	-0.131888295
MBD6	0.877586841	1.141421271	3_EWS-FLI1_bridge_target	0.189689002
LRIG3	14.04490511	156.2590543	4_EWS-FLI1_loop_and_bridge_target	3.385796171
FAM19A2	24.13006807	5.411099225	2_EWS-FLI1_loop_target	-1.970770937
RPSAP52	0.171810833	1.88211152	4_EWS-FLI1_loop_and_bridge_target	1.298386468
HMGA2	5.237133744	40.28935194	4_EWS-FLI1_loop_and_bridge_target	2.726814674
TMBIM4	25.49034779	12.48790994	4_EWS-FLI1_loop_and_bridge_target	-0.973799976
YEATS4	59.2959266	32.97635647	4_EWS-FLI1_loop_and_bridge_target	-0.827529391
FRS2	7.872211033	6.490973131	4_EWS-FLI1_loop_and_bridge_target	-0.244140534
CCT2	393.6706382	218.7480073	4_EWS-FLI1_loop_and_bridge_target	-0.84479911
ZFC3H1	6.895441944	6.20510074	4_EWS-FLI1_loop_and_bridge_target	-0.132001421
THAP2	5.186114576	3.820735272	4_EWS-FLI1_loop_and_bridge_target	-0.359780347
RAB21	7.247968835	20.22616942	4_EWS-FLI1_loop_and_bridge_target	1.363733252
TBC1D15	10.96098735	7.334535989	4_EWS-FLI1_loop_and_bridge_target	-0.521162698
TRHDE-AS1	3.353514148	1.393595452	4_EWS-FLI1_loop_and_bridge_target	-0.863001073
TRHDE	16.14536888	10.12301701	4_EWS-FLI1_loop_and_bridge_target	-0.624270786
ATXN7L3B	46.46096052	42.3108601	4_EWS-FLI1_loop_and_bridge_target	-0.132012475
PAWR	37.20417577	15.02165615	4_EWS-FLI1_loop_and_bridge_target	-1.25370705
PPP1R12A	49.36158818	51.16853636	4_EWS-FLI1_loop_and_bridge_target	0.050856178
PTPRQ	0.480850799	0.759402974	4_EWS-FLI1_loop_and_bridge_target	0.248659665
POC1B	9.981759017	9.983537852	2_EWS-FLI1_loop_target	0.00023367
GALNT4	0.027865065	0.041057726	2_EWS-FLI1_loop_target	0.018399183
EEA1	8.584351181	7.63461651	3_EWS-FLI1_bridge_target	-0.150548667
LOC643339	0.720115747	0.659883371	4_EWS-FLI1_loop_and_bridge_target	-0.051423771
LOC102724933	0.874655953	1.919715613	4_EWS-FLI1_loop_and_bridge_target	0.639202005
NUDT4	0.896409906	1.233770048	4_EWS-FLI1_loop_and_bridge_target	0.236209843
UBE2N	83.42025799	55.65561381	4_EWS-FLI1_loop_and_bridge_target	-0.575370323
MRPL42	99.81227358	43.28817081	3_EWS-FLI1_bridge_target	-1.186677976
FGD6	5.647733794	5.788853502	4_EWS-FLI1_loop_and_bridge_target	0.030305342
VEZT	44.82179444	28.67405493	4_EWS-FLI1_loop_and_bridge_target	-0.626831874
METAP2	86.59515165	79.40126363	4_EWS-FLI1_loop_and_bridge_target	-0.123632844
CCDC38	0.224846785	0.207789075	2_EWS-FLI1_loop_target	-0.020232767
AMDHD1	0.411376984	0.599053238	2_EWS-FLI1_loop_target	0.180114583
ELK3	3.135373607	9.760423057	4_EWS-FLI1_loop_and_bridge_target	1.379645221
CDK17	12.55539809	15.07345014	4_EWS-FLI1_loop_and_bridge_target	0.245812152
LOC643770	0.038011589	0.047192013	4_EWS-FLI1_loop_and_bridge_target	0.012703447
TMPO-AS1	5.471857051	1.082065695	4_EWS-FLI1_loop_and_bridge_target	-1.636164152
TMPO	115.5078095	56.63928465	4_EWS-FLI1_loop_and_bridge_target	-1.015302327
ANO4	3.86853384	4.76551171	4_EWS-FLI1_loop_and_bridge_target	0.243961288
ARL1	32.03501644	42.95451859	4_EWS-FLI1_loop_and_bridge_target	0.412015422
NUP37	52.44416041	20.07785249	4_EWS-FLI1_loop_and_bridge_target	-1.342304434
PARPBP	20.66219346	7.886089718	4_EWS-FLI1_loop_and_bridge_target	-1.285558722
IGF1	8.871209545	11.64462469	4_EWS-FLI1_loop_and_bridge_target	0.357225438
CKAP4	104.0072181	97.34701628	4_EWS-FLI1_loop_and_bridge_target	-0.094535313
TCP11L2	4.567957049	3.95013678	4_EWS-FLI1_loop_and_bridge_target	-0.169679693
POLR3B	19.33874047	12.12523758	4_EWS-FLI1_loop_and_bridge_target	-0.631886802

LOC100287944	0.304794158	0.129739528	4_EWS-FLI1_loop_and_bridge_target	-0.207832044
RIC8B	9.941946775	6.984142211	4_EWS-FLI1_loop_and_bridge_target	-0.45466012
TMEM263	38.57858497	75.24323952	4_EWS-FLI1_loop_and_bridge_target	0.945889386
MTERF2	14.74621379	8.289486061	4_EWS-FLI1_loop_and_bridge_target	-0.761334284
CRY1	12.76226991	40.46303709	4_EWS-FLI1_loop_and_bridge_target	1.591107352
SART3	39.32090144	33.4563061	4_EWS-FLI1_loop_and_bridge_target	-0.226759848
ISCU	17.43287556	22.82737935	4_EWS-FLI1_loop_and_bridge_target	0.370339134
SELPLG	0.112130092	1.303725361	4_EWS-FLI1_loop_and_bridge_target	1.050643178
CORO1C	41.98010594	144.2147016	4_EWS-FLI1_loop_and_bridge_target	1.756446576
SSH1	5.727466131	6.13508268	1_EWS-FLI1_in_promoter	0.084866925
UNG	45.05019967	25.7877052	4_EWS-FLI1_loop_and_bridge_target	-0.781636415
ACACB	8.170429926	1.565723292	1_EWS-FLI1_in_promoter	-1.837623785
ANKRD13A	24.74498043	43.44365822	3_EWS-FLI1_bridge_target	0.787686399
IFT81	21.91877423	13.96592757	4_EWS-FLI1_loop_and_bridge_target	-0.614848188
ATP2A2	29.94346099	35.96081967	4_EWS-FLI1_loop_and_bridge_target	0.256362182
ANAPC7	49.62334388	29.55370194	4_EWS-FLI1_loop_and_bridge_target	-0.728455616
ARPC3	147.8170855	153.8147642	4_EWS-FLI1_loop_and_bridge_target	0.057002893
GPN3	42.05265498	23.50523778	4_EWS-FLI1_loop_and_bridge_target	-0.813012063
FAM216A	10.42923715	8.072994489	4_EWS-FLI1_loop_and_bridge_target	-0.333078425
VPS29	51.74201375	33.95201791	4_EWS-FLI1_loop_and_bridge_target	-0.593576906
RAD9B	0.529172972	0.555020602	4_EWS-FLI1_loop_and_bridge_target	0.024182088
PPP1CC	145.4602422	118.3520209	2_EWS-FLI1_loop_target	-0.295286092
CUX2	4.241938398	0.512351708	4_EWS-FLI1_loop_and_bridge_target	-1.793306713
SH2B3	37.34156559	8.589577893	1_EWS-FLI1_in_promoter	-1.999370029
ATXN2	3.235964969	5.037869785	4_EWS-FLI1_loop_and_bridge_target	0.511348985
BRAP	17.4688336	18.76334347	4_EWS-FLI1_loop_and_bridge_target	0.09773428
ACAD10	9.101409337	5.077832004	4_EWS-FLI1_loop_and_bridge_target	-0.732927887
MAPKAPK5-AS1	22.08643844	5.616056041	4_EWS-FLI1_loop_and_bridge_target	-1.803002265
MAPKAPK5	35.41733833	24.6985609	4_EWS-FLI1_loop_and_bridge_target	-0.502937911
TMEM116	8.620572431	9.937548828	4_EWS-FLI1_loop_and_bridge_target	0.185094814
ERP29	83.49358329	85.84959447	4_EWS-FLI1_loop_and_bridge_target	0.03967733
NAA25	37.16723198	24.13786932	4_EWS-FLI1_loop_and_bridge_target	-0.602472189
TRAFD1	17.45735144	13.73563685	3_EWS-FLI1_bridge_target	-0.324886134
HECTD4	9.530862676	4.577428871	4_EWS-FLI1_loop_and_bridge_target	-0.91695151
RPL6	310.0395003	217.4522616	4_EWS-FLI1_loop_and_bridge_target	-0.509779764
PTPN11	68.83849968	64.14275961	4_EWS-FLI1_loop_and_bridge_target	-0.100417729
OAS1	8.345790414	2.576807816	4_EWS-FLI1_loop_and_bridge_target	-1.385644081
DDX54	15.77648697	12.52268764	4_EWS-FLI1_loop_and_bridge_target	-0.311058727
RITA1	20.12765799	14.38320337	4_EWS-FLI1_loop_and_bridge_target	-0.457776894
TBX3	17.12402358	1.06753235	3_EWS-FLI1_bridge_target	-3.131921465
HSPB8	22.66747959	6.969190087	2_EWS-FLI1_loop_target	-1.570401063
GCN1	36.99181109	33.56671513	4_EWS-FLI1_loop_and_bridge_target	-0.136304977
RPLP0	676.3778624	553.4590231	4_EWS-FLI1_loop_and_bridge_target	-0.288879995
PXN-AS1	13.56207504	2.241421798	4_EWS-FLI1_loop_and_bridge_target	-2.167517276
SRSF9	91.22236673	66.71255602	3_EWS-FLI1_bridge_target	-0.445693311
DYNLL1	229.5216887	193.4195951	3_EWS-FLI1_bridge_target	-0.245728861
RNF10	40.71144623	28.12852675	3_EWS-FLI1_bridge_target	-0.518010599
KDM2B	16.72304299	7.950467313	3_EWS-FLI1_bridge_target	-0.985591417
UBC	144.6685916	154.3213371	4_EWS-FLI1_loop_and_bridge_target	0.092566189
DHX37	24.0764078	13.76352228	4_EWS-FLI1_loop_and_bridge_target	-0.764293736
LINC00939	1.813843038	0.142101619	2_EWS-FLI1_loop_target	-1.300850833
SAP18	73.52156099	60.61450283	4_EWS-FLI1_loop_and_bridge_target	-0.274387922
SKA3	28.44716235	7.654469167	4_EWS-FLI1_loop_and_bridge_target	-1.766611379
MRPL57	21.91745131	10.00510028	4_EWS-FLI1_loop_and_bridge_target	-1.058274318
MIPEPP3	1.821767916	1.944539522	4_EWS-FLI1_loop_and_bridge_target	0.061442703
MICU2	61.62536312	39.27869237	4_EWS-FLI1_loop_and_bridge_target	-0.636730216

SPATA13	8.687984122	3.599285016	4_EWS-FLI1_loop_and_bridge_target	-1.074786897
C1QTNF9	0.064460437	0.018708006	4_EWS-FLI1_loop_and_bridge_target	-0.063381741
PARP4	50.04642046	41.18533349	4_EWS-FLI1_loop_and_bridge_target	-0.275068292
ATP12A	2.861049048	0.289962401	4_EWS-FLI1_loop_and_bridge_target	-1.581663866
CENPJ	11.36960271	13.33598937	2_EWS-FLI1_loop_target	0.212842309
AMER2	60.17944562	2.348189621	1_EWS-FLI1_in_promoter	-4.191593893
LINC00463	15.80731244	0.091732577	1_EWS-FLI1_in_promoter	-3.944397638
LINC01053	0.865350121	0.013847644	1_EWS-FLI1_in_promoter	-0.879605578
MTMR6	46.09664596	24.26038346	4_EWS-FLI1_loop_and_bridge_target	-0.89874778
NUP58	107.4466036	33.71223518	4_EWS-FLI1_loop_and_bridge_target	-1.6434687
RNF6	40.16765396	30.12447132	4_EWS-FLI1_loop_and_bridge_target	-0.403461904
RPL21	0.040854623	0.130427193	3_EWS-FLI1_bridge_target	0.119099495
SNORD102	34.68557515	16.06307496	3_EWS-FLI1_bridge_target	-1.064463365
SNORA27	39.36537497	18.5530957	3_EWS-FLI1_bridge_target	-1.045721257
KATNAL1	24.27251531	16.18772274	4_EWS-FLI1_loop_and_bridge_target	-0.55619085
LINC01058	1.296657015	0.25297187	4_EWS-FLI1_loop_and_bridge_target	-0.874181393
HMGB1	108.7594526	50.88343828	4_EWS-FLI1_loop_and_bridge_target	-1.080999198
USPL1	18.24730133	11.92364995	4_EWS-FLI1_loop_and_bridge_target	-0.5746426
LINC00398	0.191421496	0.095061173	1_EWS-FLI1_in_promoter	-0.121672429
HSPH1	111.9261377	32.08998593	4_EWS-FLI1_loop_and_bridge_target	-1.770912865
BRCA2	14.28832859	6.808816484	3_EWS-FLI1_bridge_target	-0.969254878
N4BP2L2	27.97470844	20.20412828	3_EWS-FLI1_bridge_target	-0.45044897
PDS5B	39.8512494	27.93833508	4_EWS-FLI1_loop_and_bridge_target	-0.497398282
STARD13	4.254776186	11.4118272	4_EWS-FLI1_loop_and_bridge_target	1.240014294
STARD13-AS	1.380252861	1.793087437	4_EWS-FLI1_loop_and_bridge_target	0.230745894
RFC3	40.01250466	15.23674901	3_EWS-FLI1_bridge_target	-1.336801054
CCDC169	1.833069165	1.119238768	4_EWS-FLI1_loop_and_bridge_target	-0.418819683
SPG20	87.25720591	50.30235559	4_EWS-FLI1_loop_and_bridge_target	-0.782689005
SPG20-AS1	1.547533168	1.361791946	4_EWS-FLI1_loop_and_bridge_target	-0.109219049
SMAD9	21.53788962	12.56765832	4_EWS-FLI1_loop_and_bridge_target	-0.732180689
ALG5	76.22438704	50.89828919	4_EWS-FLI1_loop_and_bridge_target	-0.573369533
EXOSC8	48.09110336	31.36510551	4_EWS-FLI1_loop_and_bridge_target	-0.601022386
SUPT20H	21.88285253	16.07096931	4_EWS-FLI1_loop_and_bridge_target	-0.422721928
PROSER1	2.972665852	2.611159639	4_EWS-FLI1_loop_and_bridge_target	-0.137645252
NHLRC3	8.923516472	8.949040072	4_EWS-FLI1_loop_and_bridge_target	0.003705894
LHFP	49.47811036	48.71280389	4_EWS-FLI1_loop_and_bridge_target	-0.022040428
FOXO1	6.153697368	9.166613157	4_EWS-FLI1_loop_and_bridge_target	0.507078156
MRPS31	47.9484687	22.27334106	4_EWS-FLI1_loop_and_bridge_target	-1.072585392
ELF1	12.46169812	17.84832825	4_EWS-FLI1_loop_and_bridge_target	0.48557616
KBTBD6	15.07597333	5.549342765	3_EWS-FLI1_bridge_target	-1.295484047
LOC101929140	0.788445314	0.742957469	3_EWS-FLI1_bridge_target	-0.037168639
TSC22D1	10.3357557	21.99410301	4_EWS-FLI1_loop_and_bridge_target	1.020383348
NUFIP1	14.21442125	7.547309606	4_EWS-FLI1_loop_and_bridge_target	-0.831897168
GPALPP1	22.05885989	17.1175571	4_EWS-FLI1_loop_and_bridge_target	-0.347932742
GTF2F2	82.74725761	49.84114058	4_EWS-FLI1_loop_and_bridge_target	-0.720045552
TPT1	233.040856	199.5365908	4_EWS-FLI1_loop_and_bridge_target	-0.222894898
TPT1-AS1	3.477649408	3.128315315	4_EWS-FLI1_loop_and_bridge_target	-0.117188406
SLC25A30	22.92457635	4.422202037	4_EWS-FLI1_loop_and_bridge_target	-2.141542602
SLC25A30-AS1	0.197920543	0.10530926	4_EWS-FLI1_loop_and_bridge_target	-0.116082134
COG3	17.52139766	19.4298869	4_EWS-FLI1_loop_and_bridge_target	0.141488246
ZC3H13	10.6853862	11.83919905	4_EWS-FLI1_loop_and_bridge_target	0.135849789
CPB2-AS1	0.589957996	0.48751563	4_EWS-FLI1_loop_and_bridge_target	-0.096083825
CPB2	19.18901864	0.787265234	1_EWS-FLI1_in_promoter	-3.497745131
LRRC63	1.476680115	0.291003592	4_EWS-FLI1_loop_and_bridge_target	-0.939914529
LINC00563	1.337872184	0.237284329	4_EWS-FLI1_loop_and_bridge_target	-0.918018986
KIAA0226L	35.35220482	1.380001954	1_EWS-FLI1_in_promoter	-3.933008205

LINC01198	0.792247255	0.54009519	4_EWS-FLI1_loop_and_bridge_target	-0.21875016
LRCH1	3.387587311	3.432899022	4_EWS-FLI1_loop_and_bridge_target	0.014822665
INTS6	17.87794356	15.17359639	4_EWS-FLI1_loop_and_bridge_target	-0.223061101
INTS6-AS1	4.377933669	3.236152223	4_EWS-FLI1_loop_and_bridge_target	-0.344297529
WDFY2	4.040124312	8.256257047	4_EWS-FLI1_loop_and_bridge_target	0.876969611
DHRS12	1.722421508	1.416755021	4_EWS-FLI1_loop_and_bridge_target	-0.171819215
MRPS31P5	7.793916552	3.256326035	4_EWS-FLI1_loop_and_bridge_target	-1.046897177
VPS36	25.4882581	13.93829031	4_EWS-FLI1_loop_and_bridge_target	-0.826337933
CKAP2	54.84731755	41.65597264	4_EWS-FLI1_loop_and_bridge_target	-0.388740227
SUGT1	71.16836068	37.53386435	4_EWS-FLI1_loop_and_bridge_target	-0.905239611
DIAPH3	15.76310198	28.85055604	4_EWS-FLI1_loop_and_bridge_target	0.832468664
TDRD3	18.03753106	11.11523518	4_EWS-FLI1_loop_and_bridge_target	-0.65202398
PCDH9	5.552937457	10.06911965	4_EWS-FLI1_loop_and_bridge_target	0.756326819
KLF12	3.587578204	6.019294468	3_EWS-FLI1_bridge_target	0.613593276
SLAIN1	56.75969552	4.311647711	1_EWS-FLI1_in_promoter	-3.442831767
RNF219	50.02941097	9.239909172	4_EWS-FLI1_loop_and_bridge_target	-2.31712607
RBM26	31.49442278	18.34154705	3_EWS-FLI1_bridge_target	-0.748488926
RBM26-AS1	3.780648148	2.748524807	3_EWS-FLI1_bridge_target	-0.350883278
SLITRK6	13.32337567	6.241569773	4_EWS-FLI1_loop_and_bridge_target	-0.983997168
GPC6	6.134199153	29.36275088	4_EWS-FLI1_loop_and_bridge_target	2.089479111
DCT	0.090344234	0.194625776	4_EWS-FLI1_loop_and_bridge_target	0.131775074
GPR180	15.37238636	24.33554412	4_EWS-FLI1_loop_and_bridge_target	0.629898196
FARP1	9.066683077	31.92526747	4_EWS-FLI1_loop_and_bridge_target	1.70960676
RNF113B	0.025170954	0.142857845	4_EWS-FLI1_loop_and_bridge_target	0.156781455
UBAC2-AS1	1.536501773	0.513495894	4_EWS-FLI1_loop_and_bridge_target	-0.744955408
UBAC2	25.60649578	30.74235777	4_EWS-FLI1_loop_and_bridge_target	0.254630781
LINC01232	0.852663194	0.392741139	4_EWS-FLI1_loop_and_bridge_target	-0.411673492
LINC00449	0.690843849	0.44387398	4_EWS-FLI1_loop_and_bridge_target	-0.227798601
TM9SF2	96.33346982	98.33804668	4_EWS-FLI1_loop_and_bridge_target	0.029410393
CLYBL	3.202894147	1.614276651	2_EWS-FLI1_loop_target	-0.684971302
ZIC5	9.851598713	9.306010018	4_EWS-FLI1_loop_and_bridge_target	-0.074421703
ZIC2	17.88104552	8.122464879	4_EWS-FLI1_loop_and_bridge_target	-1.049443058
GGACT	0.127447285	0.072050204	4_EWS-FLI1_loop_and_bridge_target	-0.072687512
TMTC4	27.59189328	16.20619012	2_EWS-FLI1_loop_target	-0.732678471
FGF14-IT1	0.233408409	0.011552706	2_EWS-FLI1_loop_target	-0.286079095
FGF14-AS2	1.998725518	0.567188704	2_EWS-FLI1_loop_target	-0.93617057
TPP2	45.50119044	33.30339764	4_EWS-FLI1_loop_and_bridge_target	-0.438916172
ERCC5	1.180243561	0.478892893	4_EWS-FLI1_loop_and_bridge_target	-0.55997174
LIG4	14.52664163	9.0808486	4_EWS-FLI1_loop_and_bridge_target	-0.623128723
ABHD13	11.73413051	13.98372043	4_EWS-FLI1_loop_and_bridge_target	0.234695432
TNFSF13B	0.567054829	1.179232408	4_EWS-FLI1_loop_and_bridge_target	0.475764405
LINC00399	0.168376578	0	4_EWS-FLI1_loop_and_bridge_target	-0.224505343
IRS2	24.64536174	6.433122186	4_EWS-FLI1_loop_and_bridge_target	-1.786657693
OSGEP	33.69659157	23.56240149	4_EWS-FLI1_loop_and_bridge_target	-0.498342325
APEX1	63.69072663	27.37221388	4_EWS-FLI1_loop_and_bridge_target	-1.18908019
TMEM55B	29.94321116	17.25704212	3_EWS-FLI1_bridge_target	-0.761169873
LOC101929718	0.860109636	0.686468732	3_EWS-FLI1_bridge_target	-0.141382088
SLC39A2	0.277223685	0.095444253	3_EWS-FLI1_bridge_target	-0.221495144
NDRG2	8.598133163	3.518867068	4_EWS-FLI1_loop_and_bridge_target	-1.086792711
ARHGEF40	14.75320175	16.46651485	4_EWS-FLI1_loop_and_bridge_target	0.148946693
ZNF219	2.469942719	1.326584806	4_EWS-FLI1_loop_and_bridge_target	-0.576698072
TMEM253	0.38640549	1.225525587	3_EWS-FLI1_bridge_target	0.682796815
HNRNPC	186.2451435	137.0581448	4_EWS-FLI1_loop_and_bridge_target	-0.439652298
SUPT16H	114.5349681	114.9091973	4_EWS-FLI1_loop_and_bridge_target	0.00466548
RAB2B	19.95787813	11.14376252	4_EWS-FLI1_loop_and_bridge_target	-0.787277176
TOX4	26.33477642	18.95292123	4_EWS-FLI1_loop_and_bridge_target	-0.454137594

METTL3	43.80011632	16.75776568	4_EWS-FLI1_loop_and_bridge_target	-1.335052408
SALL2	15.81041385	5.339972199	4_EWS-FLI1_loop_and_bridge_target	-1.406806823
PRMT5	79.02813063	27.70198463	3_EWS-FLI1_bridge_target	-1.479356713
LOC101926933	0.707020117	0.243417178	3_EWS-FLI1_bridge_target	-0.457169646
HAUS4	33.01270553	10.7788228	3_EWS-FLI1_bridge_target	-1.529878408
AJUBA	23.22603409	61.75012477	4_EWS-FLI1_loop_and_bridge_target	1.373060079
C14orf93	6.574936333	2.964730574	3_EWS-FLI1_bridge_target	-0.934010933
PSMB5	194.5954596	98.16673553	3_EWS-FLI1_bridge_target	-0.979944712
ACIN1	51.44498682	39.23382517	3_EWS-FLI1_bridge_target	-0.382395967
C14orf119	30.74001736	26.63349856	3_EWS-FLI1_bridge_target	-0.19988469
SLC7A8	3.60406467	7.489386067	4_EWS-FLI1_loop_and_bridge_target	0.882752127
PPP1R3E	1.720774918	0.75819934	3_EWS-FLI1_bridge_target	-0.629918963
PABPN1	1.352983428	2.114066987	3_EWS-FLI1_bridge_target	0.404308819
THTPA	4.823564095	1.182068558	3_EWS-FLI1_bridge_target	-1.416205941
TINF2	29.51274553	9.421292764	3_EWS-FLI1_bridge_target	-1.549877744
LRFN5	8.460727557	4.06164911	4_EWS-FLI1_loop_and_bridge_target	-0.902343637
WDHD1	30.95365567	15.38565903	4_EWS-FLI1_loop_and_bridge_target	-0.963547293
SOCS4	17.89515816	14.91631127	4_EWS-FLI1_loop_and_bridge_target	-0.247510576
MAPK1IP1L	16.74494531	12.04687036	4_EWS-FLI1_loop_and_bridge_target	-0.443704349
DLGAP5	109.0128787	38.83782632	4_EWS-FLI1_loop_and_bridge_target	-1.465461587
FBXO34	46.52731157	29.50954558	4_EWS-FLI1_loop_and_bridge_target	-0.639496104
ATG14	14.99165698	10.56561093	4_EWS-FLI1_loop_and_bridge_target	-0.467477956
KTN1-AS1	2.99751884	1.098538561	4_EWS-FLI1_loop_and_bridge_target	-0.92971986
KTN1	88.56717692	152.7648786	4_EWS-FLI1_loop_and_bridge_target	0.779683976
PELI2	18.37190664	8.272982814	4_EWS-FLI1_loop_and_bridge_target	-1.062860569
LOC101927690	0.114268142	0.011054476	4_EWS-FLI1_loop_and_bridge_target	-0.140235718
TMEM260	9.299932534	8.727699463	4_EWS-FLI1_loop_and_bridge_target	-0.082464325
OTX2	4.734361299	1.573778823	4_EWS-FLI1_loop_and_bridge_target	-1.155744725
OTX2-AS1	2.024635275	0.253474081	4_EWS-FLI1_loop_and_bridge_target	-1.270829021
EXOC5	28.18126567	33.65970682	4_EWS-FLI1_loop_and_bridge_target	0.248216993
AP5M1	36.52351838	32.53406246	4_EWS-FLI1_loop_and_bridge_target	-0.16216784
RHOJ	2.571786681	1.791659022	4_EWS-FLI1_loop_and_bridge_target	-0.355523181
FNTB	1.152494574	0.580483519	4_EWS-FLI1_loop_and_bridge_target	-0.44564361
MAX	16.21401213	15.95785511	4_EWS-FLI1_loop_and_bridge_target	-0.021629686
FUT8	28.72562231	30.46406252	4_EWS-FLI1_loop_and_bridge_target	0.081997945
FUT8-AS1	0.296897104	0.109365617	4_EWS-FLI1_loop_and_bridge_target	-0.225329104
GPHN	5.254168558	5.122223315	4_EWS-FLI1_loop_and_bridge_target	-0.030762433
MPP5	46.95664881	33.53002961	4_EWS-FLI1_loop_and_bridge_target	-0.473879276
ATP6V1D	102.4626486	58.70453817	4_EWS-FLI1_loop_and_bridge_target	-0.793197529
EIF2S1	83.63123829	65.09847198	4_EWS-FLI1_loop_and_bridge_target	-0.356573355
PLEK2	0.394577266	0.316899727	4_EWS-FLI1_loop_and_bridge_target	-0.082682371
VTI1B	39.69281607	33.44182651	3_EWS-FLI1_bridge_target	-0.24061247
ZFP36L1	3.757603974	12.3675864	4_EWS-FLI1_loop_and_bridge_target	1.490431911
LOC100289511	0.93722961	1.134926211	2_EWS-FLI1_loop_target	0.140191248
SRSF5	78.66340759	70.92206898	2_EWS-FLI1_loop_target	-0.147482669
PCNX1	12.26349914	17.97021673	4_EWS-FLI1_loop_and_bridge_target	0.516274728
SNORD56B	1.139901531	0.51987177	4_EWS-FLI1_loop_and_bridge_target	-0.493594802
DPF3	2.902626438	0.382100369	1_EWS-FLI1_in_promoter	-1.497582985
DCAF4	21.34079222	8.826832579	3_EWS-FLI1_bridge_target	-1.184881964
EIF2B2	45.16702626	30.09240011	4_EWS-FLI1_loop_and_bridge_target	-0.570300821
MLH3	0.682348785	1.14061025	4_EWS-FLI1_loop_and_bridge_target	0.347545306
ACYP1	26.60648968	3.201969773	4_EWS-FLI1_loop_and_bridge_target	-2.715869764
ZC2HC1C	0.359676478	0.419792759	4_EWS-FLI1_loop_and_bridge_target	0.062416944
NEK9	13.78537291	9.842017638	4_EWS-FLI1_loop_and_bridge_target	-0.447537372
TMED10	99.71823852	92.78791109	4_EWS-FLI1_loop_and_bridge_target	-0.102851075
FOS	25.56681566	19.96061873	4_EWS-FLI1_loop_and_bridge_target	-0.341944011

LINC01220	1.540001629	0.655521371	4_EWS-FLI1_loop_and_bridge_target	-0.617543788
TGFB3	25.82527303	27.4963757	4_EWS-FLI1_loop_and_bridge_target	0.087185588
IRF2BPL	7.681946801	3.252752331	4_EWS-FLI1_loop_and_bridge_target	-1.029621744
LOC283575	0.062943087	0.071167656	4_EWS-FLI1_loop_and_bridge_target	0.011119951
POMT2	3.691065171	8.148568674	3_EWS-FLI1_bridge_target	0.963630503
GSTZ1	14.14636321	15.77388703	3_EWS-FLI1_bridge_target	0.147245614
FLRT2	36.21878161	32.45663471	4_EWS-FLI1_loop_and_bridge_target	-0.153738491
ITPK1	14.68599544	12.47640104	4_EWS-FLI1_loop_and_bridge_target	-0.21904182
MOAP1	42.26751372	17.62315829	4_EWS-FLI1_loop_and_bridge_target	-1.216186463
TMEM251	16.12038845	6.269251488	4_EWS-FLI1_loop_and_bridge_target	-1.235836713
C14orf142	40.73195517	17.18951291	4_EWS-FLI1_loop_and_bridge_target	-1.198045603
UBR7	61.86877494	24.47070477	4_EWS-FLI1_loop_and_bridge_target	-1.303504772
BTBD7	11.48462647	19.50654089	4_EWS-FLI1_loop_and_bridge_target	0.715931496
UNC79	0.363056821	0.213508899	4_EWS-FLI1_loop_and_bridge_target	-0.167661015
SNHG10	16.51441373	6.161227184	2_EWS-FLI1_loop_target	-1.290263956
GLRX5	63.56553737	48.20586087	2_EWS-FLI1_loop_target	-0.391934151
AK7	1.051078035	0.727032793	2_EWS-FLI1_loop_target	-0.248086904
LOC730202	1.58358296	0.6885685	4_EWS-FLI1_loop_and_bridge_target	-0.613572504
PAPOLA	39.78913311	38.49598078	4_EWS-FLI1_loop_and_bridge_target	-0.046478998
VRK1	92.46092149	31.81749548	4_EWS-FLI1_loop_and_bridge_target	-1.50989812
LINC01550	8.366090942	3.046372503	4_EWS-FLI1_loop_and_bridge_target	-1.210817908
BCL11B	53.26821139	5.052703731	4_EWS-FLI1_loop_and_bridge_target	-3.16445572
SETD3	52.75711016	19.5478612	3_EWS-FLI1_bridge_target	-1.387467351
CCNK	3.819938629	3.677870006	3_EWS-FLI1_bridge_target	-0.043163006
CCDC85C	23.35002011	14.629411	4_EWS-FLI1_loop_and_bridge_target	-0.639659553
YY1	50.11968619	61.57658121	3_EWS-FLI1_bridge_target	0.291743861
DYNC1H1	63.40316732	56.74942956	4_EWS-FLI1_loop_and_bridge_target	-0.157324945
ZNF839	6.209295373	4.478464845	4_EWS-FLI1_loop_and_bridge_target	-0.396086576
RCOR1	35.23795719	26.70204473	4_EWS-FLI1_loop_and_bridge_target	-0.387509162
TRAF3	18.94562751	9.067915936	4_EWS-FLI1_loop_and_bridge_target	-0.986307437
EIF5	84.76360045	64.03030629	3_EWS-FLI1_bridge_target	-0.399253254
MARK3	88.43675302	49.93601529	4_EWS-FLI1_loop_and_bridge_target	-0.81218171
CKB	76.57153946	45.96562611	3_EWS-FLI1_bridge_target	-0.723922191
BAG5	31.97665857	16.40406192	3_EWS-FLI1_bridge_target	-0.922021167
APOPT1	11.82209314	11.86845519	3_EWS-FLI1_bridge_target	0.005207081
XRCC3	22.97475762	7.343016375	4_EWS-FLI1_loop_and_bridge_target	-1.522875248
ZFYVE21	30.01326476	35.06575908	4_EWS-FLI1_loop_and_bridge_target	0.217744383
PPP1R13B	2.965655145	1.563074831	2_EWS-FLI1_loop_target	-0.629683626
SIVA1	29.97685461	24.24022935	3_EWS-FLI1_bridge_target	-0.295465641
CRIP1	72.08020497	78.17182014	3_EWS-FLI1_bridge_target	0.115506338
C14orf80	4.776041109	1.778259295	3_EWS-FLI1_bridge_target	-1.055899758
DPH6	8.48953628	2.477119076	3_EWS-FLI1_bridge_target	-1.448445116
DPH6-AS1	0.125246943	0.241574357	3_EWS-FLI1_bridge_target	0.141929021
MEIS2	8.798202952	11.51065696	4_EWS-FLI1_loop_and_bridge_target	0.352568471
FSIP1	1.627620305	2.388120515	4_EWS-FLI1_loop_and_bridge_target	0.366728372
LOC105370941	0.417949879	0.394841746	4_EWS-FLI1_loop_and_bridge_target	-0.023705089
GPR176	12.19826875	73.86041718	4_EWS-FLI1_loop_and_bridge_target	2.503854388
EIF2AK4	25.19206512	48.70317311	4_EWS-FLI1_loop_and_bridge_target	0.924208144
SRP14	114.7367472	113.8190217	4_EWS-FLI1_loop_and_bridge_target	-0.011485333
SRP14-AS1	1.473092197	0.922772391	4_EWS-FLI1_loop_and_bridge_target	-0.363128031
BMF	16.31920739	42.8798824	1_EWS-FLI1_in_promoter	1.341186752
BUB1B	92.71423957	33.62291278	4_EWS-FLI1_loop_and_bridge_target	-1.436541175
ANKRD63	13.62187158	3.893960838	4_EWS-FLI1_loop_and_bridge_target	-1.579053525
PLCB2	9.603337176	7.016572986	4_EWS-FLI1_loop_and_bridge_target	-0.40346086
INAFM2	35.72875034	36.36509543	4_EWS-FLI1_loop_and_bridge_target	0.024781397
C15orf52	2.172596253	1.251843154	4_EWS-FLI1_loop_and_bridge_target	-0.494557591

BAHD1	9.745746935	11.82747725	4_EWS-FLI1_loop_and_bridge_target	0.2554717
RAD51-AS1	1.212212465	1.08402043	4_EWS-FLI1_loop_and_bridge_target	-0.08612053
RAD51	10.20023456	9.176882457	4_EWS-FLI1_loop_and_bridge_target	-0.138233267
RMDN3	19.36035489	19.73482946	4_EWS-FLI1_loop_and_bridge_target	0.026293473
GCHFR	11.46456364	4.003425802	4_EWS-FLI1_loop_and_bridge_target	-1.316844239
DNAJC17	11.8003643	13.01671074	4_EWS-FLI1_loop_and_bridge_target	0.130962966
C15orf62	1.105109355	1.284716582	4_EWS-FLI1_loop_and_bridge_target	0.118120031
ZFYVE19	10.07140605	7.950907712	4_EWS-FLI1_loop_and_bridge_target	-0.306732555
VPS18	13.18556481	13.47555677	4_EWS-FLI1_loop_and_bridge_target	0.029195245
CTDSPL2	44.2145011	22.32021196	4_EWS-FLI1_loop_and_bridge_target	-0.955204644
SPG11	13.12808342	12.62433518	4_EWS-FLI1_loop_and_bridge_target	-0.052379934
B2M	451.662182	373.1977014	4_EWS-FLI1_loop_and_bridge_target	-0.274634084
LOC100419583	21.17610089	6.846665648	4_EWS-FLI1_loop_and_bridge_target	-1.498854094
SORD	44.30848833	10.0704675	4_EWS-FLI1_loop_and_bridge_target	-2.03306521
SHF	5.233457566	3.542972006	4_EWS-FLI1_loop_and_bridge_target	-0.456396202
BLOC1S6	35.18804285	8.063995218	3_EWS-FLI1_bridge_target	-1.99729408
ZNF280D	9.840482851	8.766752666	4_EWS-FLI1_loop_and_bridge_target	-0.150478151
LOC145783	0.482861835	0.053015318	4_EWS-FLI1_loop_and_bridge_target	-0.493857758
TCF12	40.75729554	44.66771253	4_EWS-FLI1_loop_and_bridge_target	0.12914625
FAM96A	31.55292799	19.0679518	3_EWS-FLI1_bridge_target	-0.697893935
SNX1	13.87562054	11.42321565	3_EWS-FLI1_bridge_target	-0.2599112
TIPIN	50.64635933	26.93341917	3_EWS-FLI1_bridge_target	-0.886674478
MAP2K5	13.87782768	10.76747248	3_EWS-FLI1_bridge_target	-0.338359415
FEM1B	33.48101197	40.37809435	3_EWS-FLI1_bridge_target	0.263065088
ANP32A	120.6587289	83.34138968	4_EWS-FLI1_loop_and_bridge_target	-0.528527138
EWSAT1	12.81599504	0.180219469	1_EWS-FLI1_in_promoter	-3.549212402
GLCE	20.54566971	7.414997931	4_EWS-FLI1_loop_and_bridge_target	-1.356363121
LOC145694	0.432997217	1.803233049	4_EWS-FLI1_loop_and_bridge_target	0.968055881
KIF23	57.29066974	47.43656514	4_EWS-FLI1_loop_and_bridge_target	-0.267168417
RPLP1	147.6395119	197.2666291	4_EWS-FLI1_loop_and_bridge_target	0.415624204
TLE3	7.944620455	6.547364174	4_EWS-FLI1_loop_and_bridge_target	-0.245047377
UACA	8.769308175	20.3902795	4_EWS-FLI1_loop_and_bridge_target	1.130627029
UBL7	35.05242417	21.95915262	3_EWS-FLI1_bridge_target	-0.651026874
UBL7-AS1	6.604814586	1.958060181	3_EWS-FLI1_bridge_target	-1.36226167
CLK3	12.49907918	8.935898401	3_EWS-FLI1_bridge_target	-0.442138673
CSK	12.56191694	11.48189296	4_EWS-FLI1_loop_and_bridge_target	-0.119724368
ULK3	24.78947777	16.94478188	4_EWS-FLI1_loop_and_bridge_target	-0.523218172
SCAMP2	25.2615026	18.08103067	1_EWS-FLI1_in_promoter	-0.460810363
FAM219B	25.95213963	12.28192597	4_EWS-FLI1_loop_and_bridge_target	-1.020935445
COX5A	152.2887692	90.60528914	4_EWS-FLI1_loop_and_bridge_target	-0.742749197
RPP25	22.0483103	13.7169938	4_EWS-FLI1_loop_and_bridge_target	-0.647177983
PPCDC	8.12480717	4.333194878	3_EWS-FLI1_bridge_target	-0.774794025
C15orf39	2.538542206	3.037693618	4_EWS-FLI1_loop_and_bridge_target	0.190376315
COMMD4	24.97109495	20.31802668	4_EWS-FLI1_loop_and_bridge_target	-0.28483294
MAN2C1	20.34082048	12.96090874	4_EWS-FLI1_loop_and_bridge_target	-0.612222792
SIN3A	11.04255878	11.38671124	4_EWS-FLI1_loop_and_bridge_target	0.040651227
PTPN9	18.9385878	23.78078406	3_EWS-FLI1_bridge_target	0.313658604
IMP3	53.23680554	23.5546528	4_EWS-FLI1_loop_and_bridge_target	-1.143275785
LOC101929457	0.307578036	0.523698085	4_EWS-FLI1_loop_and_bridge_target	0.220680017
LINGO1	1.7389787	1.732090239	2_EWS-FLI1_loop_target	-0.003632911
LINGO1-AS2	0.087062187	0.037059132	2_EWS-FLI1_loop_target	-0.067936316
TBC1D2B	20.92263774	33.48769957	4_EWS-FLI1_loop_and_bridge_target	0.653660505
IREB2	26.86539784	33.09223425	3_EWS-FLI1_bridge_target	0.290968399
SH3GL3	22.4563505	4.803956601	4_EWS-FLI1_loop_and_bridge_target	-2.01486993
AKAP13	11.86947214	11.98442109	4_EWS-FLI1_loop_and_bridge_target	0.012828813
FANCI	79.67189209	36.28392599	3_EWS-FLI1_bridge_target	-1.11351231

POLG	28.08325494	19.22165871	4_EWS-FLI1_loop_and_bridge_target	-0.5242874
TICRR	9.831653754	4.368720992	4_EWS-FLI1_loop_and_bridge_target	-1.012603191
AP3S2	0.626514051	0.447183052	4_EWS-FLI1_loop_and_bridge_target	-0.168535867
CRTC3-AS1	1.04304227	0.526760998	3_EWS-FLI1_bridge_target	-0.420244816
BLM	32.48616553	13.0880489	3_EWS-FLI1_bridge_target	-1.249093362
PRC1	127.8944504	87.12437009	3_EWS-FLI1_bridge_target	-0.548577204
LINC00924	0.047384665	0.036672524	2_EWS-FLI1_loop_target	-0.014831157
NR2F2	2.060697836	0.970597474	3_EWS-FLI1_bridge_target	-0.63522751
UBE2I	42.24998249	28.49032489	4_EWS-FLI1_loop_and_bridge_target	-0.552457832
UNKL	4.474901668	2.350485835	3_EWS-FLI1_bridge_target	-0.708462746
C16orf91	24.32955475	12.42943589	3_EWS-FLI1_bridge_target	-0.915423013
CLCN7	11.21987251	13.28768952	3_EWS-FLI1_bridge_target	0.225543402
IFT140	6.50519139	5.290001179	3_EWS-FLI1_bridge_target	-0.254828575
MAPK8IP3	2.336407957	5.924211757	3_EWS-FLI1_bridge_target	1.053354142
NDUFB10	137.9792511	79.32929939	4_EWS-FLI1_loop_and_bridge_target	-0.790871313
RPS2	335.1834295	270.9150027	4_EWS-FLI1_loop_and_bridge_target	-0.306092863
SNORA10	31.38218704	17.94665102	4_EWS-FLI1_loop_and_bridge_target	-0.773257564
SNORA64	40.38022181	29.84089822	4_EWS-FLI1_loop_and_bridge_target	-0.424096595
SNHG9	17.02024646	8.871288224	4_EWS-FLI1_loop_and_bridge_target	-0.868308466
SNORA78	0.18207651	0.678152938	4_EWS-FLI1_loop_and_bridge_target	0.505550785
RNF151	0.101674634	0.07728412	4_EWS-FLI1_loop_and_bridge_target	-0.032299412
SLC9A3R2	2.906366432	4.941238534	3_EWS-FLI1_bridge_target	0.604936426
PKD1	1.992944467	3.685519948	3_EWS-FLI1_bridge_target	0.646643641
RAB26	6.888993384	2.493068892	3_EWS-FLI1_bridge_target	-1.175346131
SNHG19	22.67031055	10.16686668	3_EWS-FLI1_bridge_target	-1.083854198
SNORD60	32.31748367	15.71307204	3_EWS-FLI1_bridge_target	-0.995302503
TRAF7	27.46217249	15.6555076	3_EWS-FLI1_bridge_target	-0.773046463
MLST8	7.318201076	4.336476964	3_EWS-FLI1_bridge_target	-0.640383942
PGP	6.474050803	6.779552262	3_EWS-FLI1_bridge_target	0.057796756
ATP6V0C	8.556338738	9.46510105	3_EWS-FLI1_bridge_target	0.131056344
SRRM2-AS1	0.442794576	0.291697048	3_EWS-FLI1_bridge_target	-0.159598162
SRRM2	6.319279381	7.562662214	3_EWS-FLI1_bridge_target	0.226357799
CPPED1	8.507178884	8.61234203	4_EWS-FLI1_loop_and_bridge_target	0.015870678
ERCC4	6.525669514	4.695070294	4_EWS-FLI1_loop_and_bridge_target	-0.402106287
LOC101927814	0.19949193	0.12922044	3_EWS-FLI1_bridge_target	-0.087096303
METTL9	64.5987645	35.76019626	3_EWS-FLI1_bridge_target	-0.835524174
USP31	8.49469124	7.067285279	4_EWS-FLI1_loop_and_bridge_target	-0.235037812
GGA2	50.54465864	35.69350967	4_EWS-FLI1_loop_and_bridge_target	-0.490298032
EARS2	40.86976074	16.76244926	4_EWS-FLI1_loop_and_bridge_target	-1.237078145
UBFD1	43.88394676	48.14410407	4_EWS-FLI1_loop_and_bridge_target	0.130818802
PALB2	19.07507192	16.06021145	4_EWS-FLI1_loop_and_bridge_target	-0.234769628
DCTN5	29.74458486	28.15123617	4_EWS-FLI1_loop_and_bridge_target	-0.076775263
PLK1	33.187662	18.09071565	4_EWS-FLI1_loop_and_bridge_target	-0.840604578
RBBP6	16.58272604	16.65297253	4_EWS-FLI1_loop_and_bridge_target	0.005752371
ARHGAP17	4.418481644	11.83028041	4_EWS-FLI1_loop_and_bridge_target	1.243592157
ZKSCAN2	11.42232431	5.815132217	4_EWS-FLI1_loop_and_bridge_target	-0.866121586
KDM8	3.189030549	1.82344245	4_EWS-FLI1_loop_and_bridge_target	-0.56916118
IRX3	1.397642326	1.055510881	4_EWS-FLI1_loop_and_bridge_target	-0.222119449
CRNDE	16.69955742	6.489732591	4_EWS-FLI1_loop_and_bridge_target	-1.240727171
IRX5	1.637275775	1.169501382	4_EWS-FLI1_loop_and_bridge_target	-0.281684934
KATNB1	7.594297002	2.69229133	4_EWS-FLI1_loop_and_bridge_target	-1.218863245
KIFC3	9.412154983	5.957250169	4_EWS-FLI1_loop_and_bridge_target	-0.581679588
LOC388282	0.098665975	0	4_EWS-FLI1_loop_and_bridge_target	-0.135752834
CDH11	78.71811868	107.228957	4_EWS-FLI1_loop_and_bridge_target	0.441106979
LINC00920	3.930017652	1.55144481	2_EWS-FLI1_loop_target	-0.950278377
CMTM3	7.0200181	19.98747292	3_EWS-FLI1_bridge_target	1.387851066

PSMD7	67.77187527	42.76064637	2_EWS-FLI1_loop_target	-0.652184638
GLG1	49.53787129	25.46113947	2_EWS-FLI1_loop_target	-0.93348971
WWOX	16.11039587	3.38318199	1_EWS-FLI1_in_promoter	-1.964822653
CMC2	26.38637817	16.04528432	3_EWS-FLI1_bridge_target	-0.684085819
CENPN	13.29820681	9.48680265	3_EWS-FLI1_bridge_target	-0.447259348
MPHOSPH6	62.13580022	25.14366188	3_EWS-FLI1_bridge_target	-1.271997066
ANKFY1	8.94875449	21.09386669	3_EWS-FLI1_bridge_target	1.151058102
UBE2G1	50.04735039	40.73823741	2_EWS-FLI1_loop_target	-0.290466403
SNORA48	168.4605588	137.344987	3_EWS-FLI1_bridge_target	-0.292679163
SNORD10	113.8218085	93.02543295	3_EWS-FLI1_bridge_target	-0.288273735
SNORA67	91.98850829	51.72913842	3_EWS-FLI1_bridge_target	-0.818452012
TMEM88	0.079372026	0.21116073	3_EWS-FLI1_bridge_target	0.166198132
NAA38	47.29949756	26.16541602	3_EWS-FLI1_bridge_target	-0.83023704
CYB5D1	17.24710748	10.7336543	3_EWS-FLI1_bridge_target	-0.637015395
AURKB	69.62994106	23.65148459	3_EWS-FLI1_bridge_target	-1.51860536
LINC00324	1.231277182	0.78733843	2_EWS-FLI1_loop_target	-0.320056912
NDEL1	18.25756675	30.39750565	2_EWS-FLI1_loop_target	0.705224524
TMEM220	5.257429782	3.991119915	3_EWS-FLI1_bridge_target	-0.326206631
TMEM220-AS1	0.510081987	0.723743052	3_EWS-FLI1_bridge_target	0.190917856
DNAH9	0.109673145	0.199195809	2_EWS-FLI1_loop_target	0.111932454
WSB1	51.89363767	56.95208698	4_EWS-FLI1_loop_and_bridge_target	0.131766419
GIT1	10.23039969	8.956127782	3_EWS-FLI1_bridge_target	-0.173752622
ANKRD13B	3.552729884	2.648402324	3_EWS-FLI1_bridge_target	-0.319467036
NSRP1	15.91077211	14.11370894	2_EWS-FLI1_loop_target	-0.162084787
BLMH	48.71293033	33.29788589	3_EWS-FLI1_bridge_target	-0.535501494
SUZ12P1	18.53391309	11.06988858	3_EWS-FLI1_bridge_target	-0.694568626
CRLF3	26.93704646	11.4418338	3_EWS-FLI1_bridge_target	-1.166980366
ATAD5	9.074332551	5.948398284	3_EWS-FLI1_bridge_target	-0.535931903
LOC105371730	0.115690891	0.07694579	3_EWS-FLI1_bridge_target	-0.050991743
RHOT1	31.67917214	23.27649749	3_EWS-FLI1_bridge_target	-0.428811145
ZNF207	170.7501832	100.5900029	3_EWS-FLI1_bridge_target	-0.757553199
PSMD11	50.93202921	33.49049823	4_EWS-FLI1_loop_and_bridge_target	-0.590425629
CDK5R1	9.173887632	4.766300571	4_EWS-FLI1_loop_and_bridge_target	-0.81915312
TMEM98	81.78760774	61.53792957	3_EWS-FLI1_bridge_target	-0.404683373
LIG3	12.88998981	8.385398344	4_EWS-FLI1_loop_and_bridge_target	-0.565555657
SLFN5	8.183118618	12.35393724	4_EWS-FLI1_loop_and_bridge_target	0.540209079
SLFN11	100.940546	38.09321963	4_EWS-FLI1_loop_and_bridge_target	-1.382737674
SLFN12	0.924288465	0.324935317	1_EWS-FLI1_in_promoter	-0.538403156
SLFN12L	1.432963772	0.020889018	4_EWS-FLI1_loop_and_bridge_target	-1.2528888
LOC105371743	17.47592412	7.919064884	4_EWS-FLI1_loop_and_bridge_target	-1.050682162
AP2B1	20.92289104	40.0748697	4_EWS-FLI1_loop_and_bridge_target	0.905817935
TAF15	12.29008132	15.26301755	4_EWS-FLI1_loop_and_bridge_target	0.291245038
ORMDL3	13.15954134	26.93690714	3_EWS-FLI1_bridge_target	0.980397776
PSMD3	128.6818449	100.2799444	3_EWS-FLI1_bridge_target	-0.356628004
MSL1	35.68134777	50.84749647	3_EWS-FLI1_bridge_target	0.49922768
WIPF2	6.539512659	6.317720118	4_EWS-FLI1_loop_and_bridge_target	-0.043077036
CDC6	72.01450999	45.1240636	4_EWS-FLI1_loop_and_bridge_target	-0.662663573
RARA	3.367016773	3.001849191	4_EWS-FLI1_loop_and_bridge_target	-0.125981272
RARA-AS1	0.180855885	0.13059721	4_EWS-FLI1_loop_and_bridge_target	-0.062747863
TOP2A	210.3582963	127.9830076	4_EWS-FLI1_loop_and_bridge_target	-0.712509727
SMARCE1	128.3600287	67.39808592	4_EWS-FLI1_loop_and_bridge_target	-0.919364047
KRT10	17.7209792	8.330823643	4_EWS-FLI1_loop_and_bridge_target	-1.004579557
TMEM99	14.41228884	7.810477303	4_EWS-FLI1_loop_and_bridge_target	-0.806789045
KRT12	38.48148648	12.4209858	4_EWS-FLI1_loop_and_bridge_target	-1.556685664
KRTAP2-3	0.027140513	0.707603242	4_EWS-FLI1_loop_and_bridge_target	0.733339251
KRTAP4-7	0	0	4_EWS-FLI1_loop_and_bridge_target	0

KRTAP4-8	0	0.026497413	4_EWS-FLI1_loop_and_bridge_target	0.037729991
KRTAP4-9	0	0.04474048	4_EWS-FLI1_loop_and_bridge_target	0.063144612
KRTAP4-11	0.019713314	0.419561189	4_EWS-FLI1_loop_and_bridge_target	0.477281433
KRTAP4-5	0.184147915	0	1_EWS-FLI1_in_promoter	-0.243849303
KRTAP4-3	0.12273735	0	4_EWS-FLI1_loop_and_bridge_target	-0.167020467
KRT17	5.722164981	19.60451637	4_EWS-FLI1_loop_and_bridge_target	1.615962745
EIF1	95.71230728	70.815474	4_EWS-FLI1_loop_and_bridge_target	-0.42940476
HAP1	54.52885559	0.251792365	1_EWS-FLI1_in_promoter	-5.471170476
JUP	12.32649668	7.551231312	4_EWS-FLI1_loop_and_bridge_target	-0.640093492
P3H4	9.873347165	11.02220058	4_EWS-FLI1_loop_and_bridge_target	0.144904879
FKBP10	0.232600689	0.473709032	4_EWS-FLI1_loop_and_bridge_target	0.257746205
KLHL11	16.70219601	7.338338411	4_EWS-FLI1_loop_and_bridge_target	-1.086096512
ACLY	156.5918622	175.5034659	4_EWS-FLI1_loop_and_bridge_target	0.163503475
TTC25	1.087201003	0.640141229	3_EWS-FLI1_bridge_target	-0.347749495
CNP	30.62940422	16.62003433	4_EWS-FLI1_loop_and_bridge_target	-0.844049646
DNAJC7	75.93371147	64.64989148	4_EWS-FLI1_loop_and_bridge_target	-0.228823283
NKIRAS2	18.78290098	13.68361421	4_EWS-FLI1_loop_and_bridge_target	-0.430046885
DHX58	0.68478718	1.490352047	4_EWS-FLI1_loop_and_bridge_target	0.563783339
KAT2A	32.93940759	20.3769019	4_EWS-FLI1_loop_and_bridge_target	-0.666908601
KCNH4	0.792796305	0.050180502	4_EWS-FLI1_loop_and_bridge_target	-0.771574265
STAT3	30.18219294	60.49636171	4_EWS-FLI1_loop_and_bridge_target	0.979778667
PTRF	13.01584002	117.8223416	4_EWS-FLI1_loop_and_bridge_target	3.083676007
MLX	19.81581583	8.226433027	3_EWS-FLI1_bridge_target	-1.173835193
FZD2	10.64151145	16.93319957	3_EWS-FLI1_bridge_target	0.623354532
EFTUD2	113.3650227	36.40914701	3_EWS-FLI1_bridge_target	-1.612182912
CCDC103	5.533496424	2.106458377	3_EWS-FLI1_bridge_target	-1.072584538
DCAKD	13.44736012	6.813849113	3_EWS-FLI1_bridge_target	-0.8867006
HEXIM1	5.15873932	5.044874765	4_EWS-FLI1_loop_and_bridge_target	-0.026922614
HEXIM2	1.613780307	1.243573308	4_EWS-FLI1_loop_and_bridge_target	-0.220339561
LOC105371795	0.225817547	0.192244248	4_EWS-FLI1_loop_and_bridge_target	-0.040064438
MAP3K14-AS1	0.573490832	0.88905607	4_EWS-FLI1_loop_and_bridge_target	0.263696751
MAP3K14	0.626428447	1.412791621	4_EWS-FLI1_loop_and_bridge_target	0.568995969
GOSR2	17.76871127	21.22885657	3_EWS-FLI1_bridge_target	0.244104146
NFE2L1	43.37713221	67.09190155	3_EWS-FLI1_bridge_target	0.617666784
CBX1	95.93404466	74.7321497	4_EWS-FLI1_loop_and_bridge_target	-0.356097569
SNX11	17.68935452	17.33367622	3_EWS-FLI1_bridge_target	-0.027720642
HOXB2	0.072171401	0.092162037	3_EWS-FLI1_bridge_target	0.026651357
HOXB-AS3	0.949841057	1.071775299	4_EWS-FLI1_loop_and_bridge_target	0.087511013
HOXB5	0.279363959	0.46620022	4_EWS-FLI1_loop_and_bridge_target	0.19665538
HOXB6	1.93847387	1.526942382	4_EWS-FLI1_loop_and_bridge_target	-0.2176743
HOXB7	20.51679701	7.818120405	4_EWS-FLI1_loop_and_bridge_target	-1.286920253
HOXB8	0.279057471	0.148480473	4_EWS-FLI1_loop_and_bridge_target	-0.155354762
PRAC1	136.4480415	21.33057851	4_EWS-FLI1_loop_and_bridge_target	-2.621793818
PRAC2	5.472964927	2.453235581	4_EWS-FLI1_loop_and_bridge_target	-0.906477928
HOXB13	30.41271444	8.040318068	4_EWS-FLI1_loop_and_bridge_target	-1.796903177
TTLL6	11.20934298	5.506520563	4_EWS-FLI1_loop_and_bridge_target	-0.90802741
LOC105371814	1.488315103	0.566833989	4_EWS-FLI1_loop_and_bridge_target	-0.66731686
ATP5G1	234.7808951	42.58656484	4_EWS-FLI1_loop_and_bridge_target	-2.435491413
UBE2Z	45.81839464	46.84117819	4_EWS-FLI1_loop_and_bridge_target	0.031177453
SNF8	63.14170073	28.89408953	4_EWS-FLI1_loop_and_bridge_target	-1.101402336
IGF2BP1	41.32813901	16.48762439	4_EWS-FLI1_loop_and_bridge_target	-1.275282742
PHOSPHO1	27.14083737	0.164199413	1_EWS-FLI1_in_promoter	-4.595255158
ZNF652	4.320968569	6.325143632	4_EWS-FLI1_loop_and_bridge_target	0.461168167
LOC102724596	0.544436824	0.389755462	4_EWS-FLI1_loop_and_bridge_target	-0.152249806
SLC35B1	51.34297633	48.8632134	4_EWS-FLI1_loop_and_bridge_target	-0.070020102
KAT7	29.43739203	20.81763716	4_EWS-FLI1_loop_and_bridge_target	-0.480349883

DLX4	1.676644116	1.624907793	4_EWS-FLI1_loop_and_bridge_target	-0.028158587
PPP1R9B	20.7548344	17.82826935	2_EWS-FLI1_loop_target	-0.208435638
MRPL27	113.4147588	74.25933154	3_EWS-FLI1_bridge_target	-0.604330782
EME1	15.49112206	9.826180093	3_EWS-FLI1_bridge_target	-0.607165271
TOM1L1	29.10966941	14.7347485	2_EWS-FLI1_loop_target	-0.936272748
COX11	30.54596505	12.66300304	4_EWS-FLI1_loop_and_bridge_target	-1.207180873
STXBP4	7.926546972	4.568583125	4_EWS-FLI1_loop_and_bridge_target	-0.680791916
TMEM100	8.066187632	3.781769599	4_EWS-FLI1_loop_and_bridge_target	-0.922951401
TRIM25	12.18383161	3.79347703	2_EWS-FLI1_loop_target	-1.459625297
COIL	41.37766484	25.98575651	3_EWS-FLI1_bridge_target	-0.65110596
LRRC37A3	0.284210926	1.56263266	4_EWS-FLI1_loop_and_bridge_target	0.996744512
CEP112	4.320899293	2.918438279	4_EWS-FLI1_loop_and_bridge_target	-0.441391326
PRKAR1A	49.01697897	86.62367914	3_EWS-FLI1_bridge_target	0.80890287
ABCA8	0.225306034	2.39965905	4_EWS-FLI1_loop_and_bridge_target	1.472247942
CBX2	46.21966285	19.93060305	3_EWS-FLI1_bridge_target	-1.173773862
CBX8	23.53503773	13.25863565	3_EWS-FLI1_bridge_target	-0.783007547
TBC1D16	10.66784226	6.752139128	3_EWS-FLI1_bridge_target	-0.589871419
TEPSIN	2.644010465	2.501612954	3_EWS-FLI1_bridge_target	-0.057507476
C17orf89	69.5363197	18.34559397	3_EWS-FLI1_bridge_target	-1.866361279
TMEM105	0	0.029656238	3_EWS-FLI1_bridge_target	0.042162759
LOC100130370	0.135667178	0.529360955	4_EWS-FLI1_loop_and_bridge_target	0.429388851
BAHCC1	1.49341139	1.191021222	4_EWS-FLI1_loop_and_bridge_target	-0.186517476
ACTG1	522.4147441	644.5408069	4_EWS-FLI1_loop_and_bridge_target	0.302553732
NPLOC4	39.42928296	55.1619423	3_EWS-FLI1_bridge_target	0.474192214
TSPAN10	0.344647851	0.378220635	3_EWS-FLI1_bridge_target	0.035578468
OXLD1	12.68773223	3.740242651	3_EWS-FLI1_bridge_target	-1.529850625
CCDC137	31.29051332	15.30366356	3_EWS-FLI1_bridge_target	-0.985914189
ARL16	39.78160552	31.81411359	3_EWS-FLI1_bridge_target	-0.313602111
HGS	34.03201115	28.60635811	3_EWS-FLI1_bridge_target	-0.242766779
ARHGDIA	9.76108195	11.41520842	4_EWS-FLI1_loop_and_bridge_target	0.206285343
MYL12A	117.9246804	241.623631	4_EWS-FLI1_loop_and_bridge_target	1.028671924
LOC104968399	0.43884426	0.925017579	4_EWS-FLI1_loop_and_bridge_target	0.419961177
MYL12B	157.0076813	181.9433509	4_EWS-FLI1_loop_and_bridge_target	0.211402288
TGIF1	12.13841196	13.1107692	4_EWS-FLI1_loop_and_bridge_target	0.103005726
DLGAP1-AS1	1.047902572	0.830154417	4_EWS-FLI1_loop_and_bridge_target	-0.162181702
DLGAP1-AS2	0.501055618	0.759812586	4_EWS-FLI1_loop_and_bridge_target	0.229444362
LINC01387	0.230086734	0.244848396	4_EWS-FLI1_loop_and_bridge_target	0.01721001
NDUFV2	121.8470688	83.06935977	4_EWS-FLI1_loop_and_bridge_target	-0.547211443
NDUFV2-AS1	1.121979157	0.728025555	4_EWS-FLI1_loop_and_bridge_target	-0.296285932
ANKRD12	7.60180003	6.339710173	4_EWS-FLI1_loop_and_bridge_target	-0.228915497
RALBP1	35.11841458	31.0077521	4_EWS-FLI1_loop_and_bridge_target	-0.174313207
PPP4R1	45.86664532	65.54032743	4_EWS-FLI1_loop_and_bridge_target	0.505667434
PPP4R1-AS1	0.078786088	0.125761144	4_EWS-FLI1_loop_and_bridge_target	0.061491937
VAPA	36.5254877	37.54067835	4_EWS-FLI1_loop_and_bridge_target	0.038511143
APCDD1	181.8984488	22.91203267	1_EWS-FLI1_in_promoter	-2.935234161
NAPG	30.26623762	36.51347012	4_EWS-FLI1_loop_and_bridge_target	0.262803098
TUBB6	96.92184638	76.8967697	4_EWS-FLI1_loop_and_bridge_target	-0.330067259
PSMG2	72.8826034	55.75381764	3_EWS-FLI1_bridge_target	-0.380517262
CEP76	16.51798559	8.82778776	3_EWS-FLI1_bridge_target	-0.833898281
SS18	60.5941465	52.87656973	2_EWS-FLI1_loop_target	-0.193135254
KCTD1	4.76253424	5.123361491	3_EWS-FLI1_bridge_target	0.087620437
PIK3C3	28.26563251	18.73895078	2_EWS-FLI1_loop_target	-0.568162154
ATP5A1	277.5274581	245.1807764	3_EWS-FLI1_bridge_target	-0.17810145
HAUS1	73.94627193	36.42508701	3_EWS-FLI1_bridge_target	-1.001851045
C18orf32	0.111911515	0.037216067	4_EWS-FLI1_loop_and_bridge_target	-0.100325524
RPL17	20.67813021	14.12325758	4_EWS-FLI1_loop_and_bridge_target	-0.519471394

SNORD58C	16.05813664	15.44789336	4_EWS-FLI1_loop_and_bridge_target	-0.052557245
SNORD58A	21.52284406	13.81792504	4_EWS-FLI1_loop_and_bridge_target	-0.604045574
TCF4	8.882162388	15.31991823	4_EWS-FLI1_loop_and_bridge_target	0.723735161
TXNL1	55.90793661	37.89243101	4_EWS-FLI1_loop_and_bridge_target	-0.549140456
NARS	123.8470757	93.74379768	4_EWS-FLI1_loop_and_bridge_target	-0.398058623
LOC100505549	3.739228669	0.614560826	4_EWS-FLI1_loop_and_bridge_target	-1.55351048
ZNF532	26.99260736	22.56548965	4_EWS-FLI1_loop_and_bridge_target	-0.248370214
SEC11C	110.8983111	51.41213857	4_EWS-FLI1_loop_and_bridge_target	-1.094215381
GRP	174.2838065	79.1278229	4_EWS-FLI1_loop_and_bridge_target	-1.129317536
LMAN1	79.88780137	119.6282586	4_EWS-FLI1_loop_and_bridge_target	0.576573863
CDH20	1.798908414	2.034758287	4_EWS-FLI1_loop_and_bridge_target	0.116717333
PIGN	13.38329576	8.671852133	2_EWS-FLI1_loop_target	-0.572530197
KIAA1468	9.637001871	8.66713489	2_EWS-FLI1_loop_target	-0.137931295
BCL2	3.931381151	2.21911427	2_EWS-FLI1_loop_target	-0.615327975
KDSR	48.3916489	12.12137339	4_EWS-FLI1_loop_and_bridge_target	-1.912348401
VPS4B	36.86583086	29.7538096	4_EWS-FLI1_loop_and_bridge_target	-0.300131451
SERPINB5	0.386419878	0.121494276	4_EWS-FLI1_loop_and_bridge_target	-0.305941987
HMSD	18.85045391	3.208787195	4_EWS-FLI1_loop_and_bridge_target	-2.237695526
SERPINB8	3.261506802	2.252091097	4_EWS-FLI1_loop_and_bridge_target	-0.389995964
DSEL	4.317789155	27.3726313	4_EWS-FLI1_loop_and_bridge_target	2.415601471
LOC643542	0.011596648	0.057589718	4_EWS-FLI1_loop_and_bridge_target	0.064145894
MBD3L3	12.13022917	0.206917483	1_EWS-FLI1_in_promoter	-3.443493149
ZNF557	9.758509033	7.362496599	4_EWS-FLI1_loop_and_bridge_target	-0.363472532
INSR	13.61337497	12.22345652	4_EWS-FLI1_loop_and_bridge_target	-0.144190071
CD320	82.87384821	28.88550507	2_EWS-FLI1_loop_target	-1.488775125
NDUFA7	58.28063865	18.13089159	4_EWS-FLI1_loop_and_bridge_target	-1.631656879
RPS28	191.7391959	122.4243581	4_EWS-FLI1_loop_and_bridge_target	-0.64302285
ANGPTL4	14.90599695	4.391847601	4_EWS-FLI1_loop_and_bridge_target	-1.560719174
RAB11B-AS1	5.384975137	1.34834322	4_EWS-FLI1_loop_and_bridge_target	-1.443037722
RAB11B	2.720019509	2.123661277	4_EWS-FLI1_loop_and_bridge_target	-0.252072168
HNRNPM	73.796763	60.13190043	4_EWS-FLI1_loop_and_bridge_target	-0.291050418
ZNF414	0.704991365	0.671250777	4_EWS-FLI1_loop_and_bridge_target	-0.028836202
ADAMTS10	4.326424092	3.679248306	4_EWS-FLI1_loop_and_bridge_target	-0.186890513
OLFM2	20.39064134	9.623843434	3_EWS-FLI1_bridge_target	-1.009673948
PDE4A	2.261545991	0.750502677	3_EWS-FLI1_bridge_target	-0.897786704
EPOR	4.167596667	2.313503431	1_EWS-FLI1_in_promoter	-0.641136058
LOC284454	7.110477731	5.694070899	3_EWS-FLI1_bridge_target	-0.276903065
AKAP8	12.94875926	13.25166787	3_EWS-FLI1_bridge_target	0.030993967
TPM4	170.8598572	416.7460953	4_EWS-FLI1_loop_and_bridge_target	1.281393742
RAB8A	60.66125238	44.05457534	3_EWS-FLI1_bridge_target	-0.452690571
CHERP	2.131673523	1.709588899	4_EWS-FLI1_loop_and_bridge_target	-0.208859838
MED26	4.05609165	3.300031918	4_EWS-FLI1_loop_and_bridge_target	-0.233675246
SMIM7	64.50453683	29.75501879	4_EWS-FLI1_loop_and_bridge_target	-1.090772974
TMEM38A	4.916457933	5.23188285	4_EWS-FLI1_loop_and_bridge_target	0.074934392
HAUS8	14.09498532	10.99383591	3_EWS-FLI1_bridge_target	-0.331776214
MYO9B	8.452608784	10.64281105	3_EWS-FLI1_bridge_target	0.300654973
ABHD8	3.396641649	3.520369314	3_EWS-FLI1_bridge_target	0.040038696
MRPL34	22.18013535	12.21465934	3_EWS-FLI1_bridge_target	-0.810749756
ANO8	2.330750353	1.623889757	4_EWS-FLI1_loop_and_bridge_target	-0.344140119
GTPBP3	8.136876734	4.342458311	4_EWS-FLI1_loop_and_bridge_target	-0.774197347
MVB12A	10.66996233	6.190199364	3_EWS-FLI1_bridge_target	-0.698696226
RPL18A	203.8919079	167.6301083	3_EWS-FLI1_bridge_target	-0.28100086
SNORA68	53.63659114	34.32065736	3_EWS-FLI1_bridge_target	-0.629355281
KCNN1	9.207712584	1.185445902	4_EWS-FLI1_loop_and_bridge_target	-2.223660042
ARRDC2	7.604522813	4.549628224	4_EWS-FLI1_loop_and_bridge_target	-0.632704059
PIK3R2	7.112774646	8.249408973	3_EWS-FLI1_bridge_target	0.189165768

MPV17L2	8.496859105	5.744110908	3_EWS-FLI1_bridge_target	-0.493822188
RAB3A	0.901224138	0.172136089	3_EWS-FLI1_bridge_target	-0.697788542
LOC102725254	0.207947093	0.265546012	3_EWS-FLI1_bridge_target	0.067202693
JUND	10.60180595	19.77481334	4_EWS-FLI1_loop_and_bridge_target	0.84048612
SSBP4	16.22671562	10.57547766	4_EWS-FLI1_loop_and_bridge_target	-0.573575943
KXD1	38.92089635	35.20184782	3_EWS-FLI1_bridge_target	-0.141080776
COPE	66.32265324	74.52702592	3_EWS-FLI1_bridge_target	0.165900943
DDX49	39.13147494	30.23637639	3_EWS-FLI1_bridge_target	-0.361507078
URI1	67.02381136	48.3152702	1_EWS-FLI1_in_promoter	-0.464005404
TSHZ3	33.8572067	6.789160601	4_EWS-FLI1_loop_and_bridge_target	-2.161917193
LOC101927411	0.147310179	0.016947151	4_EWS-FLI1_loop_and_bridge_target	-0.174010774
ZNF507	6.759148098	4.568827283	4_EWS-FLI1_loop_and_bridge_target	-0.478524715
LOC400684	0.717297208	0.284626605	4_EWS-FLI1_loop_and_bridge_target	-0.418790663
DPY19L3	12.30537424	14.69517642	4_EWS-FLI1_loop_and_bridge_target	0.238312156
PDCD5	273.2159018	148.8657977	4_EWS-FLI1_loop_and_bridge_target	-0.871641066
DYRK1B	2.087432797	3.896947997	4_EWS-FLI1_loop_and_bridge_target	0.665475141
FBL	236.0802465	163.4218024	4_EWS-FLI1_loop_and_bridge_target	-0.527973849
PSMC4	86.18839885	62.30240345	4_EWS-FLI1_loop_and_bridge_target	-0.461875908
ZNF546	2.225373265	1.615551016	4_EWS-FLI1_loop_and_bridge_target	-0.30235122
ZNF780A	6.146713308	6.565651967	4_EWS-FLI1_loop_and_bridge_target	0.082184496
MAP3K10	1.965112135	1.765334842	2_EWS-FLI1_loop_target	-0.100632485
AKT2	22.3492904	14.27221298	4_EWS-FLI1_loop_and_bridge_target	-0.612469579
PRX	1.264118915	1.354159266	3_EWS-FLI1_bridge_target	0.056262194
SERTAD1	12.38770541	7.173712277	4_EWS-FLI1_loop_and_bridge_target	-0.711845346
SERTAD3	16.42348555	30.29375474	4_EWS-FLI1_loop_and_bridge_target	0.844841506
BLVRB	40.8852165	27.00762185	4_EWS-FLI1_loop_and_bridge_target	-0.580621642
SPTBN4	0.142957654	0.094239439	4_EWS-FLI1_loop_and_bridge_target	-0.062843493
SHKBP1	12.69115618	9.327869752	3_EWS-FLI1_bridge_target	-0.406701571
LTBP4	2.286471076	4.043977427	4_EWS-FLI1_loop_and_bridge_target	0.618022532
NUMBL	2.82313396	6.045604814	4_EWS-FLI1_loop_and_bridge_target	0.881967805
C19orf54	0.642558784	0.902772844	4_EWS-FLI1_loop_and_bridge_target	0.212158338
SNRPA	52.94749142	29.96225149	4_EWS-FLI1_loop_and_bridge_target	-0.801045491
MIA	0.051769515	0	4_EWS-FLI1_loop_and_bridge_target	-0.072818587
EGLN2	0.141773316	0.088440483	3_EWS-FLI1_bridge_target	-0.06901373
AXL	0.302024056	6.596253256	4_EWS-FLI1_loop_and_bridge_target	2.544531902
HNRNPUL1	42.66888488	37.20487623	4_EWS-FLI1_loop_and_bridge_target	-0.192848905
CCDC97	4.472302234	2.637021644	4_EWS-FLI1_loop_and_bridge_target	-0.589390401
B9D2	6.549103099	2.876018704	4_EWS-FLI1_loop_and_bridge_target	-0.961729717
TMEM91	4.712493227	2.170211948	4_EWS-FLI1_loop_and_bridge_target	-0.849541253
EXOSC5	31.5185807	11.69650402	4_EWS-FLI1_loop_and_bridge_target	-1.356832985
BCKDHA	20.16481952	13.33702599	4_EWS-FLI1_loop_and_bridge_target	-0.561922398
ATP5SL	40.1404588	26.86403587	4_EWS-FLI1_loop_and_bridge_target	-0.562153648
RPS19	479.2699121	373.5599423	4_EWS-FLI1_loop_and_bridge_target	-0.358648812
CD79A	9.126331775	0.088022804	1_EWS-FLI1_in_promoter	-3.218330959
ARHGEF1	18.86792454	8.602930105	1_EWS-FLI1_in_promoter	-1.04889459
RABAC1	66.6490275	53.77312224	3_EWS-FLI1_bridge_target	-0.304601074
DEDD2	14.64704369	7.642675972	2_EWS-FLI1_loop_target	-0.856340126
ZNF526	8.448338401	6.236519138	2_EWS-FLI1_loop_target	-0.384764728
GSK3A	16.88381522	11.16304738	3_EWS-FLI1_bridge_target	-0.556149813
CIC	2.993819341	6.020423664	4_EWS-FLI1_loop_and_bridge_target	0.813789021
ZNF180	10.59122701	11.21707041	2_EWS-FLI1_loop_target	0.075865082
BCL3	0.614068659	0.633663445	4_EWS-FLI1_loop_and_bridge_target	0.017408852
NECTIN2	11.78824442	24.07381958	4_EWS-FLI1_loop_and_bridge_target	0.97136356
TOMM40	81.34364071	60.40523377	4_EWS-FLI1_loop_and_bridge_target	-0.42329561
CCDC8	32.63606756	10.91605972	4_EWS-FLI1_loop_and_bridge_target	-1.497101789
CARD8	6.905834247	6.028054122	3_EWS-FLI1_bridge_target	-0.169792407

CARD8-AS1	3.310119414	3.798268426	3_EWS-FLI1_bridge_target	0.154786027
CCDC114	0.38060776	0.233081225	4_EWS-FLI1_loop_and_bridge_target	-0.163035663
EMP3	75.49213414	157.5532951	4_EWS-FLI1_loop_and_bridge_target	1.051584556
CYTH2	16.54096128	16.13599823	3_EWS-FLI1_bridge_target	-0.033697576
RPL18	322.5197993	279.3770572	3_EWS-FLI1_bridge_target	-0.206485706
SPHK2	3.213017213	1.137182669	3_EWS-FLI1_bridge_target	-0.979143588
DBP	5.349993001	2.099294451	4_EWS-FLI1_loop_and_bridge_target	-1.034815176
CA11	7.330552265	5.816082808	4_EWS-FLI1_loop_and_bridge_target	-0.289469278
SEC1P	0.004364612	0.027867796	4_EWS-FLI1_loop_and_bridge_target	0.033371614
MAMSTR	0.75964904	0.77066113	4_EWS-FLI1_loop_and_bridge_target	0.009000421
IZUMO1	0.479460952	0.131199866	4_EWS-FLI1_loop_and_bridge_target	-0.387217765
PPP1R15A	16.2001868	21.98441651	4_EWS-FLI1_loop_and_bridge_target	0.418231809
BAX	18.97739514	14.96234774	4_EWS-FLI1_loop_and_bridge_target	-0.323695623
FTL	736.4678127	799.8067017	4_EWS-FLI1_loop_and_bridge_target	0.118873964
GYS1	13.67418243	4.1046113	4_EWS-FLI1_loop_and_bridge_target	-1.523407112
RUVBL2	78.96432283	47.31483256	4_EWS-FLI1_loop_and_bridge_target	-0.726890304
SNRNP70	67.63483906	49.30722189	4_EWS-FLI1_loop_and_bridge_target	-0.448175553
LIN7B	9.326055304	2.330356767	4_EWS-FLI1_loop_and_bridge_target	-1.632540589
C19orf73	1.507392827	0.42996563	4_EWS-FLI1_loop_and_bridge_target	-0.810207565
PPFIA3	14.32188025	4.927160891	4_EWS-FLI1_loop_and_bridge_target	-1.370180226
TEAD2	95.57902258	37.0694476	4_EWS-FLI1_loop_and_bridge_target	-1.343076227
DKKL1	1.096566679	0.985650068	4_EWS-FLI1_loop_and_bridge_target	-0.078417316
SLC17A7	1.052843959	0.095088553	4_EWS-FLI1_loop_and_bridge_target	-0.906576433
PIH1D1	76.92440288	41.51241447	4_EWS-FLI1_loop_and_bridge_target	-0.874190996
ALDH16A1	13.39701491	6.120125968	4_EWS-FLI1_loop_and_bridge_target	-1.015795043
RPL13A	146.9139869	96.16643801	4_EWS-FLI1_loop_and_bridge_target	-0.606228494
SNORD32A	82.3429914	58.36722929	4_EWS-FLI1_loop_and_bridge_target	-0.4893941
SNORD33	19.5019298	11.71068577	4_EWS-FLI1_loop_and_bridge_target	-0.689717845
SNORD34	25.05069316	18.08261049	4_EWS-FLI1_loop_and_bridge_target	-0.449063216
SNORD35A	63.99354174	27.75470448	4_EWS-FLI1_loop_and_bridge_target	-1.176498357
RPS11	947.3027899	719.0958597	4_EWS-FLI1_loop_and_bridge_target	-0.397158809
SNORD35B	51.82902033	28.56705334	4_EWS-FLI1_loop_and_bridge_target	-0.837340186
FCGRT	310.7999227	17.08219619	1_EWS-FLI1_in_promoter	-4.107978753
PRR12	0.384673669	0.283534654	4_EWS-FLI1_loop_and_bridge_target	-0.109423764
RRAS	10.91692713	17.46965987	4_EWS-FLI1_loop_and_bridge_target	0.632145024
SCAF1	4.004965551	4.655582479	4_EWS-FLI1_loop_and_bridge_target	0.176315479
IRF3	18.13658681	9.879632765	4_EWS-FLI1_loop_and_bridge_target	-0.814703674
BCL2L12	4.894358118	5.428072889	4_EWS-FLI1_loop_and_bridge_target	0.125051573
PRMT1	224.2027206	123.1461727	4_EWS-FLI1_loop_and_bridge_target	-0.859184471
AP2A1	12.03107355	16.59355212	4_EWS-FLI1_loop_and_bridge_target	0.433090846
FUZ	19.53258367	5.619481152	4_EWS-FLI1_loop_and_bridge_target	-1.633125132
MED25	4.608952509	3.980432337	4_EWS-FLI1_loop_and_bridge_target	-0.171460384
PTOV1-AS1	4.639814677	5.642028586	4_EWS-FLI1_loop_and_bridge_target	0.235976175
PTOV1	39.13661922	29.24760067	4_EWS-FLI1_loop_and_bridge_target	-0.408098393
PNKP	13.90647696	6.183986972	4_EWS-FLI1_loop_and_bridge_target	-1.053082689
AKT1S1	3.380211088	6.695464792	4_EWS-FLI1_loop_and_bridge_target	0.813008068
TBC1D17	3.104934144	4.932864216	4_EWS-FLI1_loop_and_bridge_target	0.531369688
IL4I1	0.120436025	0.044364057	4_EWS-FLI1_loop_and_bridge_target	-0.101435564
NUP62	37.97298942	26.02155248	4_EWS-FLI1_loop_and_bridge_target	-0.528364033
ATF5	7.419303132	5.088714108	4_EWS-FLI1_loop_and_bridge_target	-0.467563252
VRK3	12.27328852	17.55076901	4_EWS-FLI1_loop_and_bridge_target	0.482953144
ZNF473	17.32725707	16.11244374	4_EWS-FLI1_loop_and_bridge_target	-0.098945085
NR1H2	12.08186541	10.93198297	4_EWS-FLI1_loop_and_bridge_target	-0.132734453
POLD1	19.69918889	9.773491503	4_EWS-FLI1_loop_and_bridge_target	-0.942088358
JOSD2	1.732612783	2.612010227	2_EWS-FLI1_loop_target	0.402520936
ASPDH	0	0.068516645	2_EWS-FLI1_loop_target	0.095609382

CLEC11A	6.04799415	4.634630346	4_EWS-FLI1_loop_and_bridge_target	-0.32289176
C19orf48	92.59782216	31.27401354	2_EWS-FLI1_loop_target	-1.536101961
SNORD88C	19.66707869	19.91412583	2_EWS-FLI1_loop_target	0.01714322
ZBTB45	1.796016836	0.996683772	3_EWS-FLI1_bridge_target	-0.485767186
CHMP2A	45.54434754	42.26701339	3_EWS-FLI1_bridge_target	-0.105338436
UBE2M	63.20330852	44.19296648	3_EWS-FLI1_bridge_target	-0.506549385
MZF1-AS1	0.896199307	1.439987615	3_EWS-FLI1_bridge_target	0.363763213
MZF1	1.65452133	1.040396123	3_EWS-FLI1_bridge_target	-0.379602469
CENPBD1P1	18.00895271	16.40238743	3_EWS-FLI1_bridge_target	-0.127393807
NRSN2-AS1	2.836553491	3.486323238	4_EWS-FLI1_loop_and_bridge_target	0.2257227
SOX12	3.733154789	2.604418428	4_EWS-FLI1_loop_and_bridge_target	-0.393035604
NRSN2	11.0879048	9.504197024	3_EWS-FLI1_bridge_target	-0.202598322
SDCBP2	0.186897376	0.137164235	2_EWS-FLI1_loop_target	-0.061754569
SDCBP2-AS1	1.506389121	2.383999942	2_EWS-FLI1_loop_target	0.433119131
SIRPA	7.639914907	7.539340305	2_EWS-FLI1_loop_target	-0.016892483
LOC727993	0.345975088	0.020082098	1_EWS-FLI1_in_promoter	-0.39996644
STK35	16.46180679	8.557462886	2_EWS-FLI1_loop_target	-0.869503246
SNRPB	344.5895069	180.4034697	4_EWS-FLI1_loop_and_bridge_target	-0.929857363
ZNF343	8.47434754	7.968148997	4_EWS-FLI1_loop_and_bridge_target	-0.079216347
NOP56	196.0032873	100.7798174	4_EWS-FLI1_loop_and_bridge_target	-0.952768196
SNORD110	64.59224883	28.05228069	4_EWS-FLI1_loop_and_bridge_target	-1.174873915
SNORA51	18.1310961	8.948095914	4_EWS-FLI1_loop_and_bridge_target	-0.943427212
SNORD56	17.42420911	11.61046952	4_EWS-FLI1_loop_and_bridge_target	-0.546980698
SNORD57	27.29883229	15.37953985	4_EWS-FLI1_loop_and_bridge_target	-0.788847695
IDH3B	87.88136093	55.73837553	3_EWS-FLI1_bridge_target	-0.647556061
ADAM33	0.084065863	0.253467937	4_EWS-FLI1_loop_and_bridge_target	0.209472683
C20orf27	32.76312429	15.86931571	4_EWS-FLI1_loop_and_bridge_target	-1.001046958
CENPB	62.20198388	29.65435219	4_EWS-FLI1_loop_and_bridge_target	-1.043877927
CDC25B	18.97954871	7.913299517	4_EWS-FLI1_loop_and_bridge_target	-1.164492505
LOC101929125	1.342878736	1.795005784	4_EWS-FLI1_loop_and_bridge_target	0.254568985
AP5S1	18.07425208	7.156194054	4_EWS-FLI1_loop_and_bridge_target	-1.225658483
PANK2	8.389842757	7.826171598	4_EWS-FLI1_loop_and_bridge_target	-0.089313202
RNF24	4.827420156	3.184225807	4_EWS-FLI1_loop_and_bridge_target	-0.477896622
PRNP	43.15022855	123.8125601	4_EWS-FLI1_loop_and_bridge_target	1.499270311
PRND	3.977073128	1.103000184	4_EWS-FLI1_loop_and_bridge_target	-1.242848609
TMEM230	74.02809938	36.90404126	3_EWS-FLI1_bridge_target	-0.985079338
PCNA	121.0306881	96.00587097	3_EWS-FLI1_bridge_target	-0.331100031
C20orf196	2.928142905	1.305972099	3_EWS-FLI1_bridge_target	-0.768472358
TRMT6	35.05964975	18.36902384	4_EWS-FLI1_loop_and_bridge_target	-0.896634137
MCM8	44.73141434	19.26522768	4_EWS-FLI1_loop_and_bridge_target	-1.174179154
FERMT1	17.87138739	6.074186736	4_EWS-FLI1_loop_and_bridge_target	-1.415564284
MKKS	21.86353919	18.35554325	4_EWS-FLI1_loop_and_bridge_target	-0.240301944
SLX4IP	4.407696259	6.023949097	4_EWS-FLI1_loop_and_bridge_target	0.377268269
JAG1	4.613542621	69.10543332	4_EWS-FLI1_loop_and_bridge_target	3.642542834
FLRT3	25.12348153	25.62237455	4_EWS-FLI1_loop_and_bridge_target	0.027292075
SNRPB2	101.5600243	65.32416094	4_EWS-FLI1_loop_and_bridge_target	-0.628862085
BFSP1	8.31500882	1.52926498	4_EWS-FLI1_loop_and_bridge_target	-1.880838946
DSTN	94.77280479	221.2680648	4_EWS-FLI1_loop_and_bridge_target	1.21461272
SNX5	76.97596037	66.04368884	4_EWS-FLI1_loop_and_bridge_target	-0.217927886
MGME1	32.35684462	23.00978846	4_EWS-FLI1_loop_and_bridge_target	-0.474360131
ZNF133	7.946583903	9.173564026	4_EWS-FLI1_loop_and_bridge_target	0.185416351
DZANK1	4.06977415	1.437767268	4_EWS-FLI1_loop_and_bridge_target	-1.056361079
POLR3F	20.13872751	17.16367236	4_EWS-FLI1_loop_and_bridge_target	-0.218832615
SEC23B	37.1201378	42.0595732	4_EWS-FLI1_loop_and_bridge_target	0.175780685
SLC24A3	16.59211407	3.226974677	4_EWS-FLI1_loop_and_bridge_target	-2.05723149
RIN2	1.939375402	12.92877759	4_EWS-FLI1_loop_and_bridge_target	2.244487121

NAA20	67.42827236	52.15890755	3_EWS-FLI1_bridge_target	-0.364281067
XRN2	138.1285042	55.14484576	4_EWS-FLI1_loop_and_bridge_target	-1.309192534
NKX2-4	16.87502863	5.456459352	4_EWS-FLI1_loop_and_bridge_target	-1.469130423
NKX2-2	7.843164508	1.756816668	4_EWS-FLI1_loop_and_bridge_target	-1.681559398
LOC101929625	0.509782116	0.065098581	1_EWS-FLI1_in_promoter	-0.503353395
RBL1	12.72798063	12.16796673	3_EWS-FLI1_bridge_target	-0.060086826
BLCAP	15.94363515	18.44653215	4_EWS-FLI1_loop_and_bridge_target	0.198769477
TTI1	24.87038843	18.99693175	4_EWS-FLI1_loop_and_bridge_target	-0.37152306
RPRD1B	19.49869195	16.9302042	4_EWS-FLI1_loop_and_bridge_target	-0.193139934
MAFB	53.35220061	9.71091252	4_EWS-FLI1_loop_and_bridge_target	-2.343257052
TOP1	33.2391814	16.34441749	3_EWS-FLI1_bridge_target	-0.981176821
SRSF6	78.79000353	60.31660891	4_EWS-FLI1_loop_and_bridge_target	-0.379930099
TOX2	2.120765808	0.627380663	4_EWS-FLI1_loop_and_bridge_target	-0.939348344
OSER1	25.42886872	17.08898737	4_EWS-FLI1_loop_and_bridge_target	-0.547003025
OSER1-AS1	1.530337819	1.415815014	4_EWS-FLI1_loop_and_bridge_target	-0.066820021
PCIF1	6.40896488	4.420548496	3_EWS-FLI1_bridge_target	-0.450833152
ZNF335	9.294674562	8.489837633	3_EWS-FLI1_bridge_target	-0.117442915
ZMYND8	7.178318071	5.725300282	4_EWS-FLI1_loop_and_bridge_target	-0.282205488
LOC101927377	0.172660496	0.108881698	4_EWS-FLI1_loop_and_bridge_target	-0.080679932
NCOA3	13.25162413	15.64551046	3_EWS-FLI1_bridge_target	0.224006774
PREX1	55.59383555	8.310006156	4_EWS-FLI1_loop_and_bridge_target	-2.60379089
ARFGEF2	16.46832894	23.83521223	4_EWS-FLI1_loop_and_bridge_target	0.507645472
CSE1L-AS1	0.162843076	0.216613237	4_EWS-FLI1_loop_and_bridge_target	0.065214187
CSE1L	194.7473433	114.2640023	4_EWS-FLI1_loop_and_bridge_target	-0.764050707
STAU1	41.36829132	34.78380752	4_EWS-FLI1_loop_and_bridge_target	-0.243678044
DDX27	56.92123822	38.39463604	4_EWS-FLI1_loop_and_bridge_target	-0.556093238
ZNFX1	8.359557804	19.53988383	4_EWS-FLI1_loop_and_bridge_target	1.133915746
ZFAS1	159.1779977	122.4737987	4_EWS-FLI1_loop_and_bridge_target	-0.375471059
SNORD12C	36.73451207	31.4599195	4_EWS-FLI1_loop_and_bridge_target	-0.217225198
SNORD12B	57.92154296	27.95194039	4_EWS-FLI1_loop_and_bridge_target	-1.025135168
SNORD12	43.67813166	28.29835333	4_EWS-FLI1_loop_and_bridge_target	-0.608749275
CEBPB-AS1	0.112779648	0.132430616	4_EWS-FLI1_loop_and_bridge_target	0.025254719
CEBPB	24.69784075	3.116764433	4_EWS-FLI1_loop_and_bridge_target	-2.642064339
LINC01272	0	0.01224242	4_EWS-FLI1_loop_and_bridge_target	0.017554839
PTPN1	35.65906869	20.81223389	4_EWS-FLI1_loop_and_bridge_target	-0.749032605
FAM65C	12.7128553	2.341756129	4_EWS-FLI1_loop_and_bridge_target	-2.036850642
LOC100506175	0.03624407	0.057854068	3_EWS-FLI1_bridge_target	0.029776775
PARD6B	10.03113347	5.811899784	4_EWS-FLI1_loop_and_bridge_target	-0.695451923
ADNP	19.90903403	19.25876152	4_EWS-FLI1_loop_and_bridge_target	-0.045580432
ADNP-AS1	2.713840668	2.171931137	4_EWS-FLI1_loop_and_bridge_target	-0.227550471
DPM1	73.97518022	50.45691381	4_EWS-FLI1_loop_and_bridge_target	-0.543048153
MOCS3	9.900955144	5.29834984	4_EWS-FLI1_loop_and_bridge_target	-0.791408751
ZFP64	4.029427879	2.328399513	2_EWS-FLI1_loop_target	-0.595565683
LOC105372672	0.030346085	0.096878991	4_EWS-FLI1_loop_and_bridge_target	0.090275366
PFDN4	59.41558278	49.76298066	4_EWS-FLI1_loop_and_bridge_target	-0.251143924
DOK5	1.471853441	0.684934071	3_EWS-FLI1_bridge_target	-0.552901065
LIPI	48.07159363	3.691795453	1_EWS-FLI1_in_promoter	-3.386676102
RBM11	44.60476512	9.278148632	1_EWS-FLI1_in_promoter	-2.149604156
HSPA13	15.78225576	56.86957195	2_EWS-FLI1_loop_target	1.785868327
NCAM2	20.40083208	1.719359993	4_EWS-FLI1_loop_and_bridge_target	-2.976327836
ATP5J	34.87020835	27.75769814	4_EWS-FLI1_loop_and_bridge_target	-0.318837923
GABPA	15.9170966	11.963882	4_EWS-FLI1_loop_and_bridge_target	-0.383984193
APP	161.4023911	221.6768186	4_EWS-FLI1_loop_and_bridge_target	0.455378502
CYYR1-AS1	0.554421011	0.374544828	4_EWS-FLI1_loop_and_bridge_target	-0.177423348
CYYR1	10.72651326	7.895223526	4_EWS-FLI1_loop_and_bridge_target	-0.398671345
ADAMTS1	14.36593842	134.017264	4_EWS-FLI1_loop_and_bridge_target	3.135336107

CBR3	17.24921138	5.753496923	2_EWS-FLI1_loop_target	-1.434127501
MORC3	20.73501334	25.75185371	3_EWS-FLI1_bridge_target	0.299617883
PIGP	10.10413128	3.749593866	3_EWS-FLI1_bridge_target	-1.225220469
TTC3	47.08138746	69.7612777	3_EWS-FLI1_bridge_target	0.55748157
ETS2	17.93152024	30.75153146	3_EWS-FLI1_bridge_target	0.746035912
WRB	32.75975748	20.16079187	3_EWS-FLI1_bridge_target	-0.673910924
BACE2	13.07927137	11.8985627	2_EWS-FLI1_loop_target	-0.12636236
RIPK4	11.89927832	7.492759292	4_EWS-FLI1_loop_and_bridge_target	-0.602985087
PRDM15	5.140255372	2.581579742	2_EWS-FLI1_loop_target	-0.777702595
ZBTB21	6.88440277	6.874753043	2_EWS-FLI1_loop_target	-0.001766797
ZNF295-AS1	0.07562262	0.143065487	2_EWS-FLI1_loop_target	0.087736059
RRP1	46.98964469	25.00217835	2_EWS-FLI1_loop_target	-0.884090641
TRAPPC10	14.38222849	14.5104822	3_EWS-FLI1_bridge_target	0.011979011
PWP2	57.80219874	16.51932416	3_EWS-FLI1_bridge_target	-1.746922981
C21orf33	36.0775301	23.14322917	4_EWS-FLI1_loop_and_bridge_target	-0.618926492
C21orf2	5.108728122	5.537734851	4_EWS-FLI1_loop_and_bridge_target	0.097918835
UBE2G2	62.28794203	36.33405298	4_EWS-FLI1_loop_and_bridge_target	-0.761438519
LINC01424	1.142643398	0.486380763	4_EWS-FLI1_loop_and_bridge_target	-0.527588026
SUMO3	94.94446433	70.13262317	4_EWS-FLI1_loop_and_bridge_target	-0.431688203
ITGB2	1.393168029	0.706885683	1_EWS-FLI1_in_promoter	-0.487555256
ITGB2-AS1	17.34037382	0.22090028	1_EWS-FLI1_in_promoter	-3.90900577
LINC01547	11.94813557	1.728702512	4_EWS-FLI1_loop_and_bridge_target	-2.246457353
FAM207A	19.19704788	5.483020138	4_EWS-FLI1_loop_and_bridge_target	-1.639406476
SSR4P1	3.754914574	1.608139745	4_EWS-FLI1_loop_and_bridge_target	-0.86639825
ADARB1	11.15569653	4.532282591	4_EWS-FLI1_loop_and_bridge_target	-1.135685806
SLC19A1	9.684958658	4.962146507	2_EWS-FLI1_loop_target	-0.841677594
CECR3	0.060406784	0.160705746	2_EWS-FLI1_loop_target	0.130384472
MICAL3	5.514478241	3.941325868	4_EWS-FLI1_loop_and_bridge_target	-0.398751434
HIRA	19.77587738	20.75337259	3_EWS-FLI1_bridge_target	0.066329686
MRPL40	20.10987868	17.32386379	3_EWS-FLI1_bridge_target	-0.204194543
UFD1L	63.08767257	41.02360714	3_EWS-FLI1_bridge_target	-0.608846875
CDC45	51.54034409	20.87996959	3_EWS-FLI1_bridge_target	-1.263814916
5-Sep	0.254106778	0.09514408	3_EWS-FLI1_bridge_target	-0.1955395
SEPT5-GP1BB	0.653464058	0.387204857	3_EWS-FLI1_bridge_target	-0.25331083
TXNRD2	4.149732972	6.654087001	3_EWS-FLI1_bridge_target	0.571732673
COMT	6.426273813	19.64340131	3_EWS-FLI1_bridge_target	1.474970282
LOC284865	1.557502252	1.044178907	3_EWS-FLI1_bridge_target	-0.323214043
LINC00896	1.090148025	0.708323322	3_EWS-FLI1_bridge_target	-0.291024069
MIATNB	1.436639954	0.790007279	4_EWS-FLI1_loop_and_bridge_target	-0.444927635
LINC01422	0.963488198	0.174519601	1_EWS-FLI1_in_promoter	-0.741348137
MN1	1.475070339	1.260324388	4_EWS-FLI1_loop_and_bridge_target	-0.130939691
MCM5	79.05749905	58.36251463	3_EWS-FLI1_bridge_target	-0.431484341
RBFOX2	36.42561196	31.51368232	4_EWS-FLI1_loop_and_bridge_target	-0.20297895
FOXRED2	9.002188866	4.454846982	3_EWS-FLI1_bridge_target	-0.87470512
JOSD1	38.52269845	53.92465152	3_EWS-FLI1_bridge_target	0.47477236
GTPBP1	10.13695727	10.27293536	3_EWS-FLI1_bridge_target	0.0175081
CBX6	29.30691986	8.128579506	3_EWS-FLI1_bridge_target	-1.73118495
APOBEC3G	3.83924305	7.104441746	3_EWS-FLI1_bridge_target	0.743931413
RPL3	588.2462701	493.0004605	4_EWS-FLI1_loop_and_bridge_target	-0.254358335
SNORD83B	27.22631167	17.59551656	4_EWS-FLI1_loop_and_bridge_target	-0.602085799
SNORD83A	26.0446073	14.2463106	4_EWS-FLI1_loop_and_bridge_target	-0.826880777
SNORD139	16.81724856	11.63252469	4_EWS-FLI1_loop_and_bridge_target	-0.496131565
SNORD43	23.12371676	11.90674053	4_EWS-FLI1_loop_and_bridge_target	-0.902327493
MIEF1	21.99196008	16.80426712	4_EWS-FLI1_loop_and_bridge_target	-0.368906411
ATF4	205.7520254	171.649929	4_EWS-FLI1_loop_and_bridge_target	-0.260051722
RPS19BP1	67.65617916	45.62496106	4_EWS-FLI1_loop_and_bridge_target	-0.558287051

CACNA1I	5.675737318	0.302608823	1_EWS-FLI1_in_promoter	-2.357523284
TNRC6B	6.277232553	7.083390743	4_EWS-FLI1_loop_and_bridge_target	0.151570673
ADSL	119.3332633	104.1814024	4_EWS-FLI1_loop_and_bridge_target	-0.194155859
MKL1	2.586052577	9.862650552	4_EWS-FLI1_loop_and_bridge_target	1.598907627
RANGAP1	57.95472409	31.39360663	3_EWS-FLI1_bridge_target	-0.863898319
POLDIP3	42.89674605	42.97110379	4_EWS-FLI1_loop_and_bridge_target	0.002441748
RNU12	61.22353197	40.13130936	4_EWS-FLI1_loop_and_bridge_target	-0.597223289
PARVG	1.313358215	5.603569508	4_EWS-FLI1_loop_and_bridge_target	1.513257394
LINC01589	0.140998273	0	3_EWS-FLI1_bridge_target	-0.190296608
LOC730668	0.122230122	0.18328332	4_EWS-FLI1_loop_and_bridge_target	0.076427005
PRR34	0.478595445	0.408780798	3_EWS-FLI1_bridge_target	-0.069780224
PRR34-AS1	1.322365454	1.091147782	3_EWS-FLI1_bridge_target	-0.151299996
CDPF1	11.38362598	4.906609229	4_EWS-FLI1_loop_and_bridge_target	-1.06803173
GTSE1-AS1	0.36559236	0.291785736	4_EWS-FLI1_loop_and_bridge_target	-0.080160097
GTSE1	25.03335533	15.49024091	4_EWS-FLI1_loop_and_bridge_target	-0.658748791
FAM19A5	26.13637009	5.431332993	2_EWS-FLI1_loop_target	-2.077038056
C22orf34	0.142439671	0.035475741	4_EWS-FLI1_loop_and_bridge_target	-0.141824228
